# A Ruthenium(CAAC‐5) Olefin Metathesis Catalyst for Bioconjugation

**DOI:** 10.1002/anie.3685338

**Published:** 2026-09-21

**Authors:** Damian Alexander Graf, Mikhail Boym, María Álvarez, Tsvetan Kardashliev, Thomas R. Ward

**Affiliations:** ^1^ Department of Chemistry University of Basel Basel Switzerland; ^2^ Swiss Nanoscience Institute University of Basel Basel Switzerland; ^3^ National Centre of Competence in Research Molecular Systems Engineering (NCCR MSE) University of Basel Basel Switzerland

**Keywords:** artificial metalloenzymes, bioconjugation, carbenes, olefin metathesis, protein modifications

## Abstract

Olefin metathesis is a powerful transformation for carbon–carbon bond formation, yet its application in biological settings is challenged by the instability of most ruthenium catalysts in aqueous media. Herein, we report a Ru(CAAC‐5) olefin metathesis catalyst bearing a functional handle for bioconjugation. The resulting hybrid catalyst catalyzes ring‐closing metathesis (RCM) and displays extended lifetimes in aqueous media. Functionalized CAAC‐5 ligands bearing a protected aniline linker were prepared via enamine alkylation and coordinated to ruthenium to afford the corresponding Ru(CAAC‐5) complex. In a benchmark RCM reaction under biologically relevant conditions, the ruthenium complex exhibits enhanced catalytic robustness and reaches turnover numbers of up to 600. Deprotection of an amine‐functionalized handle enables the bioconjugation to proteins and peptides. Using this platform, the catalyst was anchored to biomolecules through supramolecular anchoring to human carbonic anhydrase II (hCA II), click conjugation to peptides (SS01), and covalent attachment to ubiquitin (Ubi). To the best of our knowledge, this aniline‐functionalized Ru(CAAC‐5) complex is the first CAAC‐bearing metathesis catalyst amenable to bioconjugation, while retaining the prolonged catalytic activity characteristic of CAAC‐based systems in aqueous media.

## Graphical abstract

The aqueous instability of ruthenium catalysts restricts olefin metathesis in biological media. A Ru(CAAC‐5) complex bearing an aniline handle is reported that remains active under biologically relevant conditions and enables bioconjugation to proteins, peptides, and an artificial metalloenzyme, providing a robust platform for olefin metathesis in aqueous and biomolecular environments.

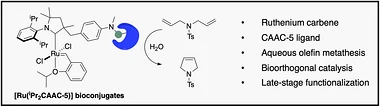

## 1 Introduction

Olefin metathesis has emerged as one of the most powerful transformations in synthetic chemistry for carbon–carbon bond formation [1, 2]. The development of ruthenium‐based catalysts—most notably the first‐ and second‐generation Grubbs and Hoveyda‐Grubbs complexes—has rendered olefin metathesis broadly applicable across ring‐closing metathesis (RCM), ring‐opening metathesis polymerization (ROMP), and cross‐metathesis (CM) [3, 4, 5, 6]. High functional group tolerance and operational simplicity have established olefin metathesis as a cornerstone of modern organic synthesis with applications in the preparation of pharmaceuticals [7, 8], polymers [9], and complex natural products [10].

Ruthenium catalysts bearing an *N*‐heterocyclic carbene ligand (NHC) react selectively with olefins while leaving most polar functional groups untouched [11]. This chemoselectivity has prompted efforts to translate metathesis into aqueous and biological environments [12, 13]. Such bioorthogonal applications offer powerful means for the site‐selective modification of proteins and other biomolecules under physiological conditions [14, 15]. One of the earliest examples of olefin CM in a biological setting is the site‐selective modification of the serine protease subtilisin *Bacillus lentus* (SBL) [16]. In this system, an engineered cysteine residue was functionalized with an allyl sulfide [17] that served as a CM partner for a range of terminal olefins. While efficient protein modification was achieved, the reaction required super‐stoichiometric amounts of the ruthenium catalyst and strictly controlled conditions to suppress catalyst decomposition Figure 1a. Beyond small‐molecule CM tagging, ROMP has enabled protein–polymer bioconjugation, complementing established atom transfer radical polymerization (ATRP)‐ and reversible addition–fragmentation chain transfer (RAFT)‐based strategies [18]. Using a protein‐bound, PEGylated ruthenium complex, water‐soluble norbornenes were polymerized directly from the protein surface in aqueous buffer, affording high‐molecular‐weight conjugates [19].

**FIGURE 1:**
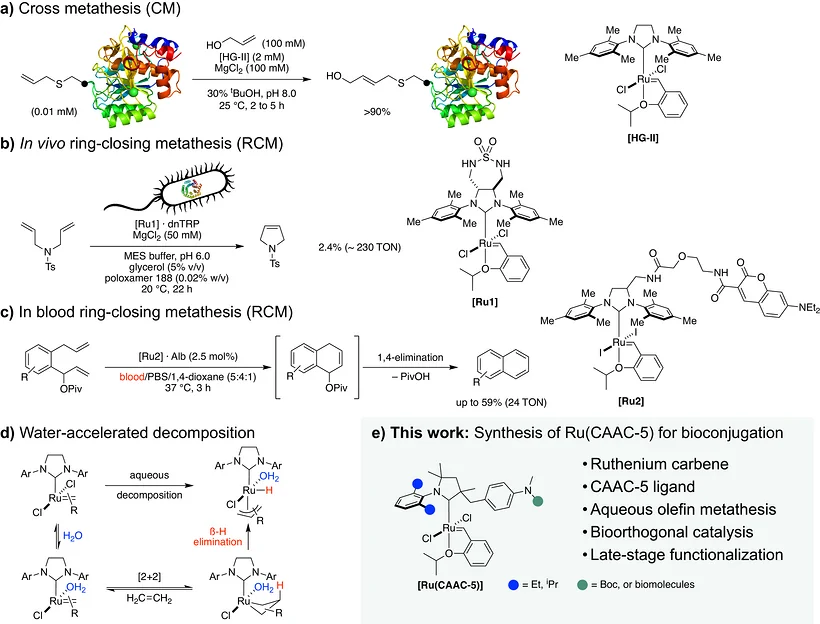
Olefin metathesis in aqueous media. (a) Cross‐metathesis (CM) between subtilisin *Bacillus lentus*  (SBL) and allyl alcohol using super‐stoichiometric amounts of Hoveyda‐Grubbs second generation **[HG‐II]** catalyst. (b) In vivo ring‐closing metathesis (RCM) catalyzed by the artificial metalloenzyme (ArM) **[Ru1] · dnTRP**. The cofactor **[Ru1]** is anchored to an engineered *de novo* tandem‐repeat protein (dnTRP). c) “Close‐to‐release” RCM in the presence of blood. The cofactor **[Ru2]** is anchored to the hydrophobic Albumin (Alb). (d) Water‐accelerated decomposition of Ru(NHC) complexes [20]. (e) This work: Design and synthesis of Ru(CAAC‐5) complexes that can be used for bioconjugation.

More recently, Baker and our groups reported a *de novo*‐designed protein scaffold that binds the ruthenium cofactor **Ru1** [21]. Directed evolution of the protein afforded variants that effectively shield the metal center from water and cellular nucleophiles while maintaining catalytic activity. The resulting artificial metalloenzyme (ArM) displayed improved robustness and enabled RCM in the cytosol of *Escherichia coli*. Under optimized conditions, turnover numbers of up to 230 were achieved with a tosyl‐diolefin model substrate, revealing that the hydrophobic protein pocket can improve catalyst lifetime in vivo, Figure 1b. The protein is genetically encodable and can be expressed in cells, providing a route toward intracellular metathesis catalysis.

Complementary strategies have been developed by Tanaka and coworkers, who demonstrated that modification of the ruthenium coordination sphere and binding to serum albumin (Alb) can protect the catalyst **Ru2** in complex biological media [22]. Substitution of chloride with iodide further strengthens the Ru–halide bond and increases catalyst hydrophobicity [23, 24]. This reduces aquation and favors association with the hydrophobic pocket of albumin (Alb), thereby shielding the metal center from deactivation by water and thiols [25, 26]. Using a “close‐to‐release” diolefin substrate [27], efficient RCM and subsequent product formation were observed in the presence of blood, reaching up to 59% conversion, Figure 1c. Related albumin‐bound systems enabled catalytic activity in vivo in mouse models, revealing that anchoring the metathesis catalyst within the hydrophobic cavity protects it against thiols in cellulo [28]. These studies highlight the importance of the microenvironment around the ruthenium catalyst to achieve metathesis activity in biological settings.

However, the implementation of olefin metathesis in biological environments is severely limited by the instability of most ruthenium catalysts in aqueous media [29, 30, 31, 32]. Mechanistic studies, first by Yoshida and Matsuo [33] and later by Fogg and coworkers [20], have shown that Ru(NHC) catalysts undergo rapid decomposition in the presence of water and other protic nucleophiles. This typically leads to catalyst deactivation via chloride exchange with water, followed by β‐hydride elimination to afford an inactive ruthenium hydride species, Figure 1d [20]. This intrinsic sensitivity limits the lifetime of metathesis catalysts under biologically relevant conditions, thus limiting their use in aqueous buffers [20], cell culture media [24], and protein‐containing systems [34]. Accordingly, maintaining catalytic activity in water is a key challenge in developing the next generation of metathesis catalysts for chemical biology applications.

Efforts to address this limitation have focused on modifying the ruthenium ligand environment to enhance hydrolytic decomposition and nucleophilic stability. In this context, cyclic (alkyl)(amino)carbene (CAAC) ligands have emerged as highly promising alternatives to the classical NHC ligands [35, 36]. Their stronger σ‐donating character and distinct steric profile can impart increased robustness of the corresponding ruthenium complexes and improve resistance toward decomposition pathways that prevail in aqueous media [20, 37]. Numerous CAAC‐based ruthenium complexes, often incorporating a nitro substituent on the benzylidene moiety—a modification introduced by Grela [38, 39] to accelerate initiation at ambient temperature—have since been reported and evaluated in olefin metathesis [37, 40, 41, 42, 43, 44]. While rapid initiation is advantageous for catalytic efficiency, it typically comes at the cost of reduced catalyst longevity in aqueous media, since the active carbene species generated during initiation is also the most susceptible to decomposition [45, 46]. Elevated temperatures, typically 60°C–70°C, are routinely used to sustain useful conversion in the presence of water, whether neat or as a co‐solvent [20, 43, 47]. Notable examples include a CAAC‐5 complex bearing a triethylammonium group for enhanced solubility in protic solvents, reported by Tuba and coworkers [44]. Fogg and coworkers developed a sulfate‐functionalized analog to improve water solubility [43]. More recently, Plenio and coworkers introduced a phenolic handle on the Ru(CAAC) complex, enabling covalent dimerization via flexible linkers to probe bimolecular decomposition in organic solvents [48].

Despite these advances, the activity and stability of CAAC‐containing catalysts remain largely unexplored in complex, biomolecule‐rich environments. To the best of our knowledge, no widely applicable strategy has been reported for bioconjugating a CAAC ligand with peptides and proteins. Accordingly, we set out to develop a modular approach to functionalize the CAAC ligands with various biomolecules, Figure 1e. Ultimately, such ligands bearing a functional handle enable: i) supramolecular anchoring via high‐affinity binders for the assembly of ArMs, ii) click bioconjugation to peptides, and iii) covalent attachment to proteins via cysteine alkylation.

## 2 Results and Discussion

### 2.1 Synthesis of Ru(CAAC‐5) Complexes

The synthesis of the CAAC‐5 ligands was adapted from the pioneering work of Bertrand and coworkers [35]. Classical routes rely on the condensation of functionalized anilines with aldehydes to form aldimine intermediates, followed by cyclization under strongly acidic conditions (typically HCl at high temperatures) to afford the CAAC‐5 scaffold. These harsh conditions significantly limit the range of functional and protecting groups that can be introduced early in the synthesis. To overcome this limitation, we pursued a functionalization strategy based on the deprotonation of the CAAC‐5 salts to the corresponding enamines, Figure 2a [49]. Subsequent alkylation with electrophiles bearing a Boc‐protected aniline linker provided the functionalized ligands **Et_2_CAAC‐5‐Boc** and **^i^Pr_2_CAAC‐5‐Boc** as tetrafluoroborate salts in moderate yields—that is, 44% and 61%, respectively. Both ligands proved compatible with the strongly basic conditions required for carbene generation and ligand exchange with the Hoveyda–Grubbs first‐generation (**HG‐I**) precursor. Complexation in the presence of LiHMDS and **HG‐I** afforded the Boc‐protected ruthenium complexes **[Ru(Et_2_CAAC‐5‐Boc)]** and **[Ru(^i^Pr_2_CAAC‐5‐Boc)]** in moderate yields—that is, 41% and 63%, respectively.

**FIGURE 2:**
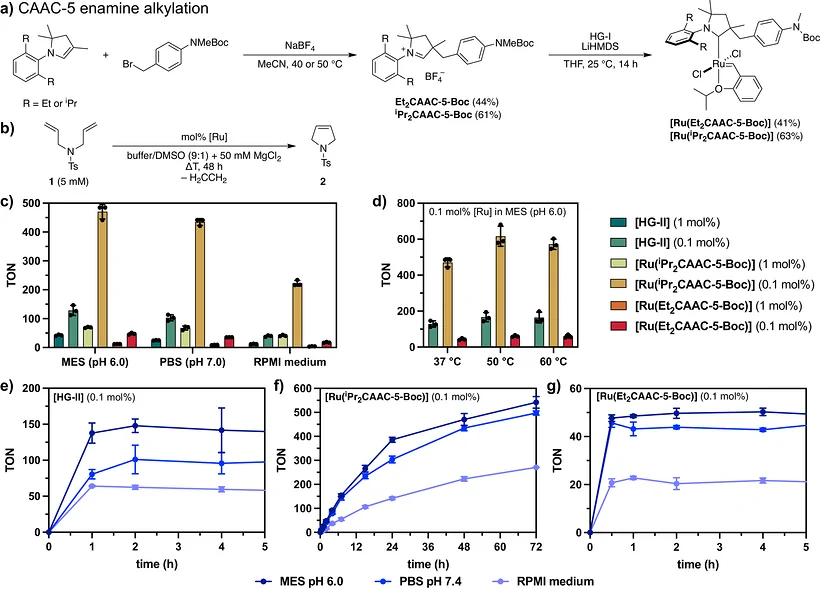
Synthesis and catalytic performance of the aniline functionalized [Ru(CAAC‐5)]‐metathesis catalysts. (a) Synthesis of functionalized CAAC–ruthenium complexes via enamine alkylation with Boc‐protected benzyl bromide, followed by complexation with the Hoveyda–Grubbs first‐generation catalyst (**HG‐I**) to yield the corresponding [Ru(CAAC‐5)] complexes. (b) Benchmark RCM of diolefin **1** (5 mM) to afford cyclic olefin **2**. All reactions were carried out in the presence of 10% DMSO as a cosolvent and 50 mM MgCl_2_. (c) Turnover number (TON) of the ruthenium complexes in different buffers (2‐(*N*‐morpholino)ethanesulfonic acid = MES and phosphate‐buffered saline = PBS) and cell culture medium (Roswell Park Memorial Institute = RPMI) at catalyst loadings of 1.0 and 0.1 mol%. (d) TON determination at different temperatures in MES buffer (pH 6.0). Time‐course monitoring of the RCM reaction at 37°C in MES buffer (pH 6.0) using 0.1 mol% (e) Hoveyda–Grubbs second‐generation catalyst **[HG‐II]**, (f) **[Ru(^i^Pr_2_CAAC‐5‐Boc)]**, and (g) **[Ru(Et_2_CAAC‐5‐Boc)]**. Conversions were determined by UHPLC at 254 nm using 2‐phenylethanol as an internal standard. Data in (c–g) are presented as mean ± standard deviation (SD) of n = 3 independent experiments; error bars represent SD. No statistical hypothesis testing was performed.

### 2.2 Catalytic Activity for RCM

We first examined the catalytic activity of the Boc‐protected complexes using model substrate tosyl‐diolefin **1**, which undergoes RCM to afford the corresponding cyclic olefin **2**, Figure 2b. The reactions were conducted under biologically relevant conditions, using 5 mM of tosyl‐diolefin **1** in various buffers containing 10% DMSO as a cosolvent and 50 mM MgCl_2,_ at 37°C for 48 h. Reactions were performed in triplicate and analyzed by UHPLC using 2‐phenylethanol as an internal standard (see Supporting Information for experimental details).

Analysis of the RCM results reveals several trends, Figure 2c: i) **[Ru(^i^Pr_2_CAAC‐5‐Boc)]** consistently displays the highest catalytic activity across all tested conditions, reaching turnover numbers (TONs) of up to 480 in 2‐(*N*‐morpholino)ethanesulfonic acid buffer (MES, pH 6.0) and outperforming both **HG‐II** and **[Ru(Et_2_CAAC‐5‐Boc)]**. ii) Decreasing the catalyst loading from 1.0 to 0.1 mol% leads to increased TONs for all complexes, suggesting that catalyst stability rather than intrinsic activity limits turnover at higher loadings. iii) All catalysts remain active in a complex environment such as cell culture medium from Roswell Park Memorial Institute (RPMI), albeit with reduced TONs compared to MES and phosphate‐buffered saline (PBS) buffers. iv) Under mildly acidic conditions (MES buffer, pH 6.0), **HG‐II** exhibits high initial catalytic activity that plateaus after approximately 1 h, most likely as a result of catalyst decomposition [20, 50]. v) **[Ru(Et_2_CAAC‐5‐Boc)]** affords uniformly low TONs across all tested buffers and catalyst loadings, reaching only ∼10% of the activity of the ^i^Pr‐substituted analog. Notably, this trend contrasts with earlier reports suggesting that increased steric bulk in CAAC‐5 ligands diminishes catalytic activity in organic solvents [51].

### 2.3 Catalytic Activity in Organic Solvent

To further investigate the origin of the catalytic performance, we evaluated both complexes under conventional metathesis conditions in organic solvents (toluene‐d_8_ and methylene chloride‐d_2_), where solubility is not a limiting factor for either complex, Figures S7–S9. Strikingly, the activity trend observed in aqueous buffer was reversed: **[Ru(^i^Pr_2_CAAC‐5‐Boc)]** was essentially inactive under all organic‐solvent conditions tested (0.1–1.0 mol%, 25°C–50°C), while **[Ru(Et_2_CAAC‐5‐Boc)]** performed comparably to **HG‐II** and **UltraNitroCat** in toluene at elevated temperature (TON up to ∼960 at 37°C–50°C, 0.1 mol%). Together, these results indicate that intrinsic differences in reactivity or solubility do not govern the divergent catalytic performance of the two complexes but rather reflect an environment‐specific effect of the ^i^Pr_2_ substituents that selectively enhances catalytic performance under aqueous, physiologically relevant conditions.

### 2.4 Catalyst Solubility

To rule out limited aqueous solubility as a contributing factor to the observed catalytic performance, the solubility of both ruthenium complexes was assessed by UV‐Vis absorbance as a function of concentration and over time, Figures S10–S12. Both the Et_2_‐ and ^i^Pr_2_‐substituted complexes exhibited Beer–Lambert linearity (380 nm, R^2^> 0.98) upon dilution and precipitated from aqueous buffer with comparable half‐lives (0.6–0.9 h) over 8 h without shaking, ruling out differential aqueous solubility as the origin of the divergent catalytic performance between the two complexes.

### 2.5 Catalyst Stability

The mechanistic origin of the stability difference between **[Ru(^i^Pr_2_CAAC‐5‐Boc)]** and **[Ru(Et_2_CAAC‐5‐Boc)]** and the decomposition of both precatalysts was monitored by ^1^H NMR spectroscopy (DMSO‐d_6_/D_2_O, 4:1) in the absence and presence of tosyl‐diolefin **1**, Figures S13, S14. For both complexes, loss of the Ru‐benzylidene signal (16.30/16.33 ppm) was accompanied by the growth of a diagnostic signal at 10.31 ppm, assigned to 2‐isopropoxybenzaldehyde, consistent with hydrolytic cleavage of the benzylidene ligand, Figure S16 [30, 52]. For **[Ru(Et_2_CAAC‐5‐Boc)]**, this hydrolysis presumably proceeded via an additional intermediate species (17.06 ppm). It was markedly accelerated in the presence of substrate, reaching complete conversion within 18 h (compared to 52% at the same time point without substrate). In contrast, **[Ru(^i^Pr_2_CAAC‐5‐Boc)]** exhibited no analogous intermediate and was less affected by the presence of substrate (9% vs. 16% aldehyde formation at 28 h), Figure S15. These assignments were further corroborated by HRMS‐ESI^+^ analysis of the same samples, Figures S79–S84. The acylium ion of 2‐isopropoxybenzaldehyde was detected in all cases, while the intact complex ([M–Cl]^+^) was no longer detectable for **[Ru(Et_2_CAAC‐5‐Boc)]** but remained clearly observable for **[Ru(^i^Pr_2_CAAC‐5‐Boc)],** directly confirming the greater persistence of the intact ^i^Pr_2_‐substituted complex. This selective, substrate‐induced acceleration of decomposition for the Et_2_‐substituted complex indicates that the two complexes diverge specifically in the stability of the reactive species formed during catalytic turnover. This is consistent with the bulkier ^i^Pr_2_‐substituents providing steric protection against water‐mediated decomposition of the propagating alkylidene intermediate. While benzylidene hydrolysis to the corresponding aldehyde has previously been reported for NHC‐ligated ruthenium catalysts [52], the present data indicate that such a pathway also operates for CAAC‐ligated systems and is selectively suppressed by the more sterically demanding ^i^Pr_2_‐substituents.

### 2.6 Temperature Dependence and Time‐course of the RCM

Next, we examined the influence of temperature on the RCM reaction, Figure 2d. Increasing the temperature from 37°C to 50°C increases TON for all complexes, whereas no further improvement is observed when the temperature is raised to 60°C. To gain insight into the catalyst lifetime, a time‐course study was performed under the same conditions, Figure 2e–g. These results indicate that **HG‐II** initially displays the highest activity, Figure 2e. However, rapid catalyst decomposition occurs, and **HG‐II** is fully deactivated within 1 h at 37°C across all tested buffers. Moreover, the extent of productive turnover depends on the buffer, suggesting that catalyst deactivation occurs at different TON values depending on the medium.

In contrast, **[Ru(^i^Pr_2_CAAC‐5‐Boc)]** exhibits lower initial activity, resulting in slower product formation, but remains active for 72 h under all tested conditions, Figure 2f. This behavior is consistent with improved robustness toward aqueous decomposition. The **[Ru(Et_2_CAAC‐5‐Boc)]** complex exhibits slightly higher initial activity than the ^i^Pr analog but substantially lower robustness, Figure 2g. Like **HG‐II**, **[Ru(Et_2_CAAC‐5‐Boc)]** decomposes after 1 h. Owing to its lower intrinsic activity compared to **HG‐II**, only TON values of up to 50 are reached. Across all tested reactions, increasing the pH results in shorter catalyst lifetimes, consistent with earlier reports on the sensitivity of ruthenium metathesis catalysts to basic aqueous environments [20, 33, 34]. The complex composition of the RPMI medium further compromises catalyst stability, although the RCM reaction still proceeds to a measurable extent.

### 2.7 Bioconjugation of Ru(^i^Pr_2_CAAC‐5)

Since **[Ru(^i^Pr_2_CAAC‐5‐Boc)]** exhibited superior activity under the selected reaction conditions, we focused our efforts on its bioconjugation. The Boc protecting group was readily removed by treatment with gaseous HCl, affording the corresponding amine‐functionalized CAAC–ruthenium complex. This transformation provides direct access to a versatile platform for functionalization, while preserving the integrity of the catalytically active ^i^Pr_2_CAAC‐5–ruthenium core. The free amine was functionalized via amide coupling with an aryl sulfonamide, an alkyl azide, and an alkyl bromide for bioconjugation to proteins and peptides, Figure 3.

**FIGURE 3:**
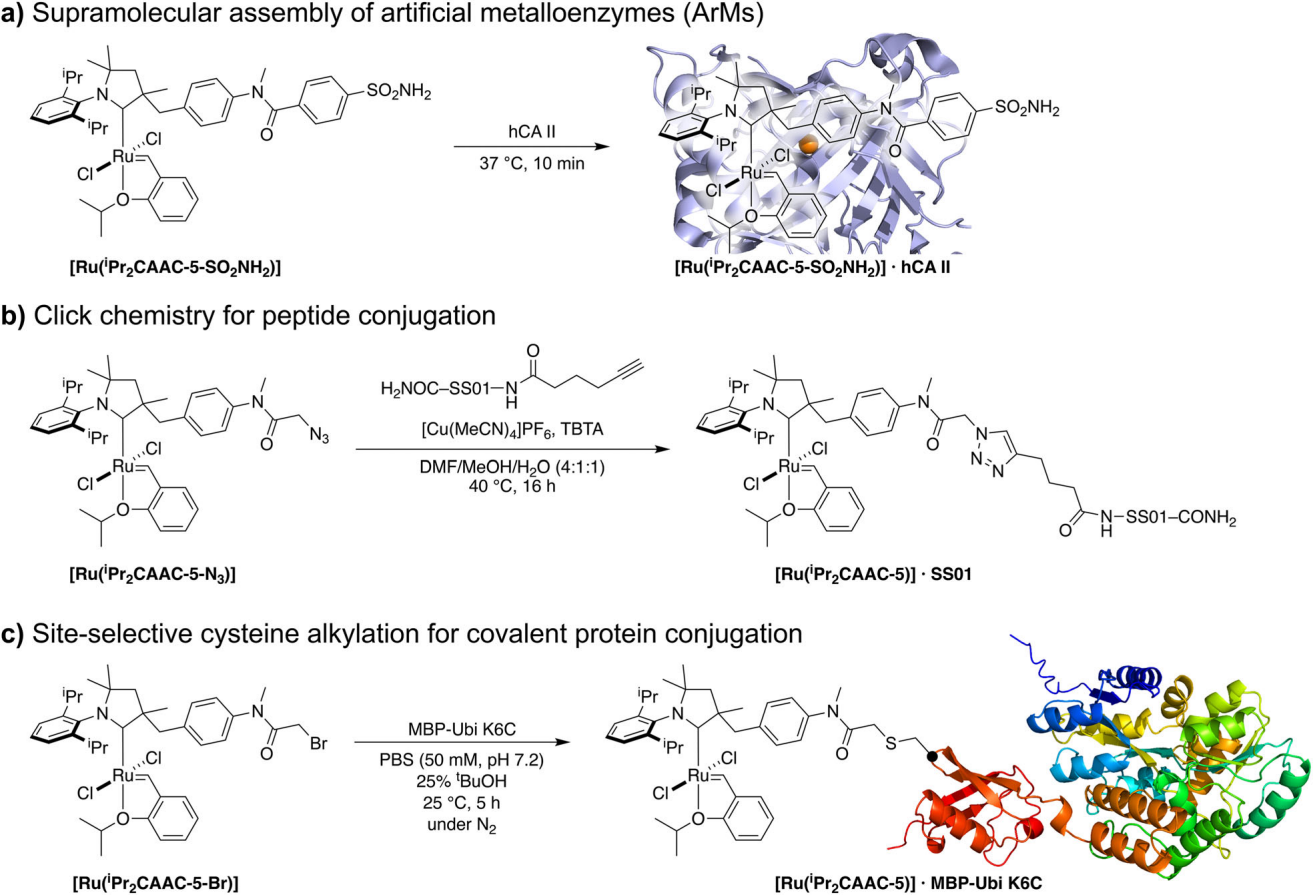
Bioconjugation of [Ru(^i^Pr_2_CAAC‐5)] complexes. (a) Assembly of an artificial metalloenzyme (ArM) via supramolecular anchoring to human carbonic anhydrase II (**hCA II**) using an aryl sulfonamide anchor. (b) Conjugation to somatostatin analog **SS01** via copper‐catalyzed azide–alkyne cycloaddition (CuAAC). (c) Site‐selective cysteine alkylation for covalent attachment to ubiquitin (**Ubi**) mutant K6C, fused with a maltose‐binding protein (**MBP‐Ubi K6C**). Conjugation was performed in phosphate‐buffered saline (PBS) at pH 7.2, with 25% *tert*‐butanol as a cosolvent.

### 2.8 Supramolecular Anchoring to hCA II

As a first approach, we explored the formation of an artificial metalloenzyme (ArM) through supramolecular anchoring to human carbonic anhydrase II (**hCA II**). Installation of an aryl sulfonamide moiety affords a high‐affinity binder for the active site Zn^2+^ ion of **hCA II** [53]. This enables a non‐covalent incorporation of the ruthenium cofactor within a well‐defined protein environment, Figure 3a [54]. The binding affinity of [**Ru(^i^Pr_2_CAAC‐5‐SO_2_NH_2_)]** for **hCA II** was determined by a competitive displacement assay with dansylamide (DNSA, *K_d_* = 1.06 ± 0.20 µM, for details, see Supporting Information Figure S1). Embedding the catalyst within a protein introduces a secondary coordination sphere that can influence the catalyst performance by increasing stability toward deactivation pathways, modulating substrate accessibility, and potentially imparting selectivity [55, 56].

### 2.9 Click Chemistry With SS01 Alkyne

Next, we examined peptide conjugation with the cyclic peptide **SS01**, a somatostatin‐derived antagonist targeting somatostatin receptors (SSTRs) [57]. Cyclic somatostatin analogs are well known for their enhanced metabolic stability, conformational rigidity, and high receptor affinity compared to linear peptides [58]. In contrast to agonists, antagonist variants such as **SS01** exhibit strong receptor binding without inducing internalization, which can be advantageous for applications that require prolonged surface association and targeting [57]. To enable bioorthogonal ligation, **SS01** was functionalized with an *N*‐terminal alkyne handle that allows conjugation to the azide‐functionalized ruthenium complex **[Ru(^i^Pr_2_CAAC‐5‐N_3_)]** via copper‐catalyzed azide–alkyne cycloaddition (CuAAC) [59]. This reaction proceeds under mild conditions and provides a robust and chemoselective route to the triazole‐linked conjugate, Figure 3b. The resulting **Ru(^i^Pr_2_CAAC‐5) · SS01** conjugate was purified by preparative RP‐HPLC and characterized by HPLC (Supporting Information Figure S4) and high‐resolution mass spectrometry (HRMS, Supporting Information Figure S75) to confirm the conjugation and purity.

### 2.10 Cysteine Alkylation of MBP‐Ubi K6C

Finally, we investigated covalent protein conjugation using an engineered ubiquitin (**Ubi**) variant bearing a single cysteine residue at position 6 (K6C), which can be readily expressed as a maltose‐binding protein (**MBP**) fusion construct [60]. As wild‐type Ubi lacks native cysteines [61], this point mutation introduces a thiol handle at a defined, solvent‐exposed position. This enables the site‐selective modification without compromising the overall protein fold. **Ubi** is a particularly attractive protein target in this context, as it plays a central role in cellular protein homeostasis through the Ubi‐proteasome system [62]. Ubi‐catalyst conjugates could therefore serve as tools to deliver functional metal complexes to Ubi‐binding domains or proteasomal receptors. This would open avenues for targeted protein degradation or the study of Ubi‐dependent signaling pathways [63].

Conjugation of the metathesis catalyst to **MBP‐Ubi K6C** was achieved following the report of Hilvert and Ward, using five equivalents of **[Ru(^i^Pr_2_CAAC‐5‐Br)]** in PBS buffer at pH 7.2 with 25% *tert*‐butanol as cosolvent [64]. Under these conditions, cysteine alkylation proceeded efficiently at 25°C within 5 h, affording 95% bioconjugation as determined by mBBr fluorescence assay. This approach gives well‐defined, site‐specific Ubi–catalyst conjugates. The product **Ru(^i^Pr_2_CAAC‐5) · MBP‐Ubi K6C** was characterized by HRMS to confirm the modification and single‐site attachment (see Supporting Information, Figure S78).

### 2.11 Catalysis of the Bioconjugates

Having established three distinct bioconjugation strategies, we next evaluated whether the resulting Ru(^i^Pr_2_CAAC‐5) bioconjugates retain catalytic activity for olefin metathesis. All bioconjugates were evaluated for the benchmark RCM reaction using tosyl‐diolefin **1** in MES buffer (pH 6.0) at 37°C and 0.1 mol% catalyst loading Table 1.

**TABLE 1:** RCM activity of Ru(^i^Pr_2_CAAC‐5) bioconjugates.^a^ ^.^

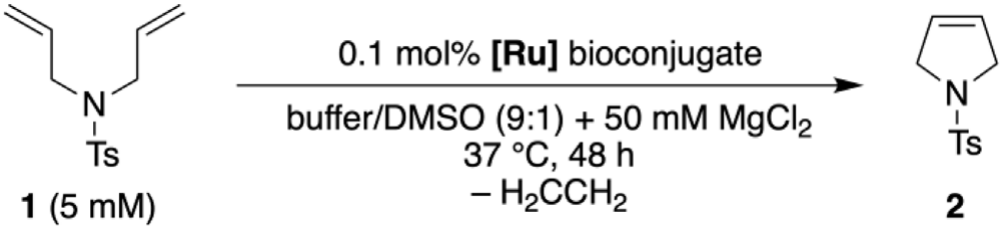			

Entry	Solvent	[Ru] bioconjugate	TON
1	MES (pH 6.0)/DMSO (9:1)	**[HG‐II]**	128 ± 17
2	MES (pH 6.0)/DMSO (9:1)	**[UltraNitroCat]**	370 ± 46
3	MES (pH 6.0)/DMSO (9:1)	**[Ru(^i^Pr_2_CAAC‐5‐Boc)]**	470 ± 25
4	MES (pH 6.0)/DMSO (9:1)	**[Ru(^i^Pr_2_CAAC‐5‐SO_2_NH_2_)]**	384 ± 30
5	MES (pH 6.0)/DMSO (9:1)	**[Ru(^i^Pr_2_CAAC‐5‐SO_2_NH_2_)] · hCA II**	197 ± 14^[b]^ (128 ± 15)^[c]^
6	MES (pH 6.0)/DMSO (9:1)	**[Ru(^i^Pr_2_CAAC‐5)] · SS01**	41 ± 2
7	MES (pH 6.0)/DMSO (9:1)	**[Ru(^i^Pr_2_CAAC‐5)] · MBP‐Ubi K6C**	174 ± 12

^a^ All reactions were carried out in 2‐(*N*‐morpholino)ethanesulfonic acid (MES) buffer + MgCl_2_ (50 mM) at 37°C for 48 h in triplicate. TON was determined by UHPLC using 2‐phenylethanol as an internal standard at 254 nm. Values are reported as mean ± standard deviation (SD) of n = 3 independent experiments; no statistical hypothesis testing was performed.

^b^ 5 µM of **[Ru(^i^Pr_2_CAAC‐5‐SO_2_NH_2_)]** was incubated with 5 µM hCA II (63% bound cofactor).

^c^ Value in parentheses: 5 µM of **[Ru(^i^Pr_2_CAAC‐5‐SO_2_NH_2_)]** was incubated with 25 µM hCA II (>95% bound cofactor).

i) Gratifyingly, all three bioconjugates (Table 1, entries 5–6) remain catalytically active, confirming that neither supramolecular anchoring, click conjugation, nor covalent protein attachment compromises the intrinsic activity of the **[Ru(^i^Pr_2_CAAC‐5)]** core. ii) The ArM **[Ru(^i^Pr_2_CAAC‐5‐SO_2_NH_2_)] · hCA II** achieves a TON = 197 (free cofactor **[Ru(^i^Pr_2_CAAC‐5‐SO_2_NH_2_)]** TON = 384), representing a substantial improvement over previously reported hCA II‐based artificial metalloenzymes for olefin metathesis (TON = 13–45 at pH 6.0) [56]. iii) The [**Ru(^i^Pr_2_CAAC‐5)] · SS01** conjugate affords a TON of approximately 41, which is particularly noteworthy given that the conjugate was subjected to reverse‐phase HPLC purification using aqueous eluents prior to catalytic testing. The persistent activity under these conditions underlines the exceptional aqueous robustness of the CAAC‐5 scaffold. iv) Finally, the covalently attached [**Ru(^i^Pr_2_CAAC‐5)] · MBP‐Ubi K6C** conjugate achieves a TON = 174. This represents a marked improvement over previously reported HG‐II‐based protein conjugates (TON = 36 at pH 5.5 and TON = 12 at pH 3.9) [26, 64]. Taken together, these results demonstrate that the modular **[Ru(^i^Pr_2_CAAC‐5)]** platform is fully compatible with diverse biomolecular environments and bioconjugation strategies. Importantly, it provides a reliable method for incorporating metathesis catalysts into biological systems.

## 3 Conclusion

We have developed a **[Ru(^i^Pr_2_CAAC‐5)]** catalyst that combines the pronounced aqueous stability of the CAAC‐5 ligand with a versatile bioconjugation handle. The ^i^Pr‐substituted complex achieves TONs of up to 600 under biologically relevant conditions—far exceeding the **HG‐II** catalyst—and retains catalytic activity upon conjugation to a protein, a peptide, and supramolecular anchoring within an artificial metalloenzyme. The modular nature of the functionalization strategy, enabled by simple late‐stage deprotection, provides a straightforward method for incorporating olefin metathesis catalysts into diverse biomolecular environments. We anticipate that this platform will serve as a foundation for the development of next‐generation bioorthogonal catalysts and artificial metalloenzymes with improved performance in complex biological settings.

## Author Contributions

**Damian Alexander Graf**: conceptualization, writing – original draft, writing – review and editing, investigation, methodology, formal analysis, visualization, validation. **Mikhail Boym**: investigation, methodology. **María Álvarez**: methodology. **Tsvetan Kardashliev**: methodology. **Thomas R. Ward**: writing – original draft, conceptualization, funding acquisition, writing – review and editing, supervision, project administration, resources.

## Conflicts of Interest

The authors declare no conflicts of Interest

## Supporting information



The authors have cited additional references within the Supporting Information [65–81].**Supporting File**: anie74875‐sup‐0001‐SuppMat.pdf.

## Data Availability

The data that supports the findings of this study are available in the Supporting Information of this article
