# Supplementary material for: A Ruthenium(CAAC‐5) Olefin Metathesis Catalyst for Bioconjugation
Source: Angew Chem Int Ed Engl. 2026 Sep 21;65(45):e3685338. doi: 10.1002/anie.3685338 (PMC13725572; doi:10.1002/anie.3685338)

Supporting Information

A Ruthenium(CAAC-5) Olefin Metathesis Catalyst for Bioconjugation

Damian Alexander Graf,[1,2,3] Mikhail Boym,[1] María Álvarez,[1] Tsvetan Kardashliev,[1] and Thomas R. Ward[1,2,3]*

[1] Department of Chemistry, University of Basel, BPR 1096, Mattenstrasse 22, CH-4002 Basel, Switzerland.
[2] Swiss Nanoscience Institute, University of Basel, Klingelbergstrasse 82, CH-4056 Basel, Switzerland.
[3] National Centre of Competence in Research Molecular Systems Engineering (NCCR MSE), University of Basel, BPR 1095, Mattenstrasse 24a, CH-4002 Basel, Switzerland.

*To whom correspondence should be addressed. thomas.ward@unibas.ch

General ... 3
Instrumentation ... 3
Synthesis Procedures ... 6
- **Ru(CAAC-5) complexes synthesis** ... 6
- **Ru($^{i}Pr_2$CAAC-5) amine S17 functionalization** ... 14

Bioconjugation via supramolecular anchoring ... 18
- **Assembly of [Ru($^{i}Pr_2$CAAC-5-SO_2NH_2)] · hCA II** ... 18
- **Binding affinity of [Ru($^{i}Pr_2$CAAC-5-SO_2NH_2)] (S19) for hCA II^{wt}** ... 18

Bioconjugation via CuAAC ... 19
- **Solid-Phase Peptide Synthesis of SS01 (S24)** ... 19
- **Synthesis of SS01 alkyne S25** ... 21

Bioconjugation via cysteine alkylation ... 24
- **Protein Expression & Purification** ... 24
- **Synthesis of Ru($^{i}Pr_2$CAAC-5) · MBP-Ubi K6C** ... 25

Substrate Synthesis ... 26
Catalytic experiments in aqueous solution ... 27
- **General procedure** ... 27
- **Temperature dependency** ... 27
- **Time-course monitoring** ... 27
- **Procedure for catalysis with Ru($^{i}Pr_2$CAAC-5) sulfonamide · hCA II** ... 27
- **Calibration curves** ... 28

Catalytic experiments in organic solution ... 28
- **Procedure for catalysis in PhMe-d_8 at different temperatures** ... 28
- **Time-course monitoring in DCM-d_2 or PhMe-d_8** ... 29

Solubility assessment of [Ru(CAAC-5-Boc)] complexes ... 31
- **Beer–Lambert linearity** ... 31
- **Solubility of [Ru(CAAC-5-Boc)] complexes over time** ... 32

Stability of [Ru(CAAC-5-Boc)] complexes ... 33
- **Precatalyst stability** ... 33
- **Catalyst stability in the presence of substrate** ... 34
- **HRMS-ESI^{+} analysis of decomposition samples** ... 35

References ... 36
NMR Spectra ... 38
HRMS Spectra ... 70

General

All chemical reactions were carried out under an inert atmosphere using standard Schlenk-line operating techniques, oven-dried glassware, and degassed anhydrous solvents unless otherwise stated. Commercially available chemicals were purchased from Sigma-Aldrich, Acros Organics, Fluka, Fluorochem, or Merck and used without further purification. Deuterated solvents were purchased from Cambridge Isotope Laboratories Inc. The water was purified using a Milli-Q system (Millipore). Thin-layer chromatography (TLC) was performed on Merck TLC Silica gel 60 F254 plates, using a UV-detector (254 nm or 360 nm). Basic $KMnO_4$ solution was used for staining. Column chromatography was performed using Merck Silica gel 60 (0.040-0.063 mm). Unless otherwise stated, the catalytic experiments were performed in non-degassed HPLC-grade solvents in 1.5 mL GC glass vials.

Instrumentation

NMR spectra were recorded on a 500 MHz or 600 MHz Bruker Avance spectrometer at 25 °C or 37 °C and evaluated with MestReNova. ^{1}H NMR and ^{13}C NMR chemical shifts (δ) are reported in parts per million (ppm) relative to the residual solvent resonance of the deuterated solvent indicated. Coupling constants (J) are reported in Hertz (Hz). Multiplicities are abbreviated as follows: s = singlet, d = doublet, t = triplet, q = quartet, m = multiplet, br = broad, dd = doublet of doublets, dt = doublet of triplets, dq = doublet of quartets, td = triplet of doublets, ddd = doublet of doublet of doublets, ddt = doublet of doublet of triplets, dtd = doublet of triplet of doublets, and dddd = doublet of doublet of doublet of doublets. Data are reported in the order: chemical shift, multiplicity, coupling constant(s), and integration.

Routine MS analysis was performed on a Shimadzu LC-MS 2020 equipped with an Agilent ZORBAX RR SB-C18 column (4.6 x 75 mm, 3.5 μm) and PDA through direct injection or column injection. Solvents for LC-MS measurements were (A) Milli-Q water + 3% HPLC-grade acetonitrile + 0.1% formic acid, (B) HPLC-grade acetonitrile + 5% Milli-Q water + 0.1% formic acid, (C) HPLC-grade methanol + 3 mM ammonium bicarbonate, and (D) HPLC-grade 2-propanol. Method 1: The flow rate was set to 1 mL/min, and the column temperature was maintained at 25 °C. The method used includes the following parameters: t_{total} = 15.00 min with 0.00 min – 100% (A); 1.00 min – 100% (A); 9.00 min – 5% (A); 11.00 min – 5% (A); 15.00 min – 100% (A); end. Mass detection was performed in scan mode from 100 to 800 m/z for positive and negative ions (cone voltage 40 V). Method 2: The flow rate was set to 1 mL/min, and the column temperature was maintained at 25 °C. The method used includes the following parameters: t_{total} = 15.00 min with 0.00 min – 100% (A); 1.00 min – 100% (A); 5.00 min – 5% (A); 11.00 min – 5% (A); 15.00 min – 100% (A); end. Mass detection was performed in scan mode from 100 to 2000 m/z for positive and negative ions (cone voltage 40 V).

High-resolution mass spectrometry (HRMS) was performed by the analytical facility of the chemistry department of the University of Basel on a Bruker maXis4G QTOF ESI mass spectrometer coupled to a

Shimadzu LC. Before HRMS submission, all samples were characterized by direct injection or by liquid injection of solutions prepared in HPLC-grade MeOH or MeCN using a Shimadzu LCMS-2020. Protein samples were prepared in PBS (50 mM, pH 7.2) or MES (50 mM, pH 6.0) buffer.

UHPLC analysis was performed on an Agilent 1290 Infinity II system equipped with an InfinityLab Poroshell 120 EC-C18 column (2.1 x 50 mm, 1.9 µm) and PDA set to 254 nm. Solvents for UHPLC measurements were (A) Milli-Q water + 3% HPLC-grade acetonitrile + 0.1% trifluoroacetic acid and (B) HPLC-grade acetonitrile + 5% Milli-Q water + 0.1% trifluoroacetic acid. The flow rate was set to 0.50 mL/min, and the column temperature was maintained at 25 °C. The method used includes the following parameters: t_{total} = 9.00 min with 0.00 min – 100% (A); 0.50 min – 100% (A); 6.00 – 10% (A); 7.00 min – 10% (A); 8.50 min – 100% (A); 9.00 min – 100% (A); end.

Preparative RP-HPLC purification was performed using a Waters 2545 Binary Gradient Pump equipped with an Agilent ZORBAX Eclipse XDB-C18 column (21.2 x 150 mm, 5.0 µm), a Waters 2489 UV/Vis Detector set to 254 nm (ChA) and 380 nm (ChB), and a Waters Fraction Collector III. Solvents for preparative RP-HPLC were (A) Milli-Q water + 3% HPLC-grade acetonitrile + 0.1% trifluoroacetic acid and (B) HPLC-grade acetonitrile + 5% Milli-Q water + 0.1% trifluoroacetic acid. The flow rate was set to 20.0 mL/min, and the column temperature was maintained at 25 °C. The method used includes the following parameters: t_{total} = 28 min with 0.00 min – 100% (A); 2.00 min – 100% (A); 20.00 min – 2% (A); 23.00 min – 2% (A); 25.00 min – 95% (A); 28.00 min – 95% (A); end.

Binding affinity was determined using fluorescence assays recorded on a TECAN M1000 Pro plate reader. Samples were prepared in Nunclon black flat-bottom 96-well plates. Measurement settings for fluorescence top reading: The excitation wavelength was set to 280 nm (bandwidth 5 nm), and the fluorescence emission was recorded at 470 nm (bandwidth 5 nm) with a gain of 100 and a flash frequency of 400 Hz at 25 °C.

Bioconjugation was determined by mBBr fluorescence assay on a BMG plate reader. Samples were prepared in Greiner black flat-bottom 96-well plates. Measurement settings for fluorescence bottom reading: The excitation wavelength was set to 390 nm (bandwidth 15 nm), and the fluorescence emission was recorded at 480 nm (bandwidth 20 nm) with a gain of 1400 and a flash frequency of 20 Hz at 25 °C.

Protein concentration was determined by Bradford assay on a BMG plate reader. Samples were prepared in Greiner flat-bottom 96-well plates. Measurement settings for absorbance top reading: The absorbance wavelength was set to 595 nm at 25 °C.

UV-Vis studies were performed on an Agilent Cary 60 UV-Vis spectrometer connected to a PCB 1500 Water Peltier System. Beer-Lambert linearity was performed using scan mode between 800.0 and 200.0 nm at

4’200 nm/min. Precipitation studies were performed using the scanning kinetics mode between 500.0 and 300.0 nm with an average time of 0.1000 s, an interval of 1.00 nm, and a scan rate of 600.0 nm/min. The measurements were performed at 37 °C, with a cycle time of 5.0 min for 480.0 min.

Synthesis Procedures

Ru(CAAC-5) complexes synthesis

Synthesis of *tert*-butyl *p*-tolylcarbamate (S1)

Prepared according to the literature.[65]

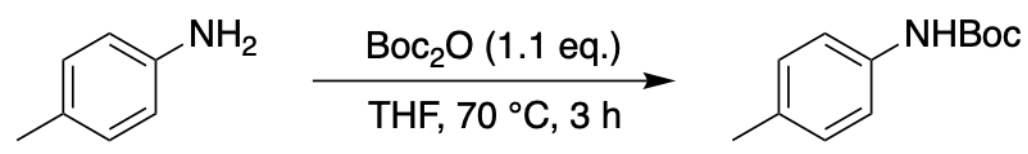

S1 (86%)

A solution of *p*-toluidine (10.7 g, 100 mmol, 1.0 eq.) and di-*tert*-butyl dicarbonate (24.0 g, 110 mmol, 1.1 eq.) in THF (100 mL) was refluxed at 70 °C for 3 hours. The solvent was removed *in vacuo*, and the resulting oil was left in the freezer for 30 min. The resulting crystals were recrystallized from hexane to afford *tert*-butyl *p*-tolylcarbamate (**S1**, 17.9 g, 86%) as a white crystalline solid.

^{1}H NMR (500 MHz, $CDCl_3$) δ 7.23 (d, *J* = 7.9 Hz, 2H), 7.09 (d, *J* = 7.9 Hz, 2H), 6.40 (br, 1H), 2.29 (s, 3H), 1.51 (s, 9H). The ^{1}H NMR data were in agreement with the reported values.

Synthesis of *tert*-butyl methyl(*p*-tolyl)carbamate (S2)

Prepared according to the literature.[66]

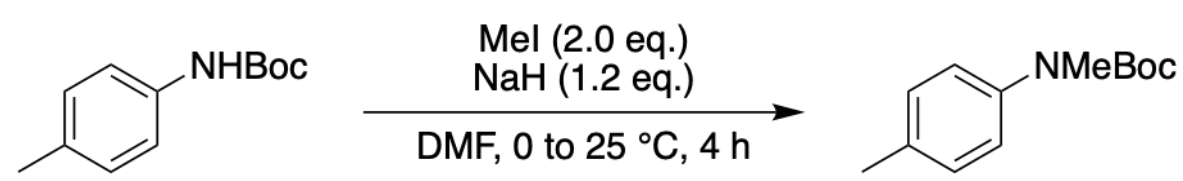

S2 (93%)

To a suspension of NaH (60% in mineral oil, 1.2 g, 30.0 mmol, 1.2 eq.) in DMF (20 mL), *tert*-butyl methyl(*p*-tolyl)carbamate (**S1**, 5.28 g, 25.0 mmol, 1.0 eq.) was added dropwise at 0 °C. The reaction mixture was stirred at 0 °C for 1 hour. Then, methyl iodide (7.10 g, 3.11 mL, 50.0 mmol, 2.0 eq.) was added in one portion, and the suspension was stirred at 25 °C for 3 hours. The reaction mixture was poured into water (200 mL) and extracted with EA (3 x 100 mL), washed with water (3 x 100 mL), brine (100 mL), dried over Na_2SO_4, and the solvent was removed *in vacuo* to afford *tert*-butyl methyl(*p*-tolyl)carbamate (**S2**, 5.13 g, 93%). The product was used without any further purification.

^{1}H NMR (500 MHz, $CDCl_3$) δ 7.19–6.79 (m, 4H), 3.23 (s, 3H), 2.33 (s, 3H), 1.44 (s, 9H). The ^{1}H NMR data were in agreement with the reported values.

Synthesis of *tert*-butyl (4-(bromomethyl)phenyl)(methyl)carbamate (S3)

Prepared according to the literature.[67]

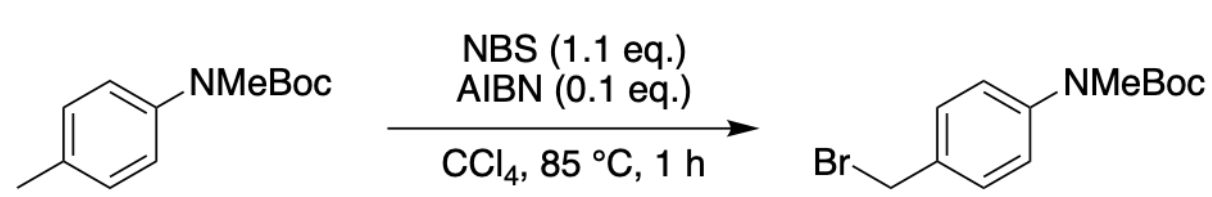

S3 (63%)

A mixture of *tert*-butyl methyl(p-tolyl)carbamate (**S2**, 4.87 g, 22.0 mmol, 1.0 eq.), NBS (4.31 g, 24.2 mmol, 1.1 eq.), and AIBN (361 mg, 2.20 mmol, 0.1 eq.) in CCl_4 (60 mL) was heated to 85 °C for 1 hour (reaction initiates at around 75 °C). The reaction solution was filtered, and the solvent was removed *in vacuo*. The

crude product was purified by column chromatography (SiO_2, hex/EA, 19:1 to 7:3) to afford *tert*-butyl (4-(bromomethyl)phenyl)(methyl)carbamate (**S3**, 4.19 g, 63%) as a yellow oil.
^{1}H NMR (500 MHz, $CDCl_3$) δ 7.34 (d, *J* = 8.1 Hz, 2H), 7.21 (d, *J* = 8.1 Hz, 2H), 4.48 (s, 2H), 3.25 (s, 3H), 1.46 (s, 9H). The ^{1}H NMR data were in agreement with the reported values.

Synthesis of 2-methylallyl propionate (S4)

Prepared according to the literature.[68]

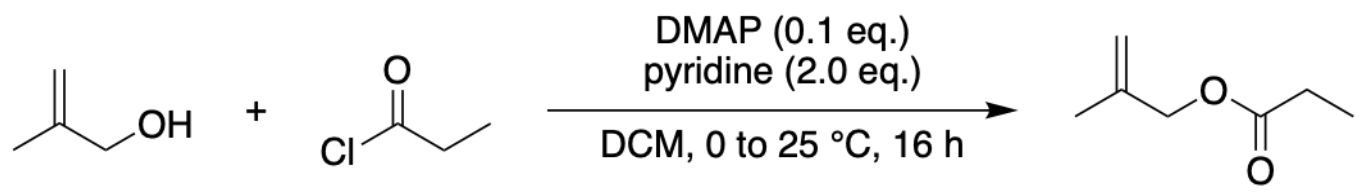

S4 (99%)

To an ice-cooled solution of 2-methyl-2-propen-1-ol (7.21 g, 100 mmol, 1.0 eq.), DMAP (1.22 g, 10.0 mmol, 0.1 eq.), and pyridine (15.8 g, 16.1 mL, 200 mmol, 2.0 eq.) in DCM (200 mL), propionyl chloride (11.1 g, 10.5 mL, 120 mmol, 1.2 eq.) was added dropwise. After the addition was complete, the reaction mixture was stirred at 25 °C for 16 hours. The reaction mixture was quenched with saturated aqueous $NaHCO_3$ (200 mL), extracted with DCM (3 x 100 mL), washed with aqueous 1 M HCl (100 mL) and brine (100 mL), dried over $MgSO_4$, and the solvent was removed *in vacuo* to afford 2-methylallyl propionate (**S4**, 12.6 g, 99%) as a clear oil. The product was used without any further purification.
^{1}H NMR (500 MHz, $CDCl_3$) δ 4.97 (dt, *J* = 1.7, 0.8 Hz, 1H), 4.92 (td, *J* = 1.7, 0.8 Hz, 1H), 4.50 (s, 2H), 2.37 (q, *J* = 7.6 Hz, 2H), 1.98–1.55 (m, 3H), 1.16 (t, *J* = 7.6 Hz, 3H). The ^{1}H NMR data were in agreement with the reported values.

Synthesis of 2,4-dimethylpent-4-en-1-ol (S5)

Prepared according to the literature.[68]

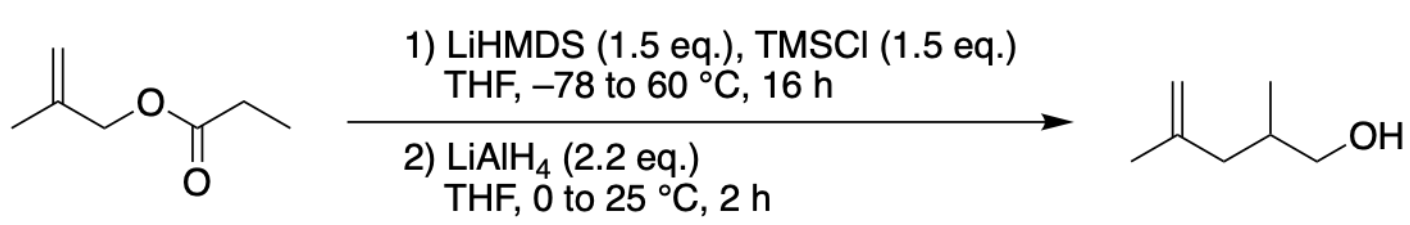

S5 (87%)

To a solution of 2-methylallyl propionate (**S4**, 4.00 g, 31.2 mmol, 1.0 eq.) and TMSCl (5.08 g, 46.8 mmol, 1.5 eq.) in THF (50 mL), a solution of 1 M LiHMDS in THF (46.8 mL, 46.8 mmol, 1.5 eq.) was added at –78 °C. After the addition was complete, the reaction mixture was warmed to 25 °C and stirred for 1 hour. The reaction mixture was heated to 60 °C in an oil bath for 16 hours, then allowed to cool to 25 °C before being quenched with aqueous 1 M HCl (80 mL). The reaction mixture was extracted with DCM (3 x 100 mL), washed with brine (200 mL), dried over $MgSO_4$, and the solvent was removed *in vacuo* to afford the unpurified carboxylic acid as a yellow oil, which was used in the next step without further purification. To a solution of the unpurified carboxylic acid dissolved in THF (50 mL), $LiAlH_4$ (2.61 g, 68.6 mmol, 2.2 eq.) was added in four portions at 0 °C. Upon completion of the addition, the reaction mixture was stirred at 25 °C for 2 hours. The reaction was quenched with EA (60 mL), and aqueous 1 M NaOH (60 mL) was added dropwise to the reaction mixture. The resulting mixture was then extracted with Et_2O (3 x 100 mL). The

combined organic layers were washed with brine (200 mL), dried over $MgSO_4$, and the solvent was removed *in vacuo*. The crude was purified by column chromatography (SiO_2, Et_2O/hex, 1:2) to afford 2,4-dimethylpent-4-en-1-ol (**S5**, 3.11 g, 87%) as a colorless oil.

^{1}H NMR (500 MHz, $CDCl_3$) δ 4.76 (dq, *J* = 2.2, 1.0 Hz, 1H), 4.71 (dq, *J* = 2.2, 1.0 Hz, 1H), 3.51 (dt, *J* = 11.0, 5.5 Hz, 1H), 3.45 (dt, *J* = 10.6, 6.0 Hz, 1H), 2.23–2.07 (m, 1H), 1.93–1.80 (m, 2H), 1.73 (t, *J* = 1.2 Hz, 3H), 1.43 (td, *J* = 6.0, 1.1 Hz, 1H), 0.90 (d, *J* = 6.5 Hz, 3H), 0.88–0.76 (m, 1H). The ^{1}H NMR data were in agreement with the reported values.

Synthesis of 2,4-dimethylpent-4-enal (S6)

Prepared according to the literature.[68]

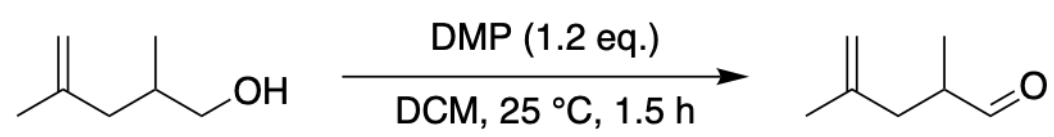

S6 (quant.)

To a solution of 2,4-dimethylpent-4-en-1-ol (**S5**, 4.34 g, 38.0 mmol, 1.0 eq.) in DCM (200 mL), Dess–Martin periodinane (19.3 g, 45.6 mmol, 1.2 eq.) was added in one portion, and the reaction mixture was stirred at 25 °C for 3 hours. Upon completion of the reaction, saturated aqueous $NaHCO_3$ (130 mL) and saturated aqueous NaS_2O_3 (130 mL) were added, and the reaction mixture was stirred at 25 °C for 1 hour. The reaction mixture was extracted with DCM (3 x 100 mL), washed with saturated aqueous $NaHCO_3$ (200 mL) and brine (200 mL), dried over Na_2SO_4, and concentrated *in vacuo* to afford 2,4-dimethylpent-4-enal (**S6**, 4.26 g, quant.) as a yellowish liquid. The product was used in the next step without any additional purification.

^{1}H NMR (500 MHz, CD_2Cl_2) δ 9.61 (d, *J* = 1.9 Hz, 1H), 4.81 (m, 1H), 4.72 (m, 1H), 2.52 (dddd, *J* = 13.6, 8.7, 6.8, 1.9 Hz, 1H), 2.45 (ddd, *J* = 14.3, 6.6, 1.1 Hz, 1H), 2.02 (ddd, *J* = 14.2, 8.0, 1.1 Hz, 1H), 1.72 (t, *J* = 1.2 Hz, 3H), 1.05 (d, *J* = 6.9 Hz, 3H). The ^{1}H NMR data were in agreement with the reported values.

Synthesis of alkenyl aldimine S7

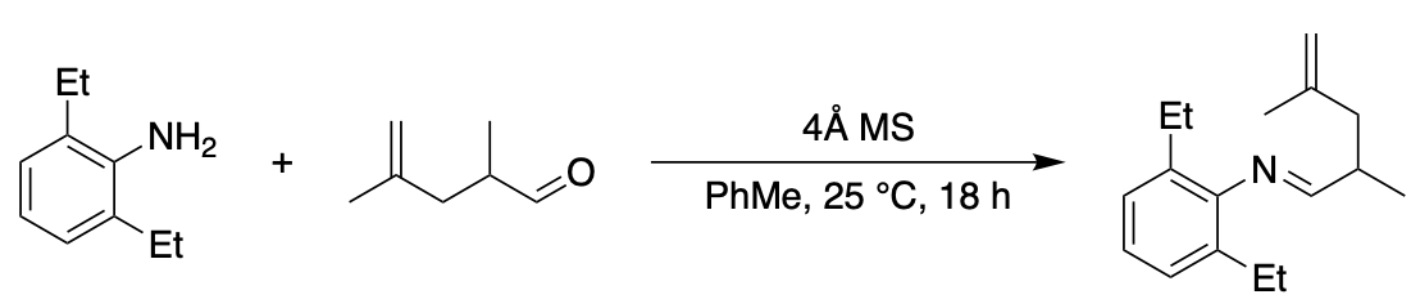

S7 (99%)

To a mixture of 4Å MS (6.0 g) in PhMe (40 mL), 2,6-diethylaniline (1.64 g, 1.81 mL, 11.0 mmol, 1.0 eq.) and 2,4-dimethylpent-4-enal (**S6**, 3.70 g, 33.0 mmol, 3.0 eq.) were added. The reaction mixture was stirred at 25 °C for 18 hours. Upon completion, the reaction mixture was filtered through basic Alox, and the solvent was removed *in vacuo* to afford the alkenyl aldimine **S7** (2.68 g, 99%) as a yellow oil. The product was used in the next step without any additional purification.

^{1}H NMR (500 MHz, $CDCl_3$) δ 7.50 (d, *J* = 5.5 Hz, 1H), 7.02 (d, *J* = 7.4 Hz, 2H), 6.98 (m, 1H), 4.82 (m, 1H), 4.79 (m, 1H), 2.90–2.80 (m, 1H), 2.44 (q, *J* = 7.6 Hz, 4H), 2.17 (dd, *J* = 14.2, 7.3 Hz, 1H), 1.80 (s, 3H), 1.21 (d, *J* = 6.9 Hz, 3H), 1.11 (t, *J* = 7.6 Hz, 6H).

Synthesis of enamine S8

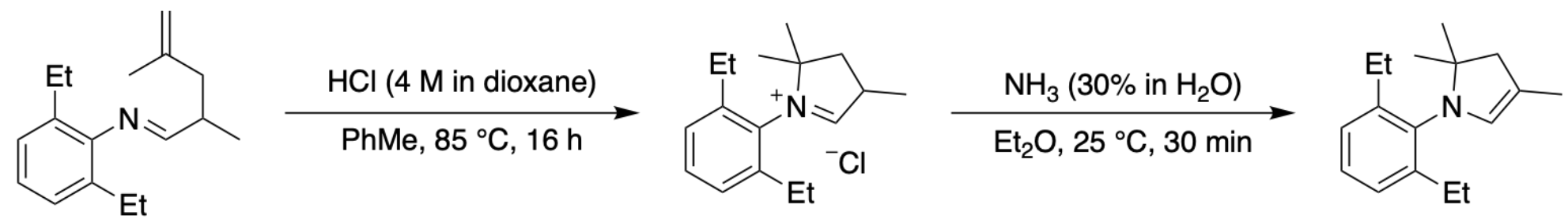

In a pressure tube, to a solution of alkenyl aldimine **S7** (2.68 g, 11 mmol, 1.0 eq.) in PhMe (22 mL), a solution of 4 M hydrogen chloride in dioxane (6.87 mL, 27.5 mmol, 2.5 eq.) was added at 0 °C. The reaction mixture was stirred at 85 °C for 16 hours, cooled to 25 °C, and the solvent was removed *in vacuo*. The crude product was precipitated from DCM/Et_2O (sometimes with the addition of hexane) and washed several times with Et_2O to afford a mixture of Et_2CAAC-5-H chloride salt and 2,6-diethyl-anilinium chloride (1.61 g). To a suspension of this mixture (1.61 g, 5.75 mmol, 1.0 eq.) dissolved in Et_2O (20 mL), a solution of 30% NH_4OH in H_2O (17.3 mL, 144 mmol, 25 eq.) was added. The reaction mixture was stirred at 25 °C for 30 minutes and extracted with Et_2O, washed with brine (50 mL), dried over $MgSO_4$, and concentrated *in vacuo*. The crude product was purified by column chromatography (SiO_2, pent/EA, 50:1) to afford enamine **S8** (432 mg, 31%) as a yellow oil.

^{1}H NMR (500 MHz, $CDCl_3$) δ 7.13 (dd, *J* = 8.6, 6.2 Hz, 1H), 7.08 (d, *J* = 8.2 Hz, 2H), 5.56 (h, *J* = 1.6 Hz, 1H), 2.88 (dq, *J* = 15.0, 7.5 Hz, 2H), 2.57 (dq, *J* = 14.9, 7.6 Hz, 2H), 2.47 (p, *J* = 1.4 Hz, 2H), 1.72 (q, *J* = 1.4 Hz, 3H), 1.16 (t, *J* = 7.6 Hz, 6H), 1.14 (s, 6H).

^{13}C NMR (126 MHz, $CDCl_3$) δ 146.46, 140.13, 133.88, 126.66, 126.30, 105.25, 65.42, 51.30, 28.41, 25.02, 16.04, 13.99.

HRMS-ESI$^+$: (m/z) calcd. for $C_{17}H_{26}N^+$ 244.2060, found 244.2052.

Synthesis of Et_2CAAC-5-H BF_4 S9

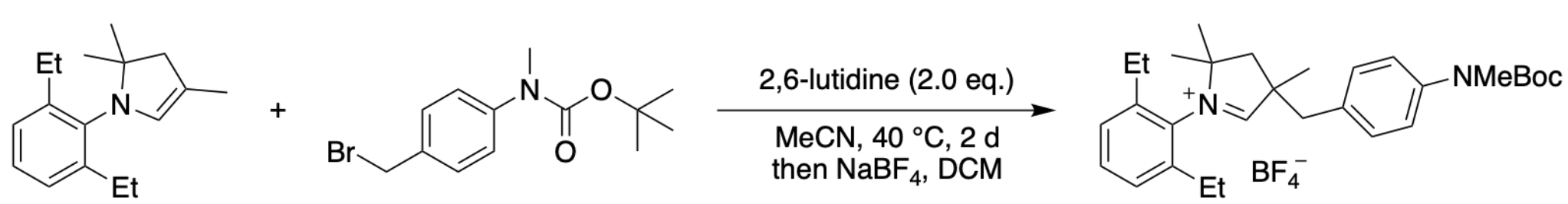

To a solution of enamine **S8** (212 mg, 0.87 mmol, 1.0 eq.) in MeCN (10 mL), 2,6-lutidine (186 mg, 203 µL, 1.74 mmol, 2.0 eq.), followed by *tert*-butyl (4-(bromomethyl)phenyl)(methyl) carbamate (**S3**, 261 mg, 0.87 mmol, 1.0 eq.) were added. The reaction mixture was stirred at 25 °C for 14 hours. The solvent was removed *in vacuo*, and the crude was precipitated from DCM/hex and dried *in vacuo*. The crude was dissolved in DCM (5 mL), and sodium tetrafluoroborate (287 mg, 2.61 mmol, 3.0 eq.) was added. The reaction was stirred at 25 °C for 14 hours. The crude was diluted with DCM (10 mL), washed with water (10 mL) and brine (10 mL), dried over $MgSO_4$, and the solvent was removed *in vacuo* to afford the **Et_2CAAC-5-H BF_4 S9** (211 mg, 44%) as an orange solid. The product was used without any additional purification.

^{1}H NMR (500 MHz, $CDCl_3$) δ 9.39 (s, 1H), 7.44 (t, *J* = 7.7 Hz, 1H), 7.36–7.32 (m, 2H), 7.28 (d, *J* = 7.8 Hz, 2H), 7.23 (dd, *J* = 7.9, 1.4 Hz, 1H), 3.74 (d, *J* = 14.0 Hz, 1H), 3.26 (s, 3H), 2.90 (d, *J* = 14.0 Hz, 1H), 2.69

(d, J = 13.7 Hz, 1H), 2.50 (q, J = 7.5 Hz, 2H), 2.23 (d, J = 13.7 Hz, 1H), 1.92–1.83 (m, 2H), 1.86 (s, 3H), 1.46 (s, 9H), 1.45 (s, 3H), 1.30 (t, J = 7.5 Hz, 3H), 1.11 (t, J = 7.5 Hz, 3H), 1.08 (s, 3H).

^{13}C NMR (126 MHz, $CDCl_3$) δ 192.22, 154.60, 143.55, 139.81, 139.45, 131.40, 130.78, 130.69, 128.59, 127.79, 127.73, 126.02, 83.43, 80.66, 54.36, 44.52, 43.35, 37.41, 28.47, 28.35, 28.04, 27.11, 24.83, 24.69, 15.22, 15.10.

HRMS-ESI$^+$: (m/z) calcd. for $C_{30}H_{43}N_2O_2^+$ 463.3319, found 463.3326.

Synthesis of [Ru(Et₂CAAC-5-Boc)] S10

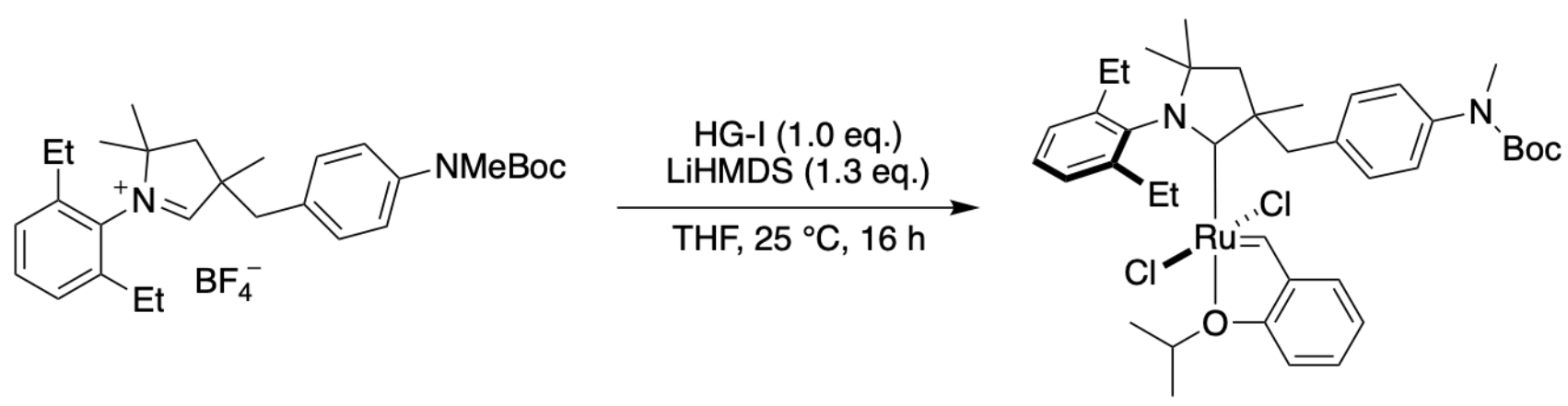

S10 (41%)

To **Et_2CAAC-5-H BF_4 S9** (7.16 mg, 13 µmol, 1.3 eq.) and **HG-I** (6.01 mg, 10.0 µmol, 1.0 eq.), a 25 mM solution of LiHMDS in THF (520 µL, 13.0 µmol, 1.3 eq.) was added. The reaction mixture was stirred at 25 °C for 16 hours. Then, the solvent was evaporated, and the crude product was purified by column chromatography (SiO_2, PE/EA, 9:1 to 4:1) to afford **[Ru(Et_2CAAC-5-Boc)] S10** (3.20 mg, 41%) as a green solid.

^{1}H NMR (500 MHz, CD_2Cl_2) δ 16.33 (s, 1H), 7.64–7.58 (m, 2H), 7.48 (d, J = 7.8 Hz, 2H), 7.37 (d, J = 8.4 Hz, 2H), 7.25 (d, J = 8.4 Hz, 2H), 7.00 (d, J = 8.3 Hz, 1H), 6.96–6.86 (m, 2H), 5.17 (h, J = 6.0 Hz, 1H), 4.37 (d, J = 12.8 Hz, 1H), 3.80 (d, J = 12.8 Hz, 1H), 3.27 (s, 3H), 2.64 (m, 2H), 2.57 (m, 2H), 2.50 (d, J = 12.6 Hz, 1H), 1.91 (d, J = 12.6 Hz, 1H), 1.89 (s, 3H), 1.76 (dd, J = 6.1, 3.5 Hz, 6H), 1.46 (s, 9H), 1.36 (s, 3H), 1.31 (s, 3H), 0.95 (t, J = 7.4 Hz, 3H), 0.89 (t, J = 7.4 Hz, 3H).

^{13}C NMR (126 MHz, CD_2Cl_2) δ 297.02, 265.95, 154.97, 152.72, 144.21, 143.79, 143.76, 142.97, 138.91, 135.58, 131.22, 131.18, 129.42, 127.38, 127.30, 125.46, 123.73, 122.43, 113.51, 80.32, 78.99, 75.49, 61.17, 48.92, 46.77, 37.57, 29.73, 28.46, 28.09, 25.72, 25.60, 24.82, 22.28, 22.23, 14.87, 14.56.

HRMS-ESI$^+$: (m/z) calcd. for $C_{40}H_{54}ClN_2O_3Ru^+$ 747.2861, found 747.2878.

Synthesis of imine S11

Prepared according to the literature.[69]

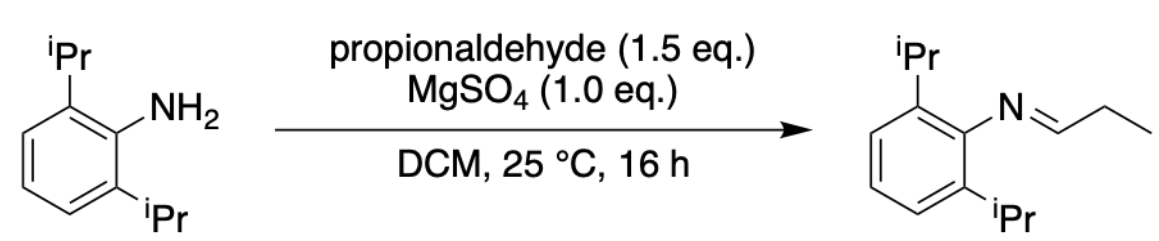

S11 (quant.)

To a mixture of $MgSO_4$ (10.8 g, 90.0 mmol, 1.0 eq.) and 2,6-diisopropylaniline (16.0 g, 17.0 mL, 90.0 mmol, 1.0 eq.) in DCM (100 mL), propionaldehyde (7.84 g, 9.74 mL, 135 mmol, 1.5 eq.) was added . The reaction mixture was stirred at 25 °C for 16 hours and then filtered through a plug of Celite. The solvent and excess

propionaldehyde were removed *in vacuo* to afford the imine **S11** (19.5 g, quant.) as a pale-yellow oil. The product was used in the next step without any additional purification.

^{1}H NMR (500 MHz, $CDCl_3$) δ 7.64 (t, *J* = 4.4 Hz, 1H), 7.13–7.09 (m, 2H), 7.08–7.04 (m, 1H), 2.92 (p, *J* = 6.9 Hz, 2H), 2.54 (qd, *J* = 7.5, 4.4 Hz, 2H), 1.26 (t, *J* = 7.5 Hz, 3H), 1.16 (d, *J* = 6.9 Hz, 12H). The ^{1}H NMR data were in agreement with the reported values.

Synthesis of alkenyl aldimine S12

Prepared according to the literature.[69]

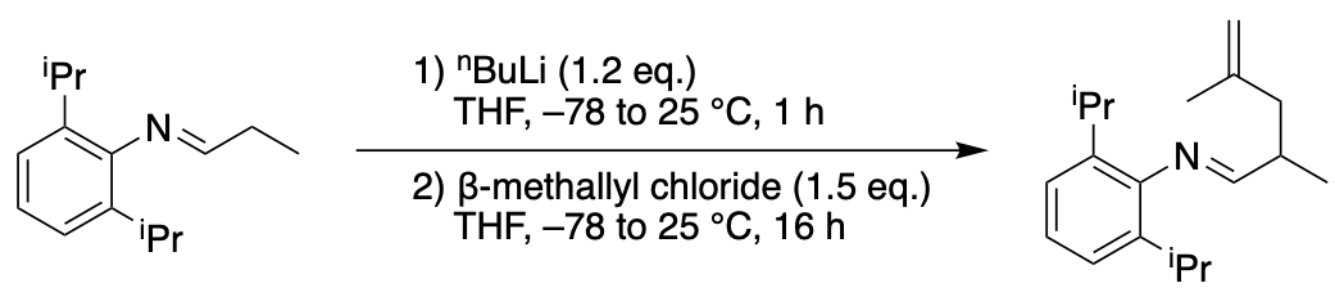

S12 (quant.)

To a solution of imine **S11** (19.5 g, 90.0 mmol, 1.0 eq.) dissolved in THF (50 mL), a solution of 2.5 M ^{n}BuLi in hexanes (43.0 mL, 108 mmol, 1.2 eq.) was added at –78 °C. The solution was warmed to 25 °C and stirred for 1 hour. The reaction mixture was cooled to –78 °C, and β-methallyl chloride (12.2 g, 13.1 mL, 135 mmol, 1.5 eq.) was added in three portions. The solution was slowly warmed to room temperature overnight. Removal of the volatiles under vacuum afforded a mixture of an oil (alkenyl aldimine **S12**) and a solid (LiCl) in quantitative yield. The product was used in the next step without any additional purification.

^{1}H NMR (500 MHz, $CDCl_3$) δ 7.48 (d, *J* = 5.5 Hz, 1H), 7.13 – 7.03 (m, 3H), 4.83–4.81 (m, 1H), 4.80–4.78 (m, 1H), 2.93 (p, *J* = 6.9 Hz, 2H), 2.90–2.83 (m, 1H), 2.41 (dd, *J* = 14.2, 7.9 Hz, 1H), 2.18 (dd, *J* = 14.2, 7.2 Hz, 1H), 1.80 (s, 3H), 1.21 (d, *J* = 6.8 Hz, 3H), 1.14 (dd, *J* = 6.9, 3.4 Hz, 12H). The ^{1}H NMR data were in agreement with the reported values.

Synthesis of ^{i}Pr$_2$CAAC-5-H BF_4 S13

Prepared according to the literature.[69]

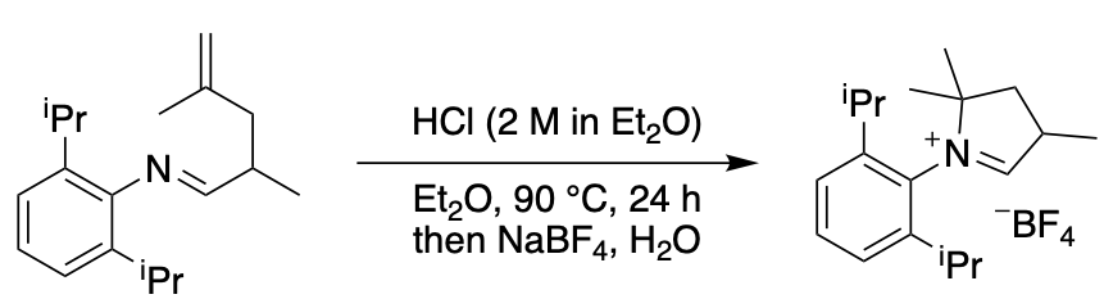

S13 (40%)

To a solution of alkenyl aldimine **S12** (24.4 g, 90.0 mmol, 1.0 eq.) in Et_2O (30 mL), a solution of 2 M HCl in Et_2O (90.0 mL, 180 mmol, 2.0 eq.) was added at –78 °C. After 15 minutes, the mixture was warmed to 25 °C and stirred for an additional 15 minutes. The solution was heated in a pressure tube at 90 °C for 24 hours and then cooled to 25 °C. The solution was filtered with a cannula, and the solid was washed with Et_2O (3 x 30 mL). The iminium salt was dried *in vacuo* to afford a colorless powder. To a Schlenk tube containing the iminium salt and $NaBF_4$ (19.8 g, 180 mmol, 2.0 eq.), H_2O (90 mL) was added. The mixture was stirred at 25 °C for 1 hour and then filtered. The solid was washed with H_2O (3 x 30 mL), Et_2O (3 x

30 mL), pentane (30 mL), and dried *in vacuo* to afford **^{i}Pr$_2$CAAC-5-H BF$_4$ S13** (12.9 g, 40%) as a white solid.

^{1}H NMR (500 MHz, $CDCl_3$) δ 8.86 (s, 1H), 7.53 (t, *J* = 7.8 Hz, 1H), 7.38 (dd, *J* = 7.9, 1.4 Hz, 1H), 7.32 (dd, *J* = 7.8, 1.4 Hz, 1H), 4.29 (h, *J* = 7.8 Hz, 1H), 2.90–2.76 (m, 2H), 2.59 (hept, *J* = 6.8 Hz, 1H), 2.07 (dd, *J* = 13.2, 8.5 Hz, 1H), 1.69–1.59 (m, 6H), 1.44 (s, 3H), 1.38 (d, *J* = 6.8 Hz, 3H), 1.32 (d, *J* = 6.7 Hz, 3H), 1.23 (d, *J* = 6.6 Hz, 3H), 1.13 (d, *J* = 6.8 Hz, 3H). The ^{1}H NMR data were in agreement with the reported values.

Synthesis of enamine S14

Prepared according to the literature.[69]

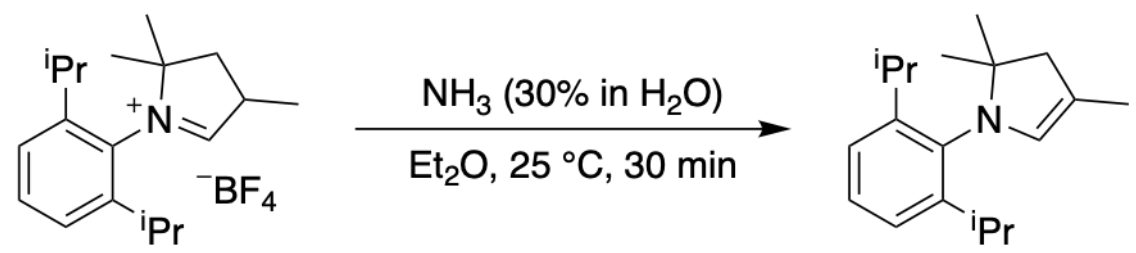

S14 (quant.)

To a suspension of **^{i}Pr$_2$CAAC-5-H BF$_4$ S13** (11.5 g, 32.0 mmol, 1.0 eq.) in Et_2O (40 mL), a solution of 30% ammonia in water (13.2 mL, 96.0 mmol, 3.0 eq.) was added. The biphasic mixture was stirred at 25 °C for 30 min. The organic phase was separated, and the aqueous phase was extracted with Et_2O (3 x 100 mL). The ether solution was combined and dried with $MgSO_4$ for 6 hours, and the solvent was removed *in vacuo* to afford the enamine **S14** (8.68 g, quant.) as a pale-yellow oil.

^{1}H NMR (500 MHz, $CDCl_3$) δ 7.20 (dd, *J* = 8.3, 6.9 Hz, 1H), 7.13–7.08 (m, 2H), 5.58 (h, *J* = 1.6 Hz, 1H), 3.51 (hept, *J* = 6.9 Hz, 2H), 2.50 (p, *J* = 1.4 Hz, 2H), 1.72 (q, *J* = 1.4 Hz, 3H), 1.23 (d, *J* = 6.9 Hz, 6H), 1.16 (s, 6H), 1.08 (d, *J* = 6.8 Hz, 6H). The ^{1}H NMR data were in agreement with the reported values.

Synthesis of ^{i}Pr$_2$CAAC-5-H BF$_4$ S15

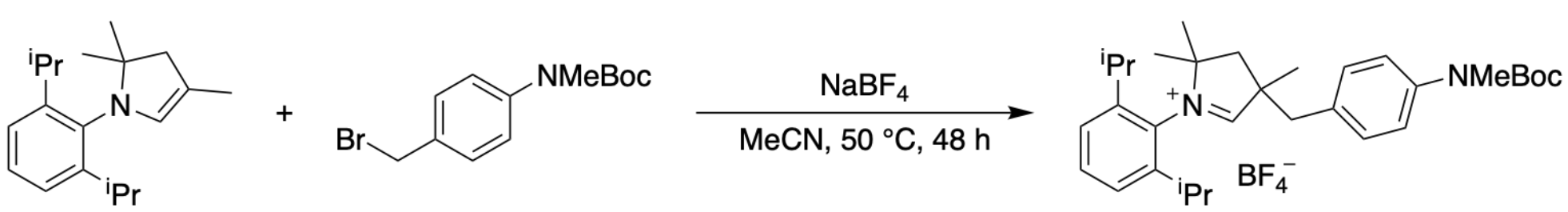

S15 (61%)

To a solution of enamine **S14** (2.36 g, 8.70 mmol, 1.0 eq.) dissolved in MeCN (30 mL), *tert*-butyl (4-(bromomethyl)phenyl)(methyl)carbamate (**S3**, 2.61 g, 8.70 mmol, 1.0 eq.) was added. The reaction mixture was heated to 50 °C for 48 hours. After cooling to room temperature, the solvent was removed *in vacuo*, and the oily residue was precipitated from DCM/Et_2O and washed with Et_2O (3 x 30 mL) to afford a yellowish solid. To the crude solid dissolved in DCM (25 mL), sodium tetrafluoroborate (2.39 g, 21.7 mmol, 2.5 eq.) was added, and the reaction was stirred at 25 °C for 1 hour. The crude was diluted with DCM (20 mL), washed with water (20 mL) and brine (20 mL), dried over $MgSO_4$, and the solvent was removed *in vacuo* to afford **^{i}Pr$_2$CAAC-5-H BF$_4$ S15** (3.05 g, 61%) as an off-white solid.

^{1}H NMR (500 MHz, $CDCl_3$) δ 9.48 (s, 1H), 7.49 (t, *J* = 7.8 Hz, 1H), 7.36 (d, *J* = 8.4 Hz, 2H), 7.29 (m, 4H), 3.81 (d, *J* = 14.1 Hz, 1H), 3.25 (s, 3H), 2.89 (d, *J* = 14.1 Hz, 1H), 2.70 (d, *J* = 13.8 Hz, 1H), 2.59 (p, *J* =

6.8 Hz, 1H), 2.24 (d, *J* = 13.8 Hz, 1H), 2.07 (p, *J* = 6.6 Hz, 1H), 1.83 (s, 3H), 1.44 (s, 9H), 1.42 (s, 3H), 1.33 (d, *J* = 6.7 Hz, 3H), 1.22 (d, *J* = 6.8 Hz, 3H), 1.16 (d, *J* = 6.6 Hz, 3H), 1.11 (d, *J* = 6.6 Hz, 3H), 0.95 (s, 3H).
^{13}C NMR (126 MHz, $CDCl_3$) δ 192.35, 154.64, 144.85, 144.48, 143.66, 133.33, 132.02, 130.82, 128.73, 126.07, 125.80, 125.27, 83.08, 80.62, 54.94, 44.39, 43.16, 37.48, 30.01, 29.53, 28.76, 28.47, 28.06, 27.78, 27.24, 26.22, 22.18, 22.16.
HRMS-ESI$^+$: (m/z) calcd. for $C_{32}H_{47}N_2O_2^+$ 491.3632, found 491.3636.

Synthesis of [Ru(^{i}Pr$_2$CAAC-5-Boc)] S16

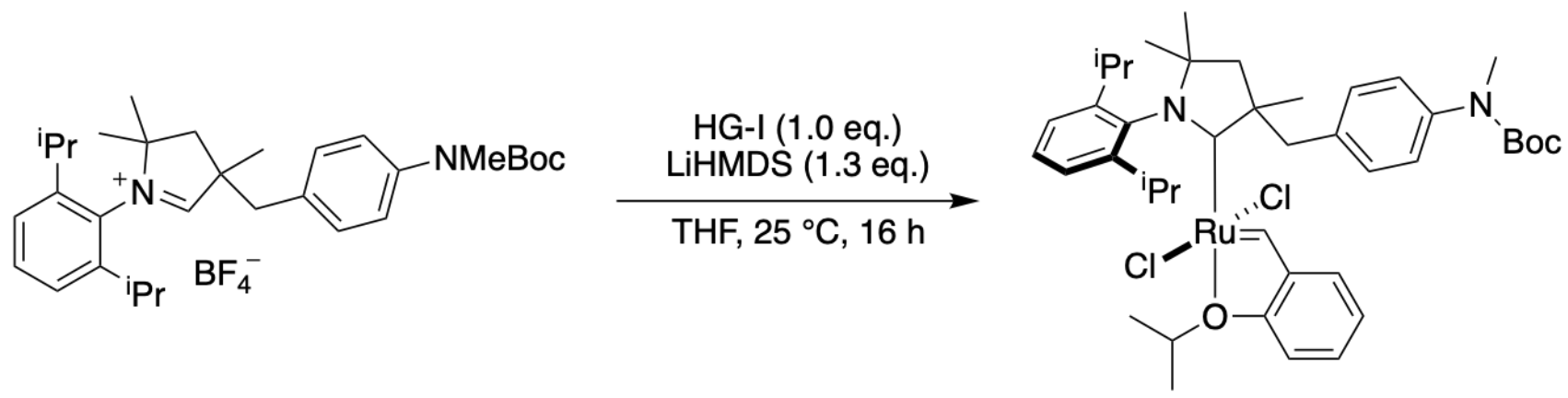

S16 (63%)

To a suspension of **^{i}Pr$_2$CAAC-5-H BF$_4$ S15** (1.13 g, 1.95 mmol, 1.3 eq.) dissolved in THF (30 mL), a solution of 1 M LiHMDS in THF (1.95 mL, 1.95 mmol, 1.95 eq.), followed by **HG-I** (1.74 g, 1.50 mmol, 1.0 eq.) were added. The reaction mixture was stirred at 25 °C for 16 hours. The crude product was filtered through neutral Alox, the solvent was removed *in vacuo*, and the residue was purified by column chromatography (SiO_2, PE/EA, 9:1) to afford the **[Ru(^{i}Pr$_2$CAAC-5-Boc)] S16** (763 mg, 63%) as a green solid.
^{1}H NMR (500 MHz, CD_2Cl_2) δ 16.37 (s, 1H), 7.64 (t, *J* = 7.8 Hz, 1H), 7.56 (ddd, *J* = 8.3, 7.3, 1.8 Hz, 1H), 7.48 (d, *J* = 8.0 Hz, 2H), 7.36 (d, *J* = 8.4 Hz, 2H), 7.24 (d, *J* = 8.4 Hz, 2H), 7.00 (d, *J* = 8.4 Hz, 1H), 6.89 (td, *J* = 7.4, 0.8 Hz, 1H), 6.84 (dd, *J* = 7.6, 1.8 Hz, 1H), 5.31–5.30 (m, 1H), 5.17 (p, *J* = 6.1 Hz, 1H), 4.45 (d, *J* = 12.7 Hz, 1H), 3.83 (d, *J* = 12.7 Hz, 1H), 3.26 (s, 3H), 3.03 (hept, *J* = 13.2, 6.5 Hz, 2H), 2.50 (d, *J* = 12.5 Hz, 1H), 1.91 (d, *J* = 12.5 Hz, 1H), 1.88 (s, 3H), 1.77 (dd, *J* = 6.1, 3.1 Hz, 6H), 1.46 (s, 9H), 1.39 (s, 3H), 1.32 (s, 3H), 1.27 (t, *J* = 6.6 Hz, 6H), 0.71 (d, *J* = 6.5 Hz, 3H), 0.63 (d, *J* = 6.4 Hz, 3H).
^{13}C NMR (126 MHz, CD_2Cl_2) δ 294.33, 266.98, 154.97, 153.20, 148.85, 148.65, 143.16, 143.14, 142.96, 136.86, 135.63, 131.27, 131.25, 131.22, 131.10, 129.95, 126.23, 126.19, 125.46, 123.85, 122.38, 113.58, 80.32, 78.60, 75.55, 61.41, 48.57, 46.04, 37.58, 31.30, 29.18, 29.03, 28.63, 28.47, 27.00, 26.42, 25.11, 24.55, 24.42, 22.38, 22.28.
HRMS-ESI$^+$: (m/z) calcd. for $C_{42}H_{58}ClN_2O_3Ru^+$ 775.3174, found 775.3173.

Synthesis of [Ru(^{i}Pr$_2$CAAC-5)] S17

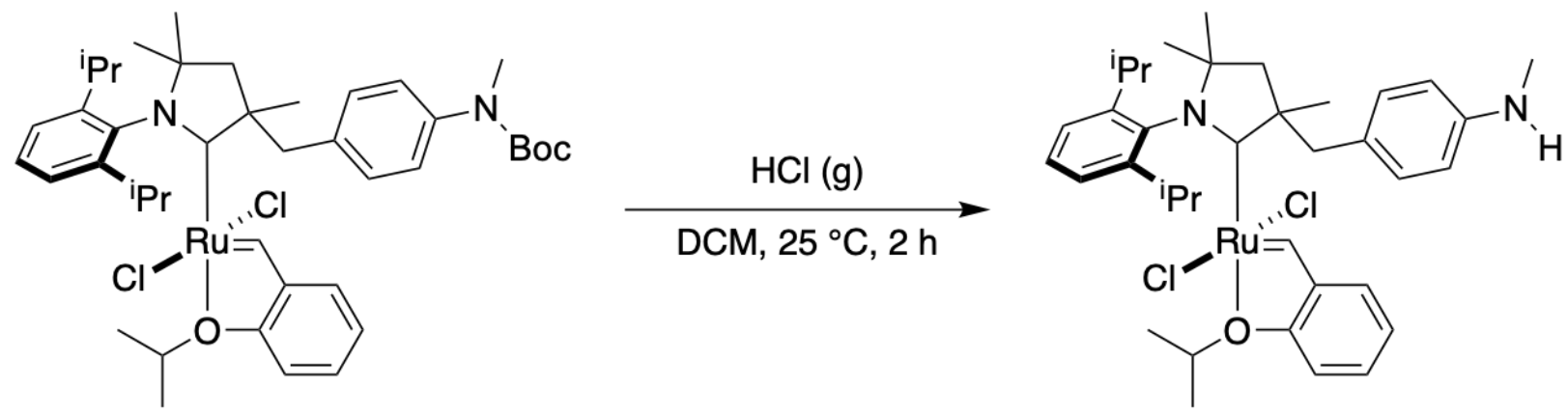

S17 (quant.)

[Ru(^{i}Pr$_2$CAAC-5-Boc)] S16 (494 mg, 0.61 mmol, 1.0 eq.) was dissolved in DCM (6.0 mL). HCl gas (generated from dropwise addition of H_2SO_4 to NH_4Cl) was bubbled through the reaction mixture until full conversion was achieved (as monitored by TLC, PE/EA, 6:1). Upon completion, the solvent was removed *in vacuo*, and the crude was purified by column chromatography (SiO_2, PE/EA, 2:1) and precipitated (DCM/hex) to afford **[Ru(^{i}Pr$_2$CAAC-5)] S17** (431 mg, quant.) as a green solid.

^{1}H NMR (500 MHz, CD_2Cl_2) δ 16.34 (s, 1H), 11.14 (br, 1H), 7.67–7.63 (m, 3H), 7.60–7.53 (m, 3H), 7.49 (d, *J* = 7.5 Hz, 2H), 7.00 (d, *J* = 8.4 Hz, 1H), 6.90 (td, *J* = 7.4, 0.8 Hz, 1H), 6.84 (dd, *J* = 7.6, 1.7 Hz, 1H), 5.18 (hept, *J* = 6.2 Hz, 1H), 4.51 (d, *J* = 12.6 Hz, 1H), 3.92 (d, *J* = 12.7 Hz, 1H), 3.03 (s, 3H), 3.11–2.95 (m, 2H), 2.47 (d, *J* = 12.5 Hz, 1H), 1.90 (d, *J* = 12.5 Hz, 1H), 1.87 (s, 3H), 1.77 (dd, *J* = 6.1, 4.7 Hz, 6H), 1.40 (s, 3H), 1.32 (s, 3H), 1.27 (dd, *J* = 7.7, 6.7 Hz, 6H), 0.71 (d, *J* = 6.5 Hz, 3H), 0.63 (d, *J* = 6.4 Hz, 3H).

^{13}C NMR (126 MHz, CD_2Cl_2) δ 294.32, 266.47, 153.22, 148.80, 148.59, 143.09, 140.13, 136.77, 136.54, 132.82, 131.18, 130.00, 126.26, 126.22, 123.85, 122.62, 122.39, 113.58, 78.64, 75.61, 61.14, 48.53, 46.14, 31.31, 29.10, 29.05, 28.63, 26.99, 26.40, 25.10, 24.54, 24.40, 22.36, 22.28.

HRMS-ESI$^+$: (m/z) calcd. for $C_{37}H_{50}ClN_2ORu^+$ 675.2650, found 675.2659.

Ru(^{i}Pr$_2$CAAC-5) amine S17 functionalization

Synthesis of 4-sulfamoylbenzoyl chloride (S18)

Prepared according to the literature.[70]

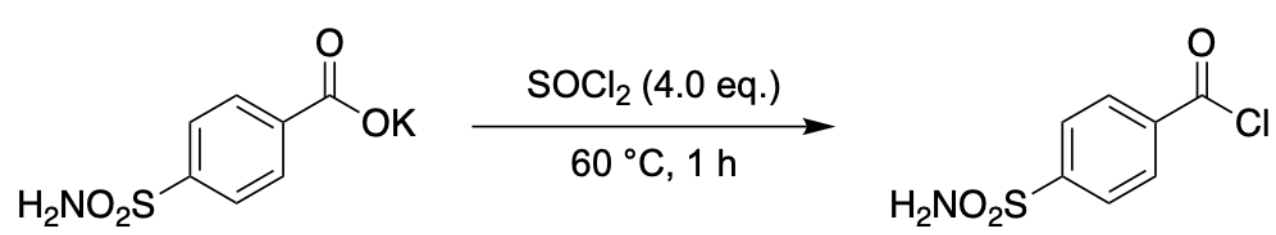

S18 (51%)

Oven-dried potassium 4-sulfamoylbenzoate (2.00 g, 8.36 mmol, 1.0 eq.) was mixed with thionyl chloride (3.98 g, 33.4 mmol, 4.0 eq.) and heated at 60 °C for 1 hour in a Schlenk flask. The reaction mixture was cooled to 25 °C and washed with hexane (3 x 10 mL). The crude was recrystallized from boiling benzene, filtered, and dried *in vacuo* to afford 4-sulfamoylbenzoyl chloride (**S18**, 931 mg, 51%) as a white crystalline solid.

^{1}H NMR (500 MHz, $CDCl_3$) δ 8.27 (d, *J* = 8.6 Hz, 2H), 8.08 (d, *J* = 8.6 Hz, 2H), 4.93 (s, 2H). The ^{1}H NMR data were in agreement with the reported values.

Synthesis of [Ru(^{i}Pr$_2$CAAC-5-SO$_2$NH$_2$)] S19

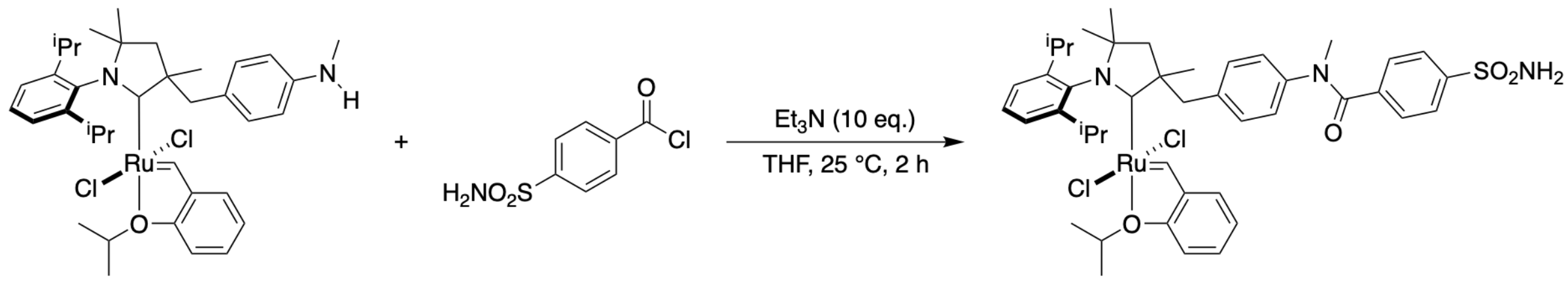

S19 (54%)

To a suspension of **[Ru(^{i}Pr$_2$CAAC-5)] S17** (17.8 mg, 25.0 μmol, 1.0 eq.) and triethylamine (25.3 mg, 34.9 μL, 0.25 mmol, 10 eq.) in THF (0.5 mL), freshly prepared 4-sulfamoylbenzoyl chloride (**S18**, 5.5 mg, 25.0 μmol, 1.0 eq.) was added. The reaction mixture was stirred at 25 °C for 2 hours. Upon completion, the solvent was removed *in vacuo*, and the crude was purified by column chromatography (SiO_2, hex/EA gradient) and precipitated (DCM/hex) to afford **[Ru(^{i}Pr$_2$CAAC-5-SO$_2$NH$_2$)] S19** (12.0 mg, 54%) as a green solid.

^{1}H NMR (500 MHz, CD_2Cl_2) δ 16.34 (s, 1H), 7.72 (d, *J* = 8.0 Hz, 2H), 7.65 (t, *J* = 7.8 Hz, 1H), 7.57 (ddd, *J* = 8.4, 7.3, 1.7 Hz, 1H), 7.48 (dq, *J* = 7.6, 1.6 Hz, 2H), 7.44 (d, *J* = 8.1 Hz, 2H), 7.29 (d, *J* = 8.2 Hz, 2H), 7.06 (d, *J* = 7.8 Hz, 2H), 7.00 (d, *J* = 8.4 Hz, 1H), 6.90 (td, *J* = 7.4, 0.8 Hz, 1H), 6.83 (dd, *J* = 7.6, 1.7 Hz, 1H), 5.33 (m, 1H), 5.17 (hept, *J* = 6.0 Hz, 1H), 4.92 (s, 2H), 4.41 (d, *J* = 12.7 Hz, 1H), 3.74 (d, *J* = 12.7 Hz, 1H), 3.51 (s, 3H), 2.99 (ddq, *J* = 10.4, 6.6, 3.4 Hz, 2H), 2.39 (d, *J* = 12.5 Hz, 1H), 1.82 (d, *J* = 12.7 Hz, 1H), 1.79 (s, 3H), 1.76 (t, *J* = 5.7 Hz, 6H), 1.37 (s, 3H), 1.32 (s, 3H), 1.27 (d, *J* = 6.7 Hz, 6H), 0.69 (d, *J* = 6.5 Hz, 3H), 0.62 (d, *J* = 6.4 Hz, 3H).

^{13}C NMR (151 MHz, CD_2Cl_2) δ 294.36, 266.44, 168.97, 153.14, 148.73, 148.53, 143.04, 141.07, 137.78, 136.74, 132.06, 131.18, 129.98, 129.69, 127.07, 126.21, 123.84, 122.38, 113.55, 78.62, 75.51, 61.06, 48.52, 45.91, 31.12, 29.20, 28.99, 28.62, 26.91, 26.41, 25.14, 24.49, 24.39, 22.34, 22.27.

HRMS-ESI^{+}: (m/z) calcd. for $C_{44}H_{56}Cl_2N_3O_4RuS^{+}$ 894.2407, found 894.2409.

Synthesis of 2-azidoacetyl chloride (S20)

Prepared according to the literature.[71]

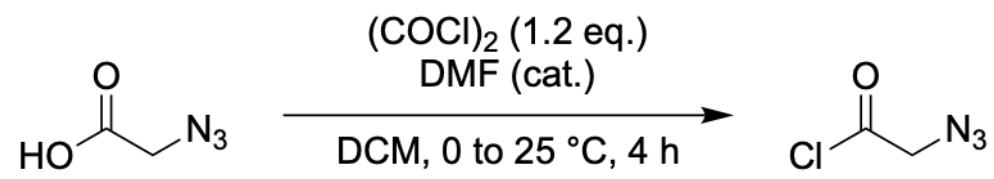

S20 (94%)

To a solution of 2-azidoacetic acid (118 mg, 87.6 μL, 1.17 mmol, 1.0 eq.) in DCM (5.0 mL), two drops of DMF were added at 25 °C. The solution was cooled to 0 °C, and oxalyl chloride (178 mg, 121 μL, 1.40 mmol, 1.2 eq.) was added dropwise. The reaction mixture was warmed to 25 °C and stirred for 4 hours. Then, excess oxalyl chloride was removed *in vacuo* to afford 2-azidoacetyl chloride (**S20**, 132 mg, 94%) as a colorless liquid. The crude product (containing DCM and DMF) was used directly in the next step without further purification.

^{1}H NMR (500 MHz, $CDCl_3$) δ 4.29 (s, 2H). The ^{1}H NMR data were in agreement with the reported values.

Synthesis of [Ru(^{i}Pr$_2$CAAC-5-N$_3$)] S21

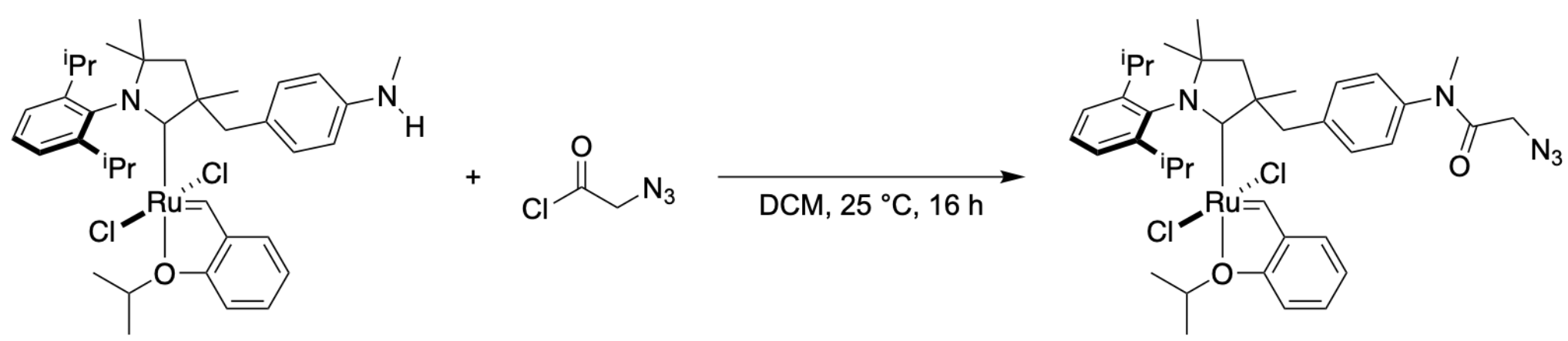

S21 (36%)

To a solution of **[Ru(^{i}Pr$_2$CAAC-5)] S17** (355 mg, 0.50 mmol, 1.0 eq.) in DCM (5.0 mL), freshly prepared 2-azidoacetyl chloride (**S20**, 89.6 mg, 0.75 mmol, 1.5 eq.) was added. The reaction mixture was stirred at 25 °C for 16 hours. The volatiles were evaporated under reduced pressure, and the crude product was purified by column chromatography (SiO_2, PE/EA, 2:1) to afford **[Ru(^{i}Pr$_2$CAAC-5-N$_3$)] S21** (142 mg, 36%) as a green powder.

^{1}H NMR (500 MHz, CD_2Cl_2) δ 16.35 (s, 1H), 7.66 (t, *J* = 7.8 Hz, 1H), 7.58 (ddd, *J* = 8.3, 7.3, 1.8 Hz, 1H), 7.52–7.45 (m, 4H), 7.22 (d, *J* = 8.2 Hz, 2H), 7.01 (d, *J* = 8.4 Hz, 1H), 6.91 (td, *J* = 7.4, 0.8 Hz, 1H), 6.85 (dd, *J* = 7.6, 1.7 Hz, 1H), 5.19 (dq, *J* = 12.6, 6.3 Hz, 1H), 4.51 (d, *J* = 12.6 Hz, 1H), 3.89 (d, *J* = 12.6 Hz, 1H), 3.65 (s, 2H), 3.32 (s, 3H), 3.03 (m, 2H), 2.49 (d, *J* = 12.5 Hz, 1H), 1.92 (d, *J* = 12.5 Hz, 1H), 1.88 (s, 3H), 1.78 (dd, *J* = 6.1, 4.0 Hz, 6H), 1.41 (s, 3H), 1.34 (s, 7H), 1.28 (dd, *J* = 6.7, 5.0 Hz, 6H), 0.72 (d, *J* = 6.5 Hz, 3H), 0.63 (d, *J* = 6.4 Hz, 3H).

^{13}C NMR (151 MHz, CD_2Cl_2) δ 294.30, 266.52, 167.55, 153.17, 148.76, 148.54, 143.05, 141.03, 139.35, 136.73, 132.76, 131.19, 130.00, 127.15, 126.24, 126.21, 123.85, 122.40, 113.56, 78.63, 75.54, 61.16, 51.08, 48.56, 46.20, 37.69, 31.29, 29.10, 29.03, 28.62, 26.96, 26.38, 25.05, 24.51, 24.37, 22.34, 22.24.

HRMS-ESI$^+$: (m/z) calcd. for $C_{39}H_{52}Cl_2N_5O_2Ru^+$ 794.2536, found 794.2537.

Synthesis of [Ru(^{i}Pr$_2$CAAC-5-Br)] S22

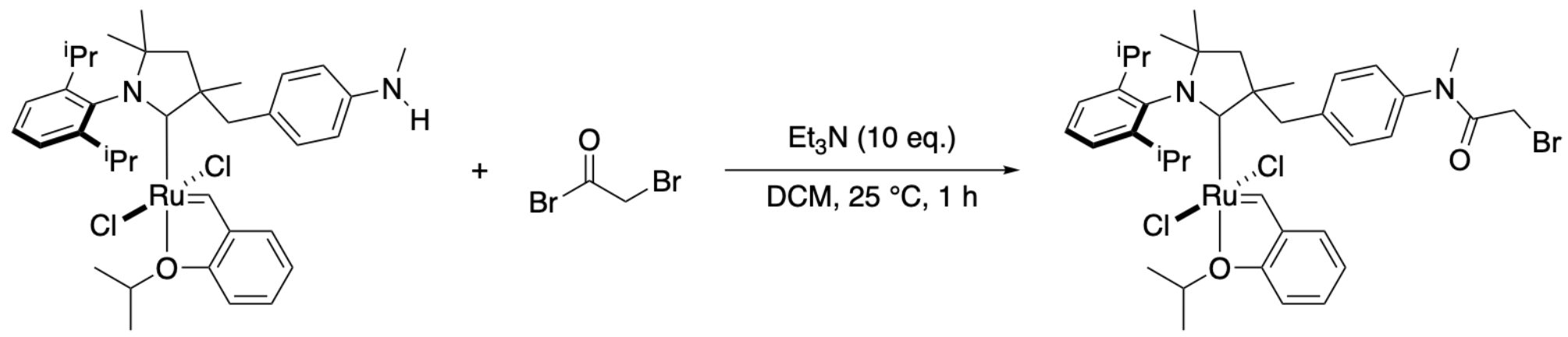

S22 (59%)

To a solution of **[Ru(^{i}Pr$_2$CAAC-5)] S17** (71.1 mg, 0.10 mmol, 1.0 eq.) in DCM (2.0 mL), triethylamine (101 mg, 141 µL, 1.00 mmol, 10 eq.), followed by bromoacetyl bromide (30.3 mg, 13.1 µL, 0.15 mmol, 1.5 eq.) were added. The reaction was stirred at 25 °C for 1 hour. The volatiles were removed *in vacuo*, and the crude product was purified by column chromatography (SiO_2, PE/EA, 3:1 to 2:1) to afford **[Ru(^{i}Pr$_2$CAAC-5-Br)] S22** (48.8 mg, 59%) as a green powder.

^{z1}H NMR (500 MHz, CD_2Cl_2) δ 16.35 (s, 1H), 7.66 (t, *J* = 7.8 Hz, 1H), 7.58 (ddd, *J* = 8.3, 7.3, 1.8 Hz, 1H), 7.50 (dq, *J* = 7.6, 1.7 Hz, 4H), 7.35–7.20 (m, 2H), 7.01 (d, *J* = 8.4 Hz, 1H), 6.91 (td, *J* = 7.4, 0.8 Hz, 1H), 6.85 (dd, *J* = 7.6, 1.7 Hz, 1H), 5.19 (hept, *J* = 6.5 Hz, 1H), 4.52 (d, *J* = 12.7 Hz, 1H), 3.89 (d, *J* = 12.6 Hz, 1H), 3.74 (s, 2H), 3.31 (s, 3H), 3.03 (dp, *J* = 11.3, 6.5 Hz, 2H), 2.51 (d, *J* = 12.5 Hz, 1H), 1.94 (d, *J* = 12.4 Hz, 1H), 1.89 (s, 3H), 1.78 (dd, *J* = 6.1, 3.7 Hz, 6H), 1.41 (s, 3H), 1.34 (s, 3H), 1.30–1.26 (m, 6H), 0.72 (d, *J* = 6.5 Hz, 3H), 0.63 (d, *J* = 6.4 Hz, 3H).

^{13}C NMR (151 MHz, CD_2Cl_2) δ 294.26, 266.55, 166.47, 153.17, 148.75, 148.55, 143.05, 141.99, 139.24, 136.73, 132.62, 131.18, 129.99, 127.09, 126.23, 126.20, 123.84, 122.39, 113.56, 78.63, 75.54, 61.17, 48.54, 46.16, 38.25, 31.97, 31.27, 29.15, 29.02, 28.62, 27.93, 26.96, 26.39, 25.06, 24.51, 24.38, 23.04, 22.34, 22.25, 14.27.

HRMS-ESI$^+$: (m/z) calcd. for $C_{39}H_{52}BrCl_2N_2O_2Ru^+$ 831.1628, found 831.1621.

Synthesis of perfluorophenyl hex-5-ynoate (S23)

Prepared according to the literature.[72]

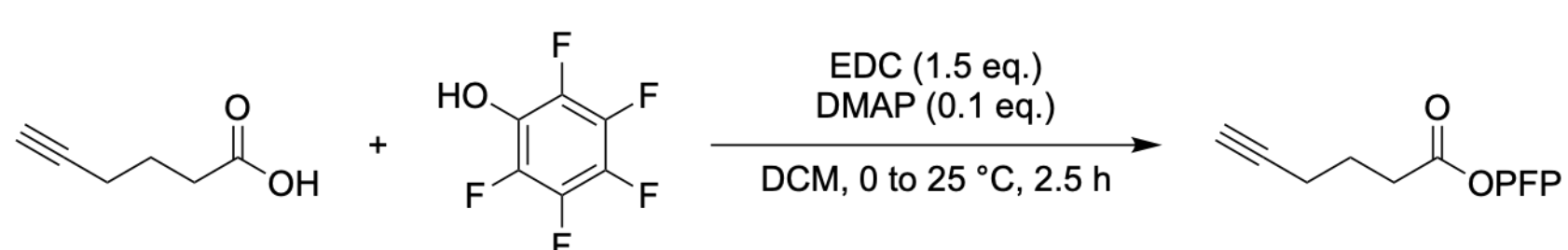

S24 (79%)

To a solution of 5-hexynoic acid (561 mg, 545 µL, 5.00 mmol, 1.0 eq.) and pentafluorophenol (1.38 g, 7.50 mmol, 1.5 eq.) dissolved in DCM (12 mL), EDC (1.44 g, 7.50 mmol, 1.5 eq.) and DMAP (61.1 mg, 0.50 mmol, 0.1 eq.) were added at 0 °C. The reaction mixture was stirred at 25 °C for 2.5 hours. Upon completion of the reaction, the suspension was filtered, washed with Et_2O, and the filtrate was concentrated *in vacuo*. The crude residue was purified by column chromatography (SiO_2, PE/EA, 98:2 to 95:5) to afford perfluorophenyl hex-5-ynoate (**S23**, 1.10 g, 79%) as a colorless liquid.

^{1}H NMR (500 MHz, $CDCl_3$) δ 2.84 (t, *J* = 7.4 Hz, 2H), 2.36 (td, *J* = 6.9, 2.6 Hz, 2H), 2.03 (t, *J* = 2.7 Hz, 1H), 2.03–1.95 (q, *J* = 7.2 Hz, 2H). The ^{1}H NMR data were in agreement with the reported values.

Bioconjugation via supramolecular anchoring

Assembly of [Ru(^{i}Pr$_2$CAAC-5-SO$_2$NH$_2$)] · hCA II

The cofactor **[Ru(^{i}Pr$_2$CAAC-5-SO$_2$NH$_2$)] S19** was incubated for 10 minutes with human carbonic anhydrase II (hCA II) at 37 °C in the corresponding reaction buffer.

Binding affinity of [Ru(^{i}Pr$_2$CAAC-5-SO$_2$NH$_2$)] (S19) for hCA II^{wt}

The binding affinity (K_d) of **[Ru(^{i}Pr$_2$CAAC-5-SO$_2$NH$_2$)] S19** for **hCA II** was determined using a competitive displacement assay with dansylamide (**DNSA**) as described in the literature.[73]

The following stock solutions were freshly prepared in PBS buffer (100 mM, pH 7.0):

- **DNSA** (800 µM, 400 µM) in DMSO
- **[Ru(^{i}Pr$_2$CAAC-5-SO$_2$NH$_2$)] S19** (4 mM, 400 µM, 40 µM) in DMSO
- **hCA II** (0.278 µM) in PBS buffer (pH 7.4)

All the titration experiments were performed in triplicate. The K_d of **DNSA** was determined by titrating 0.25 µM hCA II^{wt} (180 µL) with **DNSA** concentrations ranging from 0–40 µM in a total assay volume of 200 µL. The K_d (**DNSA**) was then determined by fitting the data to equation **1** using Prism 11.0 software:

$$r = \frac{[DNSA]}{K_d(DNSA) + [DNSA]} \qquad (\boldsymbol{eq.1})$$

The K_d for **[Ru(^{i}Pr$_2$CAAC-5-SO$_2$NH$_2$)] S19** was then determined by competitive binding with **DNSA**. A fixed concentration of 20 µM DNSA (5 µL) and 0.25 µM **hCA II** (180 µL) was titrated against **[Ru(^{i}Pr$_2$CAAC-5-SO$_2$NH$_2$)] S19** from 0–300 µM. The K_d value of **[Ru(^{i}Pr$_2$CAAC-5-SO$_2$NH$_2$)] S19** was determined by fitting the data to equation **2** using Prism 11.0 software:

$$r = \frac{1}{1 + (K_d(DNSA)/[DNSA])(1 + [Ru]/K_d(Ru))} \qquad (\boldsymbol{eq.2})$$

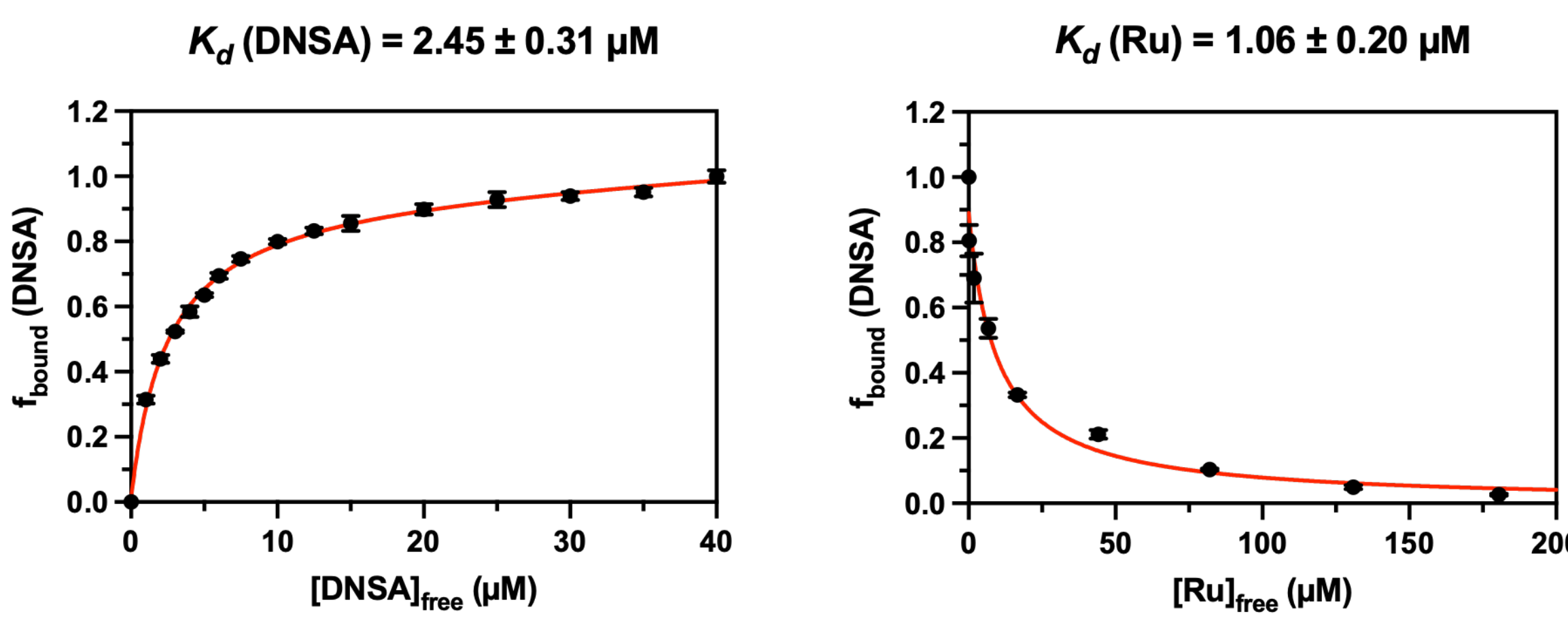

Figure S1. Determination of **DNSA** and **[Ru(^{i}Pr$_2$CAAC-5-SO$_2$NH$_2$)] S19** binding affinity (K_d) to human carbonic anhydrase II (**hCA II**) by competitive displacement assay. The fluorescence emission was monitored at 470 nm (excitation at 280 nm).

Bioconjugation via CuAAC

Solid-Phase Peptide Synthesis of SS01 (S24)

Prepared according to the literature.[74]

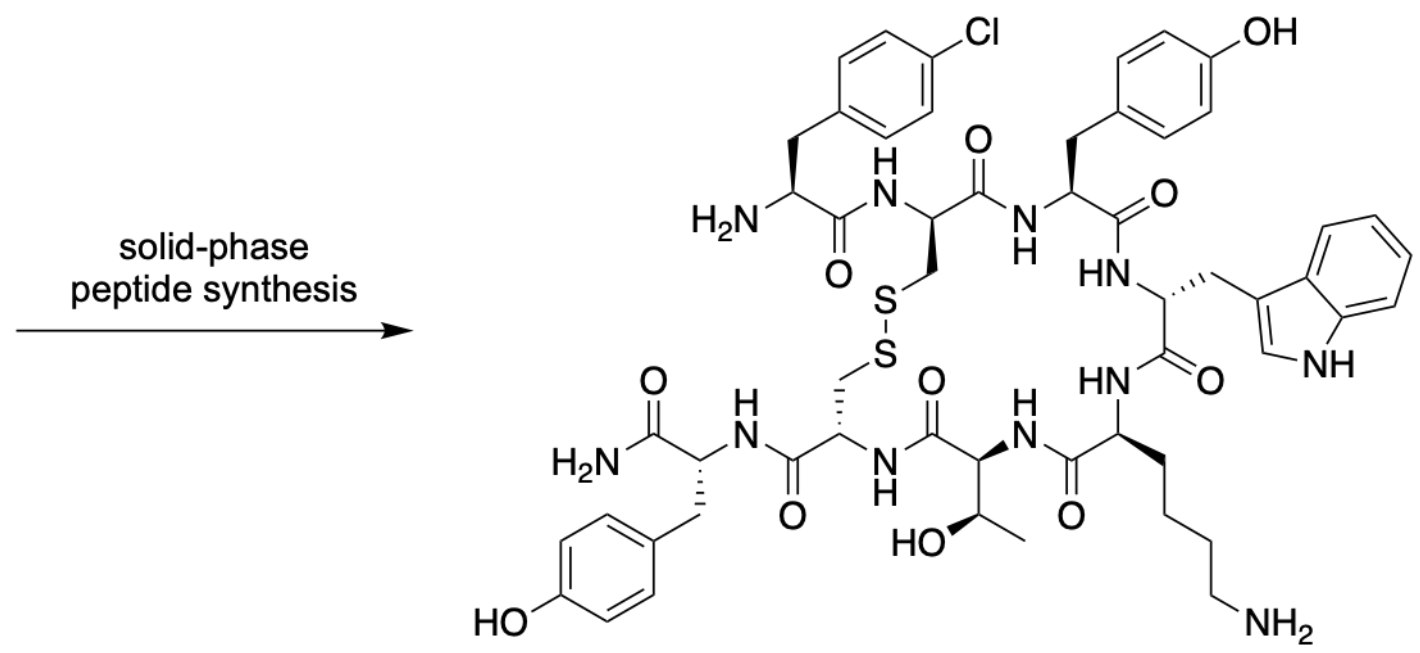

SS01 (S24)

H_2N-*p*-Cl-Phe-cyclo(*D*-Cys-Tyr-*D*-Trp-Lys-Thr-Cys)-*D*-Tyr-$CONH_2$

All the peptides were synthesized by standard Fmoc solid-phase peptide synthesis on Rink Amide MBHA resin using a Liberty Blue peptide synthesizer from CEM. The Fmoc-protected *p*-Cl-Phe-OH, *D*-Tyr(^{t}Bu)-OH, *L*-Cys(Acm)-OH, *L*-Thr(tBu)-OH, *L*-Lys(Boc)-OH, *D*-Trp(Boc)-OH, *L*-Tyr(^{t}Bu)-OH, and *D*-Cys(Acm)-OH (Acm = acetamido methyl) were used in a 4.2-equivalent excess based on the original substitution of the resin. The couplings were mediated by *N*,*N*-diisopropylcarbodiimide (DIC, 1.0 M in DMF) and Oxyma (0.1 M in DMF) in DMF. Fmoc removal was achieved with 20% piperidine in *N*,*N*-dimethylformamide (DMF). Cyclization was performed on the resin in DMF (0.1 M) with 10 equivalents of iodine at 25 °C for 16 hours. The resin was then washed five times with DMF, followed by three washes with DCM, dried under a flow of N_2, and stored in the fridge. A small amount of the peptide (4 mg) was cleaved after the cyclization using 200 µL of TFA (95%), H_2O (2.5%), and TIS (2.5%) at 25 °C for 2 hours, and then analyzed by LC-MS.

LC-MS (Method 1 at 290 nm): T_R = 6.247 min.

LC-MS (ESI$^+$ at 6.247 min): (m/z) 594.75 (calcd. for $[M+2Na]^{2+}$ 594.69), 630.35 (calcd. for $[M+4H_2O+2Na]^{2+}$ 630.71).

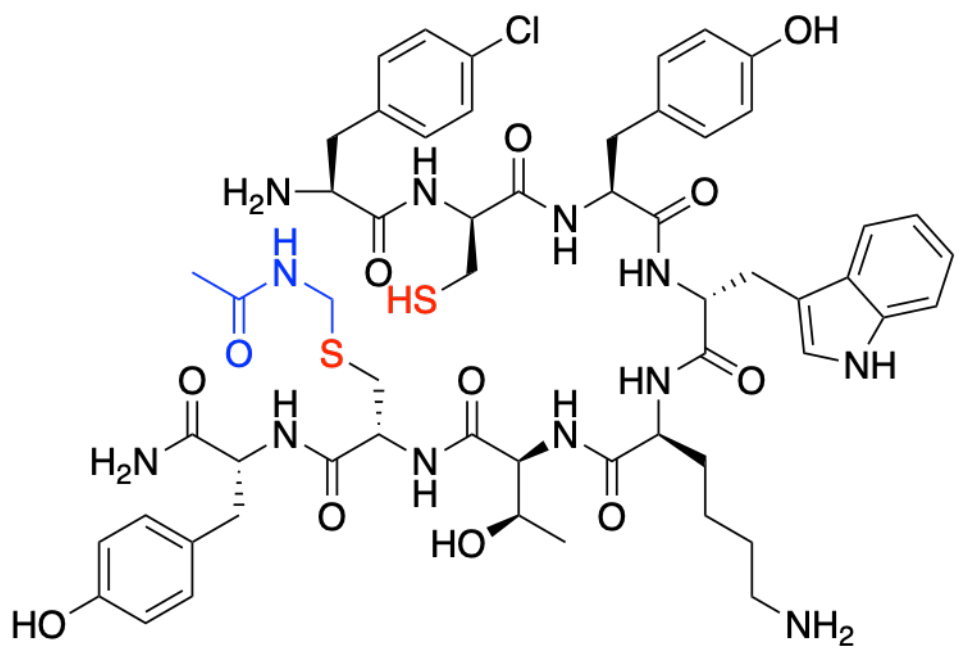

$[SS01_{ac}^{Acm}+2H]^{2+}$
$C_{57}H_{75}ClN_{12}O_{12}S_2^{2+}$
m/z: 609.24 (100.0%)
T_R = 6.101 min

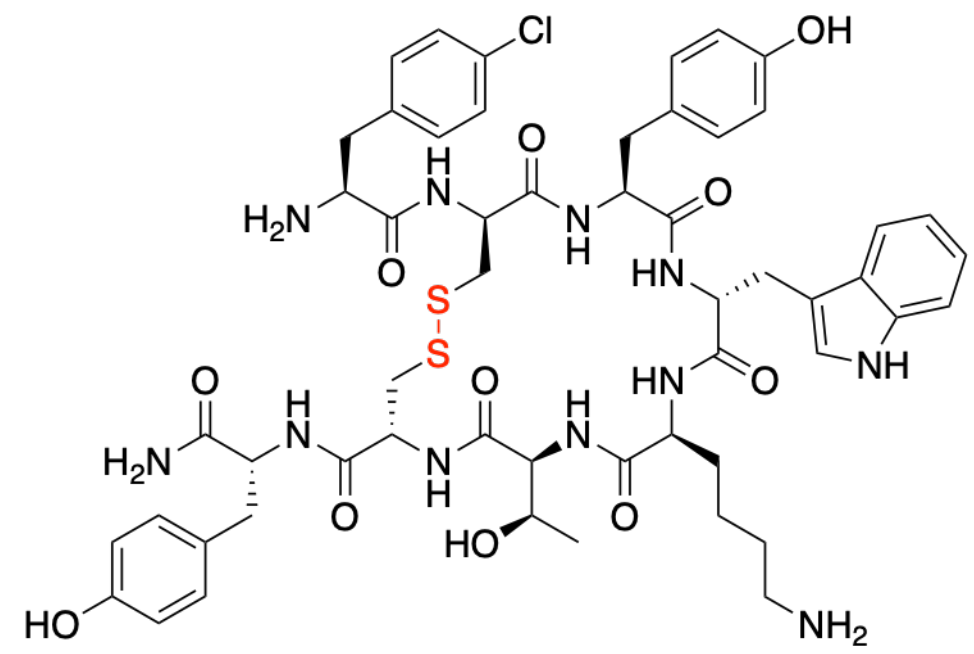

$[SS01_{cy}+2Na]^{2+}$
$C_{54}H_{66}ClN_{11}Na_2O_{11}S_2^{2+}$
m/z: 594.69 (100.0%)
T_R = 6.247 min

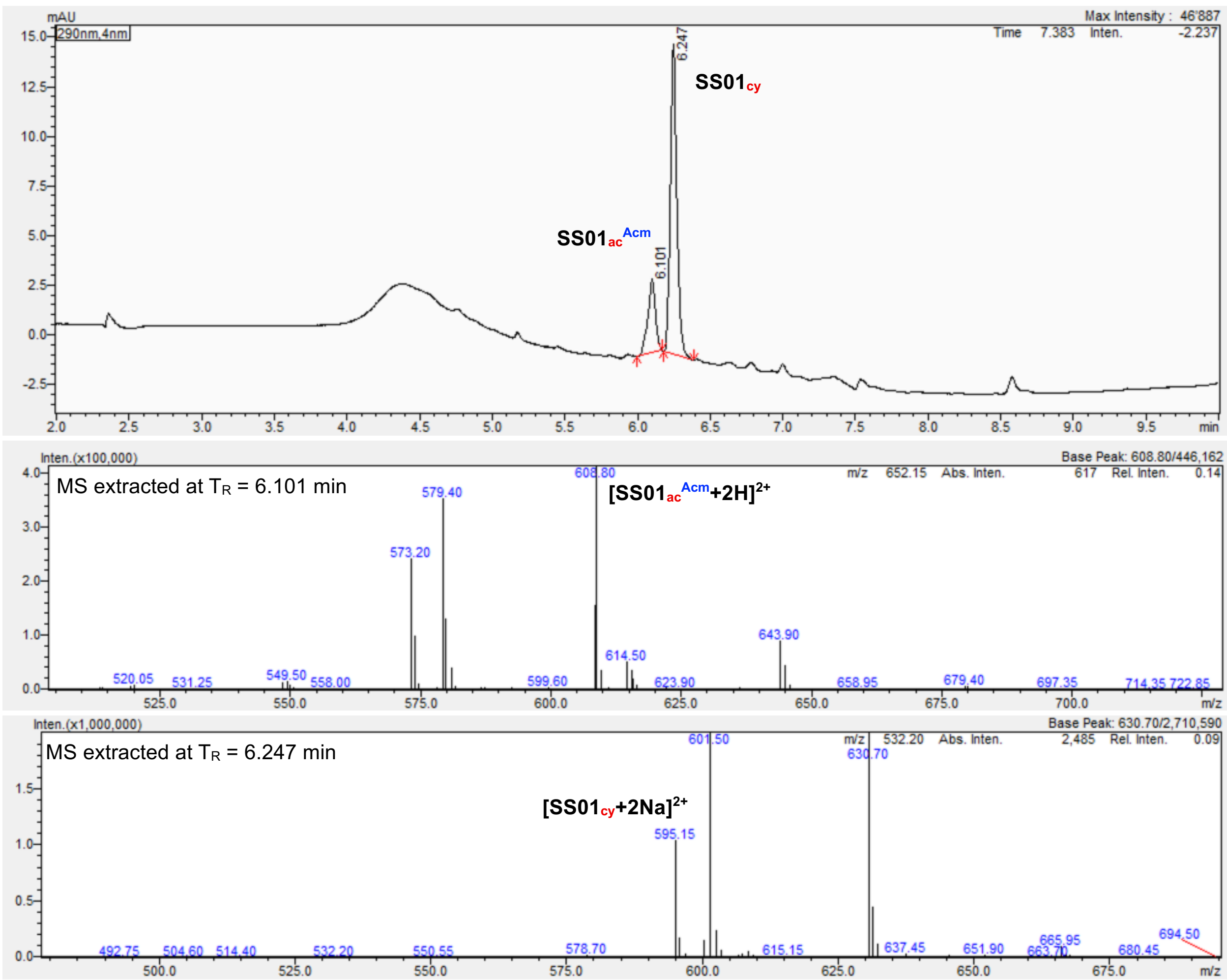

Figure S2. LC-MS analysis at 290 nm using Method 1 of the crude peptide after on-resin I_2-mediated cyclization and TFA/TIS/H_2O cleavage. The chromatogram displays two closely eluting species: the correctly cyclized, disulfide-bridged product (**$SS01_{cy}$**, T_R = 6.247 min) and an incompletely deprotected impurity retaining one acetamidomethyl (Acm) protecting group on one of the cysteines ($SS01_{ac}^{Acm}$, T_R = 6.101 min), identified by their respective extracted mass spectra.

Synthesis of SS01 alkyne S25

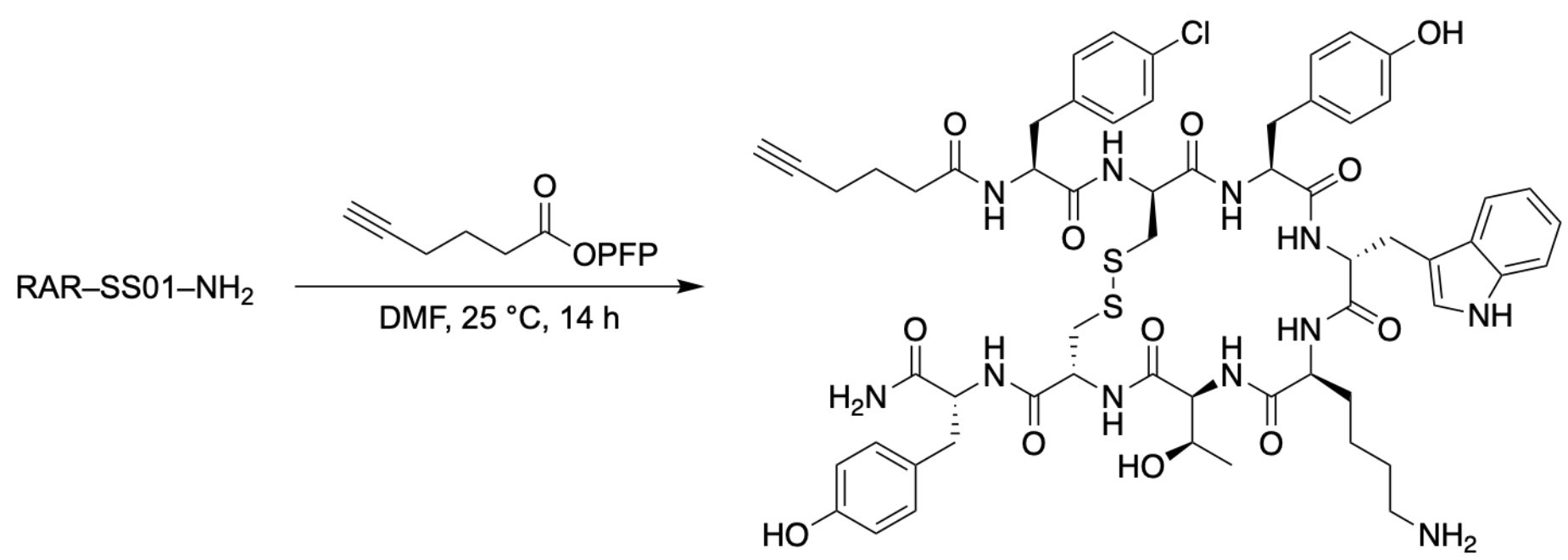

SS01 alkyne (S25)

RAR-SS01-NH$_2$ (625 mg, 0.25 mmol, 1.0 eq.) was swollen in DMF (3.0 mL) for 30 minutes, followed by two washings with DMF (3.0 mL) for 2 minutes. After removal of DMF, the resin was treated with a solution of DIPEA (0.41 mL, 2.50 mmol, 10 eq.) in DMF (1.0 mL) followed by a solution of perfluorophenyl hex-5-ynoate (**S23**, 104 mg, 0.38 mmol, 1.5 eq.) in DMF (1.0 mL). The reaction was shaken at 25 °C overnight. Then the coupling mixture was removed, and the resin was washed five times with DMF, followed by three times with DCM. After drying the resin under a stream of N$_2$, the peptide was cleaved with a 5 mL cleavage mixture containing TFA (95%), H$_2$O (2.5%), and TIS (2.5%) at 25 °C for 2 hours, then precipitated into ice-cold Et$_2$O. The peptide was kept in a freezer at –20 °C for 30 minutes, centrifuged (4'400 rpm, 4 °C, 20 min), decanted, resuspended in a mixture of H$_2$O/MeOH, and lyophilized to afford the **SS01 alkyne S25** (110 mg, 36%) as a white solid. The product was used without any further purification.

LC-MS (Method 1 at 290 nm): T_R = 7.297 min.

LC-MS (ESI$^+$ at 7.297 min): (m/z) 619.80 (calcd. for $[M+2H]^{2+}$ 619.73), 648.90 (calcd. for $[M+2H_2O+Na+H]^{2+}$ 648.73), 655.80 (calcd. for $[M+MeOH+H_2O+Na+H]^{2+}$ 655.74), 1296.40 (calcd. for $[M+2H_2O+Na]^+$ 1296.46), 1310.40 (calcd. for $[M+MeOH+H_2O+Na]^+$ 1310.48).

HRMS-ESI$^+$: (m/z) calcd. for $C_{60}H_{73}ClN_{11}O_{12}S_2^+$ 1238.4565, found 1238.4560.

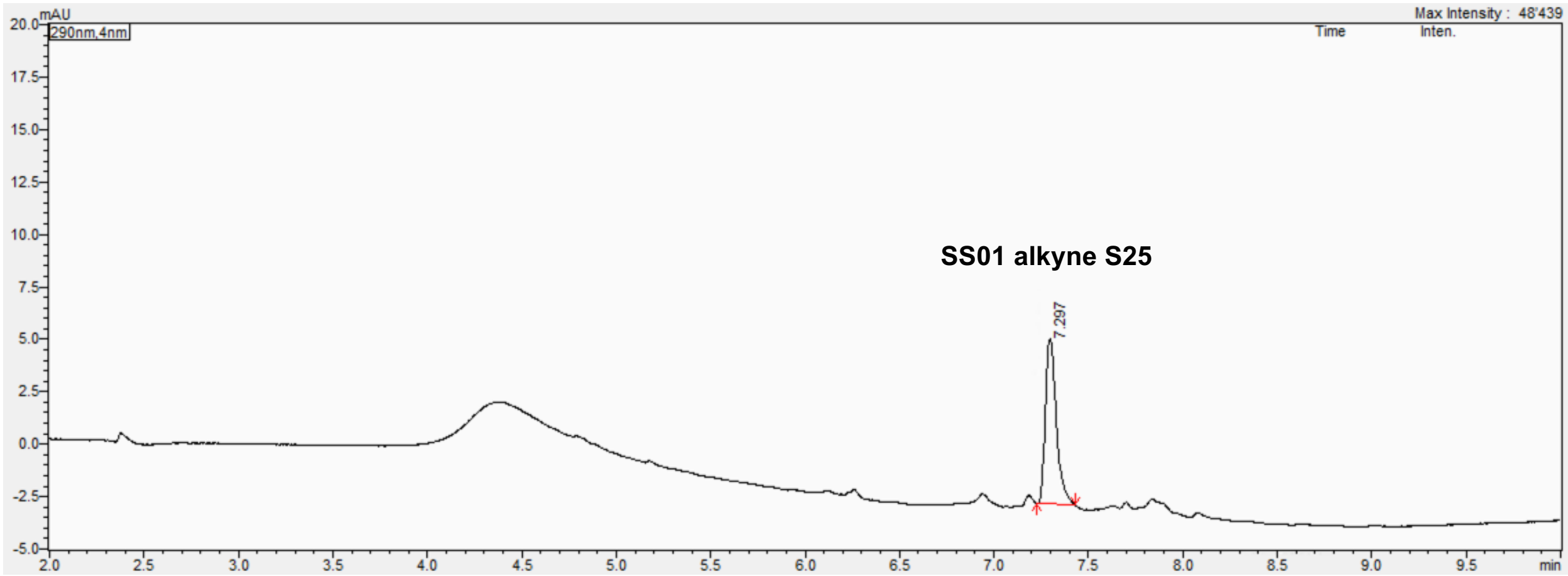

Figure S3. LC-MS chromatogram of peptide **SS01 alkyne S25** (T_R = 7.297 min) at 290 nm using Method 1.

Synthesis of [Ru(^{i}Pr$_2$CAAC-5)] · SS01

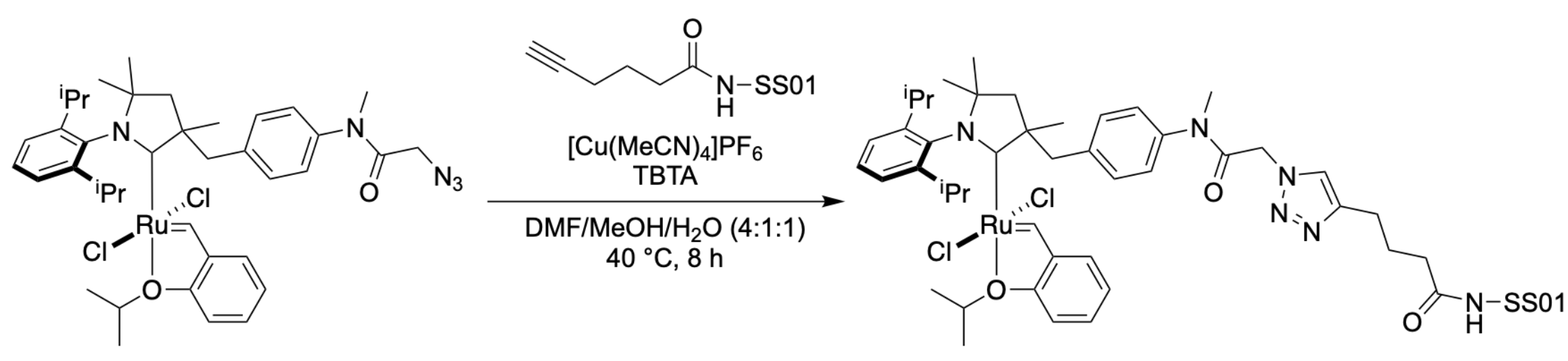

To a vial containing $[Cu(MeCN)_4]PF_6$ (1.0 eq.) and DMF (73.6 µL), 20 µL of a stock solution of **[Ru(^{i}Pr$_2$CAAC-5-N$_3$)] S21** (39.7 mg/mL in DMF, 1.0 eq.), 20 µL of a stock solution of TBTA (13.25 mg/mL in DMF, 0.5 eq.) and 20 µL of a stock solution of the peptide **SS01 alkyne S25** (62 mg/mL in DMF, 1.0 eq.) were added sequentially. To the reaction mixture, MeOH (33.2 µL) and H_2O (33.2 µL) were added, and the mixture was maintained at 40 °C for 8 hours. The crude product was then directly subjected to preparative RP-HPLC (T_R = 16.8 min) and lyophilized to afford the **[Ru(^{i}Pr$_2$CAAC-5)] · SS01** (3.5 mg, 17%).

LC-MS (Method 2 at 380 nm): T_R = 7.915 min.

LC-MS (ESI$^+$ at 7.915 min): (m/z) 1017.90 (calcd. for $[M+2H]^{2+}$ 1016.86).

HRMS-ESI$^+$: (m/z) calcd. for $C_{99}H_{124}Cl_3N_{16}O_{14}RuS_2^+$ 2031.7028, found 2031.7028.

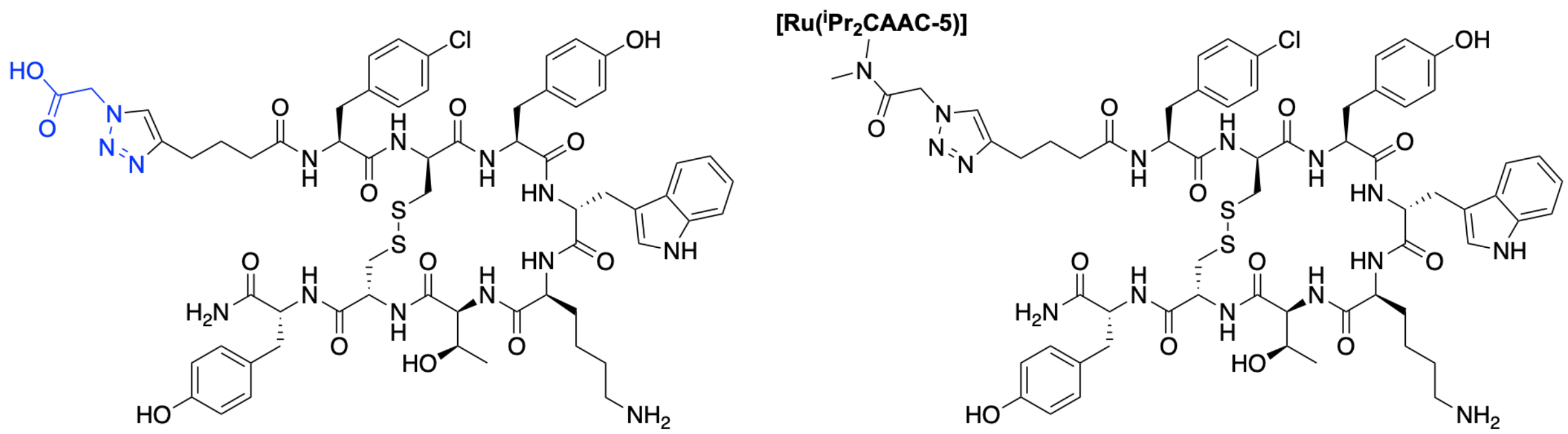

[SS01^{CO2H}+H+Na]$^{2+}$
$C_{62}H_{76}ClN_{14}NaO_{14}S_2^{2+}$
m/z: 681.2341 (100.0%)
T_R = 5.730 min

[[Ru(^{i}Pr$_2$CAAC-5)]·SS01+2H]$^{2+}$
$C_{99}H_{125}Cl_3N_{16}O_{14}RuS_2^{2+}$
m/z: 1016.8567 (100.0%)
T_R = 7.915 min

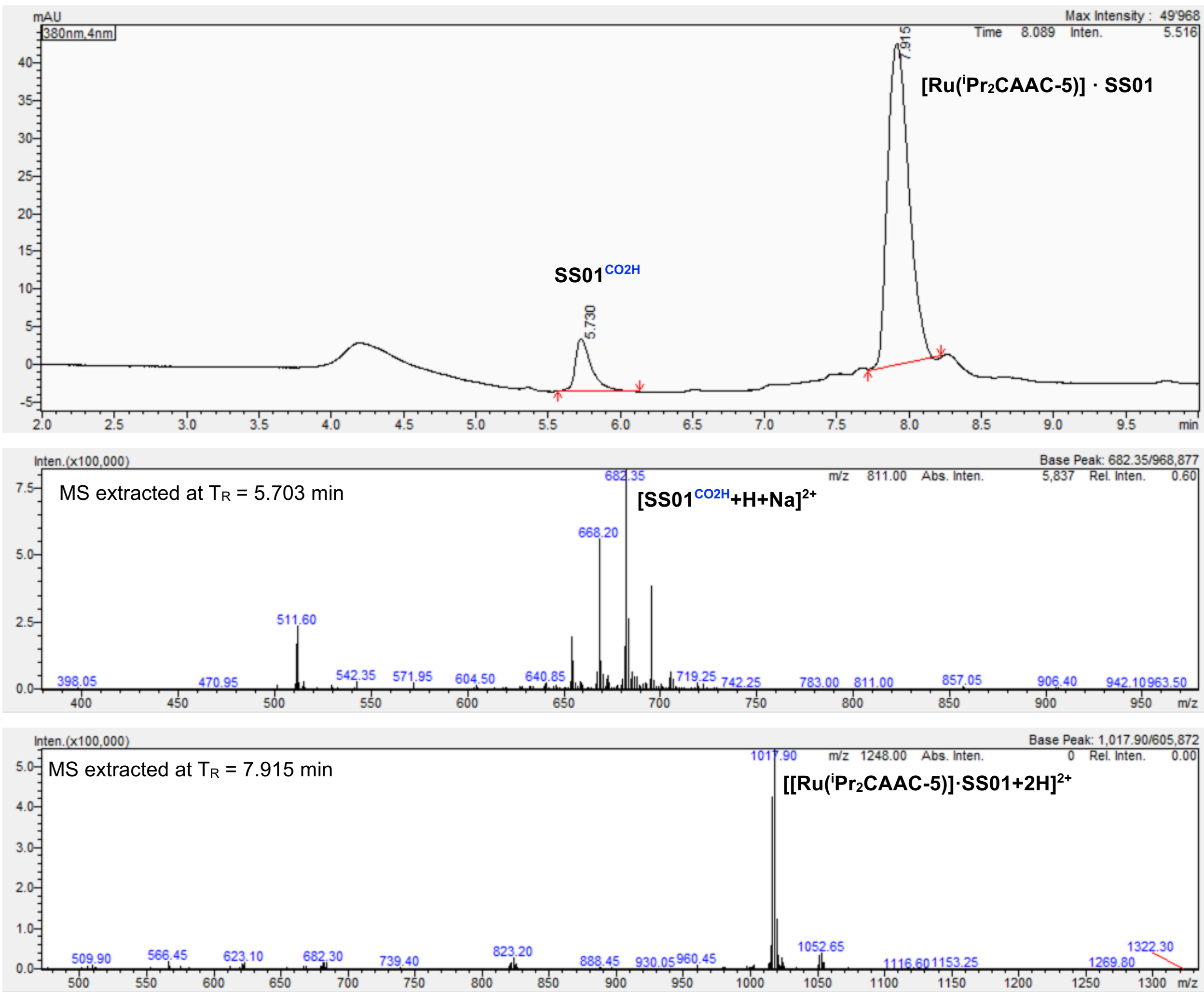

Figure S4. LC-MS analysis at 380 nm using Method 2 following CuAAC conjugation of the azide-functionalized **[Ru(iPr$_2$CAAC-5-N$_3$)]** cofactor to **SS01 alkyne S25**. Two species are observed: an impurity arising from amide hydrolysis of the bioconjugate to peptide **SS01^{CO2H}** (T_R = 5.730 min) and the desired bioconjugate **[Ru(^{i}Pr$_2$CAAC-5)] · SS01** (T_R = 7.915 min), identified by their respective extracted mass spectra.

Bioconjugation via cysteine alkylation

MBP-Ubiquitin K6C (Addgene plasmid #86587; http://n2t.net/addgene:86587; RRID:Addgene_86587) was a gift from Sharon Rozovsky.[75] MBP-ubiquitin K6C:

MKIHHHHHHEEGKLVIWINGDKGYNGLAEVGKKFEKDTGIKVTVEHPDKLEEKFPQVAATGDGPDIIFWA
HDRFGGYAQSGLLAEITPDKAFQDKLYPFTWDAVRYNGKLIAYPIAVEALSLIYNKDLLPNPPKTWEEIPAL
DKELKAKGKSALMFNLQEPYFTWPLIAADGGYAFKYENGKYDIKDVGVDNAGAKAGLTFLVDLIKNKHMN
ADTDYSIAEAAFNKGETAMTINGPWAWSNIDTSKVNYGVTVLPTFKGQPSKPFVGVLSAGINAASPNKEL
AKEFLENYLLTDEGLEAVNKDKPLGAVALKSYEEELVKDPRIAATMENAQKGEIMPNIPQMSAFWYAVRT
AVINAASGRQTVDEALKDAQTNSSSENLYFQSAQIFVCTLTGKTITLEVEPSDTIENVKAKIQDKEGIPPDQ
QRLIFAGKQLEDGRTLSDYNIQKESTLHLVLRLRGG

Black: His6-cytosolic MBP; Blue: TEV cleavage site; Orange: ubiquitin AA 2-76; Green: K6 mutated to C6

Protein Expression & Purification

Cells were plated on agar plates supplemented with carbenicillin (100 µg/mL) and incubated at 37 °C overnight. Pre-cultures were initiated by inoculating four test tubes containing 3 mL of LB medium (10 g/L tryptone, 5 g/L yeast extract, 10 g/L NaCl) supplemented with carbenicillin (100 µg/mL), then incubating at 37 °C overnight. These cultures were then used to inoculate 250 mL of LB medium supplemented with 0.2% glucose and carbenicillin (100 µg/mL) in shaker flasks. The cultures were grown at 37 °C for 6 hours, then cooled to 18 °C for 30 minutes before induction. Protein expression was induced by the addition of 0.5 mM isopropyl β-*D*-1-thiogalactopyranoside (IPTG). The cells were harvested after 24 hours, and the cell paste was resuspended in 50 mM sodium phosphate (pH 7.5) and 200 mM NaCl (amylose buffer). Cells were lysed on ice by sonication (FisherBrand) for 15 seconds for 2 minutes (30 seconds off time). The cell debris was removed by centrifugation at 20’000 g for 1 hour. The supernatant was loaded onto an amylose column, and the column was washed with the amylose buffer. The ubiquitin fusion protein MBP-Ubi was eluted using amylose buffer containing 20 mM maltose.

Cleavage of the fusion partner MBP was conducted by incubating a 1:8 molar ratio of cold-active TEV protease to MBP-Ubi at 4 °C for 36 hours. The protein was loaded onto a 1 mL histidine affinity column (Histrap FF, GE Healthcare) to remove MBP and TEV protease. The flow-through was pooled and concentrated. The buffer was exchanged into the bioconjugation buffer (PBS, pH 7.2) using a PD-10 desalting column (Sephadex™ G-25 resin), and then further concentrated. Protein purity was determined with a 4–15% Tris-glycine sodium dodecyl sulfate−polyacrylamide gel electrophoresis (SDS−PAGE) gel, Figure S5.

HRMS (ESI-Q-TOF): (m/z) deconvoluted average mass calcd. for MBP-Ubi K6C: $C_{2301}H_{3572}N_{598}O_{690}S_8$ 50’909.8, found 50’909.0.

HRMS (ESI-Q-TOF): (m/z) deconvoluted average mass calcd. for Ubi K6C: $C_{376}H_{623}N_{105}O_{120}S_1$ 8’565.0, found 8’565.6.

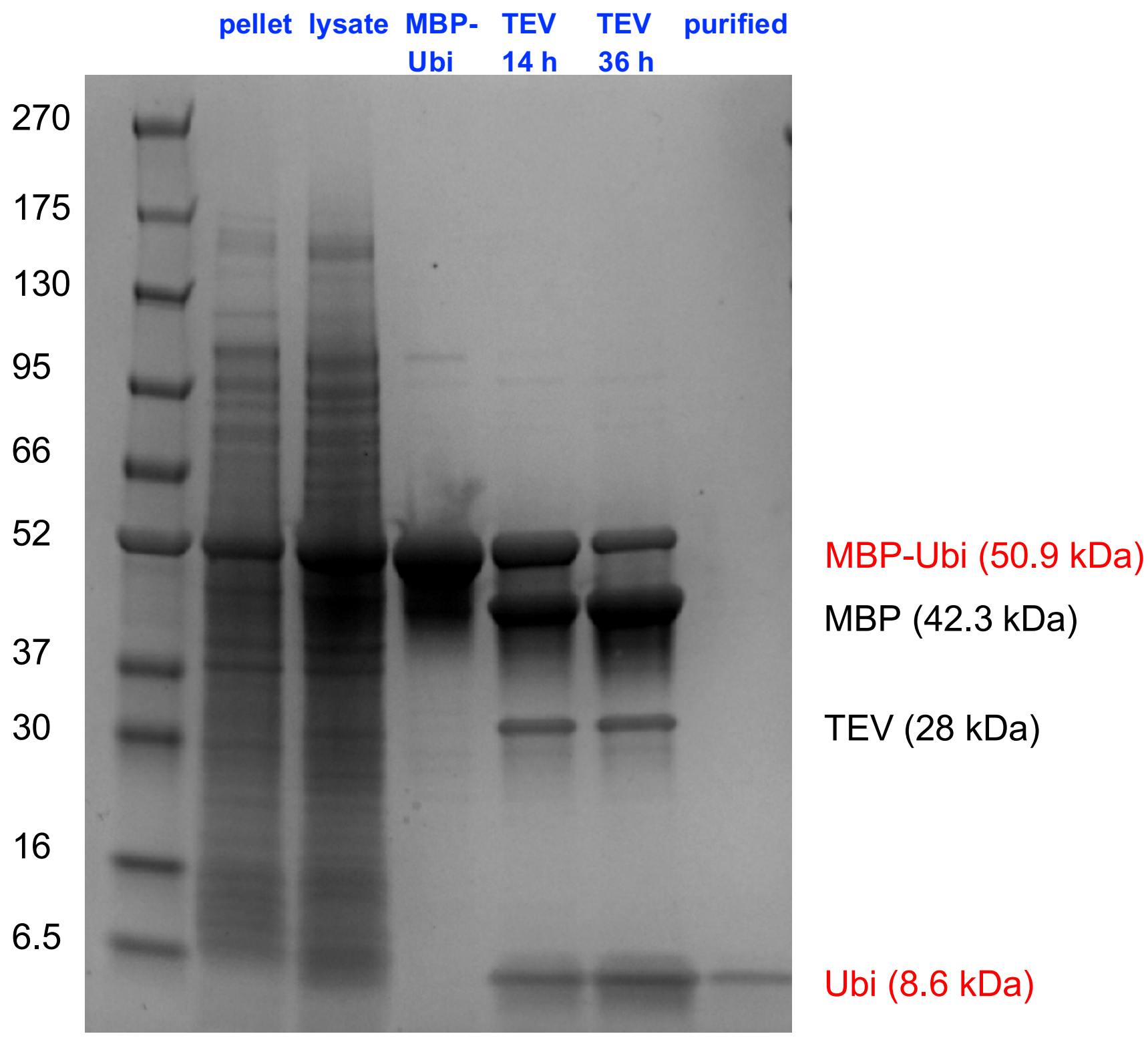

Figure S5. SDS page of MBP-Ubi K6C expression and purification. Protein ladder, cell pellet, cell lysate, purified MBP-Ubi K6C with amylose resin, cleavage of the fusion protein using TEV after 14 hours, and after 36 hours, purified Ubi K6C with Ni-NTA resin (from left to right).

Synthesis of Ru($^{i}Pr_2$CAAC-5) · MBP-Ubi K6C

Adapted from the literature.[76]

All steps were carried out in degassed buffers under an N_2 atmosphere. To a solution of MBP-Ubi K6C (1.00 mL, 3.3 mg/mL) in PBS buffer (50 mM, pH 7.2), ^{t}BuOH (100 µL) was added. The solution was stirred for 10 minutes, and then **[Ru($^{i}Pr_2$CAAC-5-Br)] S22** (0.27 mg, 324 nmol, 5.0 eq.) dissolved in ^{t}BuOH (200 µL) was added, and the reaction mixture was stirred at 25 °C. The progress of the reaction was monitored using the mBBr assay.[77] Once 95% conversion was achieved, the reaction mixture was diluted with 5 mL MQ H_2O, and the bioconjugate was concentrated employing Amicon Ultra-15 centrifugal filters (10 kDa cutoff). Two additional washing cycles were performed using 5 mL MQ H_2O. Then, the buffer was exchanged with 5 mL MES (50 mM, pH 6.0), followed by concentration using Amicon Ultra-15 centrifugal filters (10 kDa cutoff). The bioconjugate was transferred to a Slide-A-Lyzer dialysis chamber (6-8 kDa cutoff) and dialyzed against MES (50 mM, pH 6.0) buffer at 4 °C for 20 hours. The concentration of the bioconjugate **[Ru($^{i}Pr_2$CAAC-5)] · MBP-Ubi K6C** was determined by Bradford assay (using BSA for the standard curve). HRMS (ESI-Q-TOF): (m/z) deconvoluted average mass calcd. for **[Ru($^{i}Pr_2$CAAC-5)] · MBP-Ubi K6C**: $C_{2340}H_{3623}N_{600}O_{692}S_8Ru$ 51’660.0, found 51’658.5.

Substrate Synthesis

Synthesis of *N,N*-diallyl-4-methylbenzenesulfonamide (1)

Prepared according to the literature.[78]

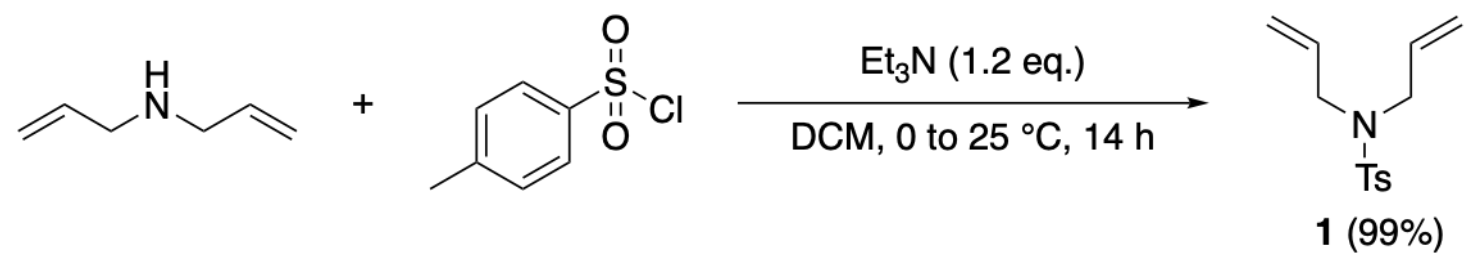

To a solution of diallylamine (1.17 g, 1.48 mL, 12.0 mmol, 1.2 eq.) dissolved in DCM (20 mL), triethylamine (1.21 g, 1.69 mL, 12.0 mmol, 1.2 eq.) was added dropwise at 0 °C. After 30 minutes, *p*-toluenesulfonyl chloride (1.91 g, 10.0 mmol, 1.0 eq.) was added slowly to the reaction mixture at 0 °C. The reaction mixture was stirred at 25 °C for 14 hours. It was then washed with water and brine, dried over Na_2SO_4, and the solvent was removed *in vacuo*. The crude residue was purified by column chromatography (SiO_2, hex/EA, 4:1) to afford *N,N*-diallyl-4-methylbenzenesulfonamide (**1**, 1.50 g, 99%) as a colorless liquid.

^{1}H NMR (500 MHz, $CDCl_3$) δ 7.70 (d, *J* = 8.3 Hz, 2H), 7.29 (d, *J* = 7.9 Hz, 2H), 5.61 (ddt, *J* = 17.5, 9.9, 6.4 Hz, 2H), 5.28–5.01 (m, 4H), 3.80 (dt, *J* = 6.2, 1.3 Hz, 4H), 2.43 (s, 3H). ^{13}C NMR (126 MHz, $CDCl_3$) δ 143.35, 137.59, 132.82, 129.81, 127.33, 119.10, 49.46, 21.65. The ^{1}H and ^{13}C NMR data were in agreement with the reported values.

UHPLC: T_R = 5.44 min.

Synthesis of 1-tosyl-2,5-dihydro-1*H*-pyrrole (2)

Prepared according to the literature.[79]

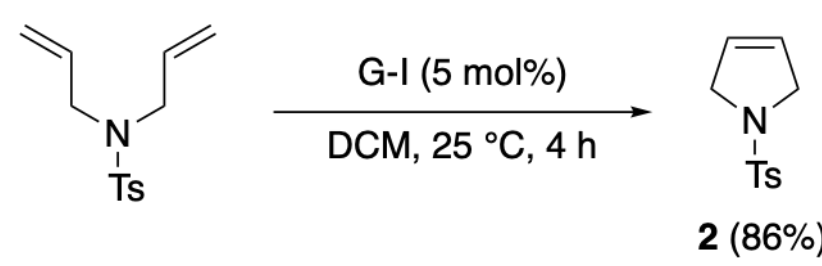

To a solution of **G-I** (41.2 mg, 0.05 mmol, 5 mol%) in anhydrous DCM (5.0 mL), *N,N*-diallyl-4-methylbenzenesulfonamide (**1**, 251 mg, 1.00 mmol, 1.0 eq.) was added. The reaction mixture was stirred at 25 °C for 4 hours, and then the solvent was removed *in vacuo*. The crude product was purified by column chromatography (SiO_2, PE/EA, 8:1 to 4:1) to afford 1-tosyl-2,5-dihydro-1*H*-pyrrole (**2**, 191 mg, 86%) as a white solid.

^{1}H NMR (500 MHz, $CDCl_3$) δ 7.72 (d, *J* = 8.3 Hz, 2H), 7.32 (d, *J* = 7.9 Hz, 2H), 5.65 (s, 2H), 4.12 (s, 4H), 2.42 (s, 3H). ^{13}C NMR (126 MHz, $CDCl_3$) δ 143.56, 134.46, 129.90, 127.56, 125.59, 54.98. 0.14. The ^{1}H and ^{13}C NMR data were in agreement with the reported values.

UHPLC: T_R = 4.53 min.

Catalytic experiments in aqueous solution

General procedure

All catalytic reactions were performed in triplicate. A 1.5 mL GC glass vial was charged with *N,N*-diallyl-4-methylbenzenesulfonamide (**1**, 10 µL of a 100 mM stock solution in DMSO), followed by the corresponding buffer (180 µL) and the corresponding **[Ru]** complex (10 µL of a 1 mM or 100 µM stock solution in DMSO). The vial was then placed in a Thermoshaker at 37 °C for 48 hours at 600 rpm. The reaction was quenched by the addition of 2-phenylethanol (200 µL of a 5 mM solution in MeOH) and analyzed by UHPLC (UV detection at 254 nm), Figure S6.

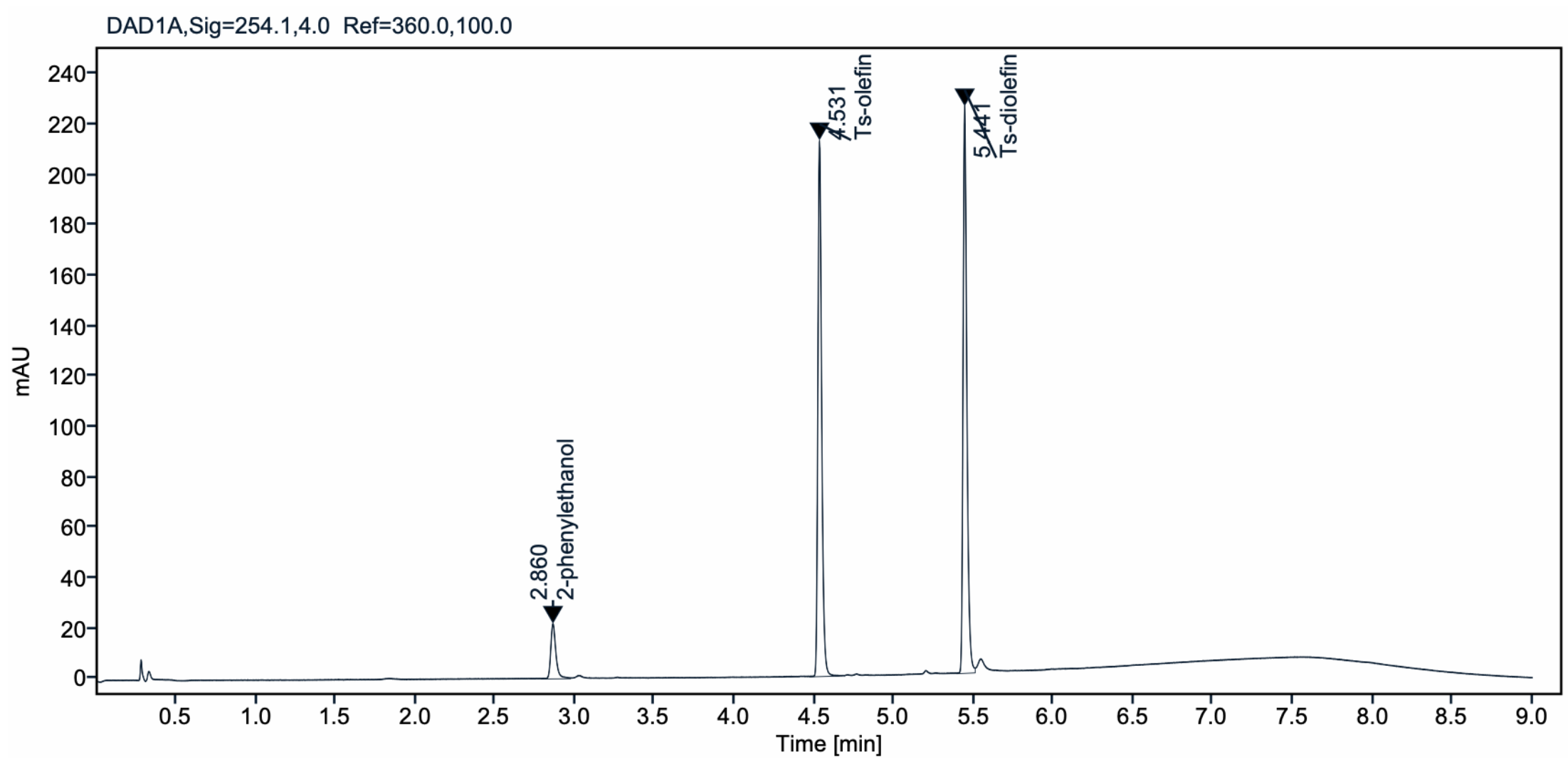

Figure S6. UHPLC chromatogram monitored at 254 nm of *N,N*-diallyl-4-methylbenzenesulfonamide (**1**) and 1-tosyl-2,5-dihydro-1*H*-pyrrole (**2**), along with 2-phenylethanol as internal standard using the previously described method.

Temperature dependency

Following the general procedure, the reaction vials were placed in a Thermoshaker at 37, 50, or 60 °C for 48 hours. The reaction was quenched by the addition of 2-phenylethanol (200 µL of a 5 mM solution in MeOH) and analyzed by UHPLC (UV detection at 254 nm).

Time-course monitoring

Following the general procedure, the reaction vials were placed in a Thermoshaker at 37 °C. The reaction was quenched after 1, 2, 4, 7, 24, 48, and 72 hours by the addition of 2-phenylethanol (200 µL of a 5 mM solution in MeOH) and analyzed by UHPLC (UV detection at 254 nm).

Procedure for catalysis with Ru(^{i}Pr$_2$CAAC-5) sulfonamide · hCA II

All catalytic reactions were performed in triplicate. A 1.5 mL GC glass vial was charged with the corresponding **[Ru]** complex (10 µL of a 1 mM or 100 µM stock solution in DMSO), followed by hCA **II** (180 µL of a 55.6 µM or 5.56 µM stock solution in the corresponding buffer). The ArM was assembled by incubation at 37 °C for 20 min. Then, *N,N*-diallyl-4-methylbenzenesulfonamide (**1**, 10 µL of a 100 mM stock

solution in DMSO) was added, and the vial was placed in a Thermoshaker at 37 °C for 48 hours at 600 rpm. The reaction was quenched by the addition of 2-phenylethanol (200 µL of a 5 mM solution in MeOH) and analyzed by UHPLC (UV detection at 254 nm).

Calibration curves

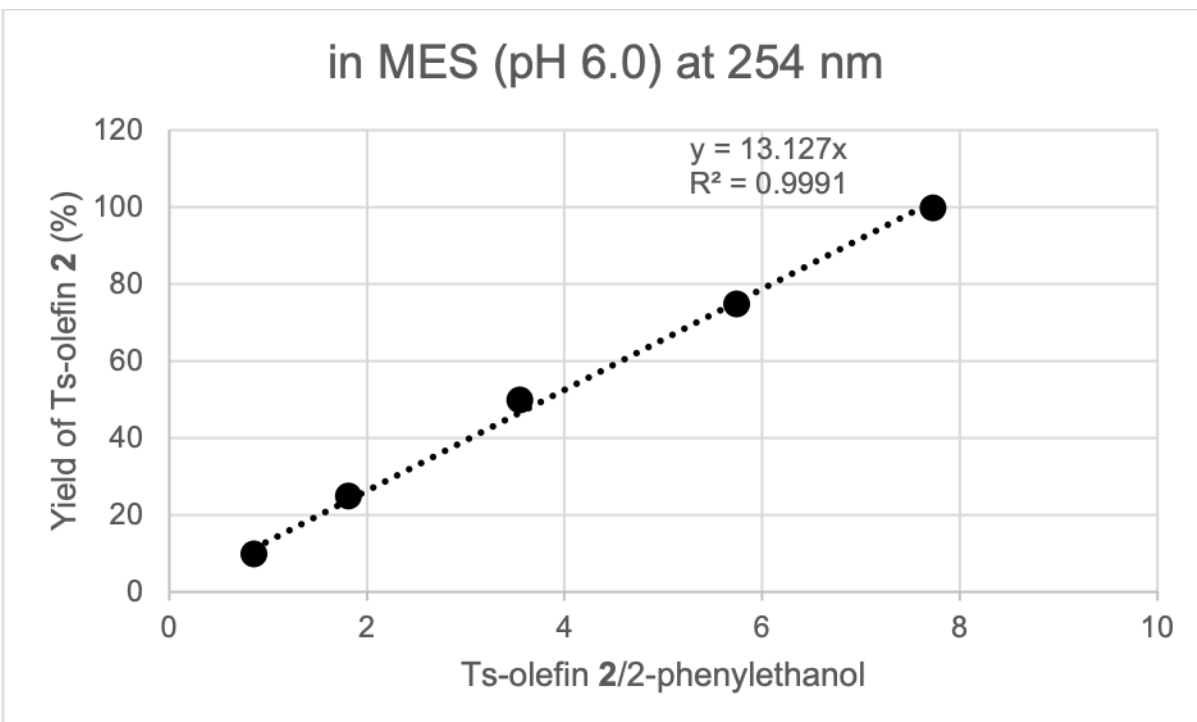

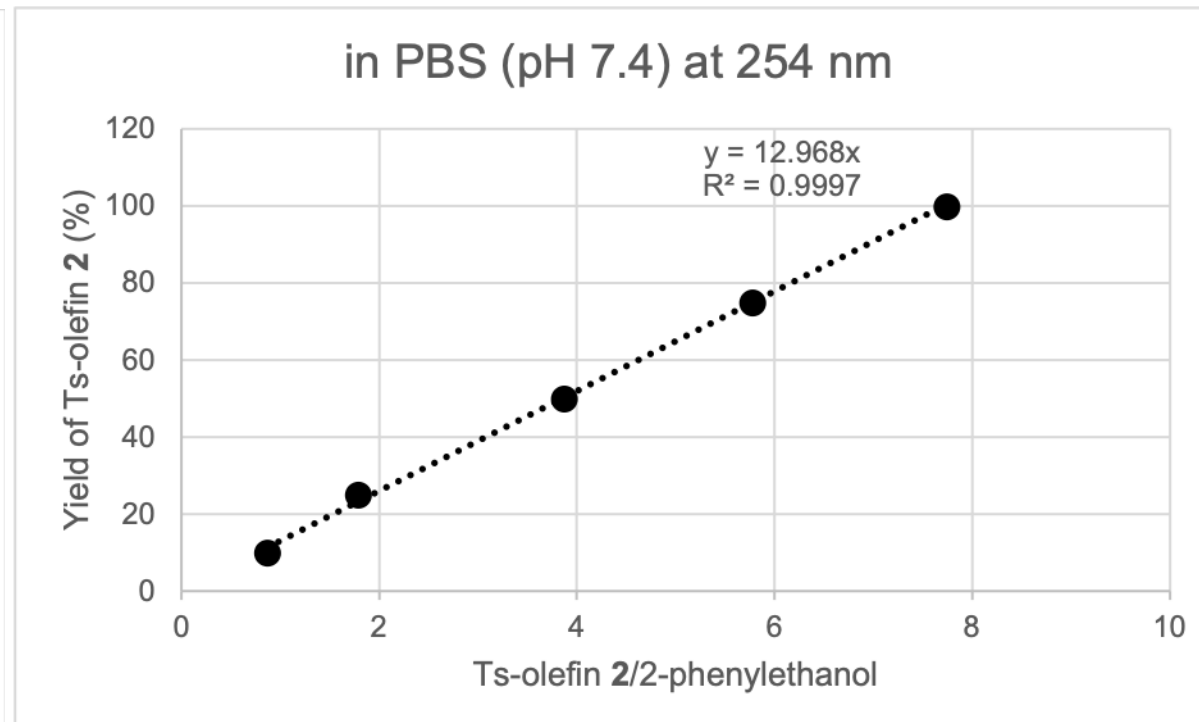

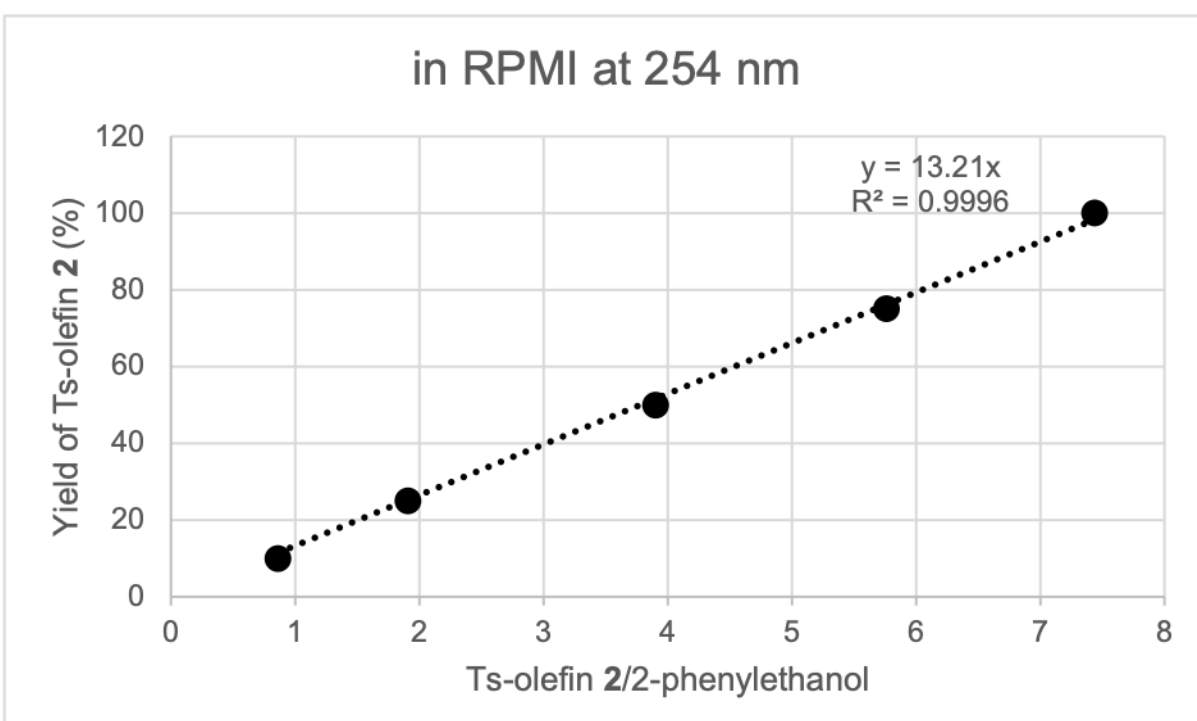

Catalytic experiments in organic solution

Procedure for catalysis in PhMe-d$_8$ at different temperatures

A GC vial was charged with tosyl diolefin **1** (150 µL of a 20 mM stock solution in PhMe-d$_8$), followed by trimethoxybenzene (150 µL of a 20 mM stock solution in PhMe-d$_8$) and 0.1 or 1.0 mol% of ruthenium catalyst (30 or 300 µL of a 100 µM stock solution in PhMe-d$_8$). The amount of solvent was adjusted to 600 µL for all reactions by addition of more PhMe-d$_8$. The GC vials were incubated at 37 or 50 °C for 48 hours and then analyzed by ^{1}H NMR spectroscopy. The proton signals of the starting material tosyl diolefin **1** (3.66 ppm, 4H) and product tosyl olefin **2** (3.84 ppm, 4H) were normalized using the signal of trimethoxybenzene (3.33 ppm, 9H), and the TON was calculated, Figure S7.

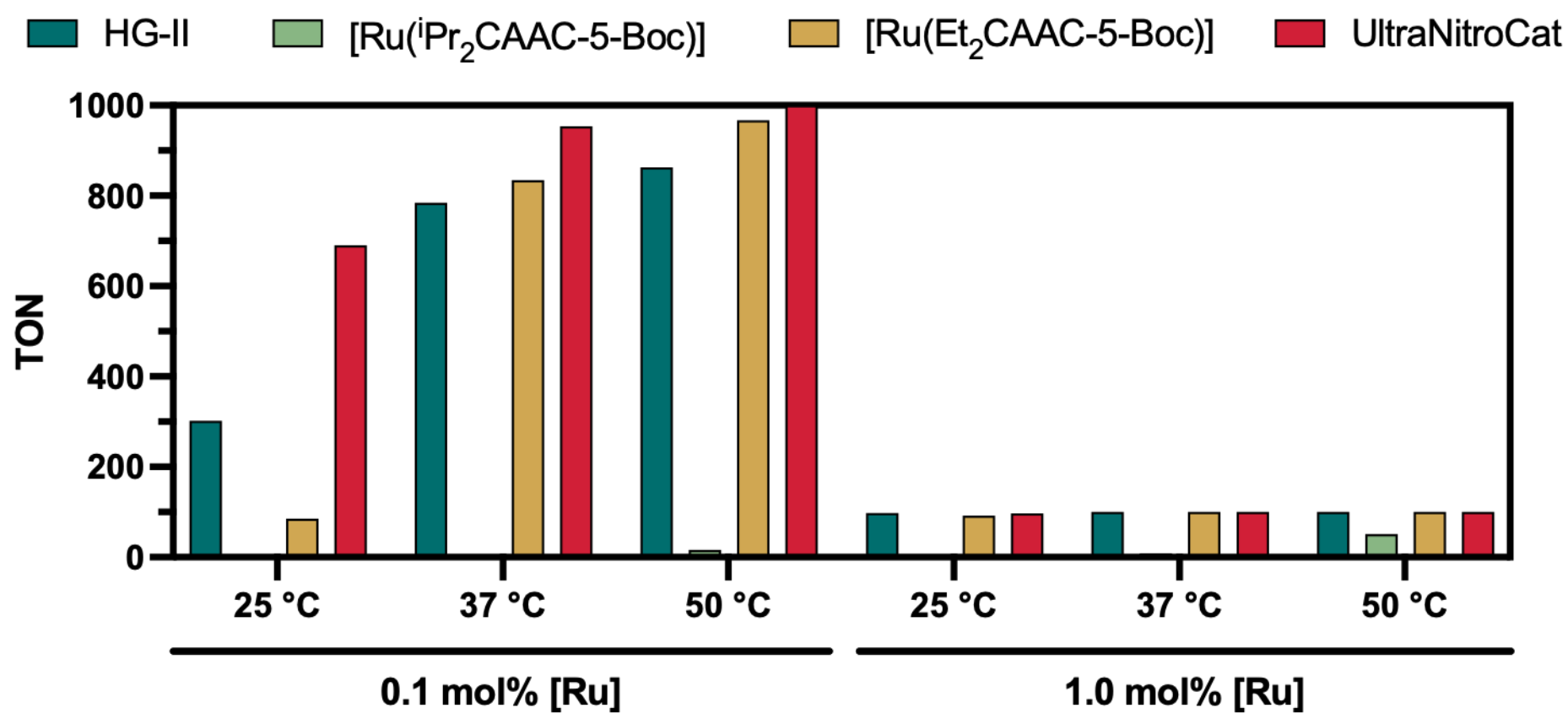

Figure S7. RCM of tosyl diolefin **1** (5 mM) in the presence of ruthenium catalyst (0.1 or 1.0 mol%) in toluene at different temperatures (25, 37, 50 °C) for 48 hours. Yields were determined by ^{1}H NMR spectroscopy (3.33 ppm – trimethoxybenzene, 3.66 ppm – tosyl diolefin **1**, and 3.84 ppm – tosyl olefin **2**) using trimethoxybenzene as internal standard.

Time-course monitoring in DCM-d$_2$ or PhMe-d$_8$

An NMR tube was charged with tosyl diolefin **1** (150 µL of a 20 mM stock solution in deuterated solvent), followed by trimethoxybenzene (150 µL of a 20 mM stock solution in deuterated solvent) and ruthenium catalyst (30 or 300 µL of a 100 µM stock solution in deuterated solvent). The amount of solvent was adjusted to 600 µL for all reactions by addition of more deuterated solvent. The reaction progress was then monitored by ^{1}H NMR spectroscopy at 25 °C for 72 hours. The proton signals of the starting material tosyl diolefin **1** (3.66 ppm, 4H) and product tosyl olefin **2** (3.84 ppm, 4H) were normalized using the signal of trimethoxybenzene (3.33 ppm, 9H), and the TON was calculated, Figure S8–S9.

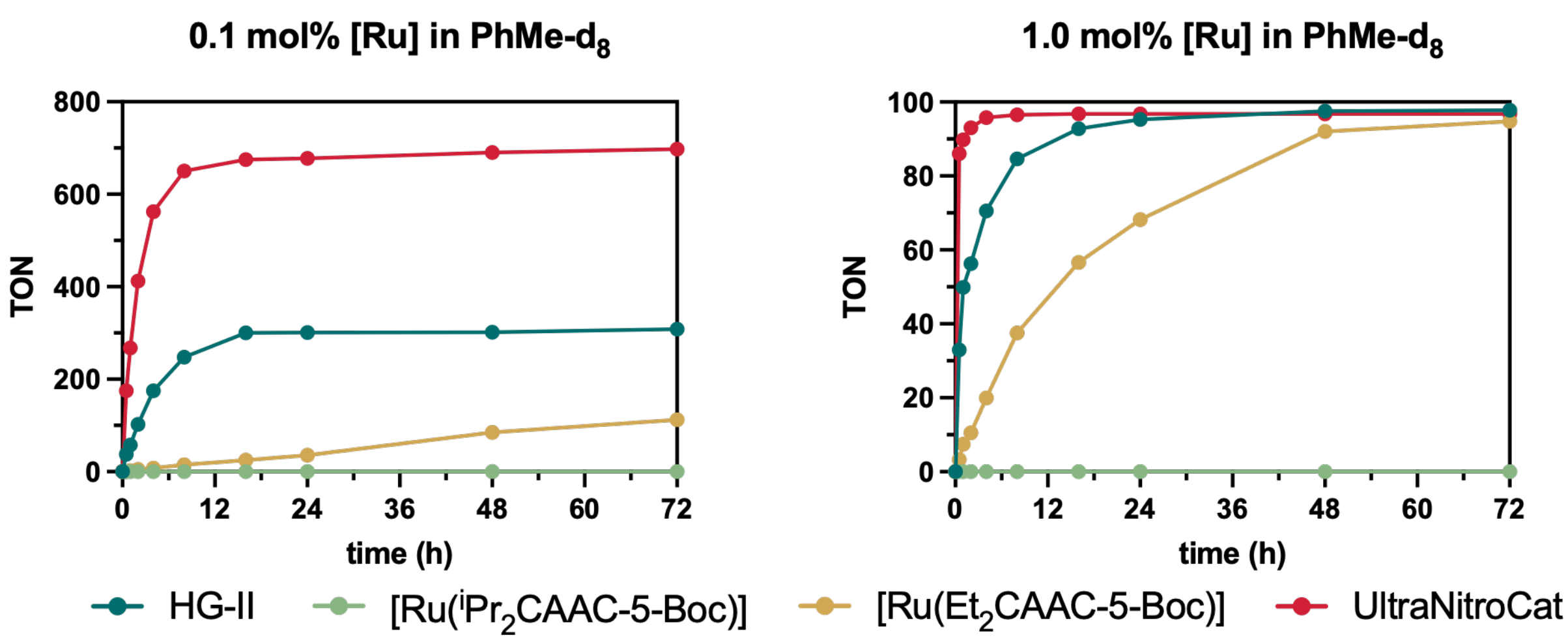

Figure S8. Time-course monitoring of the RCM reaction of tosyl diolefin **1** (5 mM) in the presence of ruthenium catalyst (0.1 mol% or 1.0 mol%) in PhMe-d$_8$ at 25 °C. Yields were determined by ^{1}H NMR spectroscopy (3.33 ppm – trimethoxybenzene, 3.66 ppm – tosyl diolefin **1**, and 3.84 ppm – tosyl olefin **2**) using trimethoxybenzene as internal standard.

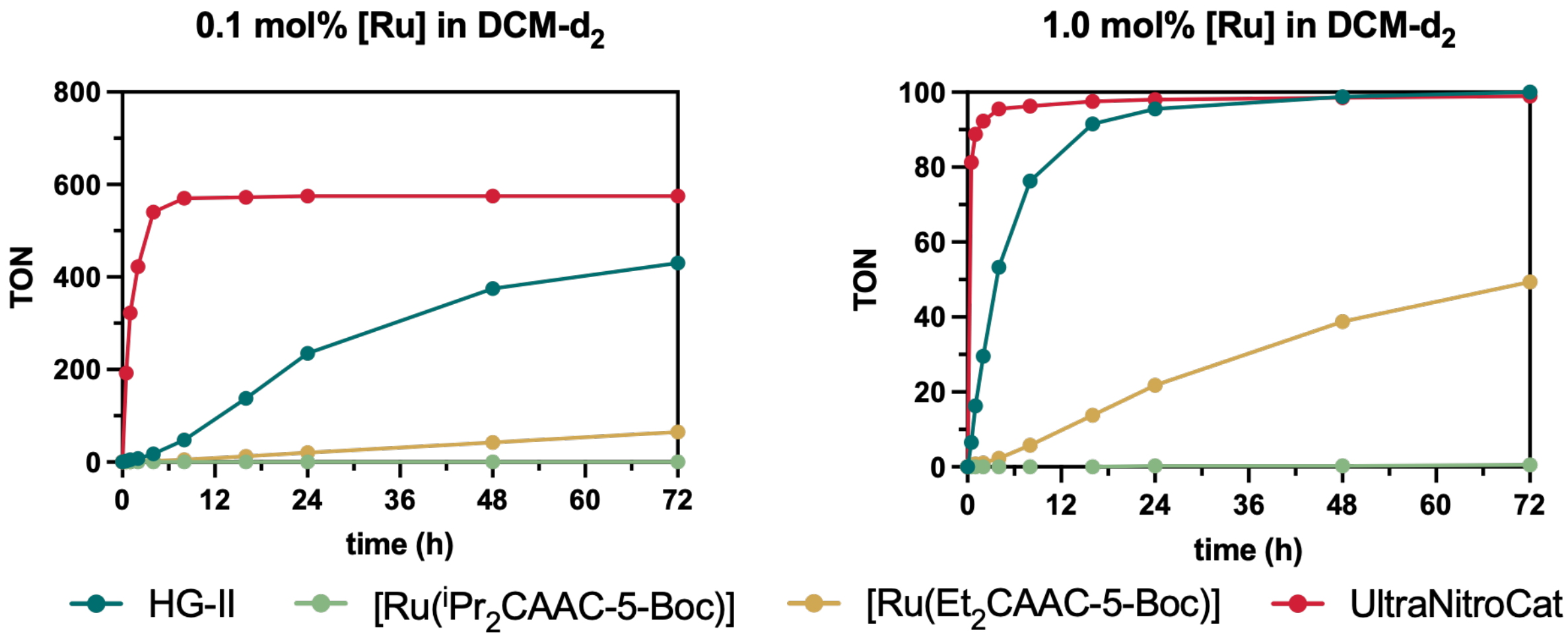

Figure S9. Time-course monitoring of the RCM reaction of tosyl diolefin **1** (5 mM) in the presence of ruthenium catalyst (0.1 mol% or 1.0 mol%) in DCM-d_2 at 25 °C. Yields were determined by ^{1}H NMR spectroscopy (3.33 ppm – trimethoxybenzene, 3.66 ppm – tosyl diolefin **1**, and 3.84 ppm – tosyl olefin **2**) using trimethoxybenzene as internal standard.

Solubility assessment of [Ru(CAAC-5-Boc)] complexes

Beer–Lambert linearity

A stock solution of the ruthenium complex (1.0 mM) was prepared in DMSO. An aliquot of this stock was diluted into the corresponding buffer containing 50 mM $MgCl_2$ to a final volume of 2'000 µL, affording a 50 µM solution of the complex in 10% DMSO (v/v). This solution was serially diluted with the same buffer to yield final concentrations of 50, 25, 12.5, and 6.25 µM, while keeping the DMSO concentration at 10% (v/v) throughout the dilution series. UV-Vis absorption spectra (300–500 nm) of each solution were recorded at 25 °C. The wavelength of maximum absorbance in the 300–500 nm region was determined from the spectrum of the highest-concentration sample (50 µM) and fixed for all subsequent concentrations (380 nm; Figures S10–S11). The absorbance at 380 nm was plotted against the complex concentration and fit by linear regression, Figures S10–S11. A linear correlation between absorbance and concentration across the tested range (R^2 > 0.98) was taken as evidence that there is no solubility difference between the two complexes.

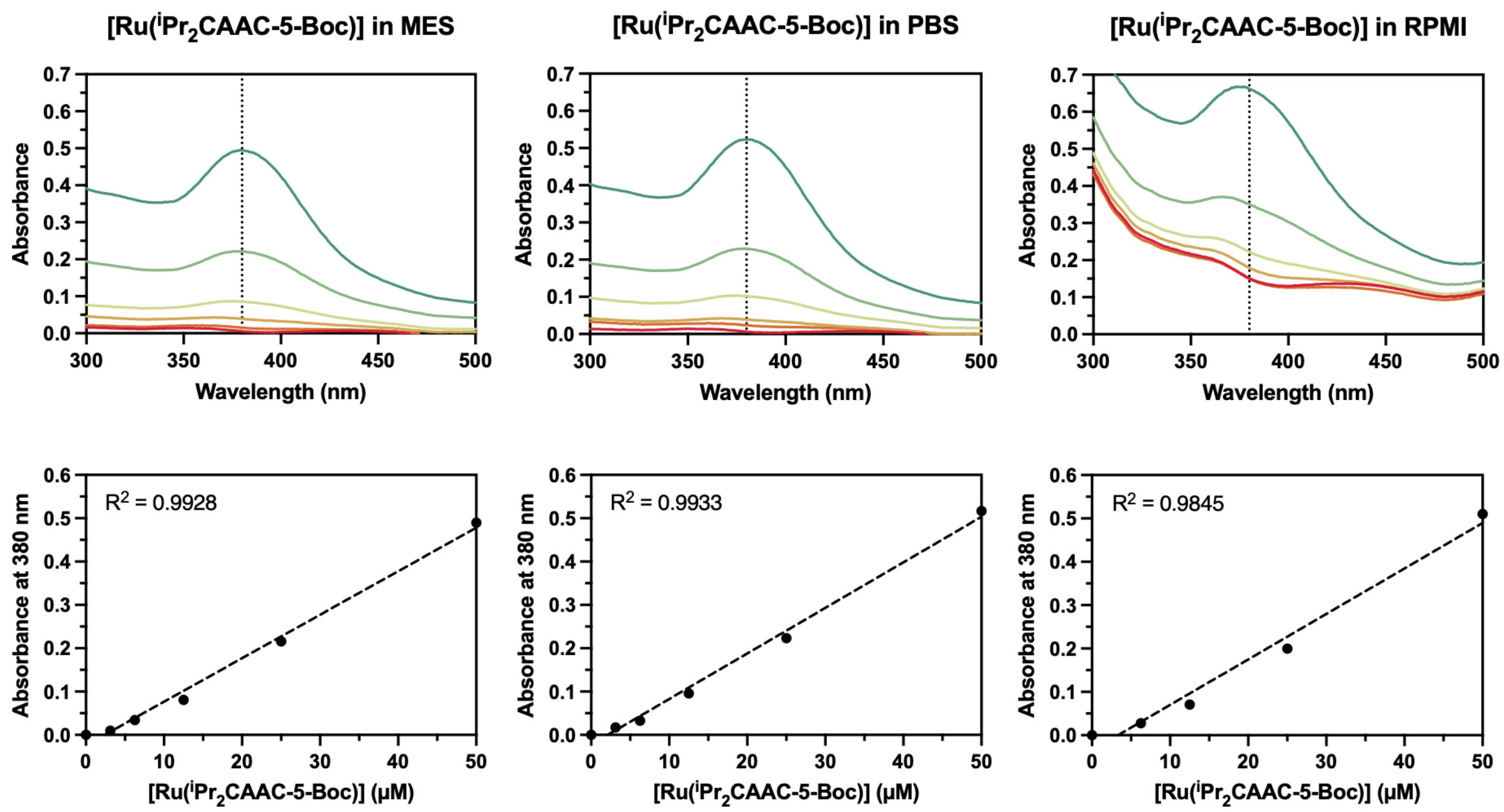

Figure S10. Top: UV-Vis absorption spectra (300–500 nm) of **[Ru(^{i}Pr$_2$CAAC-5-Boc)]** recorded at 3.125, 6.25, 12.5, 25, and 50 µM in MES, PBS, and RPMI buffers (containing 10% DMSO). The dashed vertical line marks the wavelength (380 nm) used for the Beer–Lambert linearity analysis. Bottom: Beer–Lambert linearity plot of **[Ru(^{i}Pr$_2$CAAC-5-Boc)]** at 380 nm across 3.125–50 µM in MES, PBS, and RPMI (blank-corrected absorbance). Dashed lines represent linear fit.

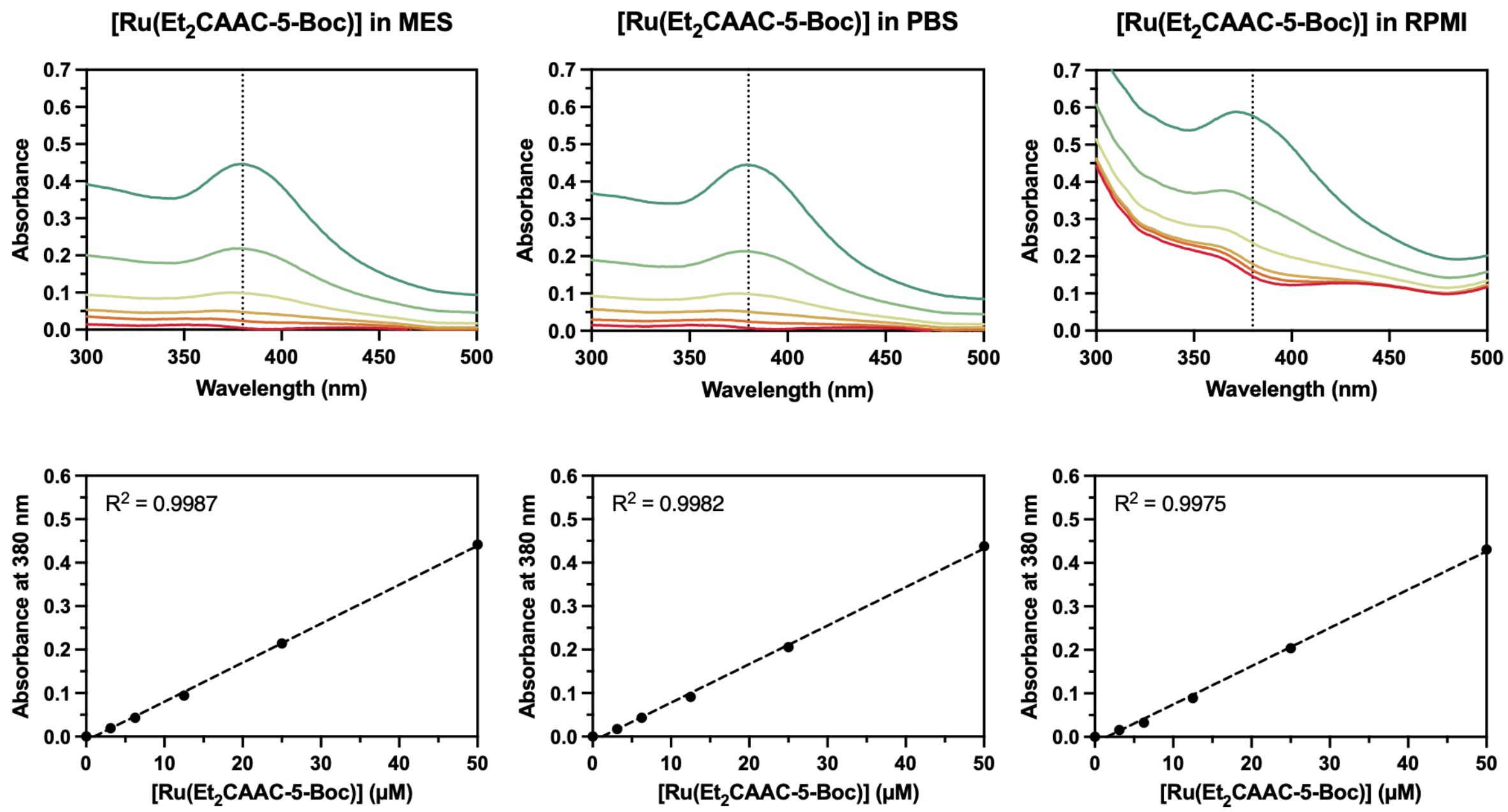

Figure S11. Top: UV-Vis absorption spectra (300–500 nm) of **[Ru(Et$_2$CAAC-5-Boc)]** recorded at 3.125, 6.25, 12.5, 25, and 50 µM in MES, PBS, and RPMI buffers (containing 10% DMSO). The dashed vertical line marks the wavelength (380 nm) used for the Beer–Lambert linearity analysis. Bottom: Beer–Lambert linearity plot of **[Ru(Et$_2$CAAC-5-Boc)]** at 380 nm across 3.125–50 µM in MES, PBS, and RPMI (blank-corrected absorbance). Dashed lines represent linear fit.

Solubility of [Ru(CAAC-5-Boc)] complexes over time

A stock solution of the ruthenium complex (1.0 mM) was prepared in DMSO. An aliquot of this stock was diluted into MES or PBS buffer containing 50 mM $MgCl_2$ to a final volume of 2’000 µL, affording a 25 µM solution of the complex in 10% DMSO (v/v). UV-Vis absorption at 380 nm of each solution was recorded at 37 °C for 8 hours, Figure S12. Following an initial plateau during the first hour, the absorbance decayed over time, consistent with precipitation of the complex. The decay (1–8 hours) was fit to a one-phase exponential decay model, giving half-lives of 0.88 h and 0.78 h for **[Ru(^{i}Pr$_2$CAAC-5-Boc)]** in MES (pH 6) and PBS (pH 7), respectively, and 0.66 h and 0.60 h for **[Ru(Et$_2$CAAC-5-Boc)]** in MES (pH 6) and PBS (pH 7), respectively (R^2 = 0.96–0.99). The similar magnitude of these half-lives across both complexes indicates that precatalyst precipitation occurs on a comparable timescale for the Et^2- and iPr^2-substituted complexes, in contrast to the markedly larger difference in catalytic lifetime observed between the two complexes in the presence of substrate, indicating that differential precatalyst solubility does not account for this difference, Figure S12.

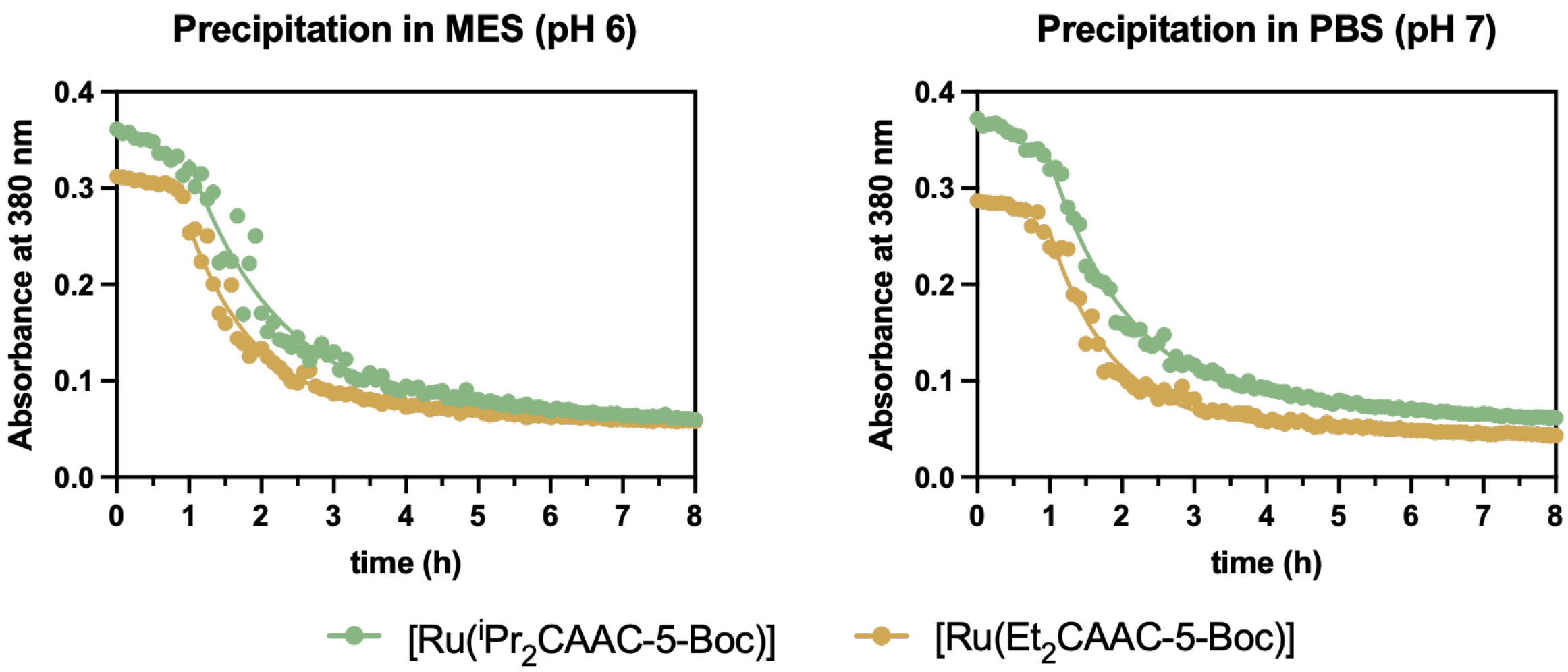

Figure S12. Time-dependent precipitation of [Ru(^{i}Pr$_2$CAAC-5-Boc)] and [Ru(Et$_2$CAAC-5-Boc)] in MES (pH 6, left) and PBS (pH 7, right) buffer. Absorbance at 380 nm was monitored at 37 °C over 8 hours for 25 µM solutions of each complex (10% DMSO v/v, 50 mM $MgCl_2$). Solid lines represent one-phase exponential decay fits to the data from 1–8 hours (excluding the initial plateau). Data points before t = 1 hour were excluded from the fit but are displayed for reference.

Stability of [Ru(CAAC-5-Boc)] complexes

Precatalyst stability

An NMR tube was charged with the ruthenium precatalyst **[Ru(^{i}Pr$_2$CAAC-5-Boc)]** (0.98 mg, 1.20 µmol) or **[Ru(Et$_2$CAAC-5-Boc)]** (0.94 mg, 1.2 µmol) dissolved in DMSO-d$_6$ (480 µL) and D_2O (120 µL). The decomposition of the precatalyst was monitored by ^{1}H NMR spectroscopy over time at 37 °C, Figure S49–S56.

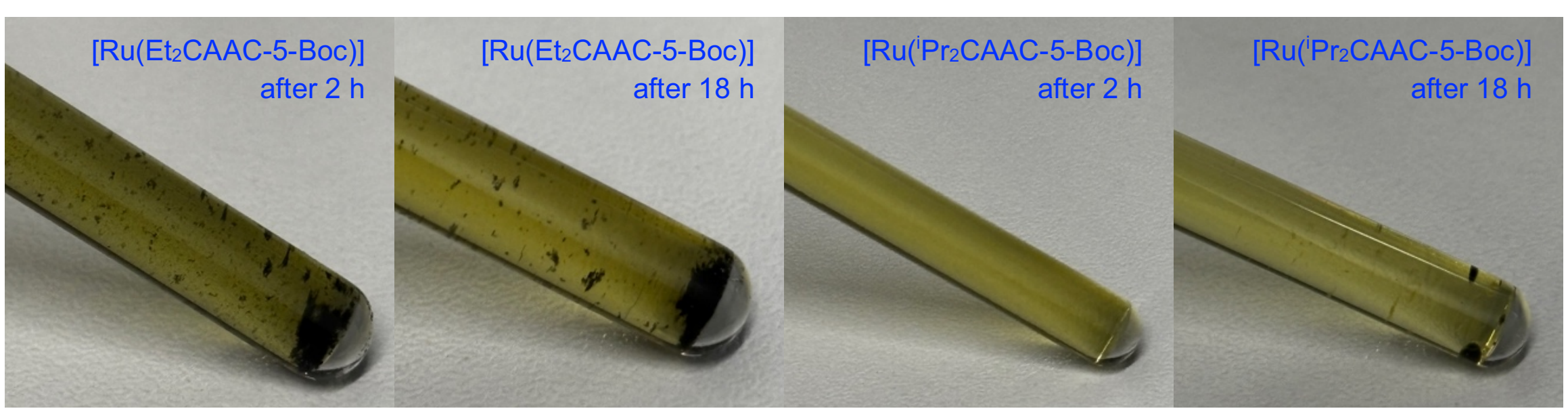

Figure S13. Photographs of NMR tubes containing **[Ru(Et$_2$CAAC-5-Boc)] and [Ru(^{i}Pr$_2$CAAC-5-Boc]** (1.2 µmol) in DMSO-d$_6$/D_2O (4:1, 600 µL total) after 2 hours and 18 hours at 37 °C. A visible precipitate forms over time for both complexes, consistent with precatalyst decomposition observed by ^{1}H NMR spectroscopy.

Time-resolved ^{1}H NMR monitoring of both precatalysts in DMSO-d$_6$/D_2O (4:1) at 37 °C revealed the concomitant loss of the Ru-benzylidene signal (16.30/16.33 ppm) and growth of a singlet at 10.31 ppm, identified as 2-isopropoxybenzaldehyde, consistent with hydrolytic cleavage of the benzylidene ligand as previously reported for Ru(NHC) complexes.[80,81] For **[Ru(Et$_2$CAAC-5-Boc)]**, an additional signal at 17.06 ppm, tentatively assigned to a chloride/aqua-exchanged intermediate, was observed alongside the

benzylidene from the earliest time points and decreased in relative intensity as the aldehyde signal grew, consistent with its role as a transient species along the decomposition pathway. No analogous intermediate was detected for **[Ru(ⁱPr₂CAAC-5-Boc)]**. Quantification of aldehyde formation over 52 hours indicated markedly slower decomposition for **[Ru(ⁱPr₂CAAC-5-Boc)]** (45% conversion at 52 hours) compared to **[Ru(Et₂CAAC-5-Boc)]** (74% conversion at 52 hours) at every time point measured, consistent with the enhanced catalytic lifetime of the ⁱPr₂-substituted complex under aqueous reaction conditions, Figure S14.

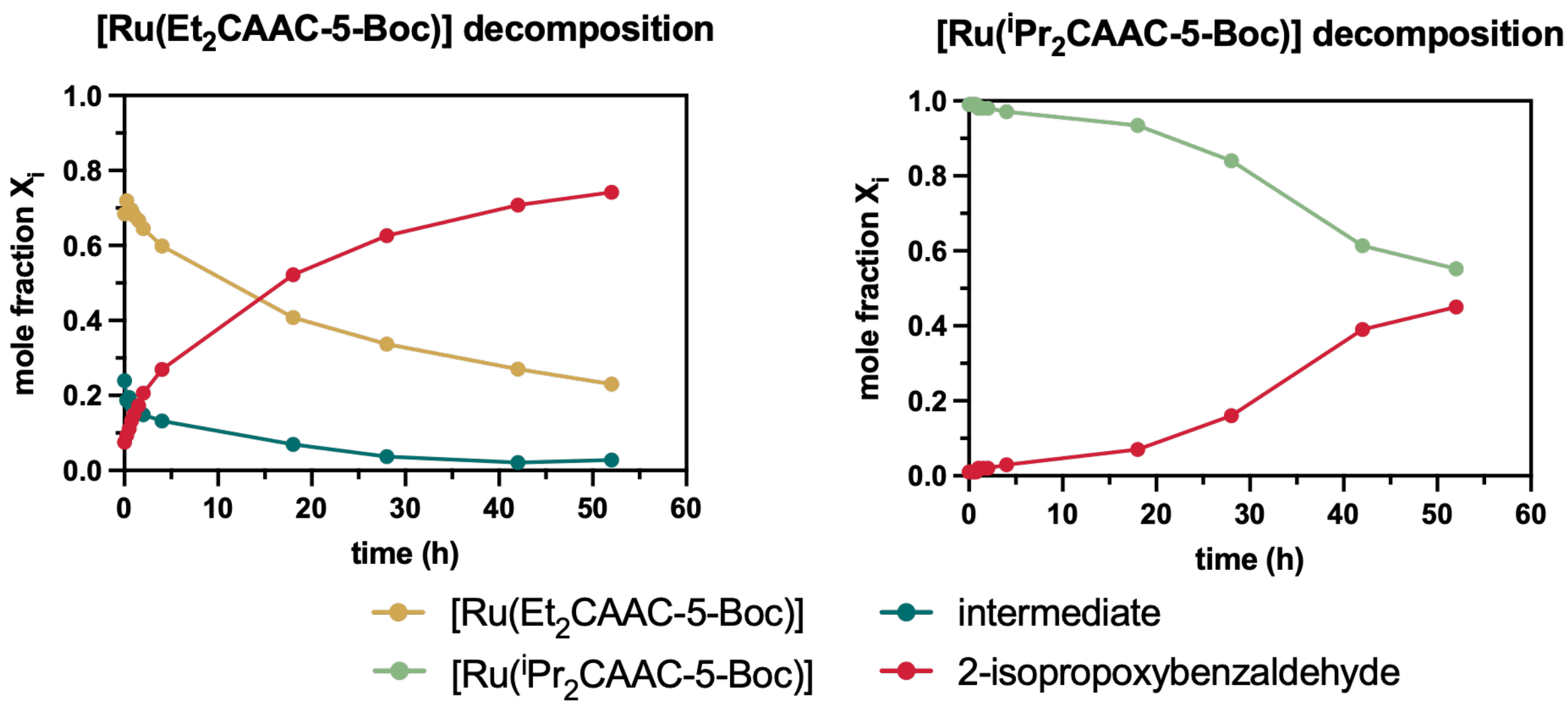

Figure S14. Time-dependent decomposition of **[Ru(Et₂CAAC-5-Boc)]** (left) and **[Ru(ⁱPr₂CAAC-5-Boc)]** (right) in DMSO-d6/D2O (4:1) at 37 °C, monitored by ^{1}H NMR spectroscopy. Mole fractions were determined by integration of the Ru-benzylidene (16.30/16.33 ppm), 2-isopropoxybenzaldehyde (10.31 ppm), and, for **[Ru(Et₂CAAC-5-Boc)]**, an additional intermediate species (17.06 ppm) signals.

Catalyst stability in the presence of substrate

An NMR tube was charged with the ruthenium precatalyst **[Ru(ⁱPr₂CAAC-5-Boc)]** (0.98 mg, 1.20 µmol) or **[Ru(Et₂CAAC-5-Boc)]** (0.94 mg, 1.2 µmol), and tosyl diolefin **1** (480 µL of a 12.5 mM stock solution in DMSO-d_6) and D_2O (120 µL). The decomposition of the precatalyst was monitored by ^{1}H NMR spectroscopy over time at 37 °C, Figure S57–S64.

In the presence of substrate, **[Ru(Et₂CAAC-5-Boc)]** decomposed markedly faster, reaching complete conversion to 2-isopropoxybenzaldehyde by 18 hours, compared to only 52% conversion at the same time point in the absence of substrate (74% after 52 hours), Figures S14 & S15. **[Ru(ⁱPr₂CAAC-5-Boc)]** was essentially unaffected by the presence of substrate over the timeframe measured (9% aldehyde at 28 hours, vs. 16% without substrate at the same time point). This selective, substrate-induced acceleration of decomposition in the Et₂-substituted complex, and its absence in the ⁱPr₂-substituted complex, indicates that the divergent catalytic lifetimes of the two complexes arise primarily from the differential stability of the reactive intermediate formed during turnover, rather than from differences in precatalyst stability.

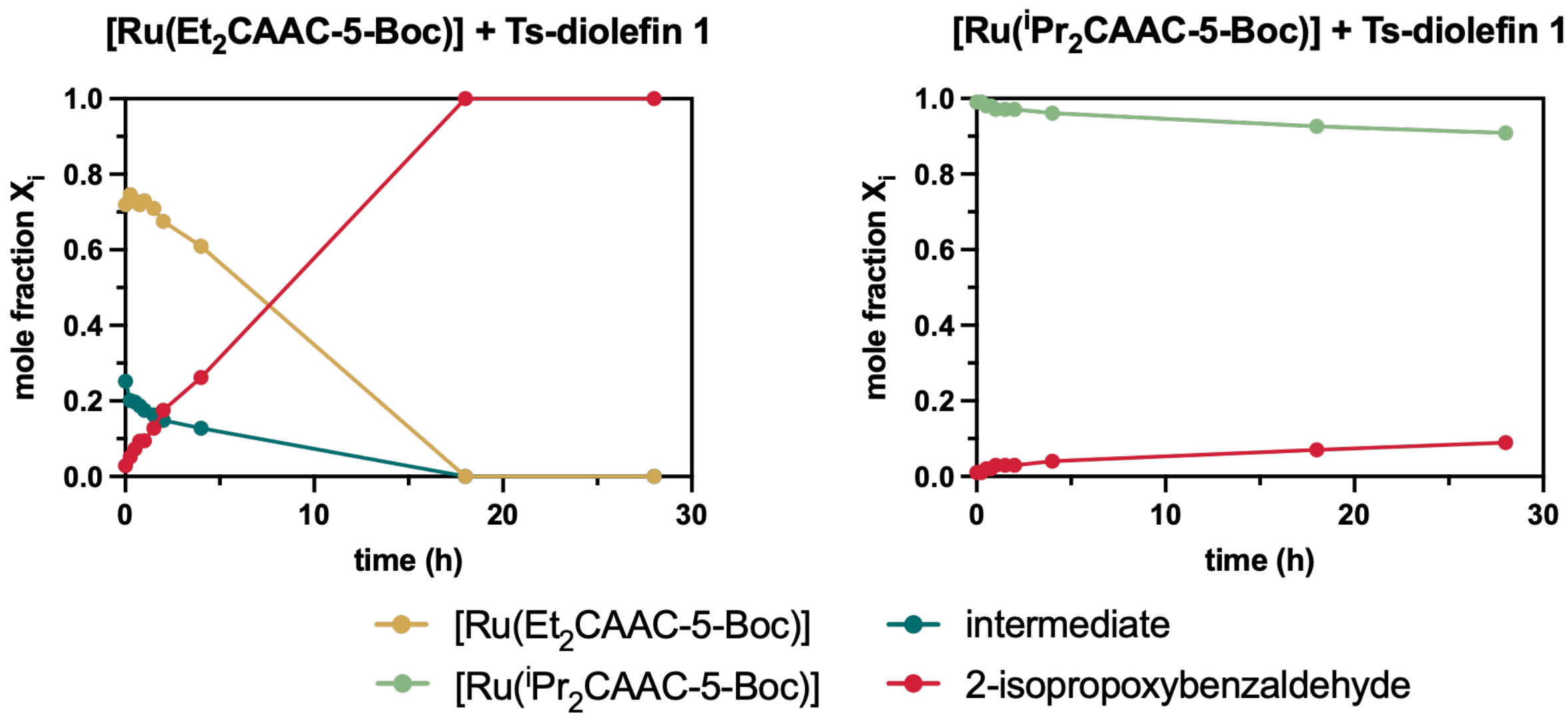

Figure S15. Time-dependent decomposition of **[Ru(Et2CAAC-5-Boc)]** (left) and **[Ru(iPr2CAAC-5-Boc)]** (right) in DMSO-d6/D2O (4:1) in the presence of Ts-diolefin **1** (10 eq.) at 37 °C, monitored by ^{1}H NMR spectroscopy. Mole fractions were determined by integration of the Ru-benzylidene (16.30/16.33 ppm), 2-isopropoxybenzaldehyde (10.31 ppm), and, for **[Ru(Et2CAAC-5-Boc)]**, an additional intermediate species (17.06 ppm) signals.

HRMS-ESI$^+$ analysis of decomposition samples

The NMR samples were analyzed by HRMS-ESI$^+$, Figures S79–S84. Ruthenium complexes were detected as [M–Cl]$^+$ ions, consistent with loss of chloride during ionization, as previously observed for these complexes.[80,81] The acylium ion of 2-isopropoxybenzaldehyde (m/z 163.0754, [M–H]$^+$) was detected in all four samples, corroborating its identification by ^{1}H NMR. **[Ru(Et2CAAC-5-Boc)]** (calcd. for [M–Cl]$^+$: m/z 747.2866) was not detected as a discrete chromatographic species in either the absence or presence of substrate, indicating complete decomposition of the precatalyst. In contrast, **[Ru(iPr2CAAC-5-Boc)]** (calcd. for [M–Cl]$^+$: m/z 775.3179) was clearly detected in both samples (t_R = 12.4 min), together with the corresponding [M+H]$^+$ ion (calcd. m/z 811.2941), confirming that a substantial fraction of the intact complex persists under these conditions. In the samples containing substrate, both tosyl diolefin **1** (m/z 252.1053, [M+H]$^+$) and the RCM product tosyl olefin **2** (m/z 224.074, [M+H]$^+$) were also detected.

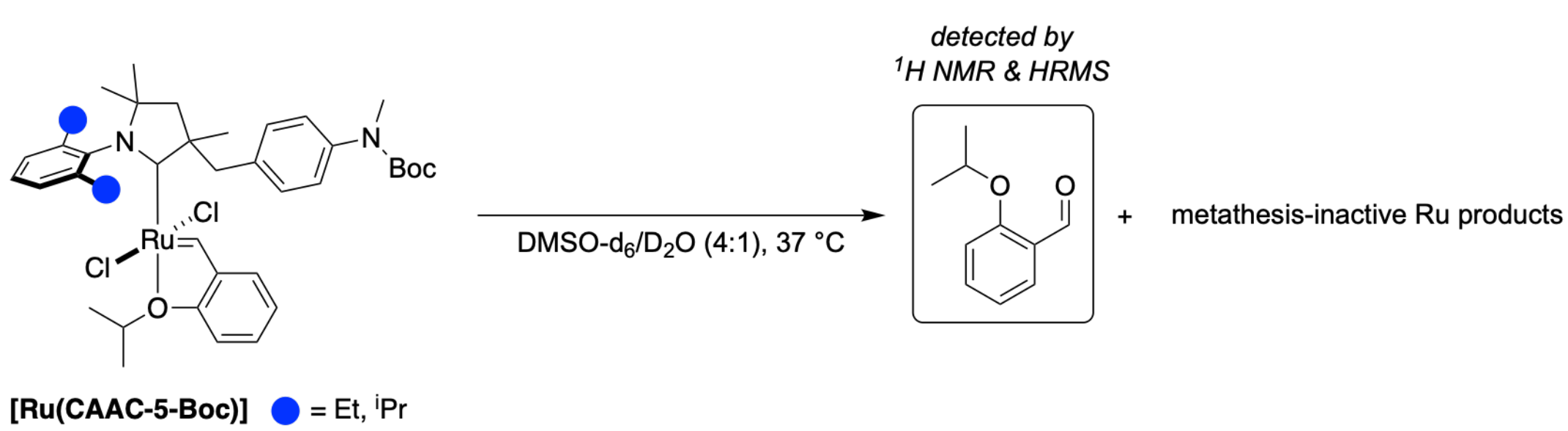

Figure S16. Hydrolytic cleavage of the ruthenium benzylidene affords 2-isopropoxybenzaldehyde and metathesis-inactive ruthenium products.

References

[65] H. Lavrard, F. Popowycz, "Harnessing Cascade Suzuki-Cyclization Reactions of Pyrazolo[3,4-b]pyridine for the Synthesis of Tetracyclic Fused Heteroaromatics" *Eur. J. Org. Chem.* **2017**, *2017*, 600–608.

[66] T. Ishida, S. Kikuchi, T. Tsubo, T. Yamada, "Silver-catalyzed incorporation of carbon dioxide into o-alkynylaniline derivatives" *Org. Lett.* **2013**, *15*, 848–851.

[67] R. Naito, M. Takeuchi, K. Morihira, M. Hayakawa, K. Ikeda, T. Shibanuma, Y. Isomura, "Selective muscarinic antagonists. I. Synthesis and antimuscarinic properties of 4-piperidyl benzhydrylcarbamate derivatives" *Chem. Pharm. Bull.* **1998**, *46*, 1274–1285.

[68] J. P. Stasiak, K. A. Woerpel, "Triethylamine-Catalyzed Cyclization of Unsaturated Hydroperoxides in the Presence of Triethylammonium Hydrochloride: A Synthesis of 1,2-Dioxanes" *Org. Lett.* **2025**, *27*, 2037–2041.

[69] J. Chu, D. Munz, R. Jazzar, M. Melaimi, G. Bertrand, "Synthesis of hemilabile cyclic (alkyl)(amino)carbenes (CAACs) and applications in organometallic chemistry" *J. Am. Chem. Soc.* **2016**, *138*, 7884–7887.

[70] J. F. Hower, R. W. Henkens, D. B. Chesnut, "Spin label investigation of the active site of an enzyme. Bovine carbonic anhydrase" *J. Am. Chem. Soc.* **1971**, *93*, 6665–6671.

[71] O. Galeote, S. C. D. Kennington, G. Benedito, L. Fraedrich, E. Davies-Howe, A. M. Costa, P. Romea, F. Urpí, G. Aullón, M. Font-Bardia, C. Puigjaner, "Direct, Stereodivergent, and Catalytic Michael Additions of Thioimides to α,β-Unsaturated Aldehydes – Total Synthesis of Tapentadol" *Angew. Chem. Int. Ed.* **2024**, *63*, e202319308.

[72] S. Specklin, J. Cossy, "Chemoselective synthesis of β-ketophosphonates using lithiated α-(trimethylsilyl)methylphosphonate" *J. Org. Chem.* **2015**, *80*, 3302–3308.

[73] S. C. Wang, D. B. Zamble, "Fluorescence analysis of sulfonamide binding to carbonic anhydrase" *Biochem. Mol. Biol. Educ.* **2006**, *34*, 364–368.

[74] M. Fani, L. Del Pozzo, K. Abiraj, R. Mansi, M. L. Tamma, R. Cescato, B. Waser, W. A. Weber, J. C. Reubi, H. R. Maecke, "PET of somatostatin receptor-positive tumors using64Cu- and 68Ga-somatostatin antagonists: The chelate makes the difference" *J. Nucl. Med.* **2011**, *52*, 1110–1118.

[75] J. Liu, Q. Chen, S. Rozovsky, "Utilizing Selenocysteine for Expressed Protein Ligation and Bioconjugations" *J. Am. Chem. Soc.* **2017**, *139*, 3430–3437.

[76] C. Mayer, D. G. Gillingham, T. R. Ward, D. Hilvert, "An artificial metalloenzyme for olefin metathesis" *Chem. Commun.* **2011**, *47*, 12068–12070.

[77] N. S. Kosower, E. M. Kosower, "Thiol Labeling with Bromobimanes" *Methods Enzymol.* **1987**, *143*, 76–84.

[78] C. M. So, S. Kume, T. Hayashi, "Rhodium-catalyzed asymmetric hydroarylation of 3-pyrrolines giving 3-arylpyrrolidines: Protonation as a key step" *J. Am. Chem. Soc.* **2013**, *135*, 10990–10993.

[79] P. Linnepe, A. M. Schmidt, P. Eilbracht, “2,3-Disubstituted indoles from olefins and hydrazines via tandem hydroformylation-Fischer indole synthesis and skeletal rearrangement” *Org. Biomol. Chem.* **2006**, *4*, 302–313.

[80] M. Kim, M. S. Eum, M. Y. Jin, K. W. Jun, C. W. Lee, K. A. Kuen, C. H. Kim, C. S. Chin, “Reactions of ruthenium benzylidenes with H2O to give benzaldehyde and (aqua)ruthenium complex” *J. Organomet. Chem.* **2004**, *689*, 3535–3540.

[81] C. O. Blanco, J. Sims, D. L. Nascimento, A. Y. Goudreault, S. N. Steinmann, C. Michel, D. E. Fogg, “The Impact of Water on Ru-Catalyzed Olefin Metathesis: Potent Deactivating Effects even at Low Water Concentrations” *ACS Catal.* **2021**, *11*, 893–899.

NMR Spectra

Figure S17. ^{1}H NMR spectrum of *tert*-butyl *p*-tolylcarbamate (**S1**) in $CDCl_3$.

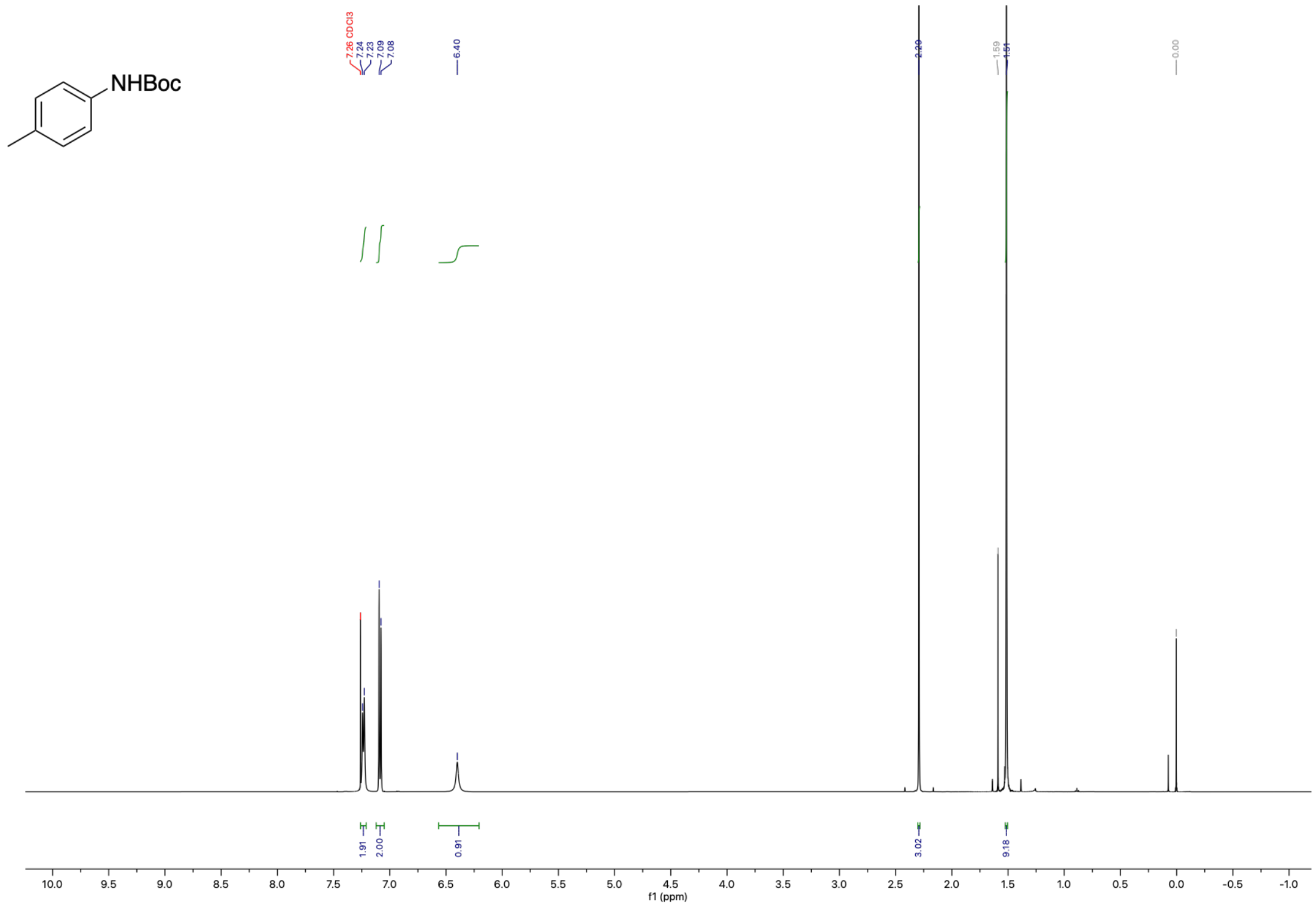

Figure S18. ^{1}H NMR spectrum of *tert*-butyl methyl(*p*-tolyl)carbamate (**S2**) in $CDCl_3$.

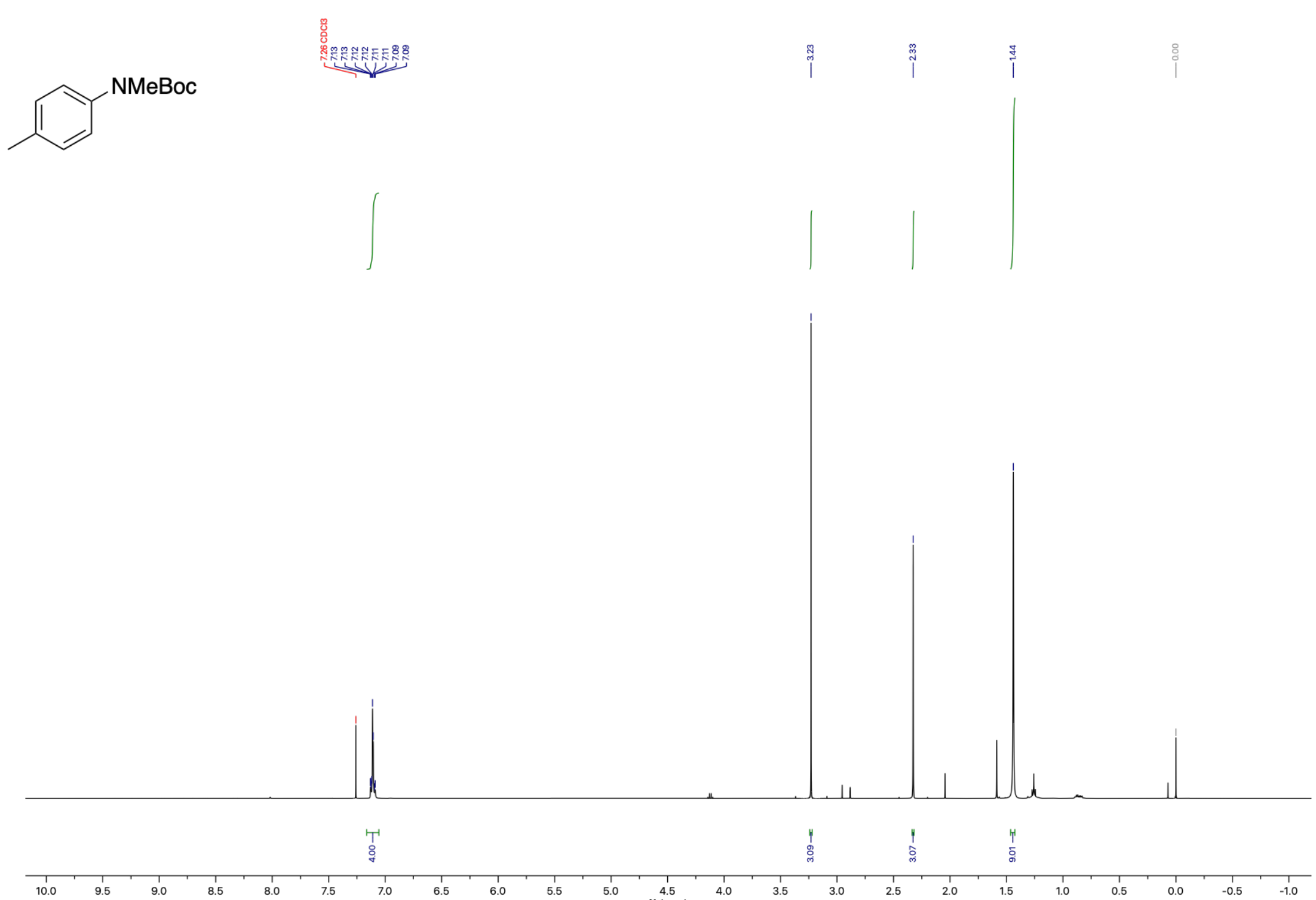

Figure S19. ^{1}H NMR spectrum of *tert*-butyl (4-(bromomethyl)phenyl)(methyl)carbamate (**S3**) in $CDCl_3$.

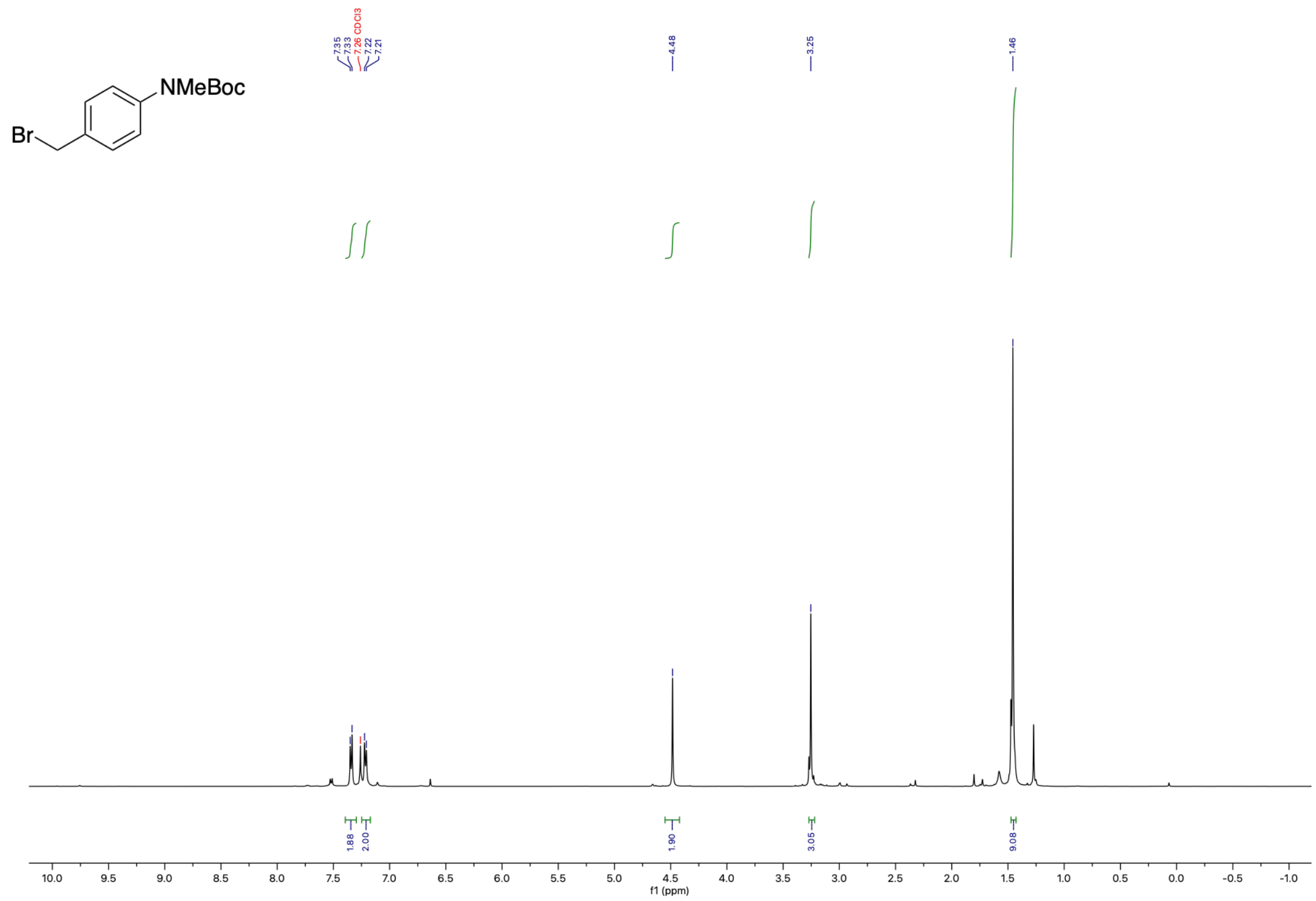

Figure S20. ^{1}H NMR spectrum of 2-methylallyl propionate (**S4**) in $CDCl_3$.

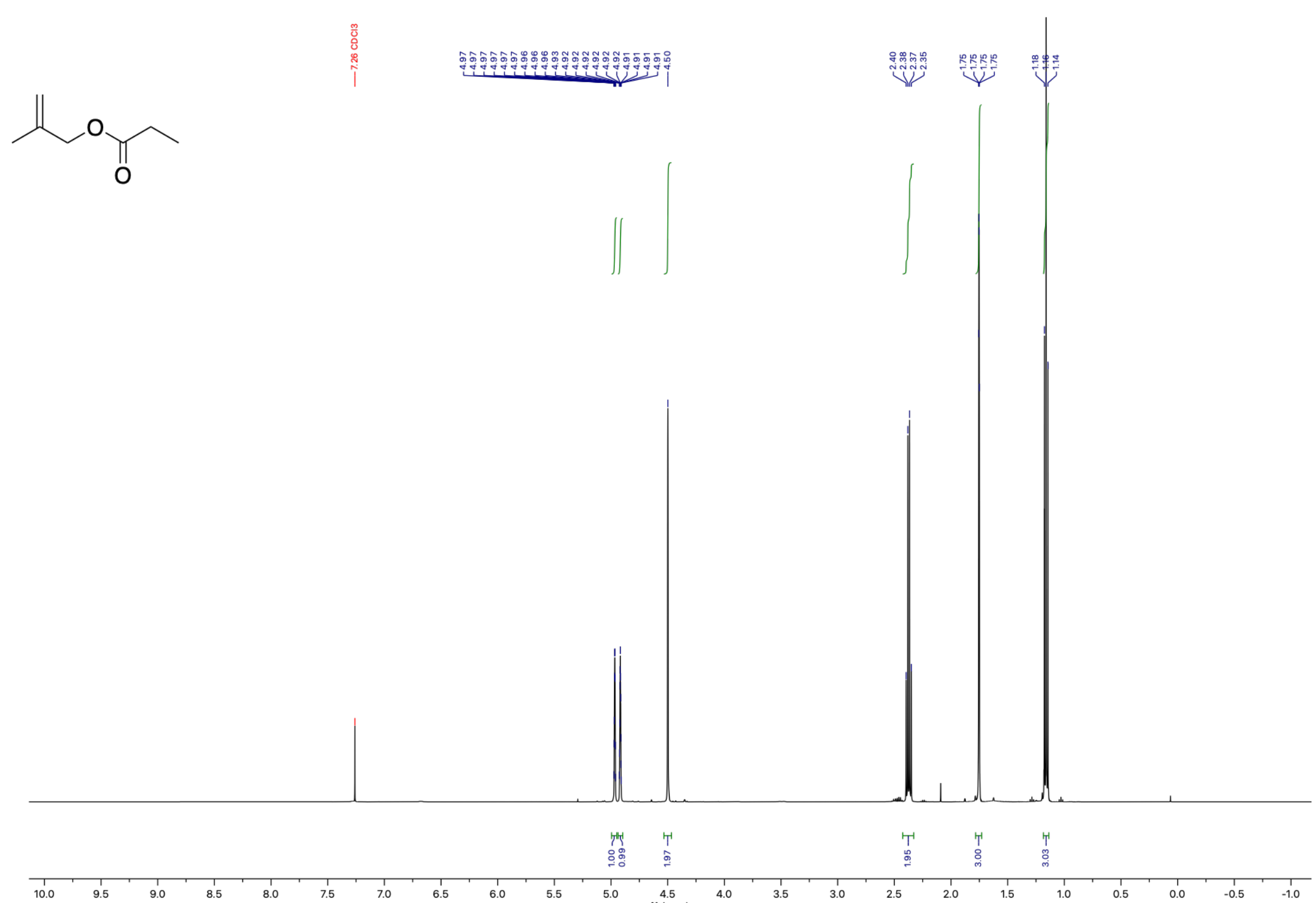

Figure S21. ^{1}H NMR spectrum of 2,4-dimethylpent-4-en-1-ol (**S5**) in $CDCl_3$.

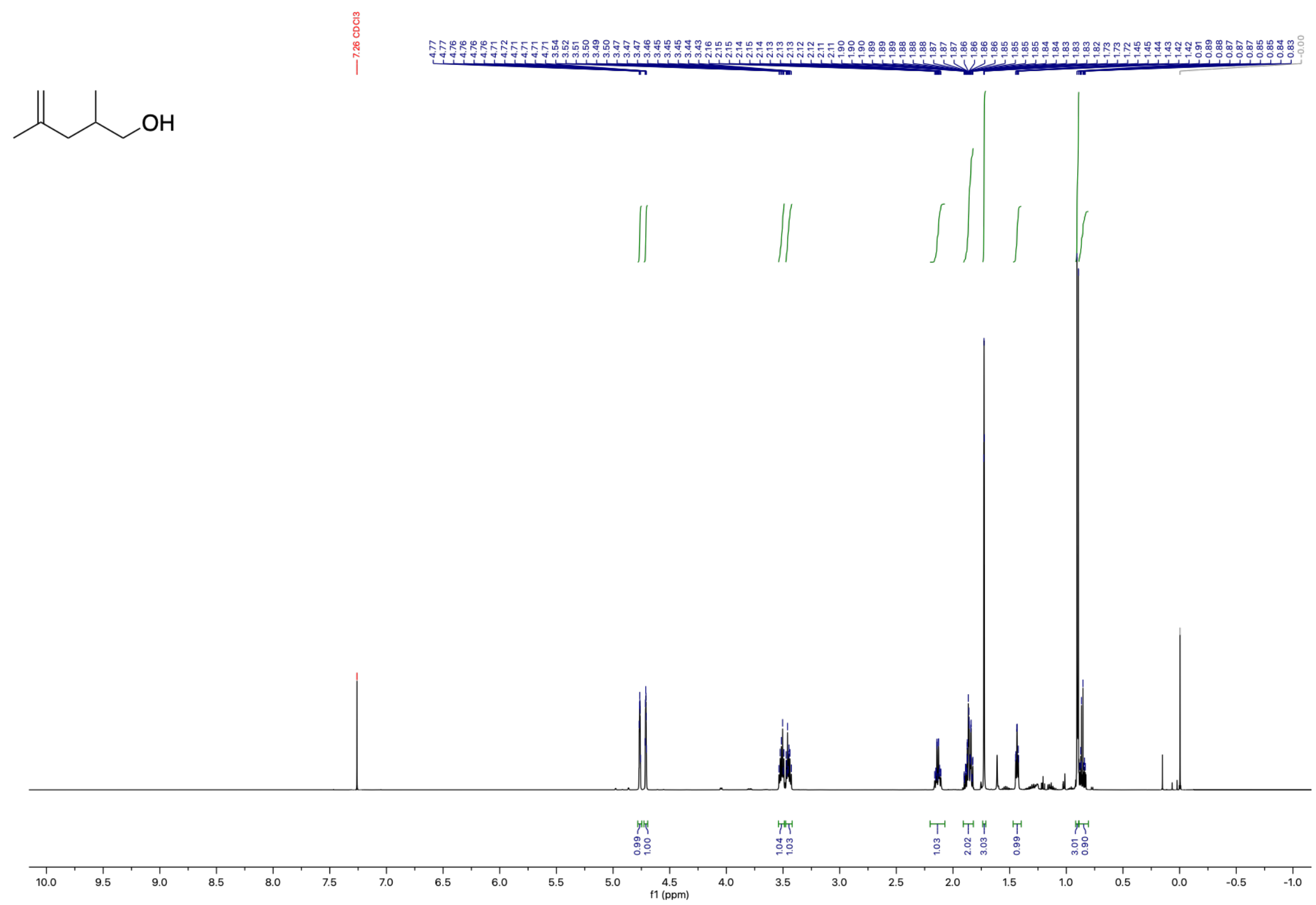

Figure S22. ^{1}H NMR spectrum of 2,4-dimethylpent-4-enal (**S6**) in CD_2Cl_2.

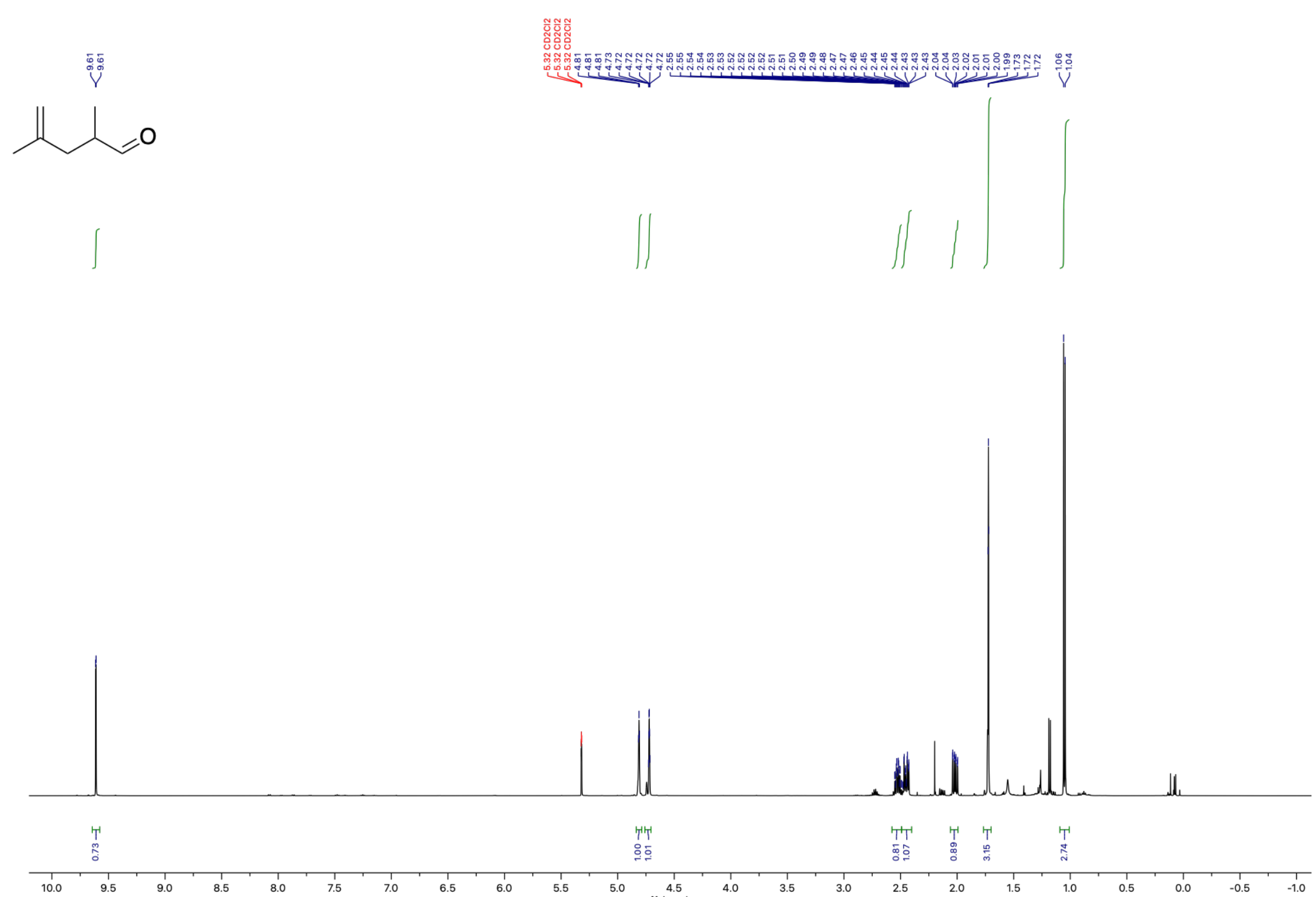

Figure S23. ^{1}H NMR spectrum of alkenyl aldimine **S7** in $CDCl_3$.

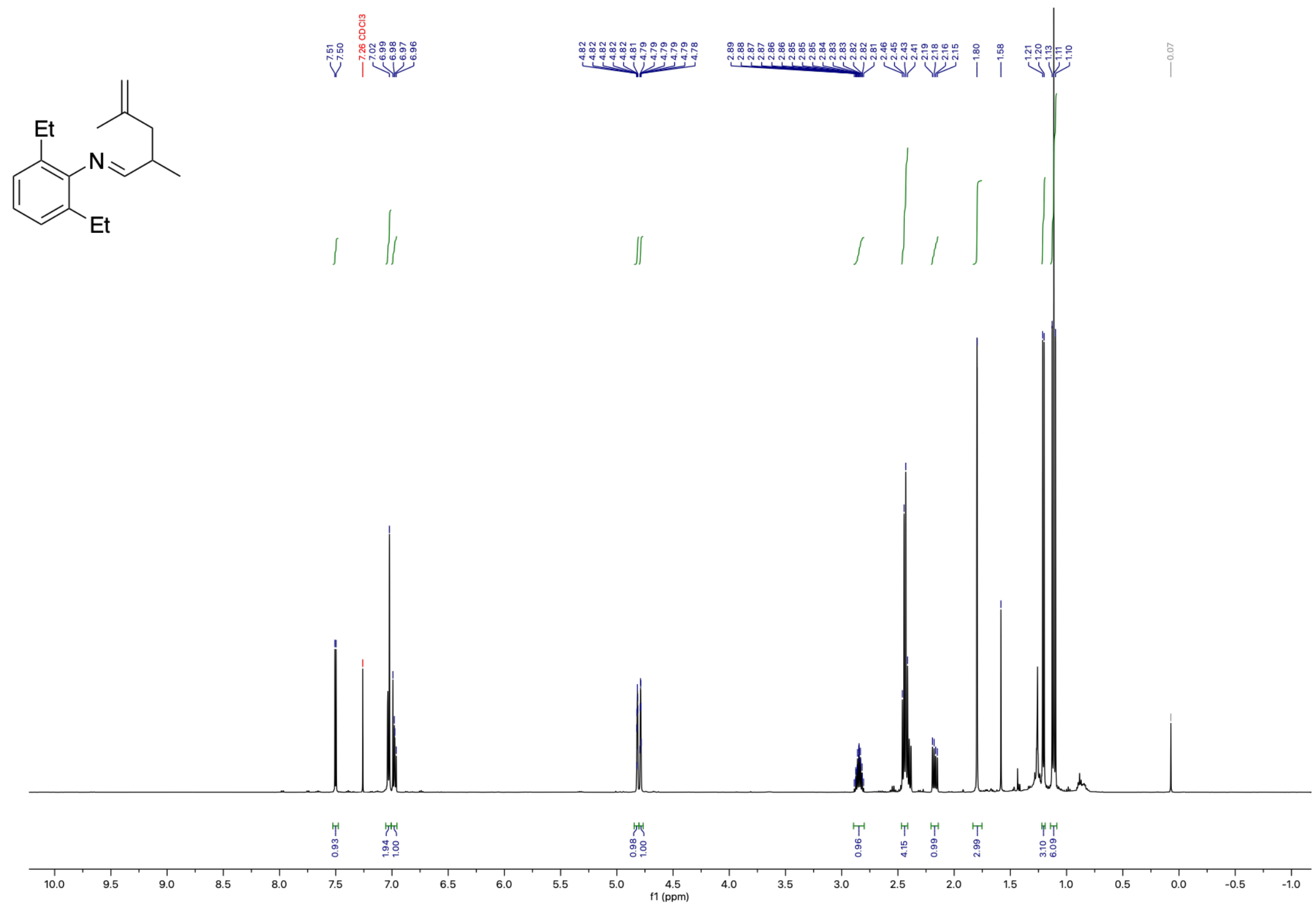

Figure S24. ^{1}H NMR spectrum of enamine **S8** in $CDCl_3$.

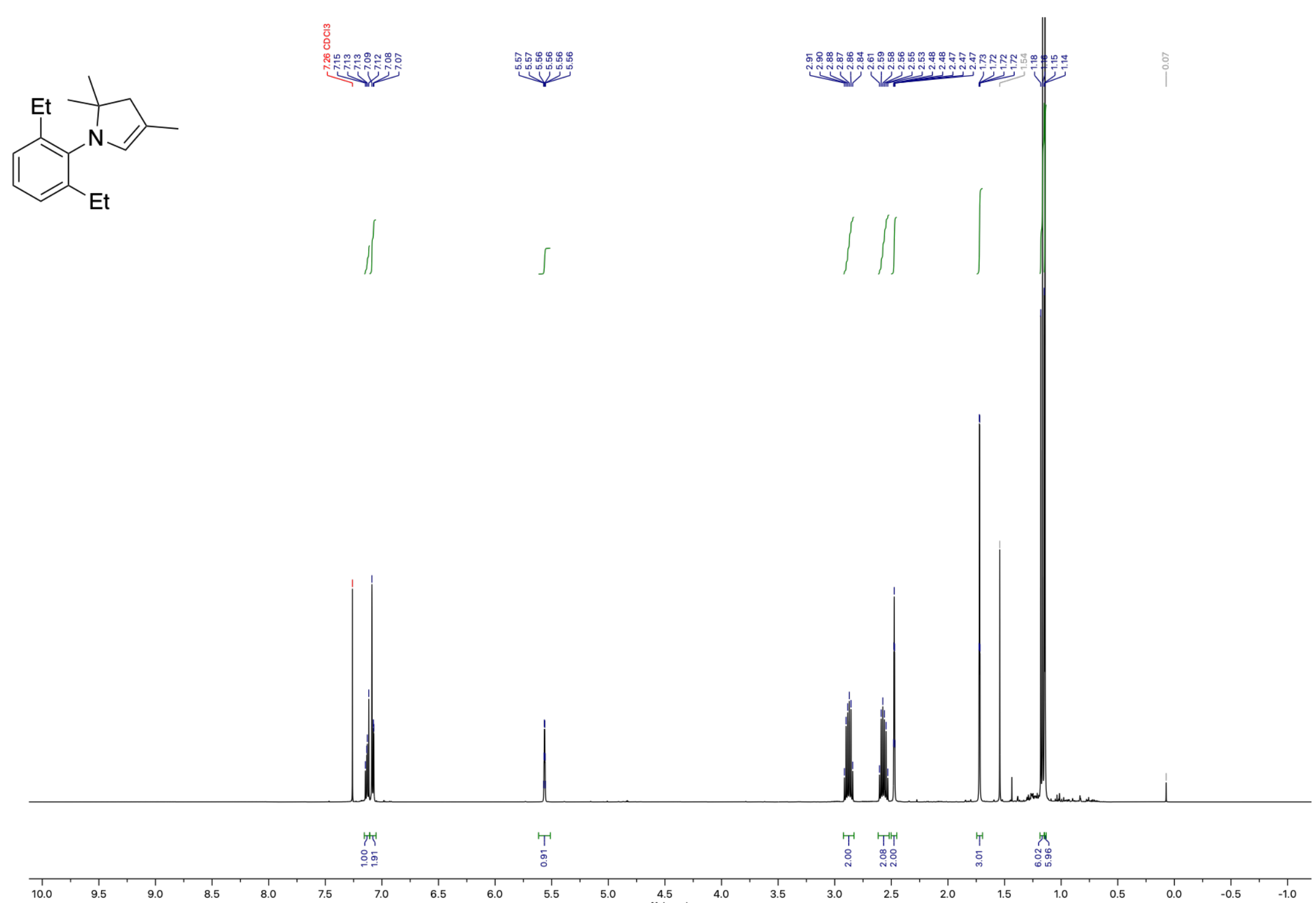

Figure S25. ^{13}C NMR spectrum of enamine **S8** in $CDCl_3$.

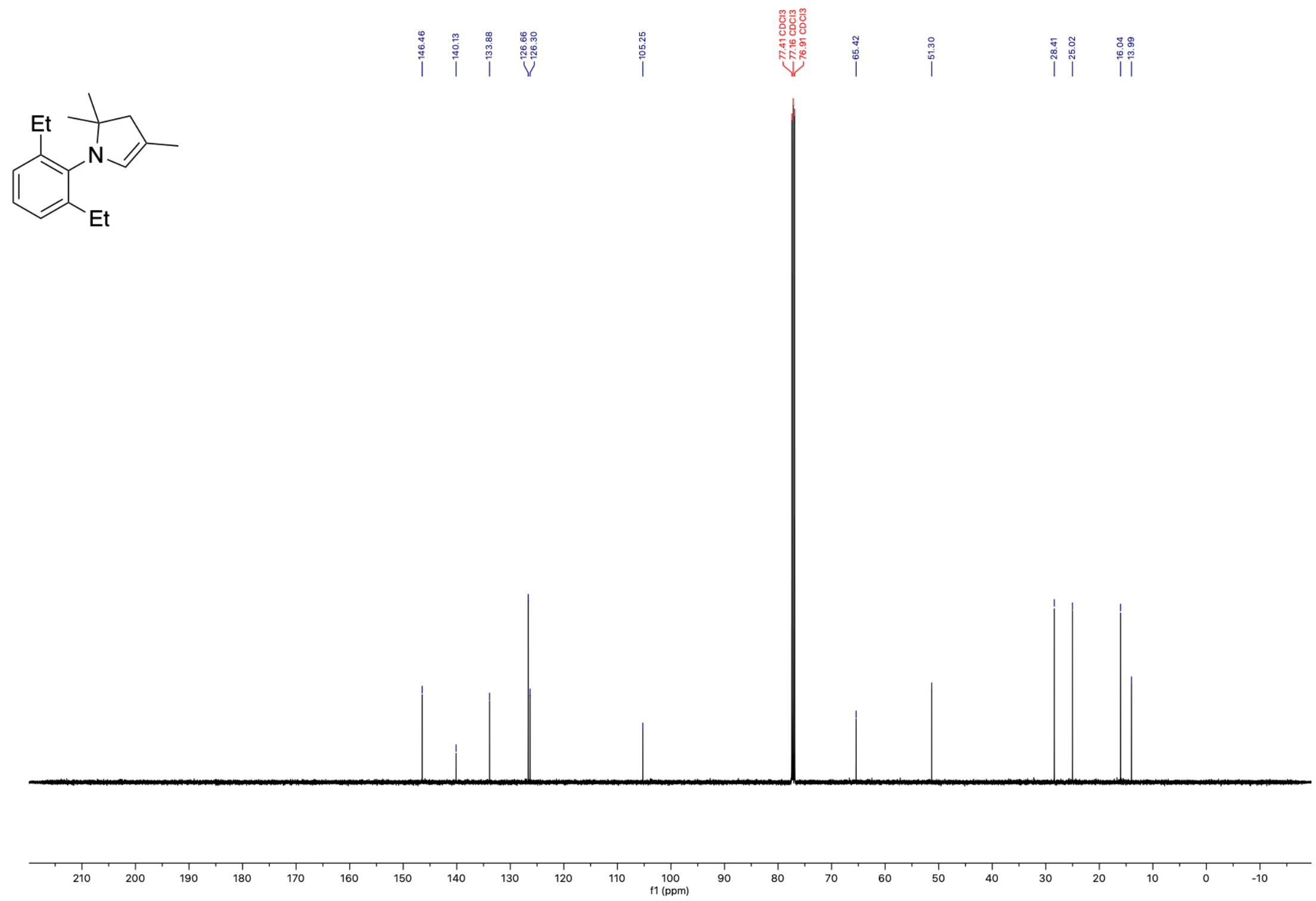

Figure S26. ^{1}H NMR spectrum of **Et_2CAAC-5-H BF_4 S9** in $CDCl_3$.

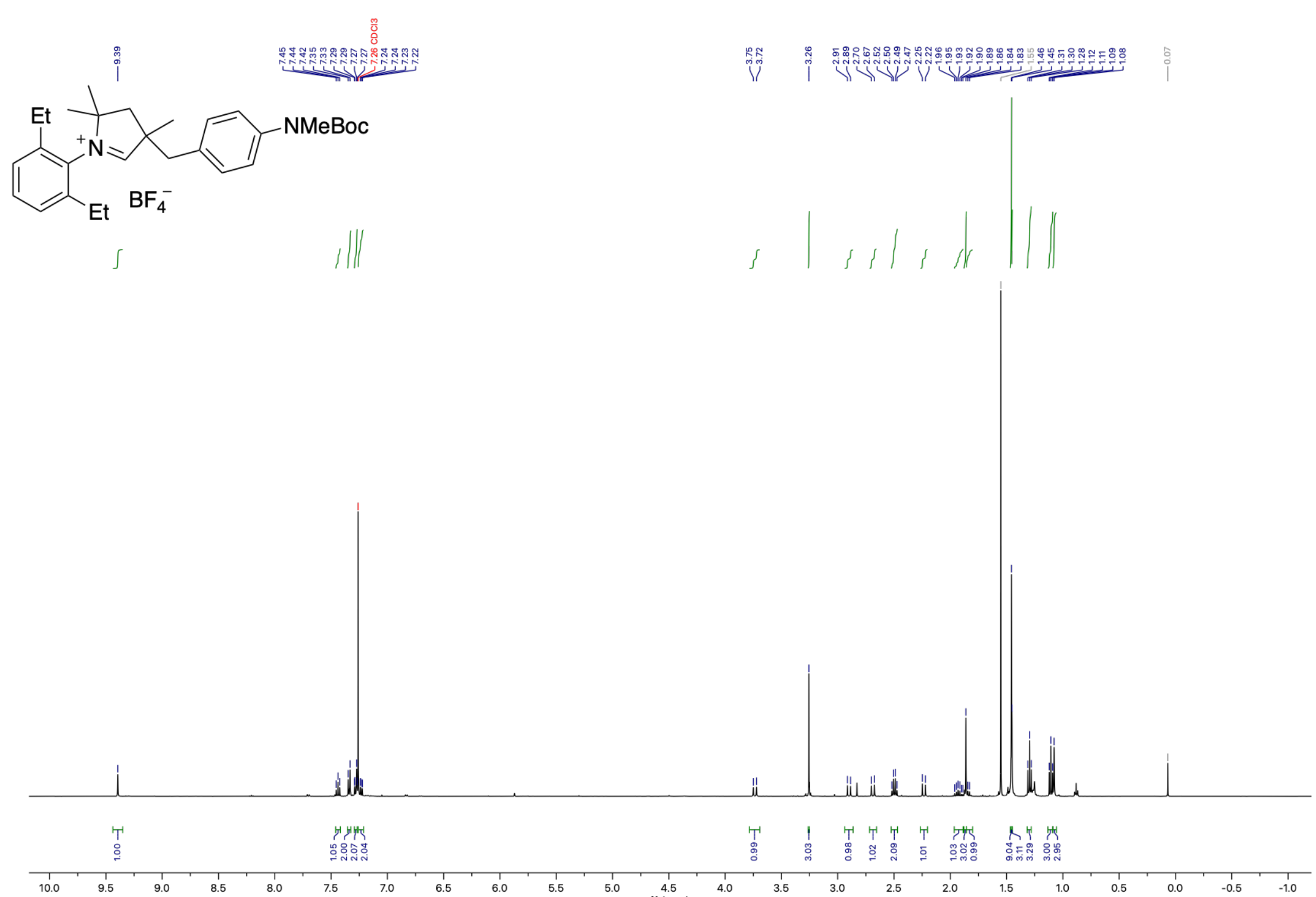

Figure S27. ^{13}C NMR spectrum of **Et_2CAAC-5-H BF_4 S9** in $CDCl_3$.

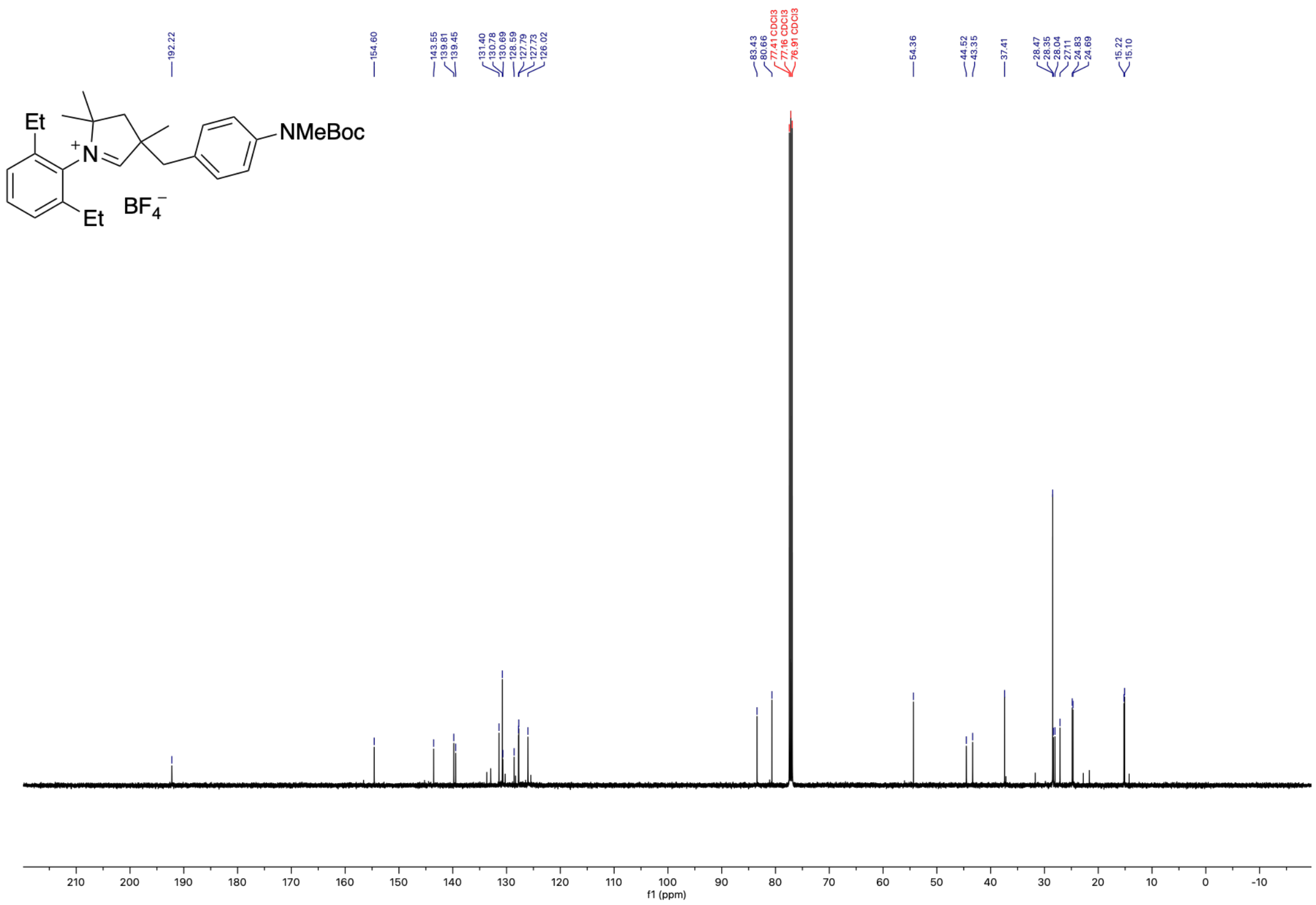

Figure S28. ^{1}H NMR spectrum of **[Ru(Et_2CAAC-5-Boc)] S10** in CD_2Cl_2.

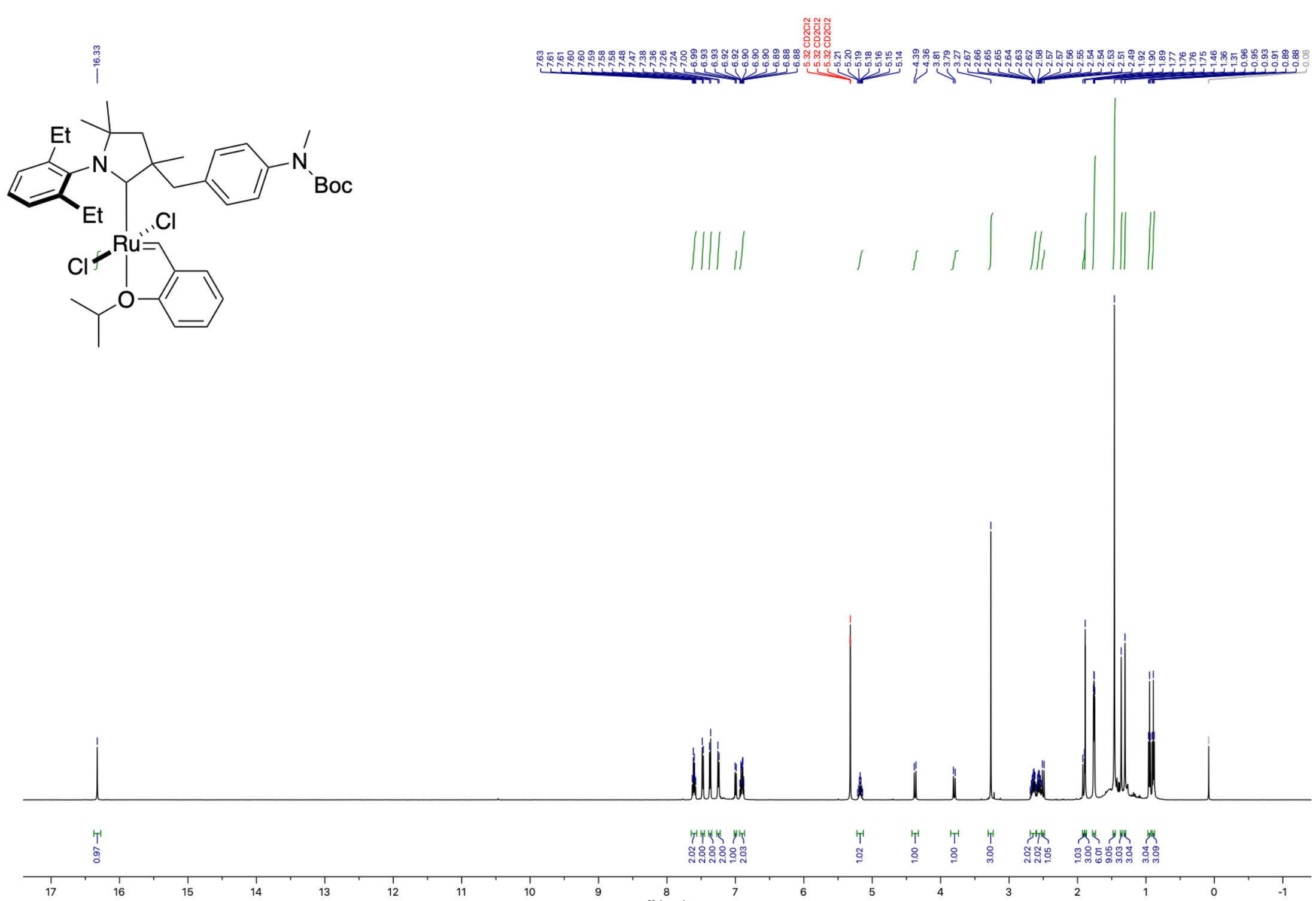

Figure S29. ^{13}C NMR spectrum of **[Ru(Et$_2$CAAC-5-Boc)] S10** in CD_2Cl_2.

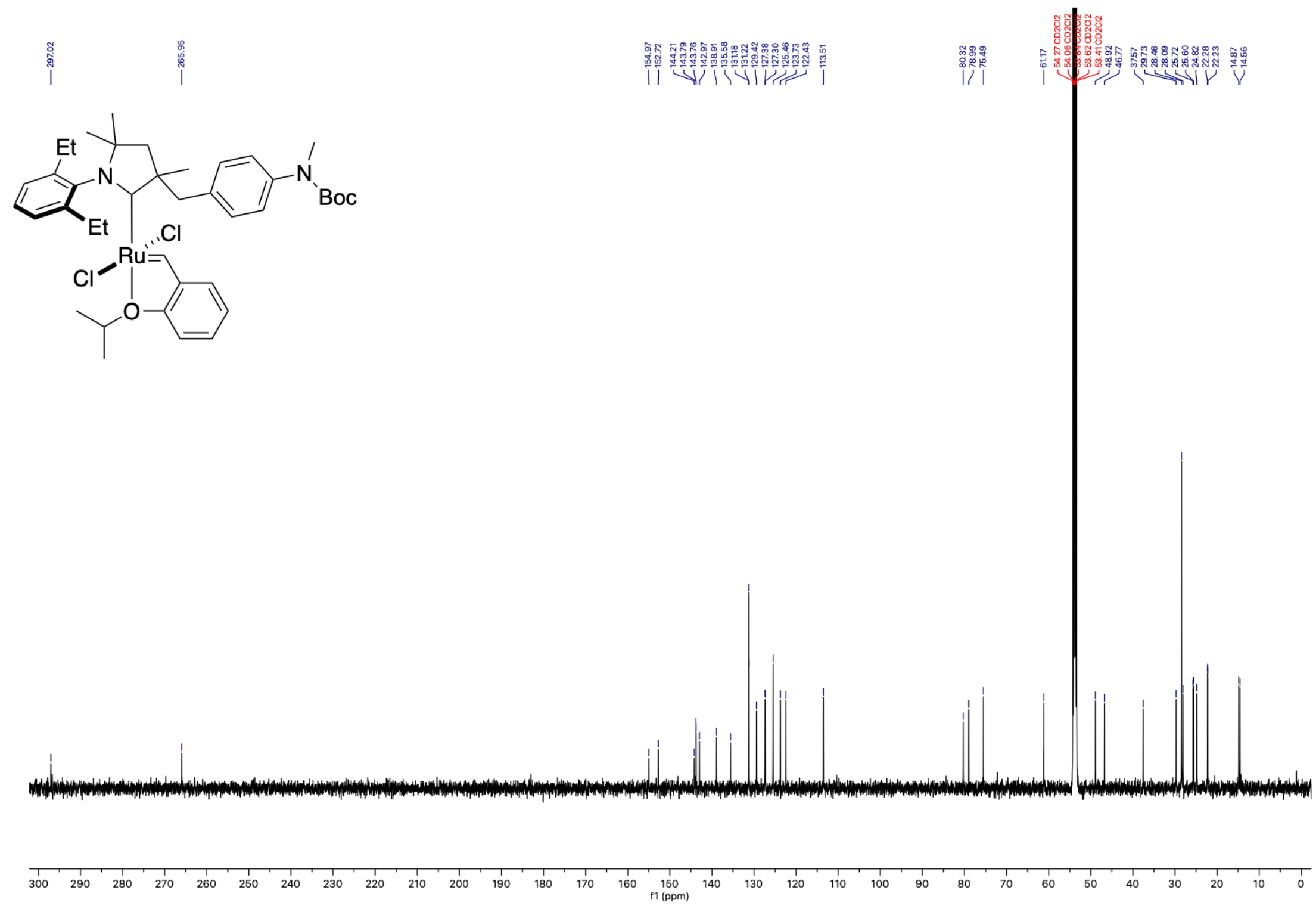

Figure S30. ^{1}H NMR spectrum of imine **S11** in $CDCl_3$.

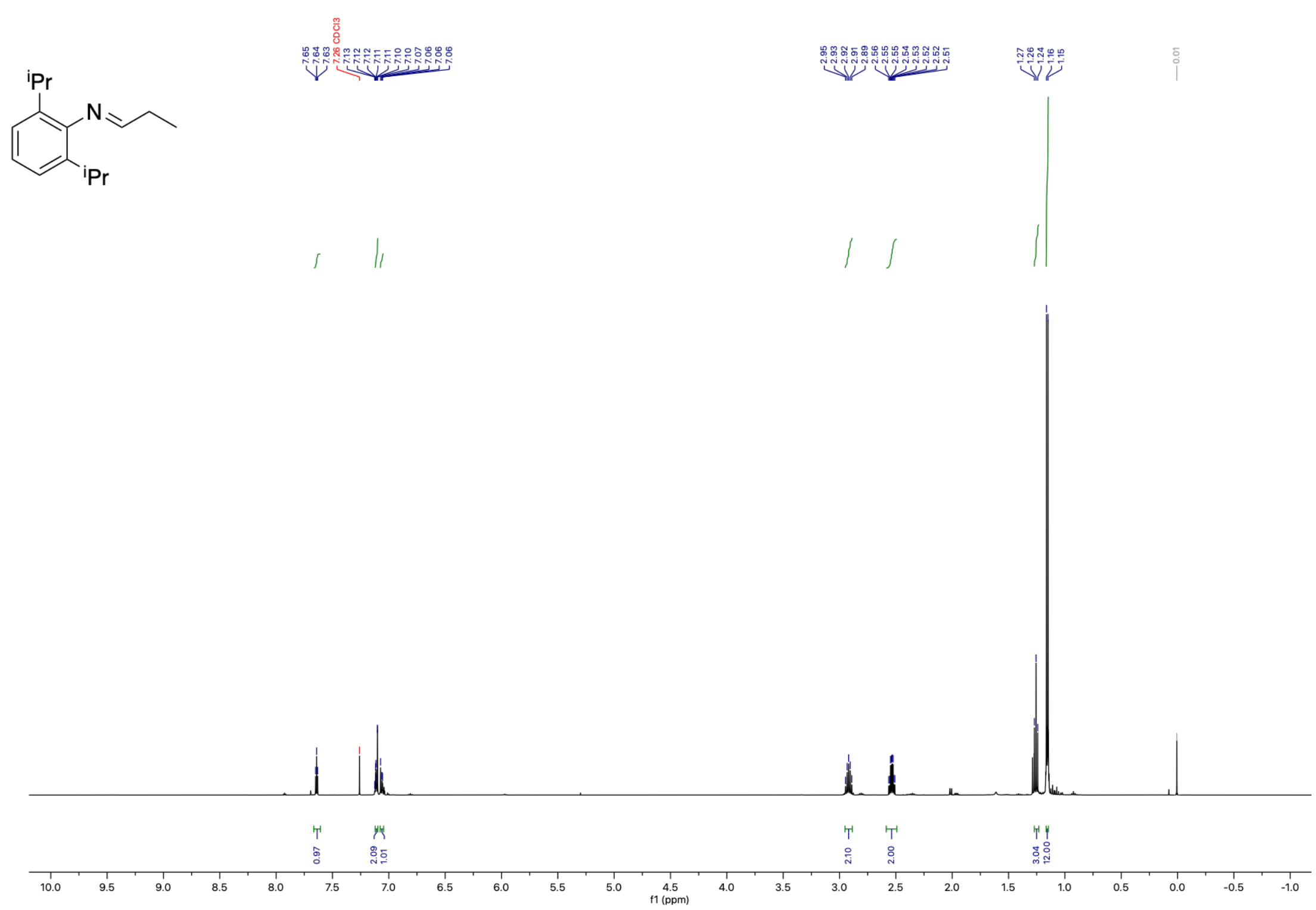

Figure S31. ^{1}H NMR spectrum of alkenyl aldimine **S12** in $CDCl_3$.

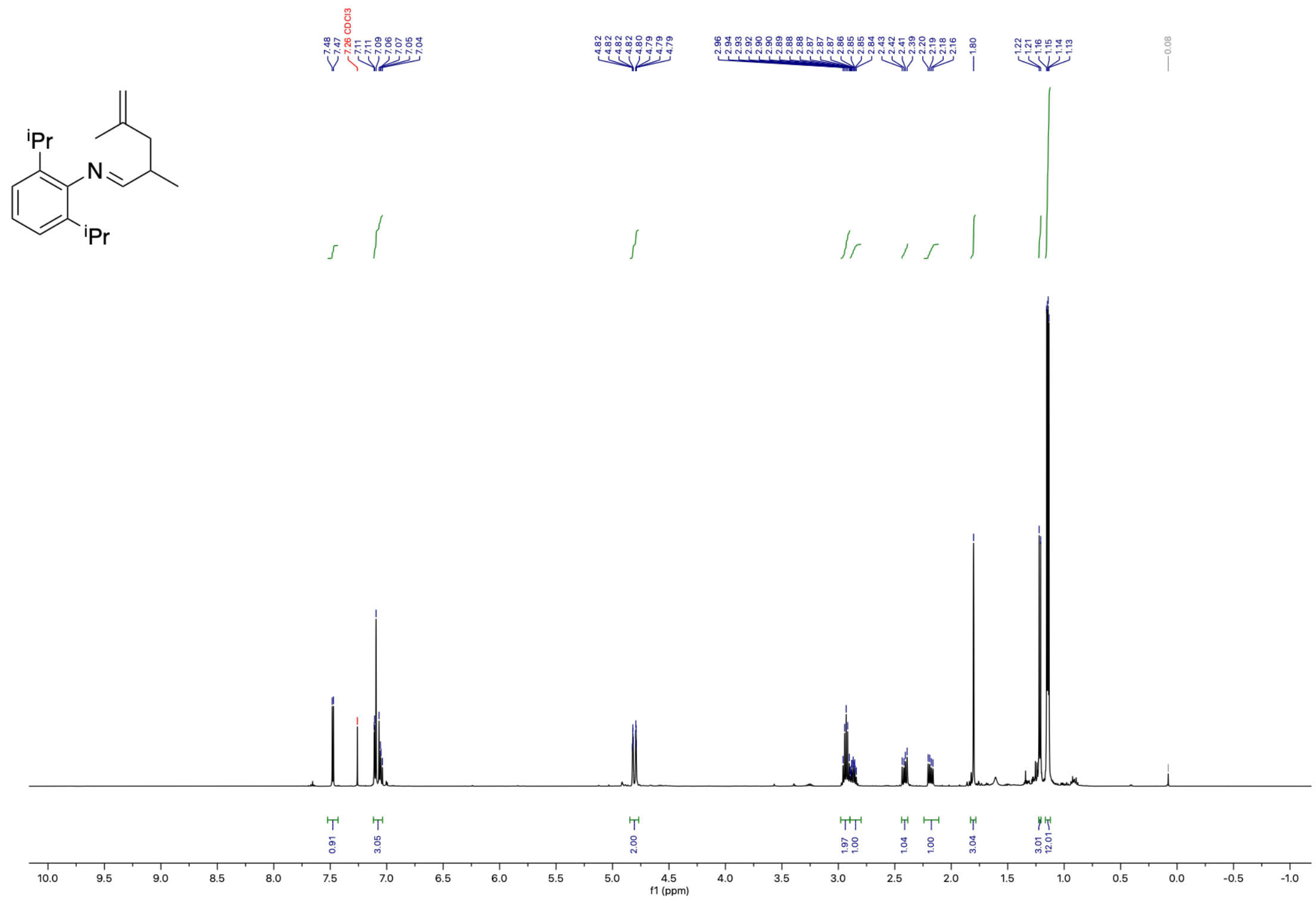

Figure S32. ^{1}H NMR spectrum of **$^iPr_2$CAAC-5-H BF_4 S13** in $CDCl_3$.

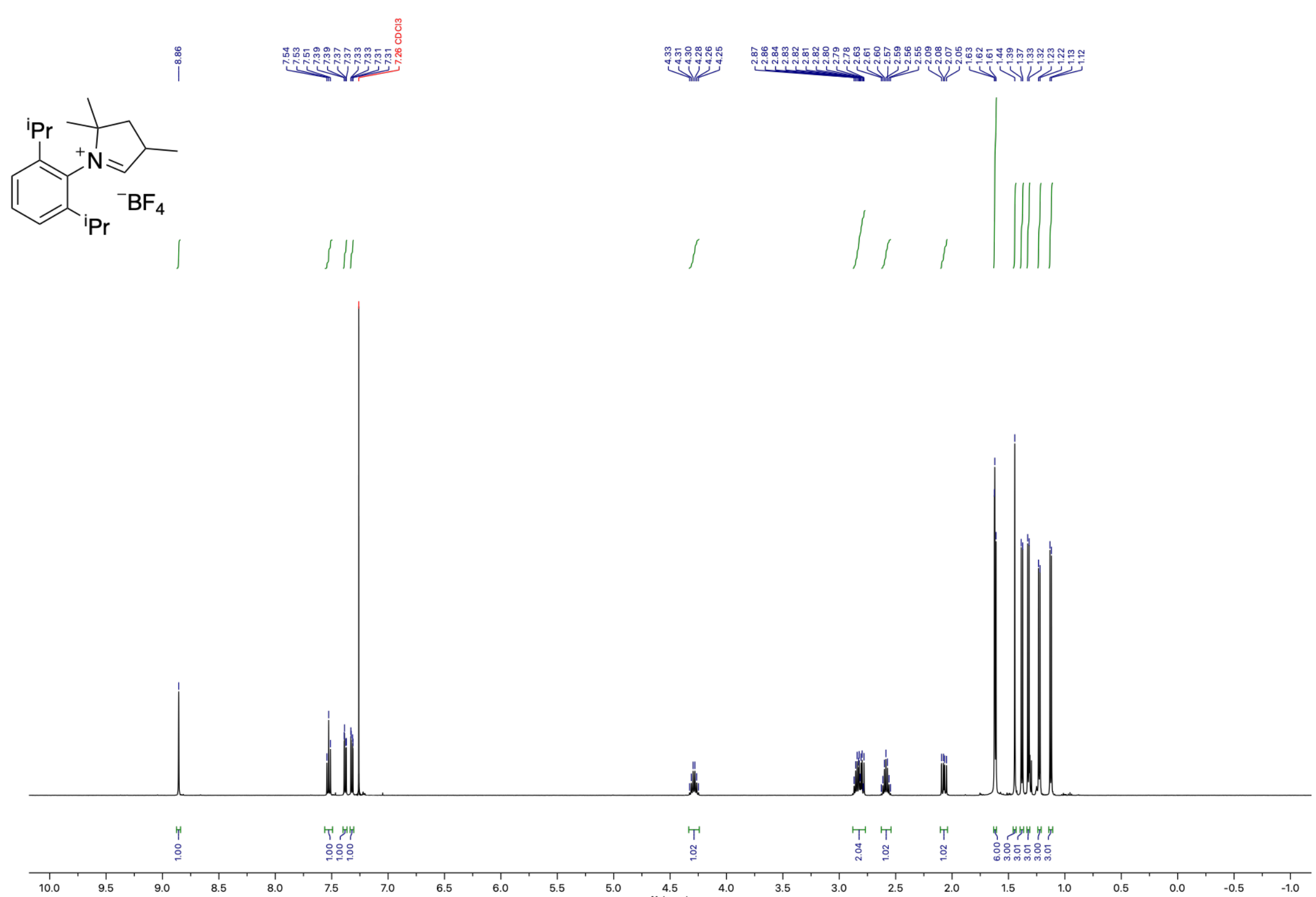

Figure S33. ^{1}H NMR spectrum of enamine **S14** in $CDCl_3$.

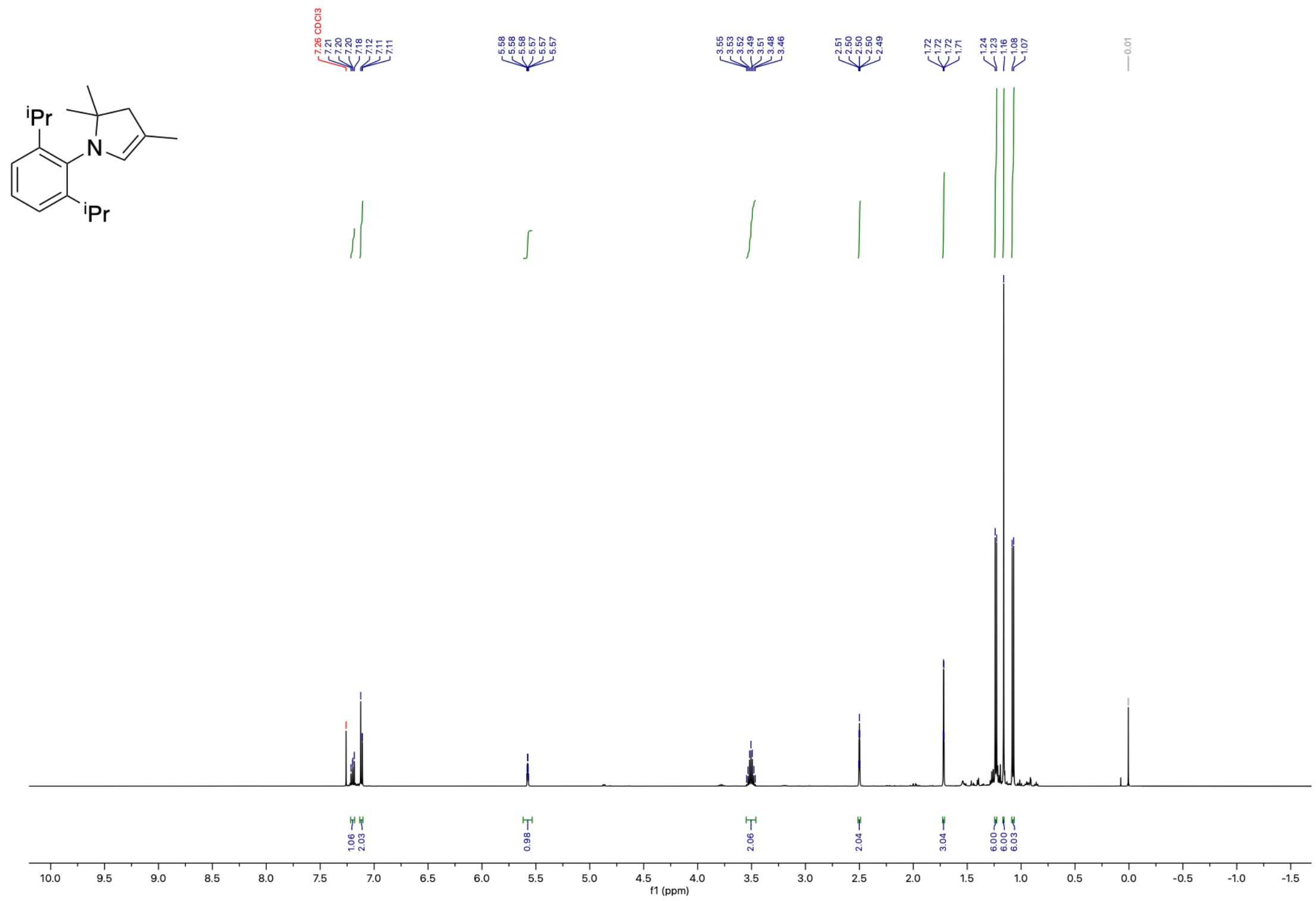

Figure S34. ^{1}H NMR spectrum **of** $^{\mathbf{i}}$**Pr$_2$CAAC-5-H BF$_4$ S15** in $CDCl_3$.

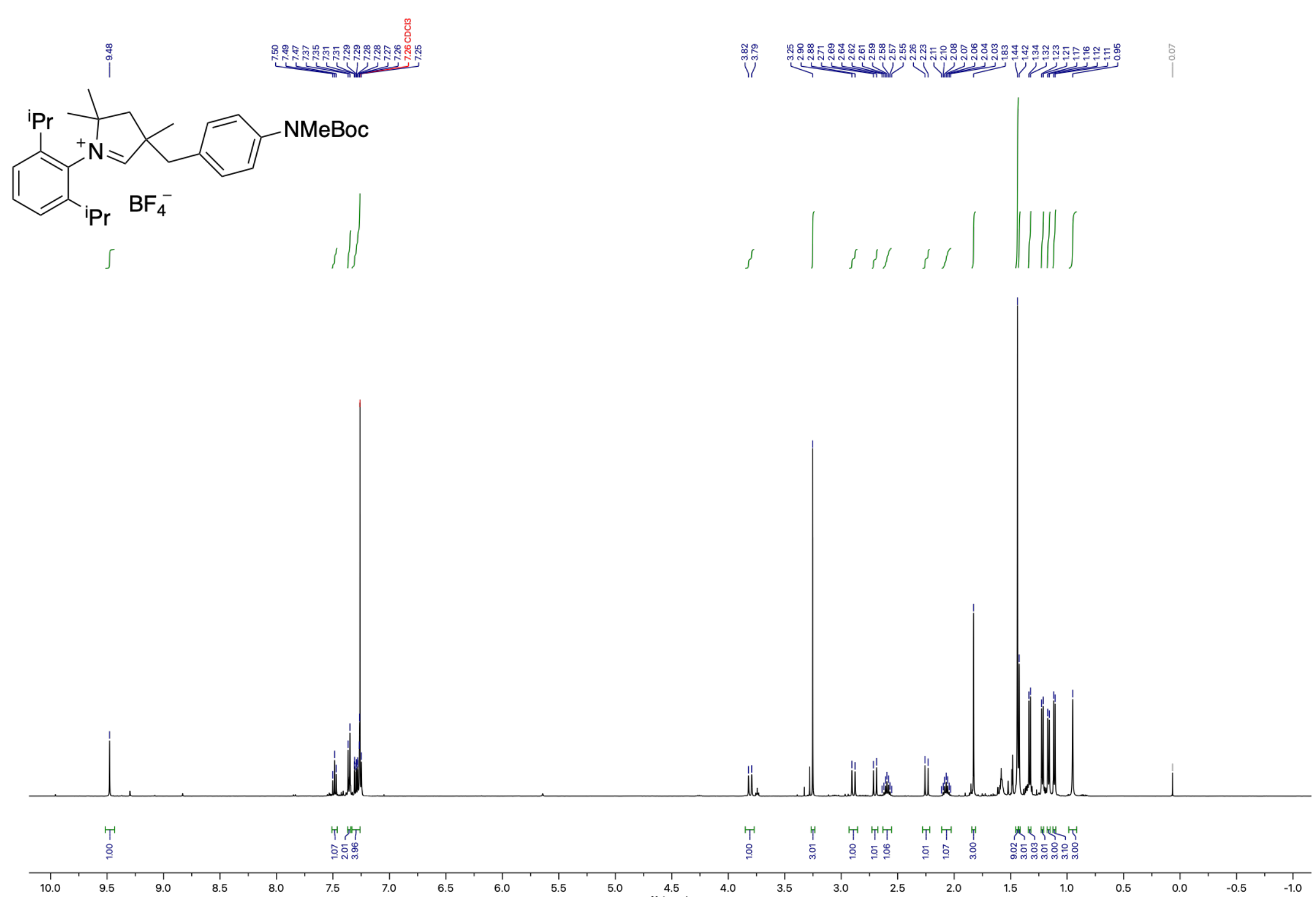

Figure S35. ^{13}C NMR spectrum of **^{i}Pr$_2$CAAC-5-H BF$_4$ S15** in $CDCl_3$.

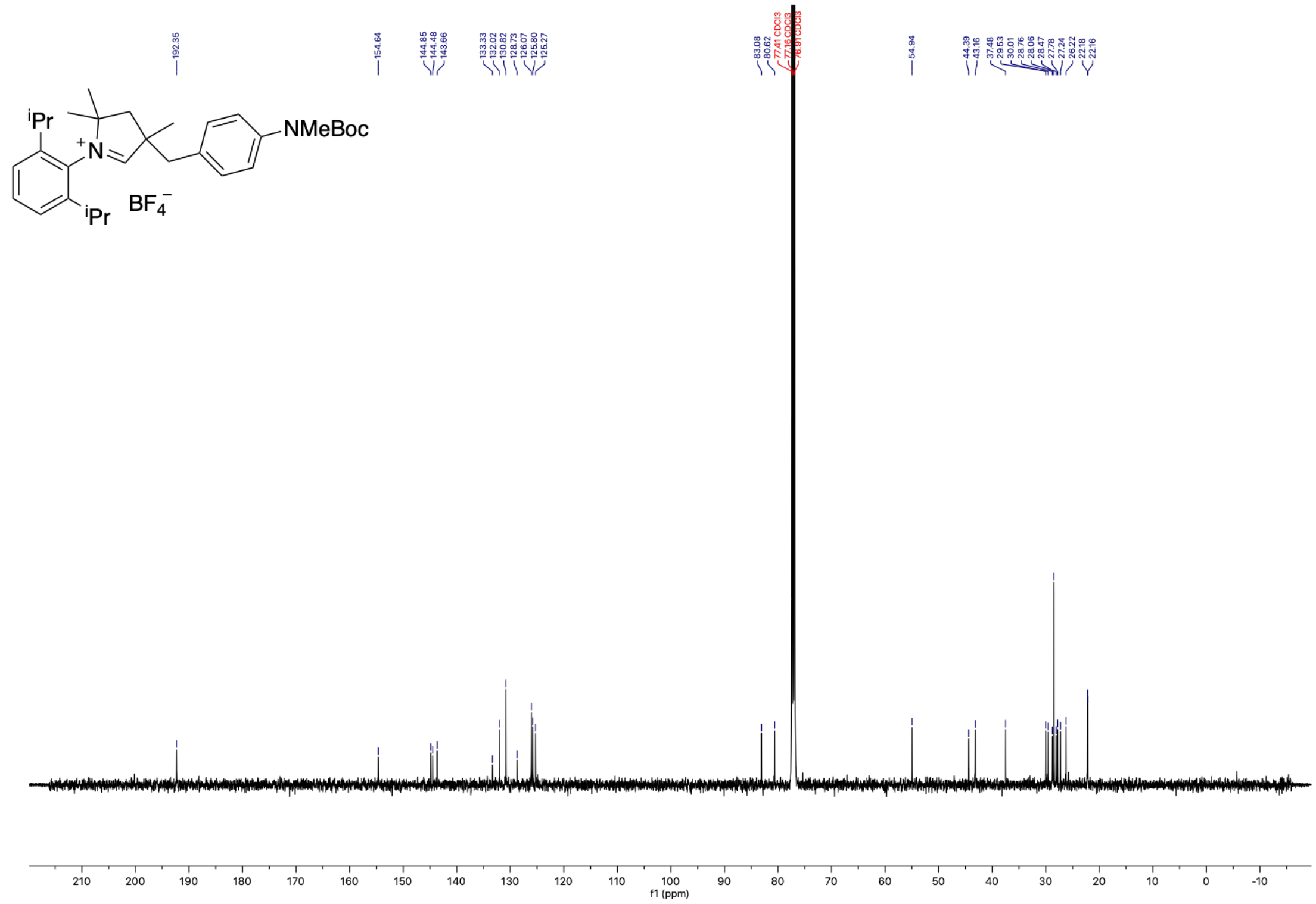

Figure S36. ^{1}H NMR spectrum **of [Ru(^{i}Pr$_2$CAAC-5-Boc)] S16** in CD_2Cl_2.

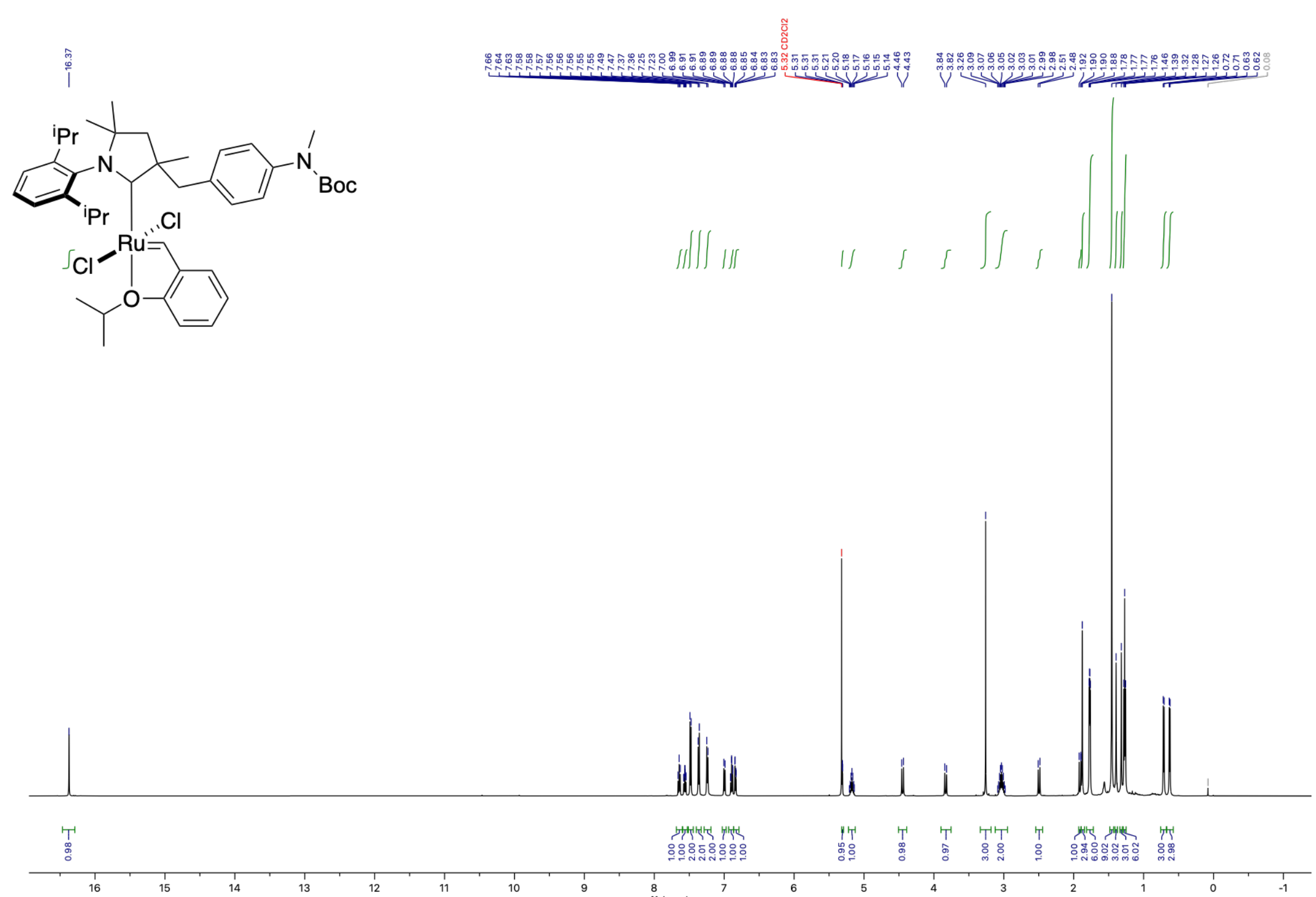

Figure S37. ^{13}C NMR spectrum of **[Ru(^{i}Pr$_2$CAAC-5-Boc)] S16** in CD_2Cl_2.

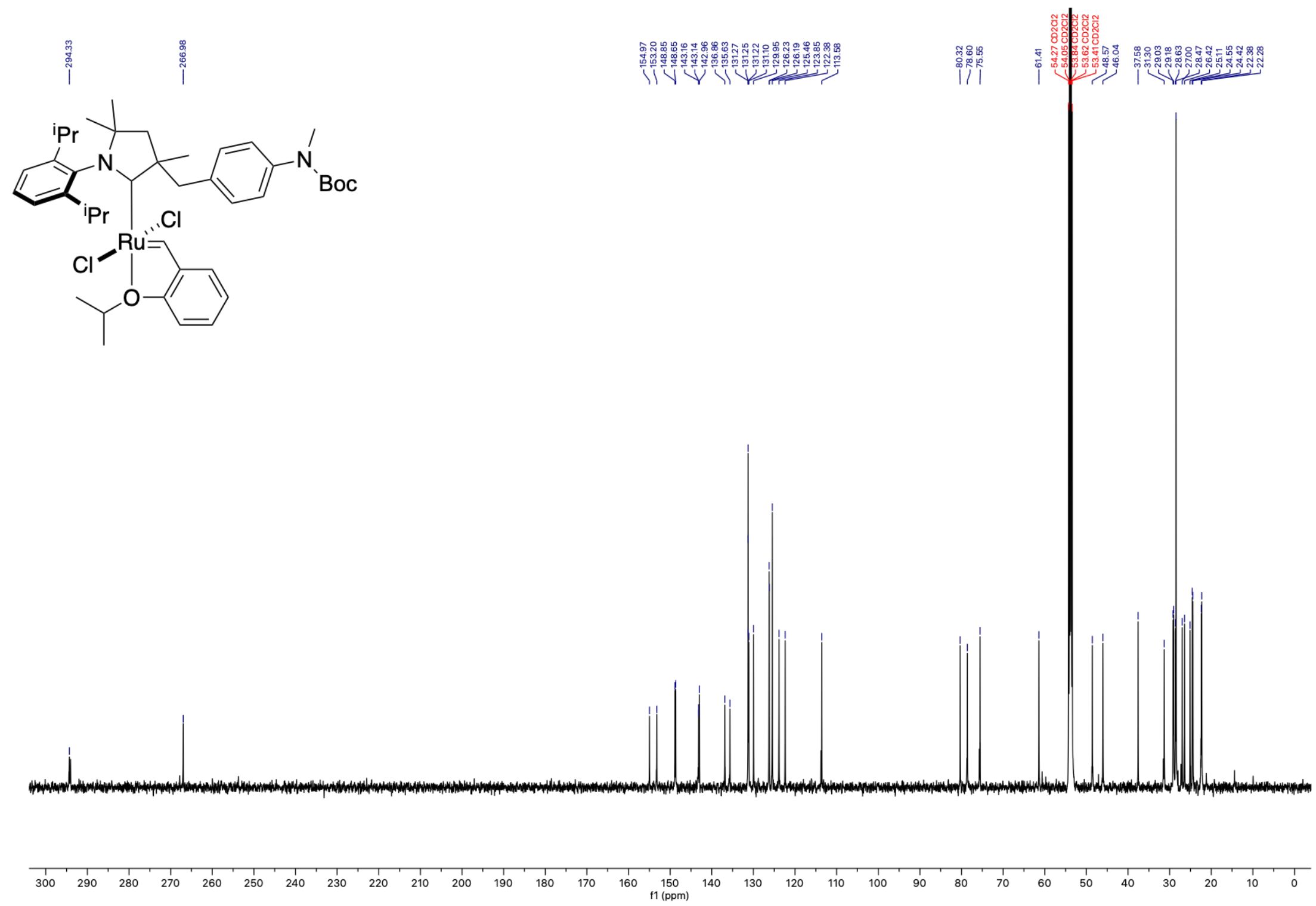

Figure S38. ^{1}H NMR spectrum of **[Ru(^{i}Pr$_2$CAAC-5)] S17** in CD_2Cl_2.

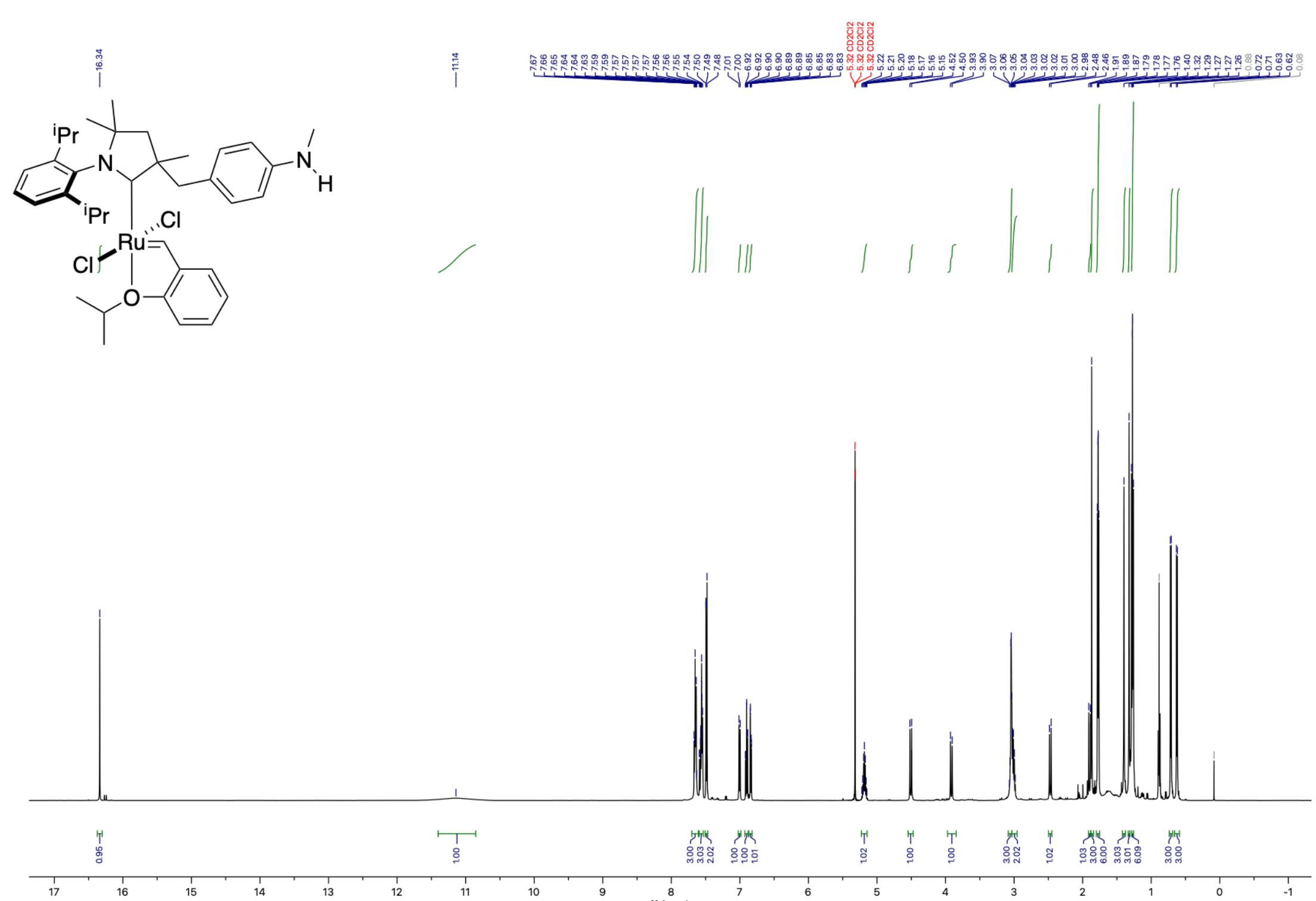

Figure S39. ^{13}C NMR spectrum of **[Ru(^{i}Pr$_2$CAAC-5)] S17** in CD_2Cl_2.

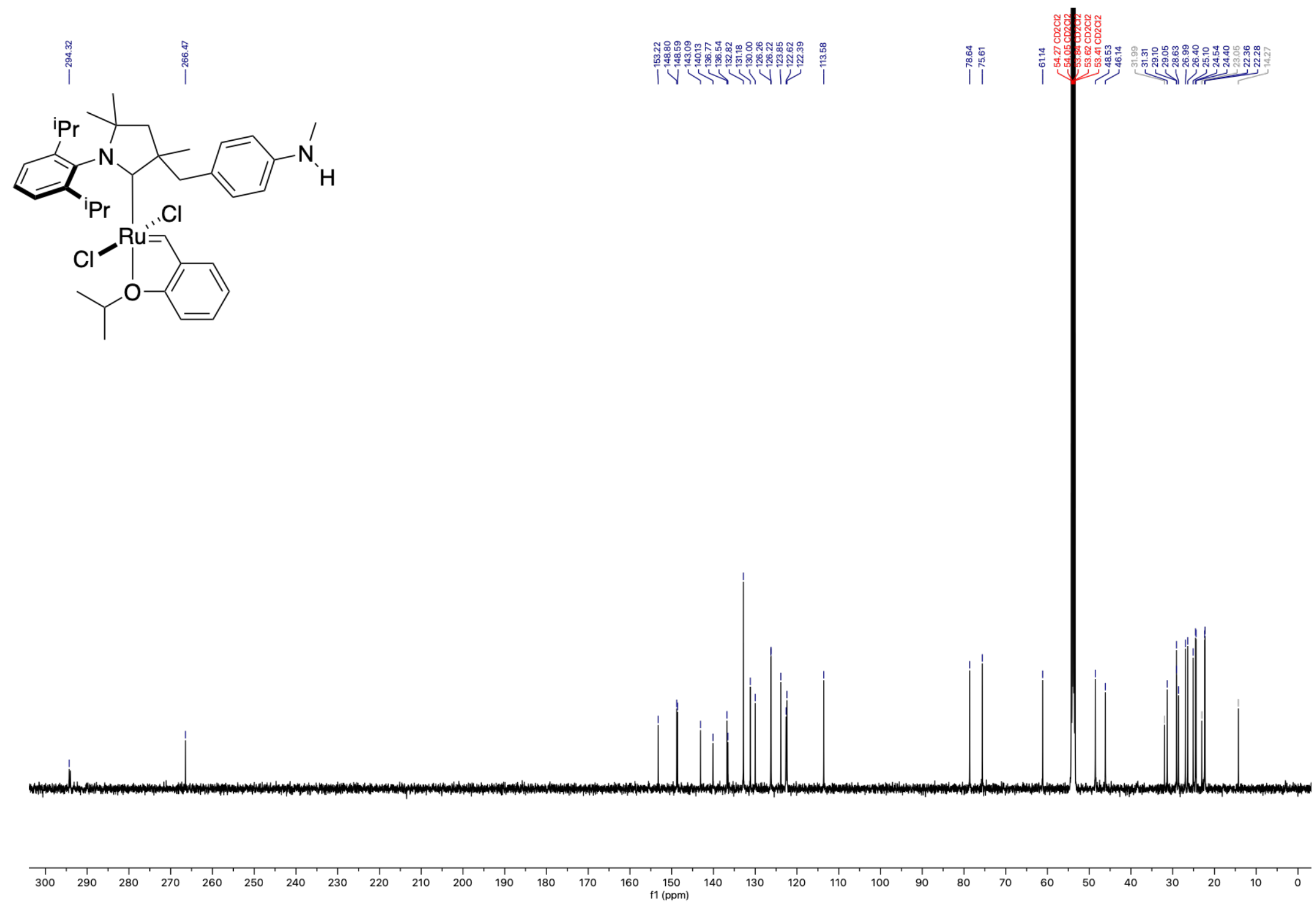

Figure S40. ^{1}H NMR spectrum of 4-sulfamoylbenzoyl chloride (**S18**) in $CDCl_3$.

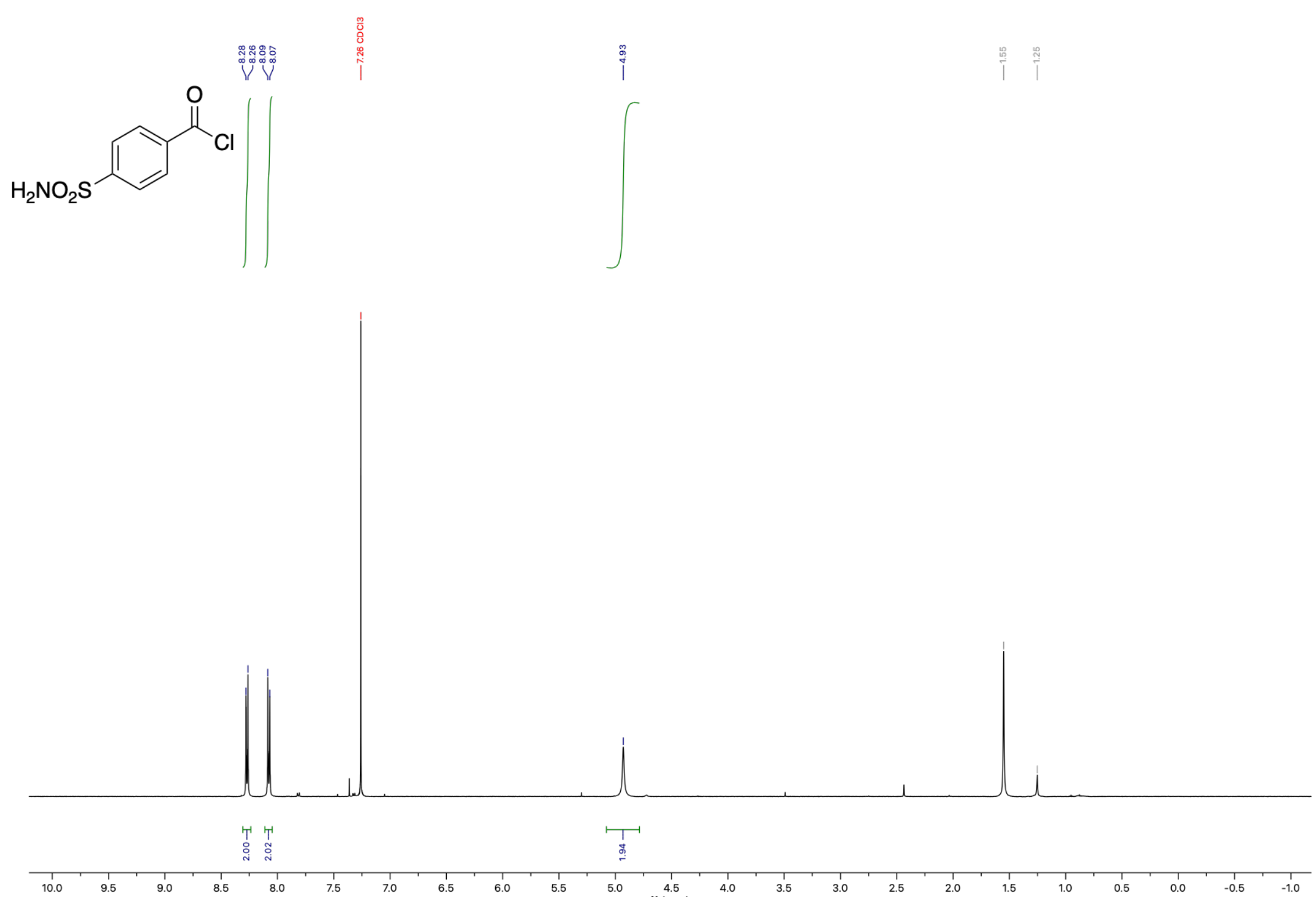

Figure S41. ^{1}H NMR spectrum of **[Ru($^{i}Pr_2$CAAC-5-SO_2NH_2)] S19** in CD_2Cl_2.

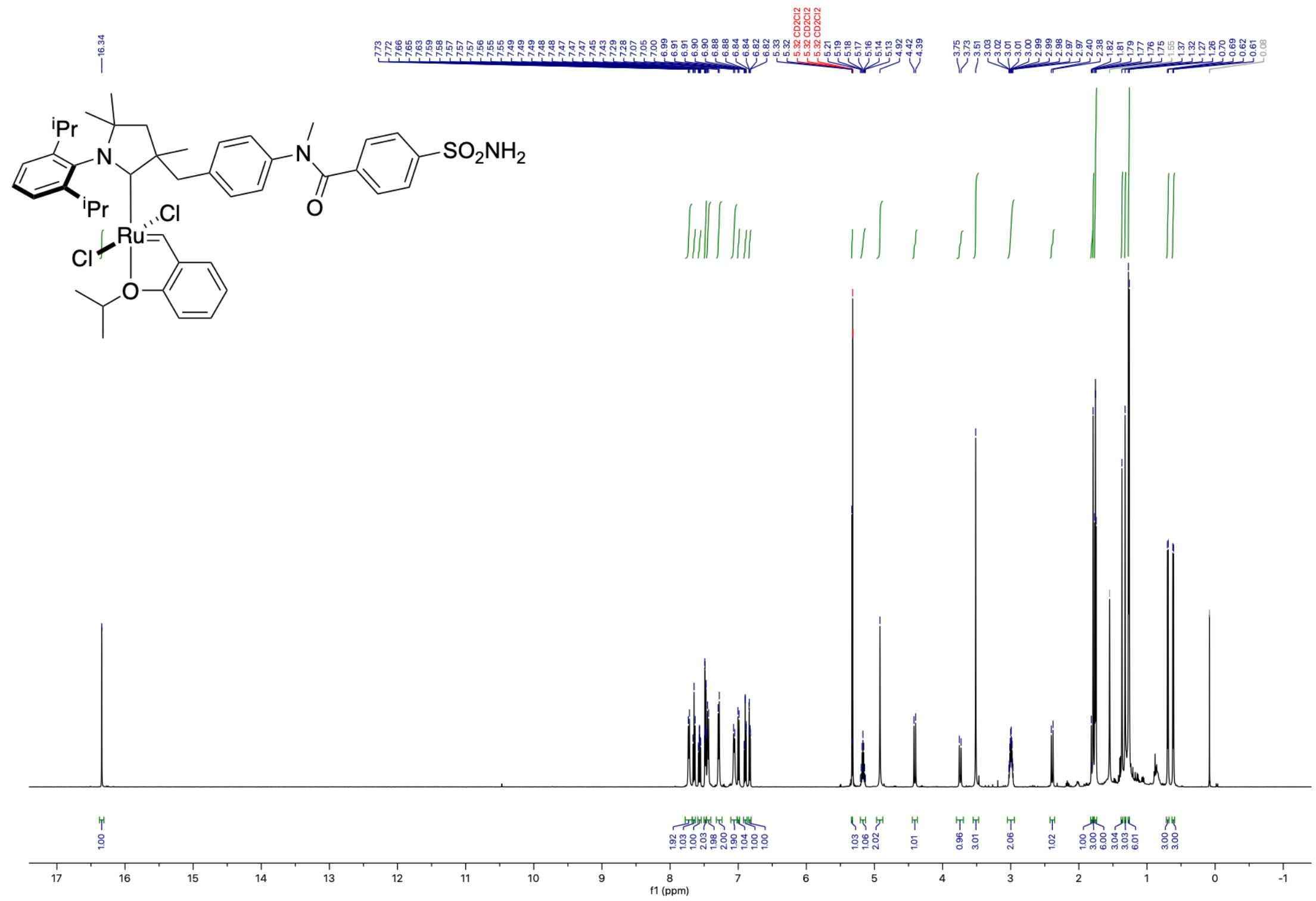

Figure S42. ^{13}C NMR spectrum of **[Ru($^{i}Pr_2$CAAC-5-SO_2NH_2)] S19** in CD_2Cl_2.

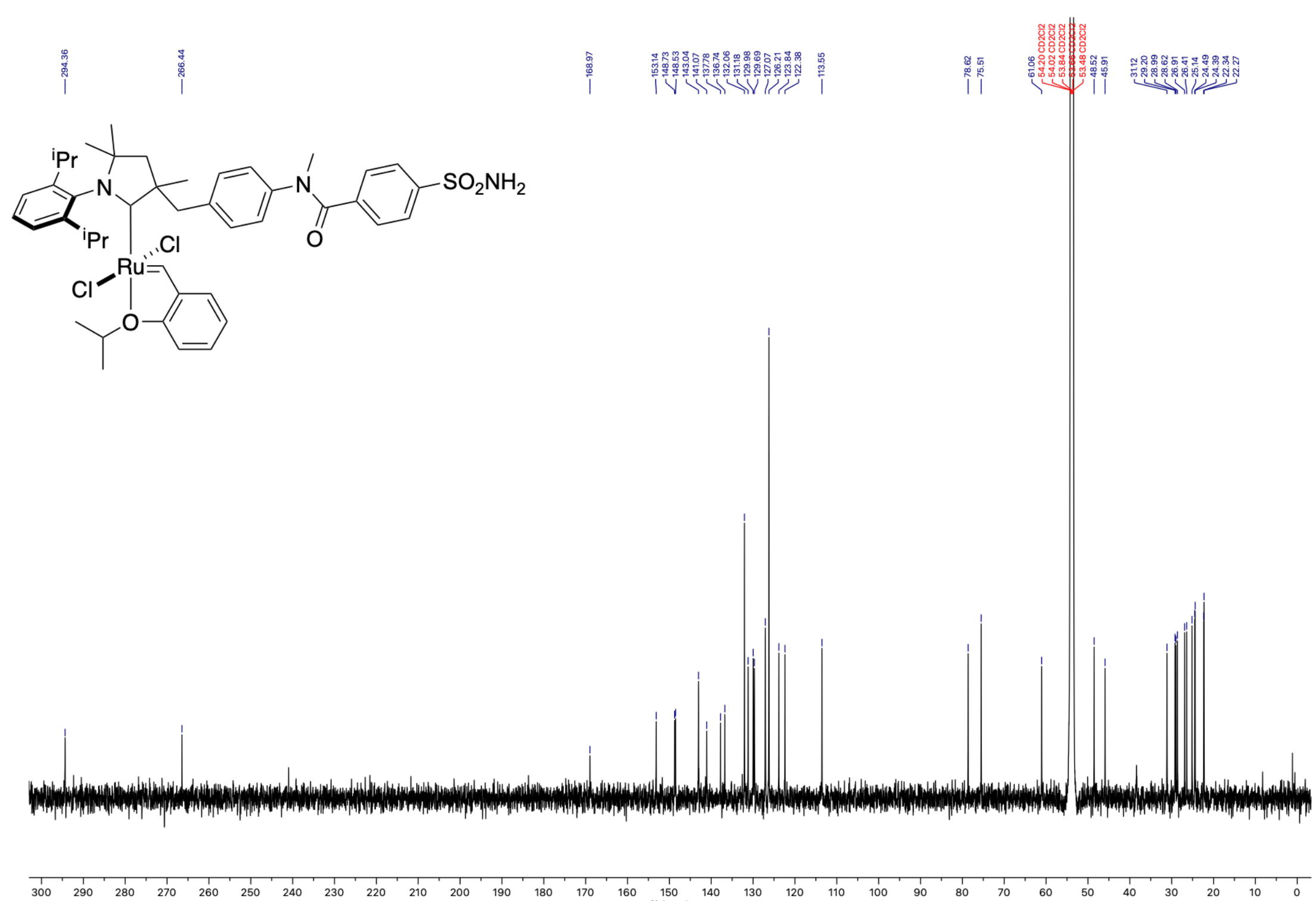

Figure S43. ^{1}H NMR spectrum of 2-azidoacetyl chloride (**S20**) in $CDCl_3$.

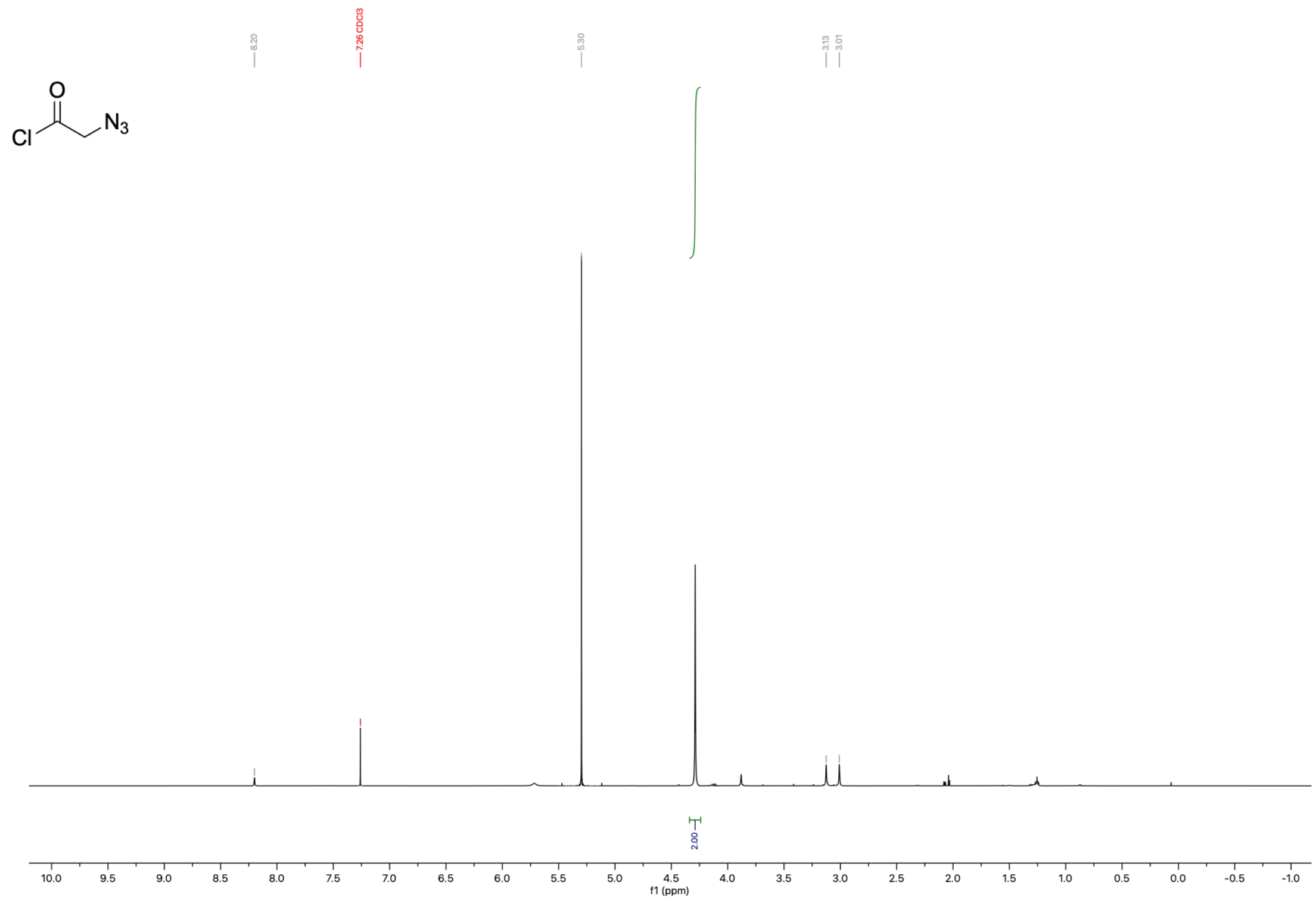

Figure S44. ^{1}H NMR spectrum of **[Ru(^{i}Pr$_2$CAAC-5-N$_3$)] S21** in CD_2Cl_2.

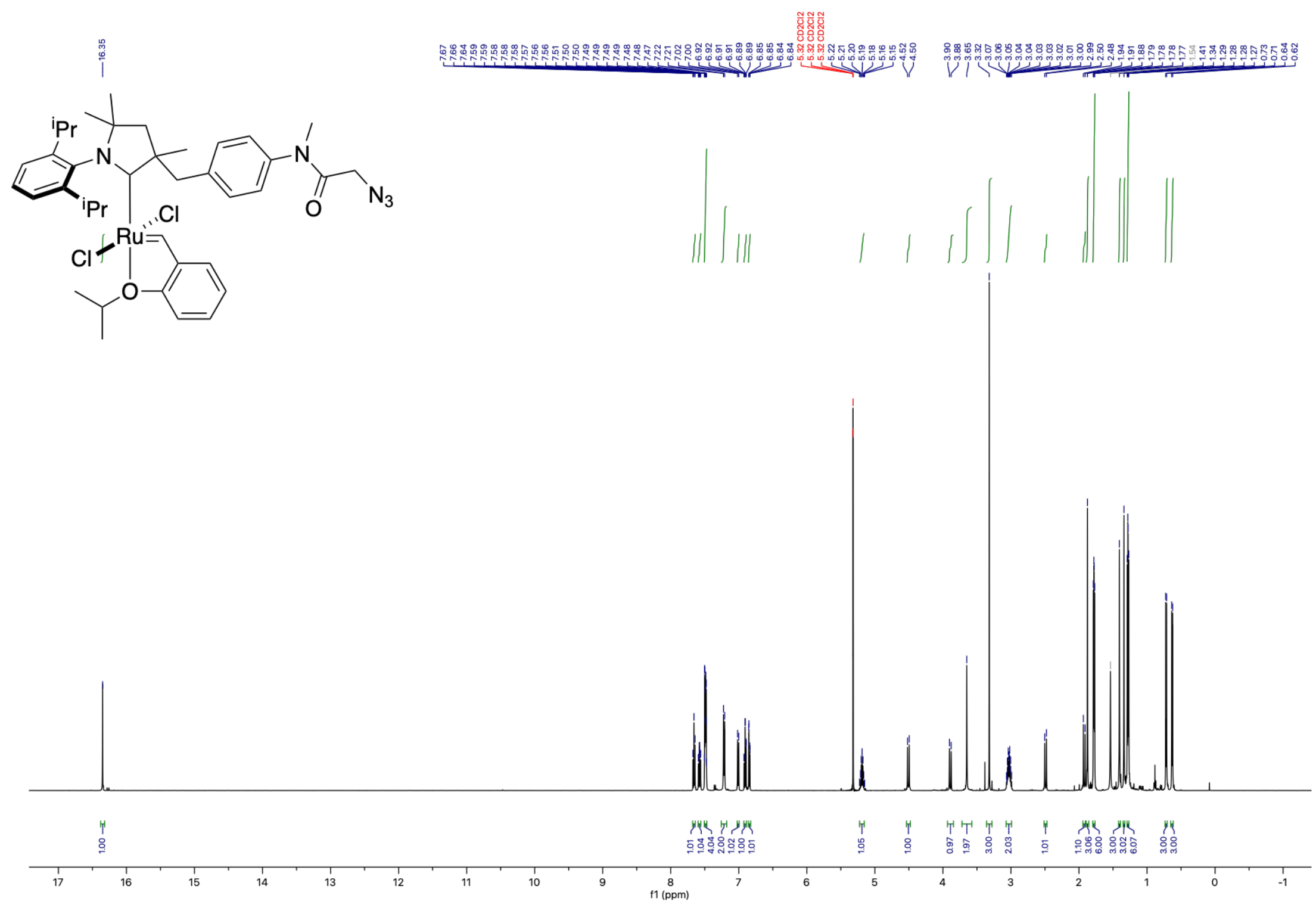

Figure S45. ^{13}C NMR spectrum of **[Ru(^{i}Pr$_2$CAAC-5-N$_3$)] S21** in CD_2Cl_2.

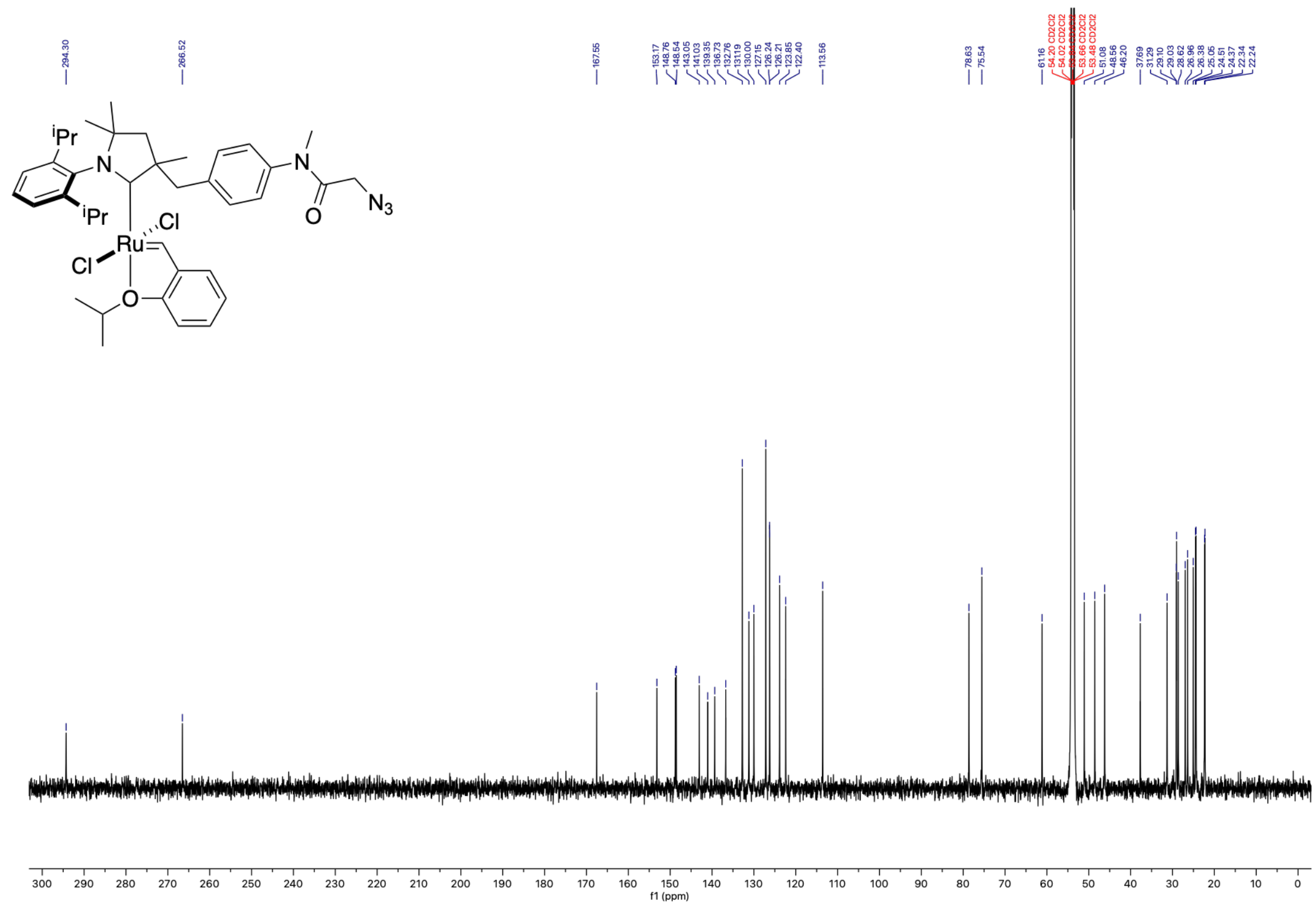

Figure S46. ^{1}H NMR spectrum of **[Ru(^{i}Pr$_2$CAAC-5-Br)] S22** in CD_2Cl_2.

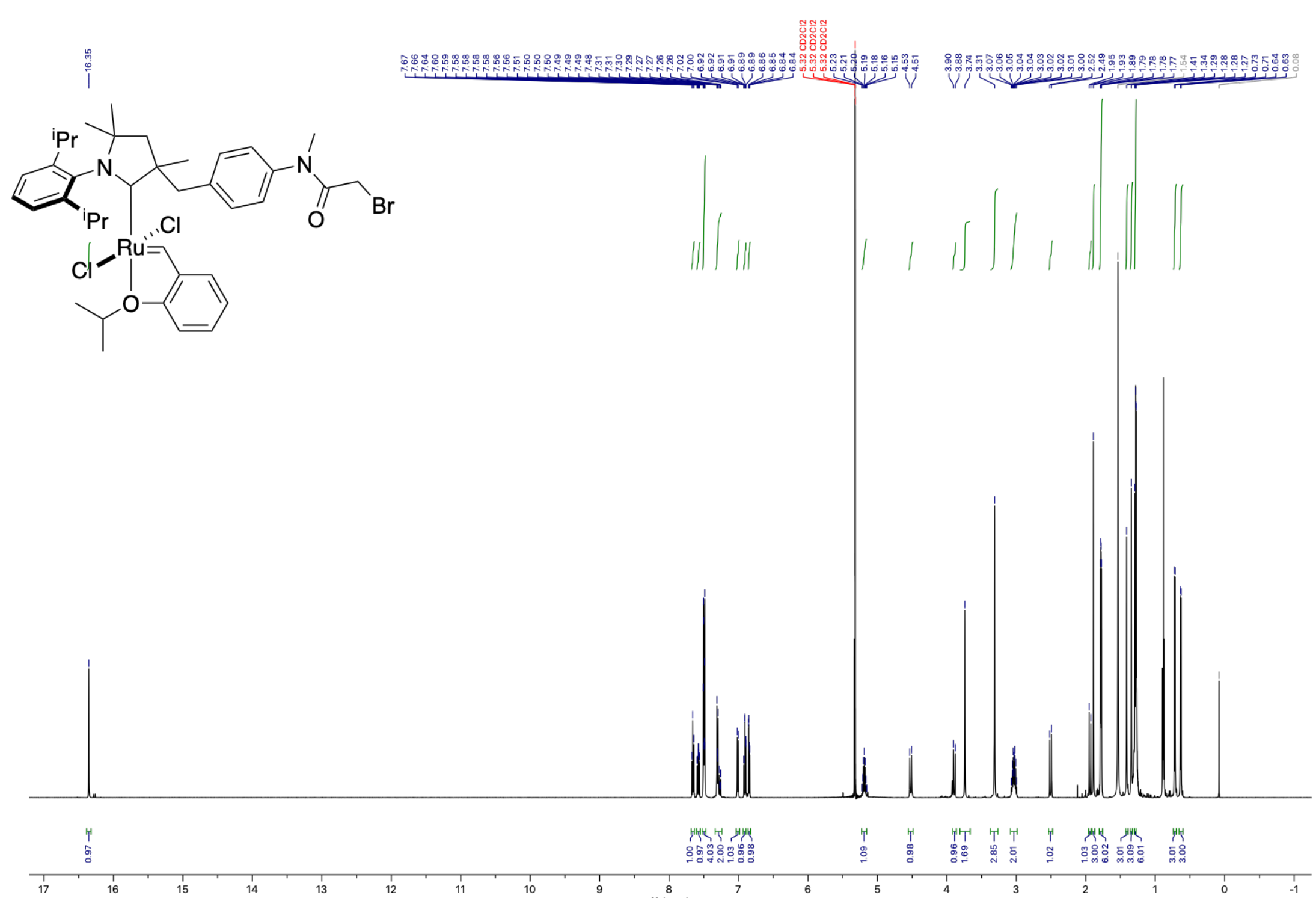

Figure S47. ^{13}C NMR spectrum of **[Ru(^{i}Pr$_2$CAAC-5-Br)] S22** in CD_2Cl_2.

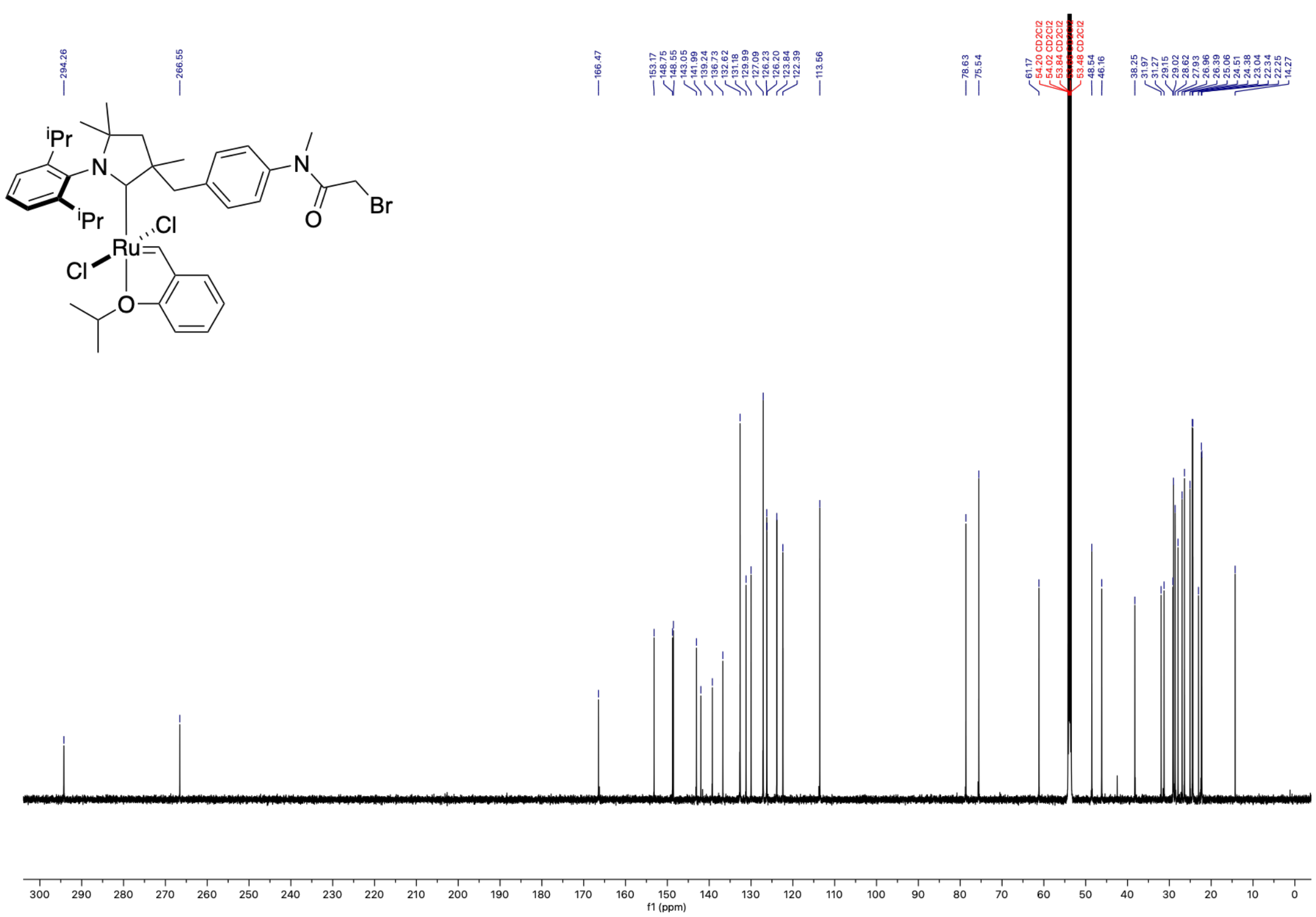

Figure S48. ^{1}H NMR spectrum of perfluorophenyl hex-5-ynoate (**S23**) in $CDCl_3$.

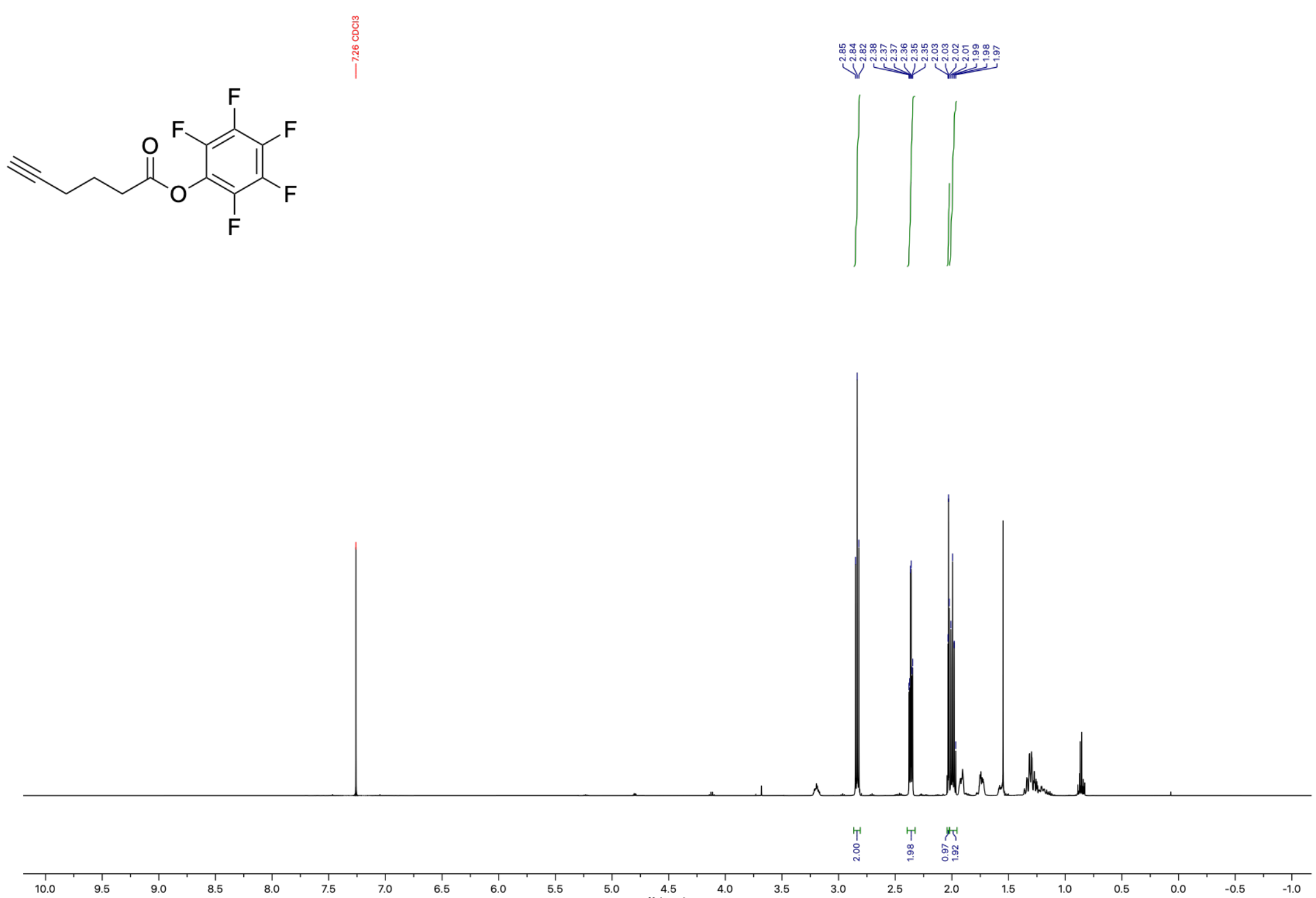

Figure S49. ^{1}H NMR spectrum of **[Ru(Et$_2$CAAC-5-Boc)]** in DMSO-d$_6$/D$_2$O (4:1) over time, normalized to the DMSO-d$_5$ signal (2.50 ppm).

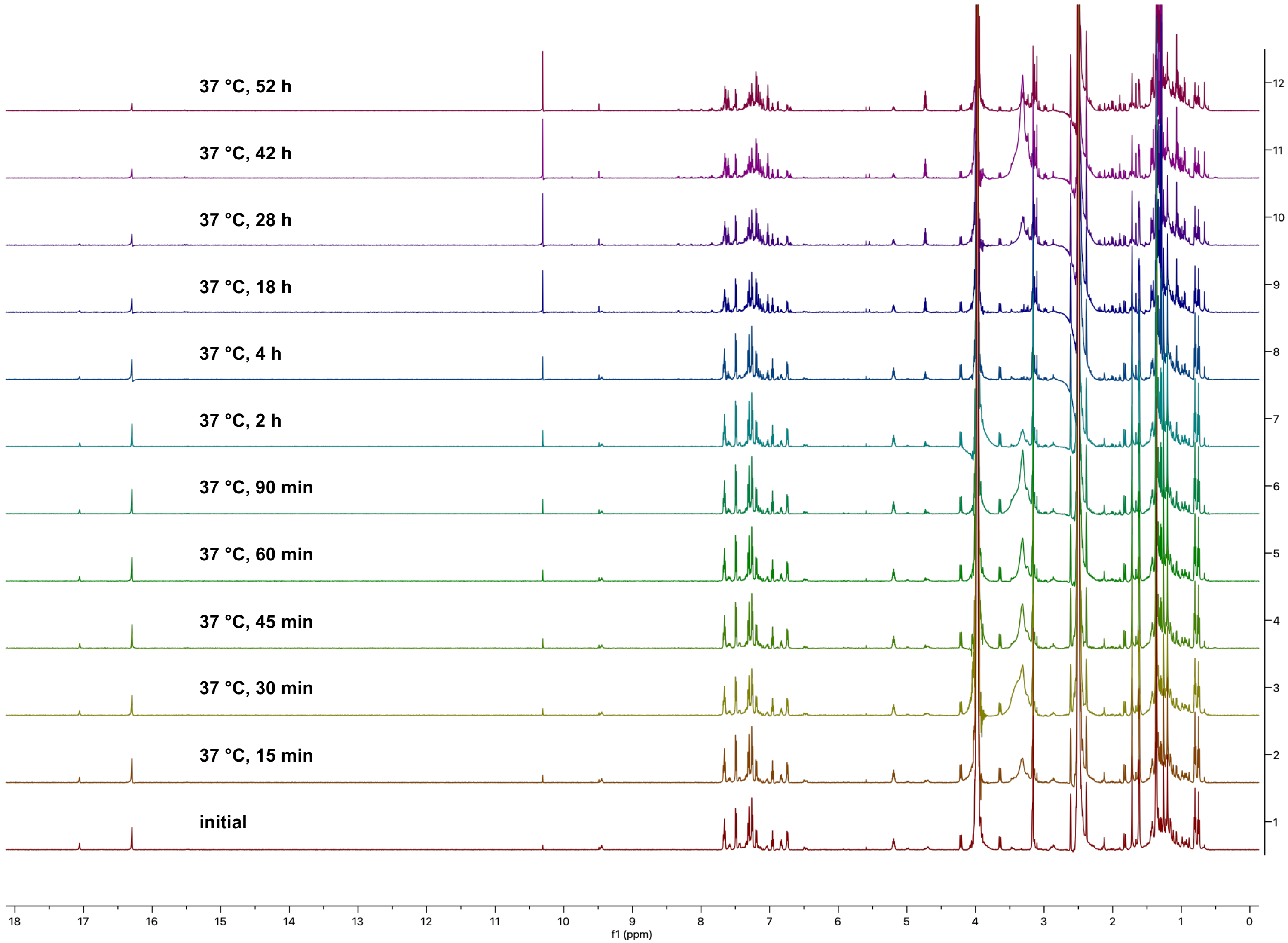

Figure S50. ^{1}H NMR spectrum (aliphatic region) of **[Ru(Et$_2$CAAC-5-Boc)]** in DMSO-d$_6$/D_2O (4:1) over time, normalized to the DMSO-d$_5$ signal (2.50 ppm).

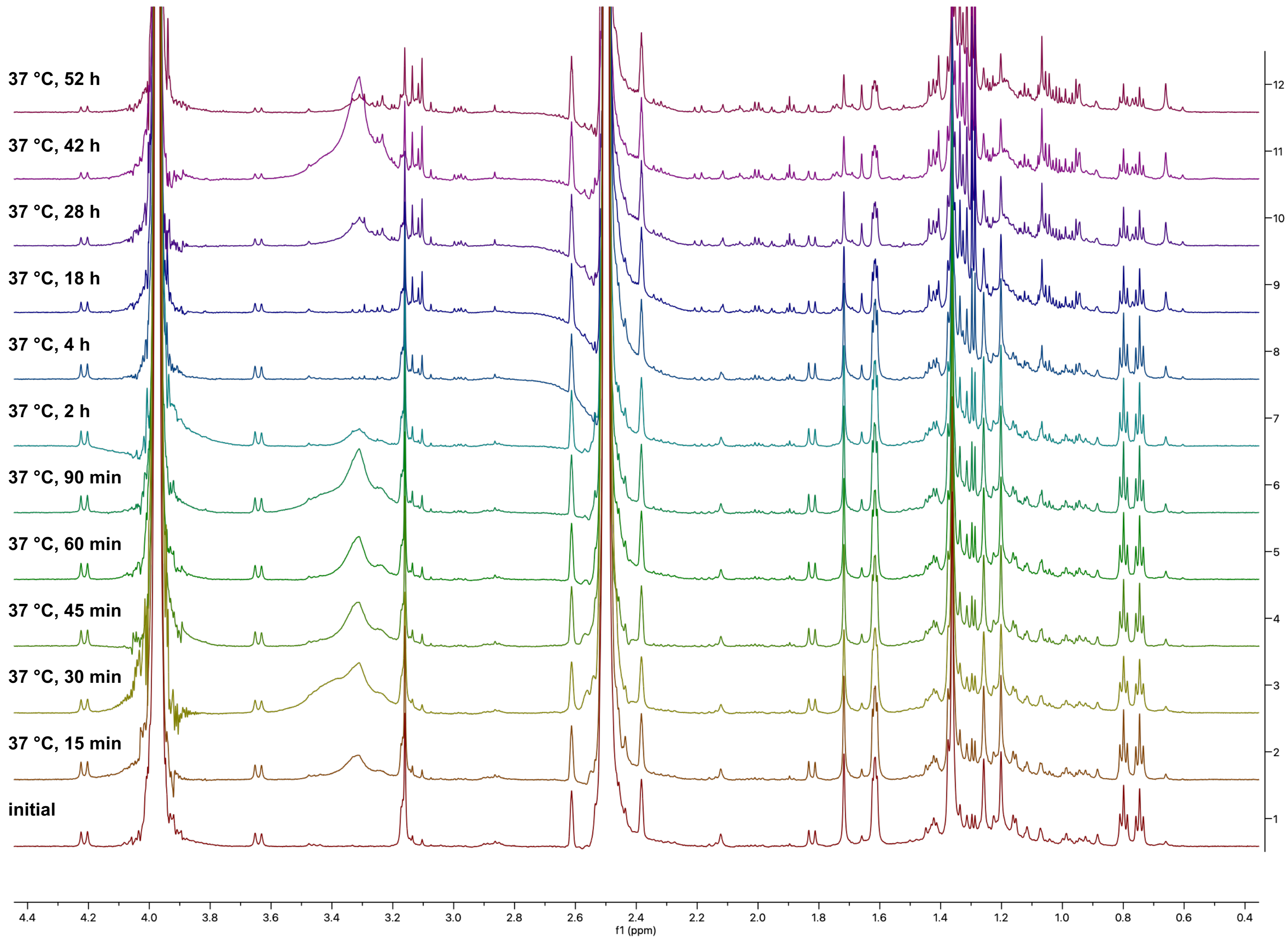

Figure S51. ^{1}H NMR spectrum (aromatic region) of **[Ru(Et$_2$CAAC-5-Boc)]** in DMSO-d$_6$/D$_2$O (4:1) over time, normalized to the DMSO-d$_5$ signal (2.50 ppm).

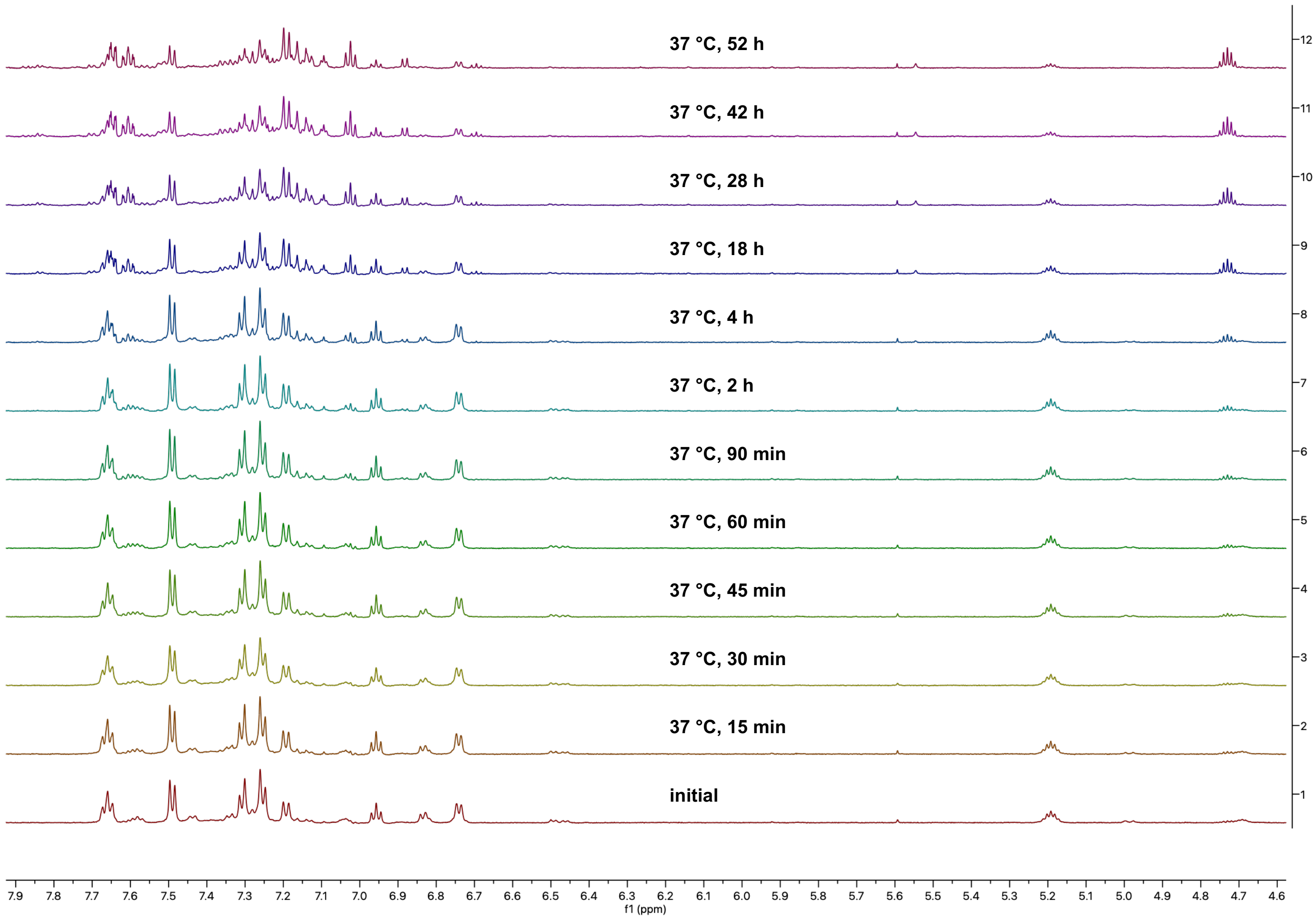

Figure S52. ^{1}H NMR spectrum (aldehyde, alkylidene region) of **[Ru(Et$_2$CAAC-5-Boc)]** in DMSO-d$_6$/D$_2$O (4:1) over time, normalized to the DMSO-d$_5$ signal (2.50 ppm).

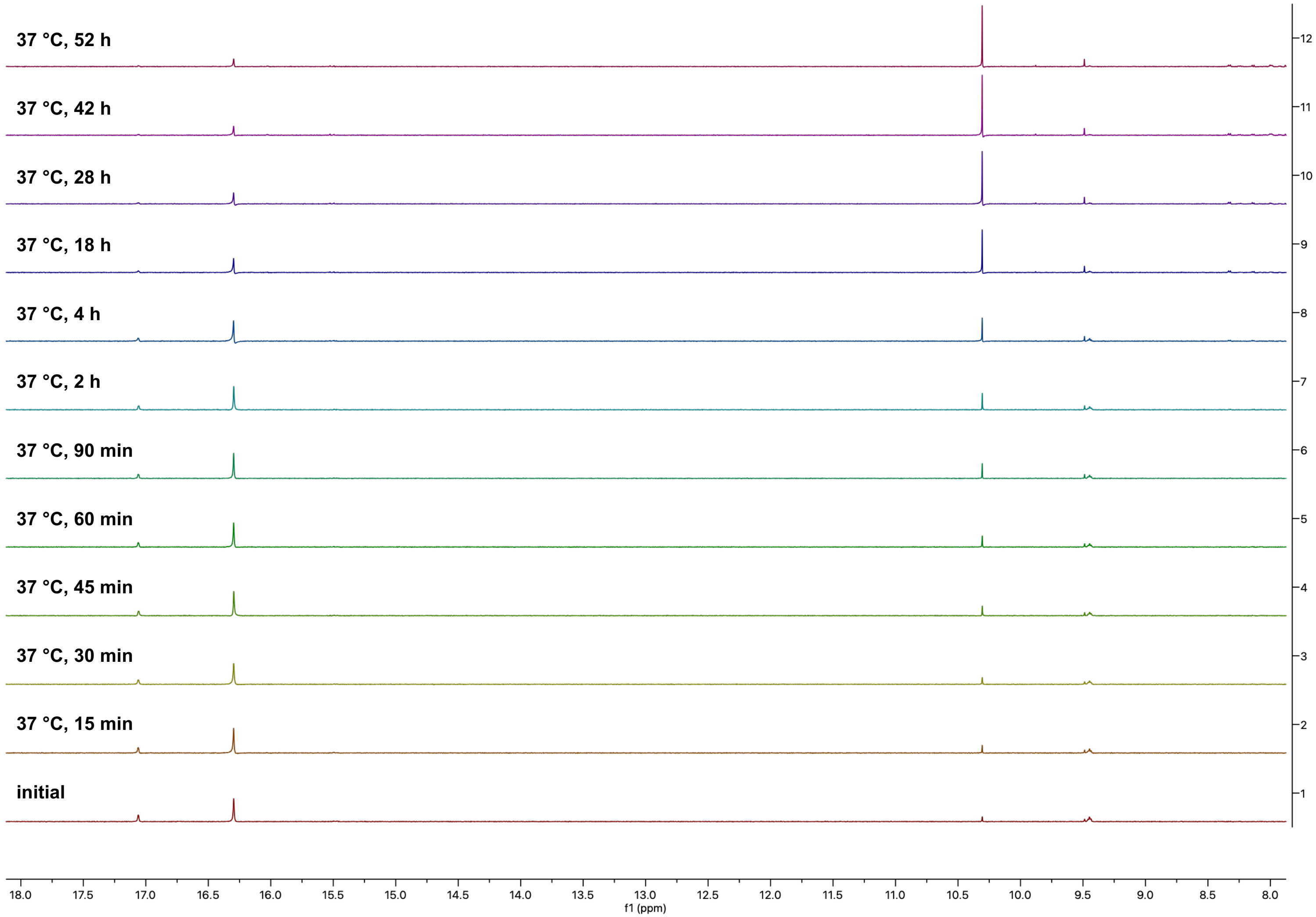

Figure S53. ^{1}H NMR spectrum of **[Ru(^{i}Pr$_2$CAAC-5-Boc)]** in DMSO-d$_6$/D$_2$O (4:1) over time, normalized to the DMSO-d$_5$ signal (2.50 ppm).

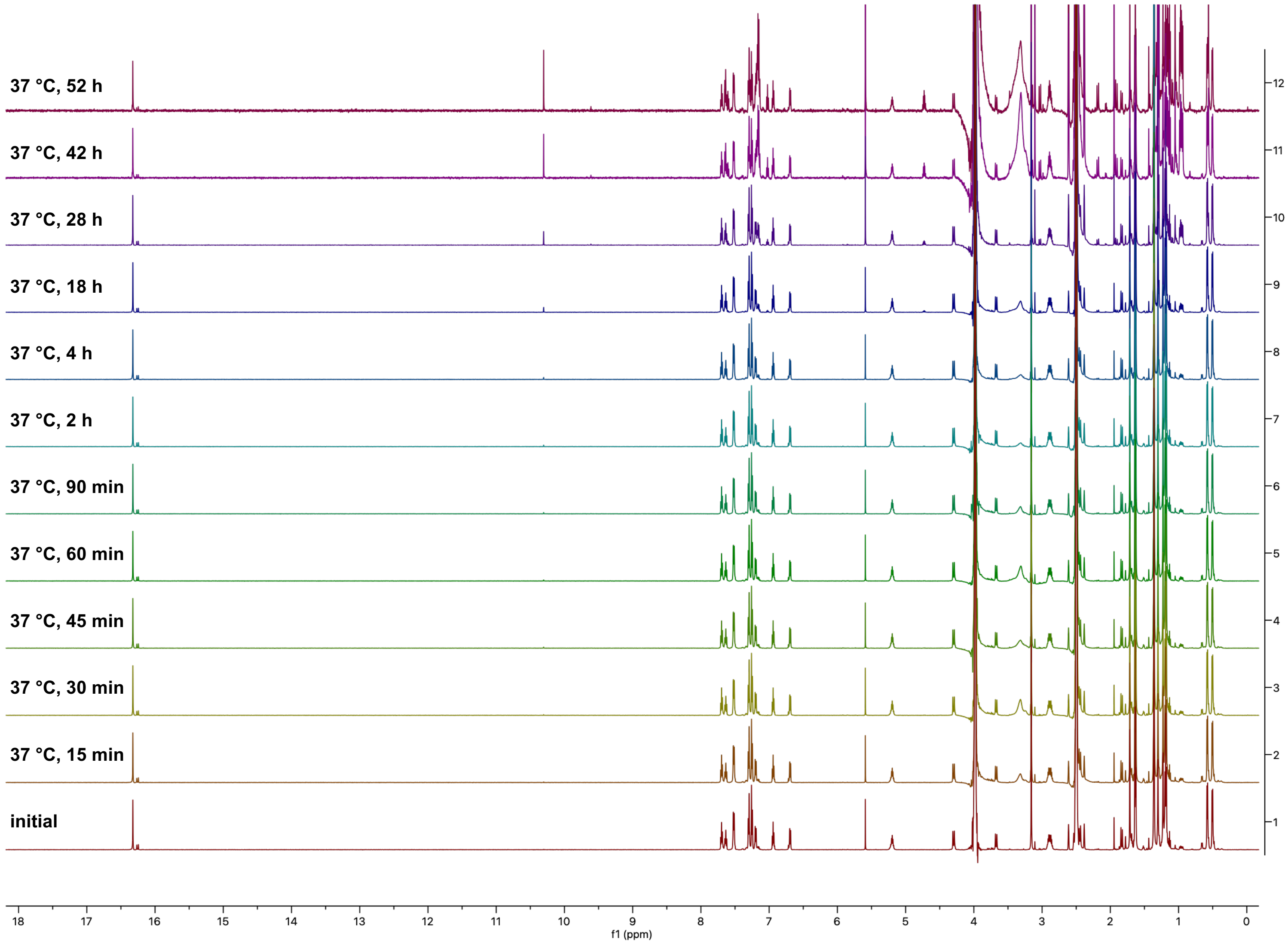

Figure S54. ^{1}H NMR spectrum (aliphatic region) of **[Ru(^{i}Pr$_2$CAAC-5-Boc)]** in DMSO-d$_6$/D$_2$O (4:1) over time, normalized to the DMSO-d$_5$ signal (2.50 ppm).

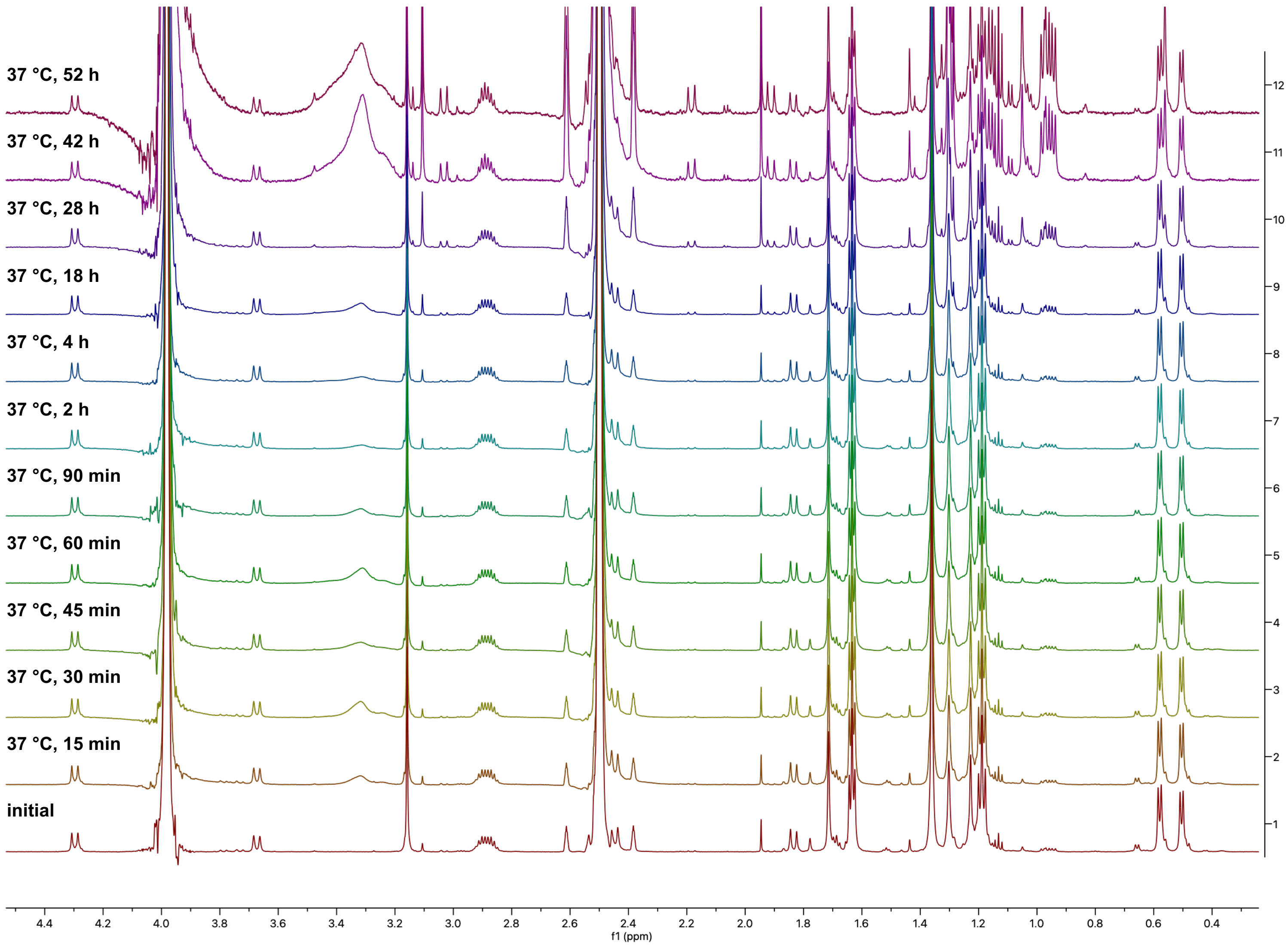

Figure S55. ^{1}H NMR spectrum (aromatic region) of **[Ru(^{i}Pr$_2$CAAC-5-Boc)]** in DMSO-d$_6$/D$_2$O (4:1) over time, normalized to the DMSO-d$_5$ signal (2.50 ppm).

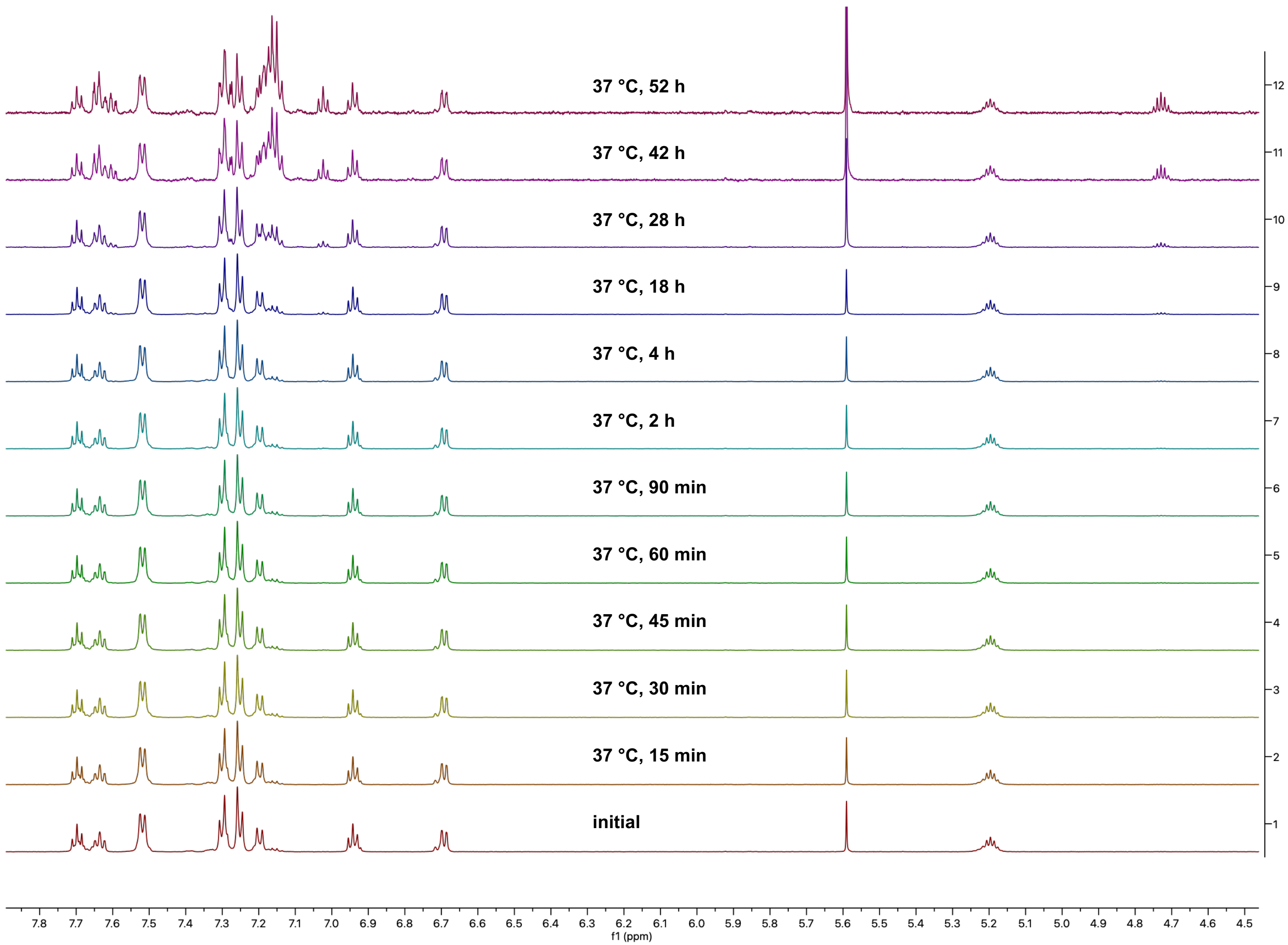

Figure S56. ^{1}H NMR spectrum (aldehyde, alkylidene region) of **[Ru(^{i}Pr$_2$CAAC-5-Boc)]** in DMSO-d$_6$/D$_2$O (4:1) over time, normalized to the DMSO-d$_5$ signal (2.50 ppm).

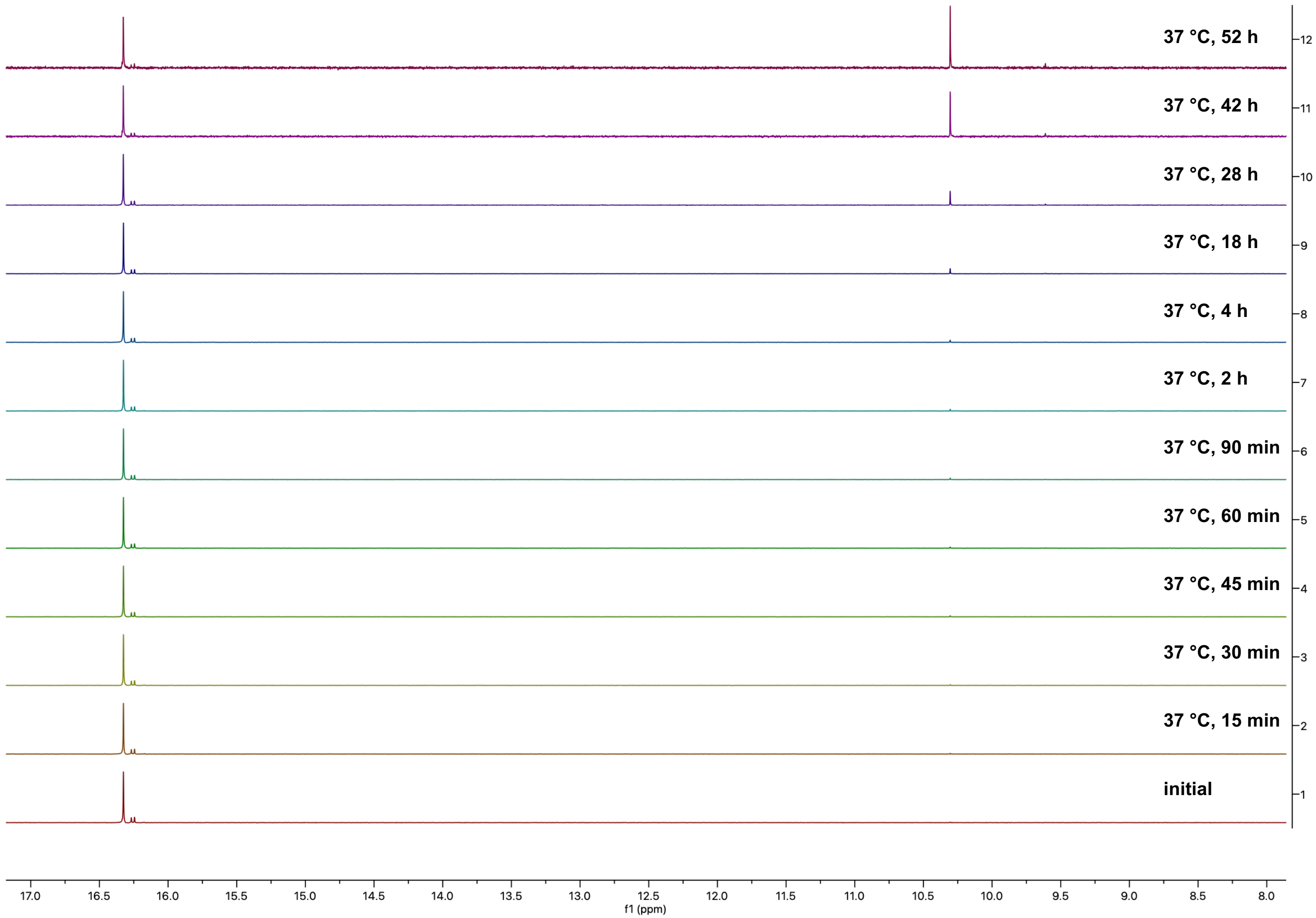

Figure S57. ^{1}H NMR spectrum of **[Ru(Et$_2$CAAC-5-Boc)]** + Ts-diolefin **1** (10 eq.) in DMSO-d$_6$/D$_2$O (4:1) over time, normalized to the DMSO-d$_5$ signal (2.50 ppm).

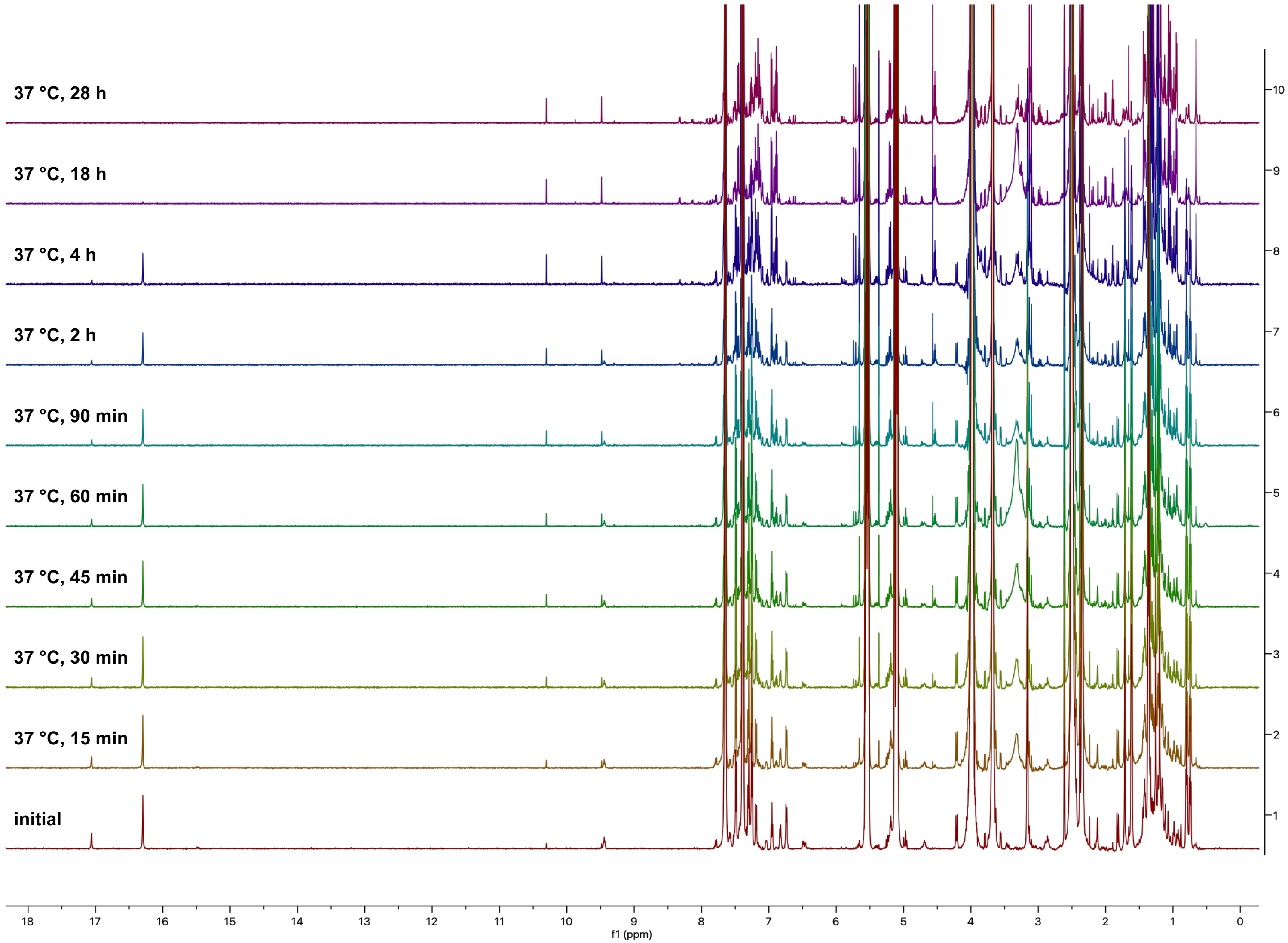

Figure S58. ^{1}H NMR spectrum (aliphatic region) of **[Ru(Et$_2$CAAC-5-Boc)]** + Ts-diolefin **1** (10 eq.) in DMSO-d$_6$/D$_2$O (4:1) over time, normalized to the DMSO-d$_5$ signal (2.50 ppm).

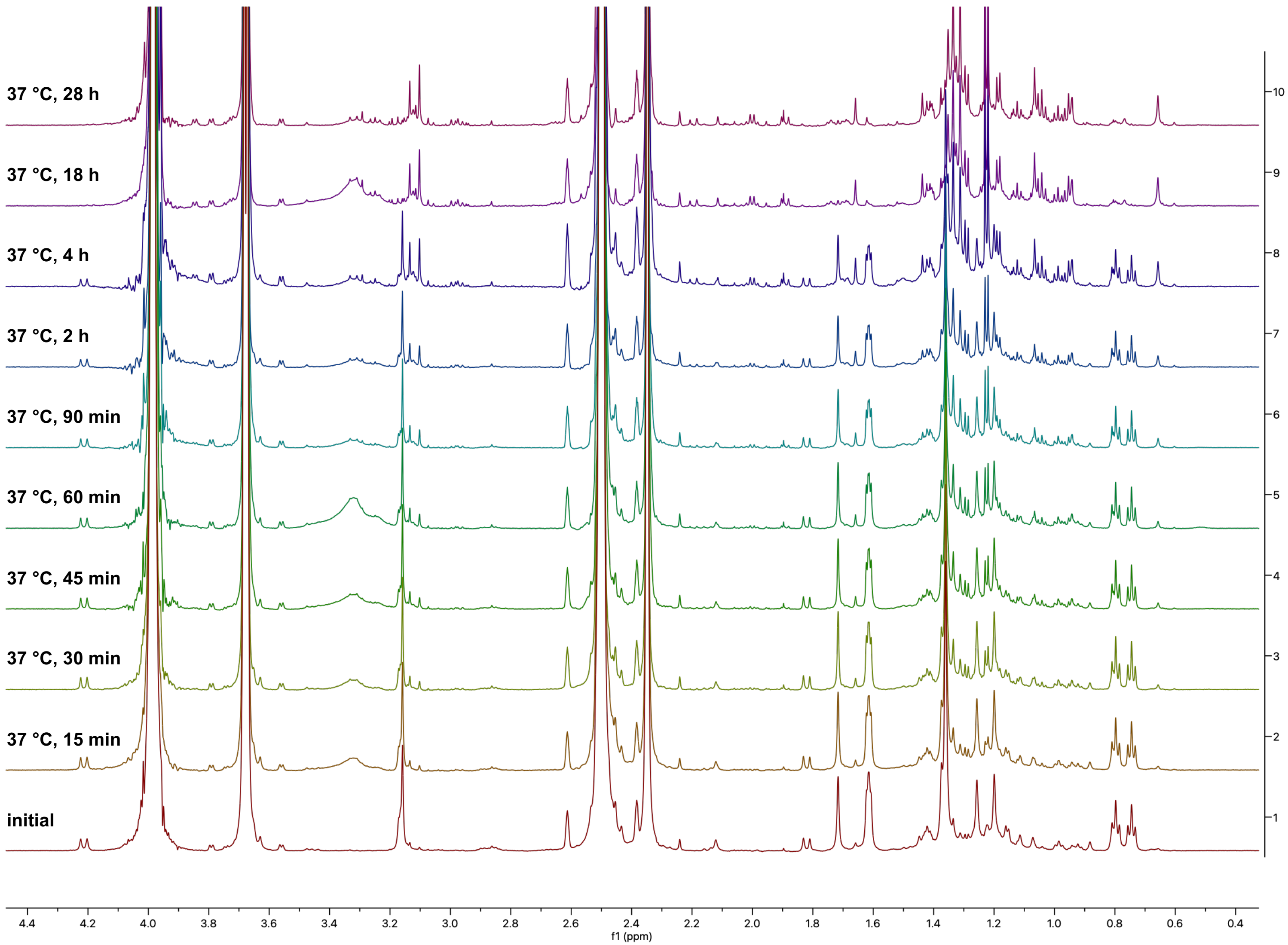

Figure S59. ^{1}H NMR spectrum (aromatic region) of **[Ru(Et$_2$CAAC-5-Boc)]** + Ts-diolefin **1** (10 eq.) in DMSO-d$_6$/D_2O (4:1) over time, normalized to the DMSO-d$_5$ signal (2.50 ppm).

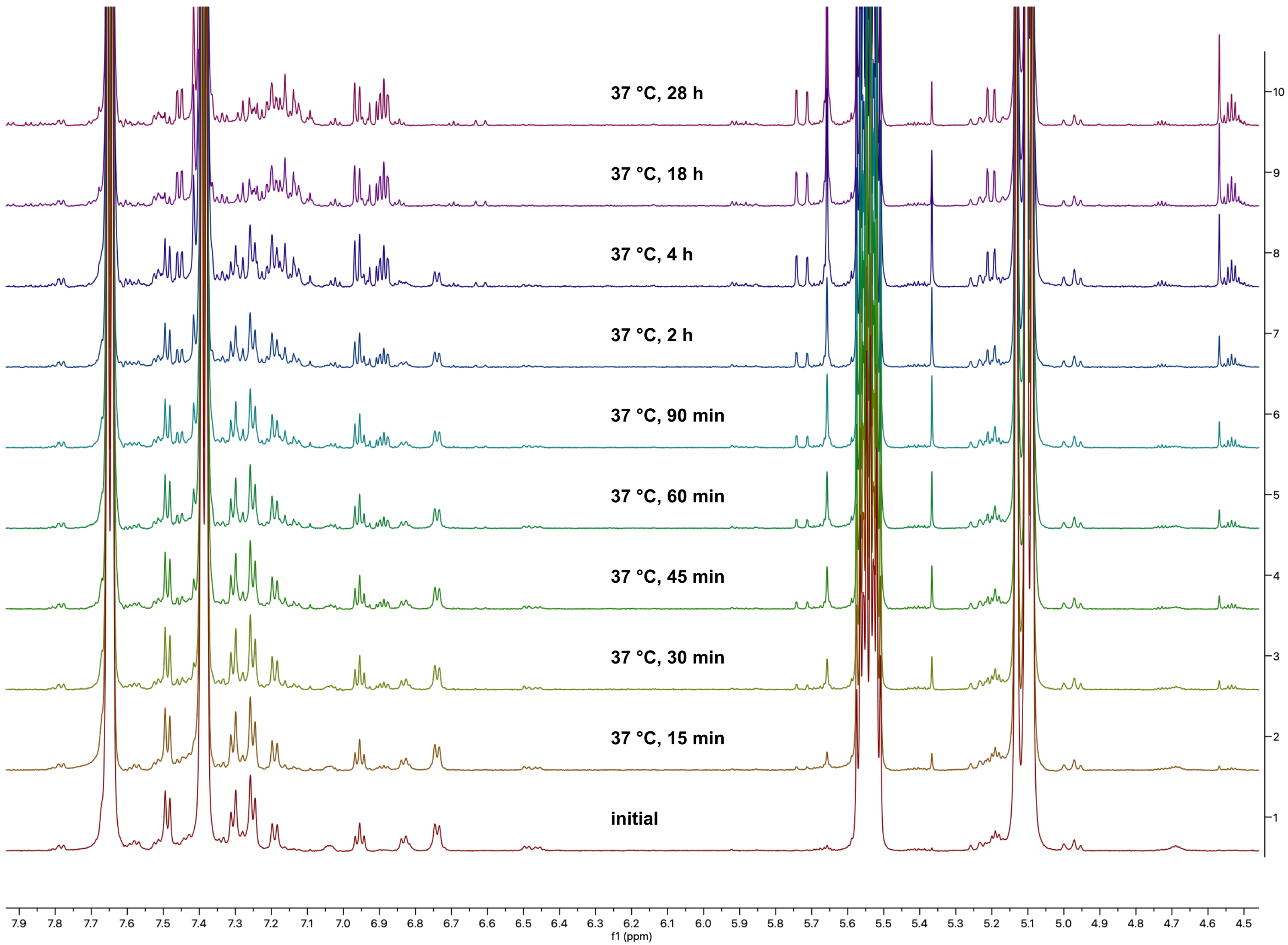

Figure S60. ^{1}H NMR spectrum (aldehyde, alkylidene region) of **[Ru(Et$_2$CAAC-5-Boc)]** + Ts-diolefin **1** (10 eq.) in DMSO-d_6/D_2O (4:1) over time, normalized to the DMSO-d_5 signal (2.50 ppm).

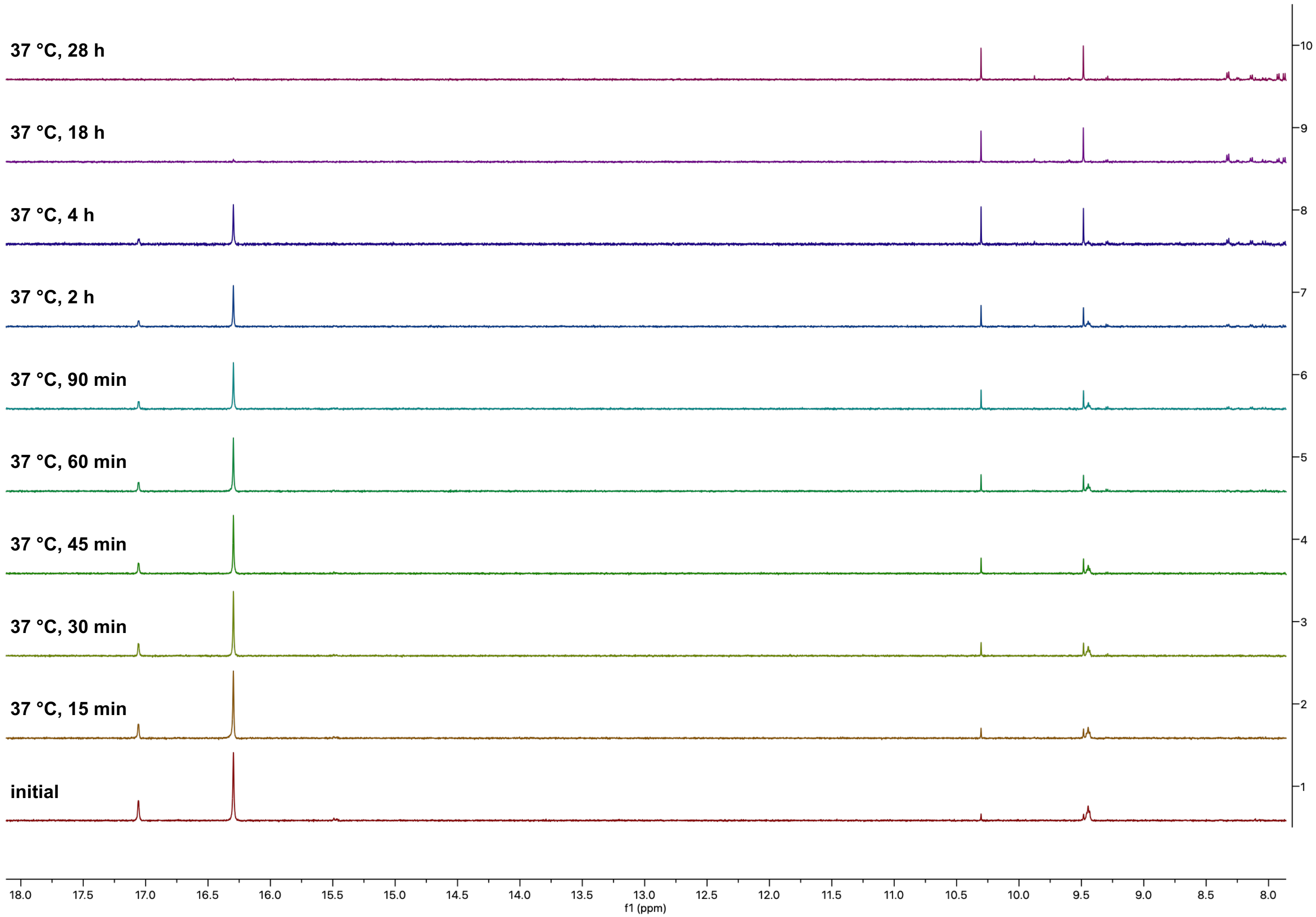

Figure S61. ^{1}H NMR spectrum of **[Ru(^{i}Pr$_2$CAAC-5-Boc)]** + Ts-diolefin **1** (10 eq.) in DMSO-d_6/D_2O (4:1) over time, normalized to the DMSO-d_5 signal (2.50 ppm).

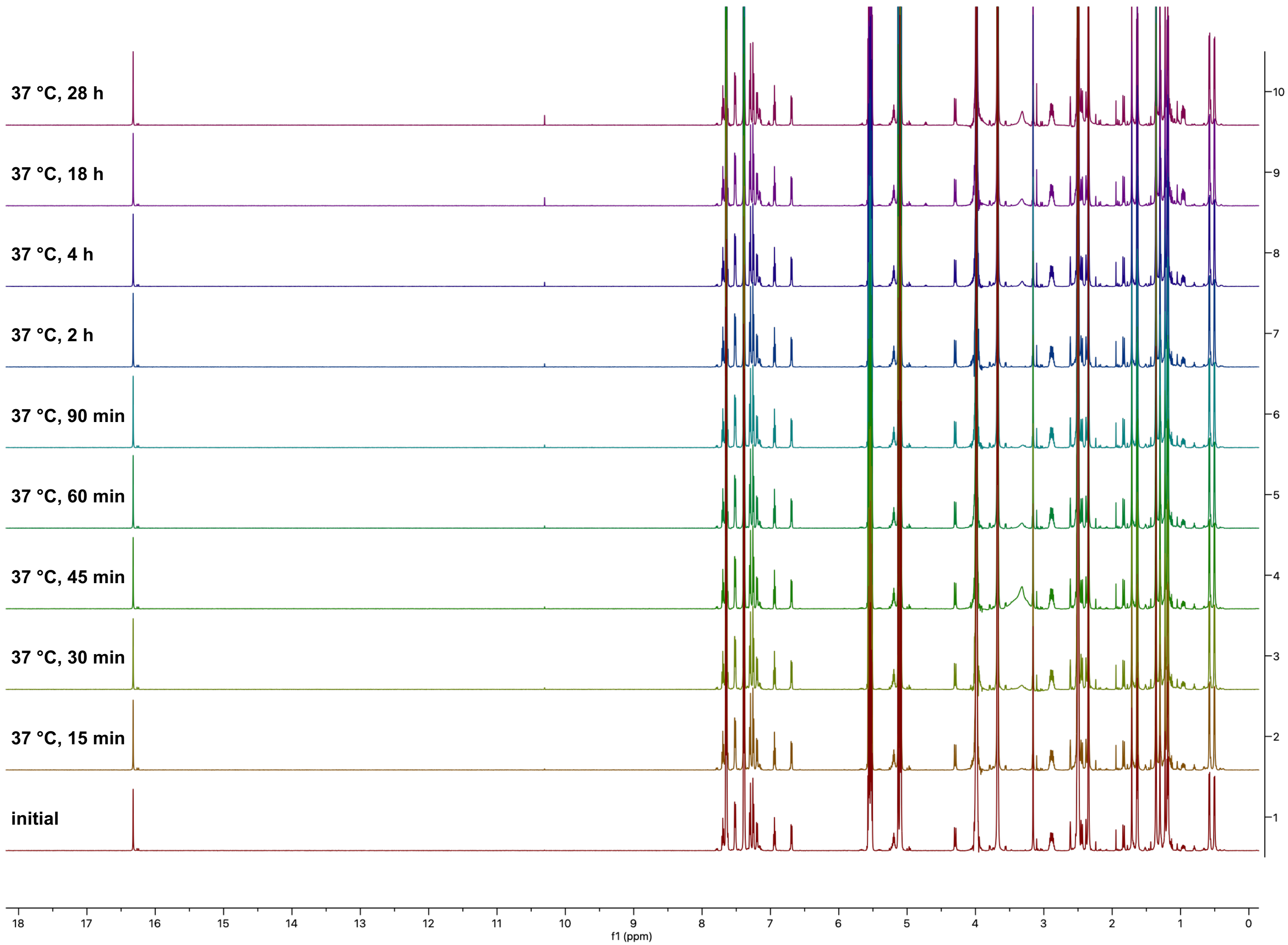

Figure S62. ^{1}H NMR spectrum (aliphatic region) of **[Ru(^{i}Pr$_2$CAAC-5-Boc)]** + Ts-diolefin **1** (10 eq.) in DMSO-d_6/D_2O (4:1) over time, normalized to the DMSO-d_5 signal (2.50 ppm).

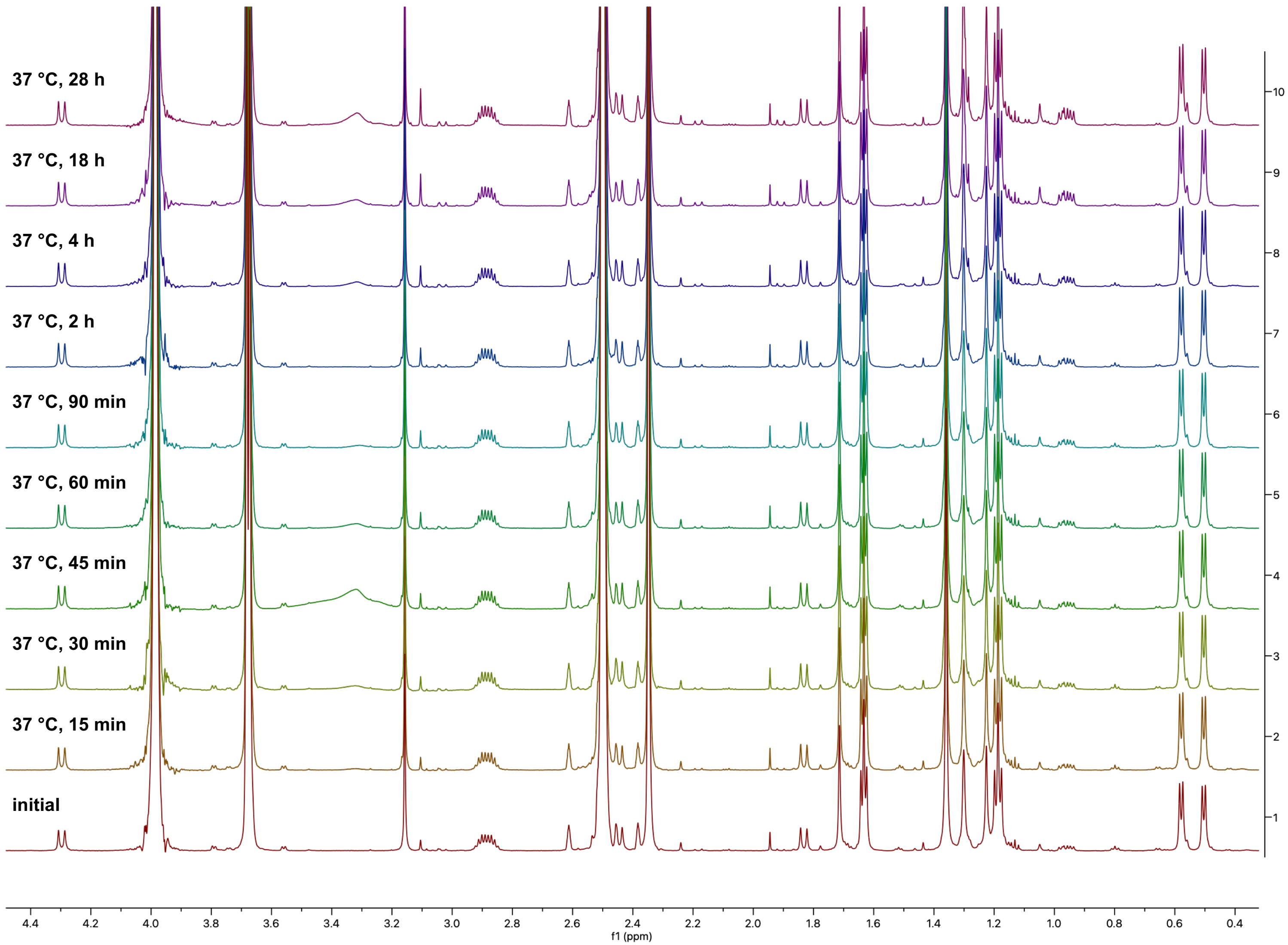

Figure S63. ¹H NMR spectrum (aromatic region) of **[Ru(ⁱPr₂CAAC-5-Boc)]** + Ts-diolefin **1** (10 eq.) in DMSO-d₆/D₂O (4:1) over time, normalized to the DMSO-d₅ signal (2.50 ppm).

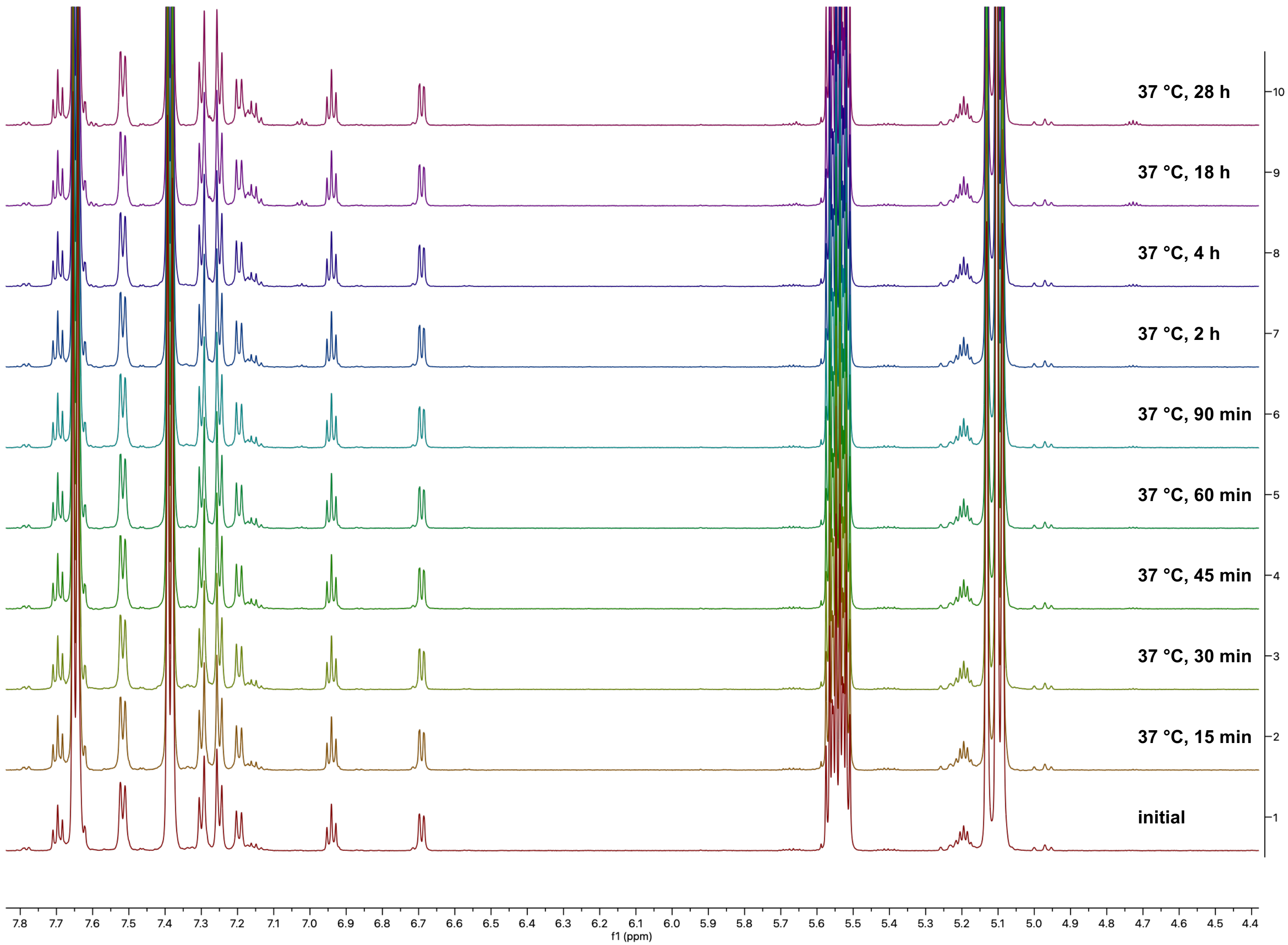

Figure S64. ^{1}H NMR spectrum (aldehyde, alkylidene region) of **[Ru(^{i}Pr$_2$CAAC-5-Boc)]** + Ts-diolefin **1** (10 eq.) in DMSO-d_6/D_2O (4:1) over time, normalized to the DMSO-d_5 signal (2.50 ppm).

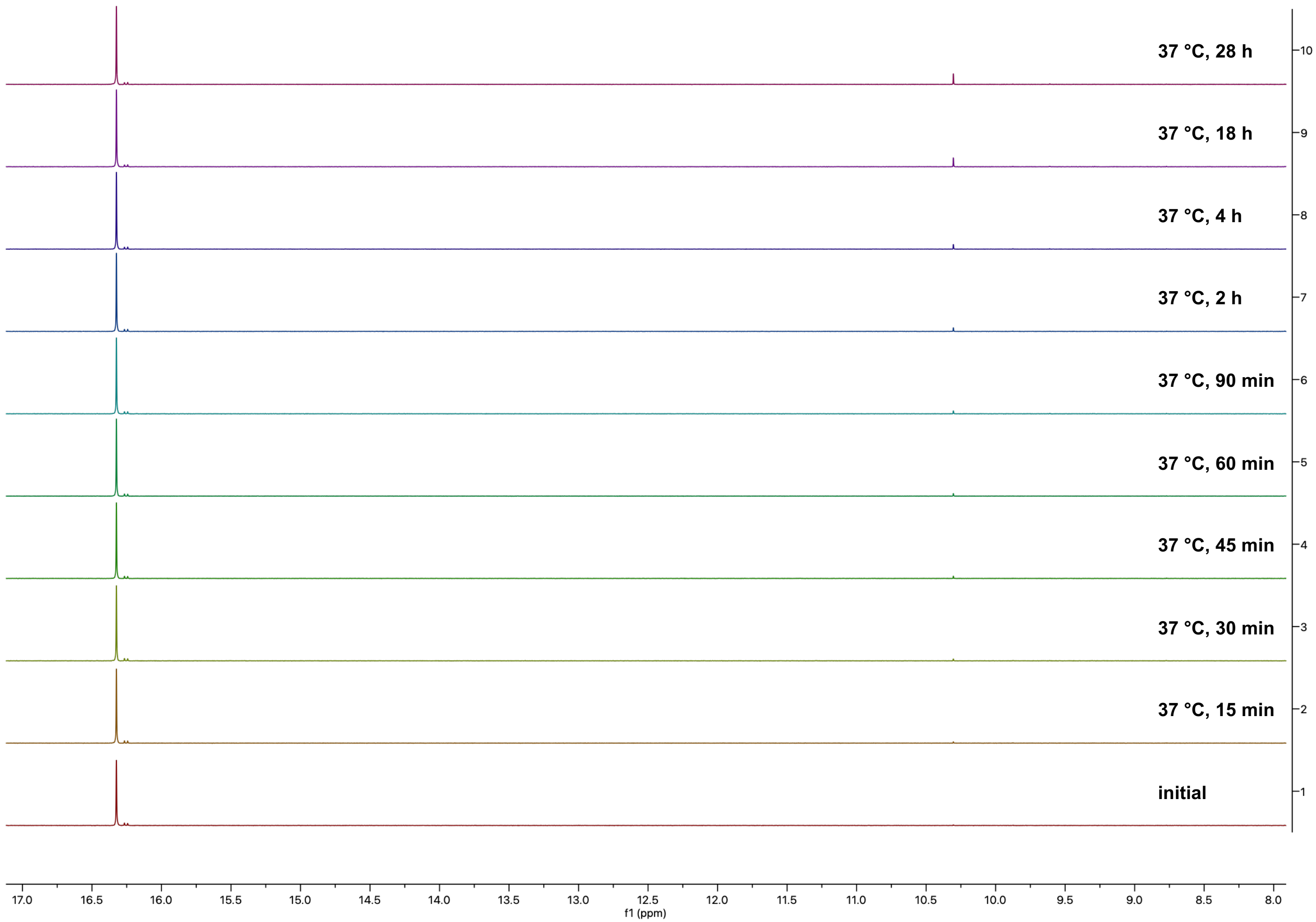

HRMS Spectra

Figure S65. HRMS-ESI$^+$ Spectrum of enamine **S8** in MeCN.

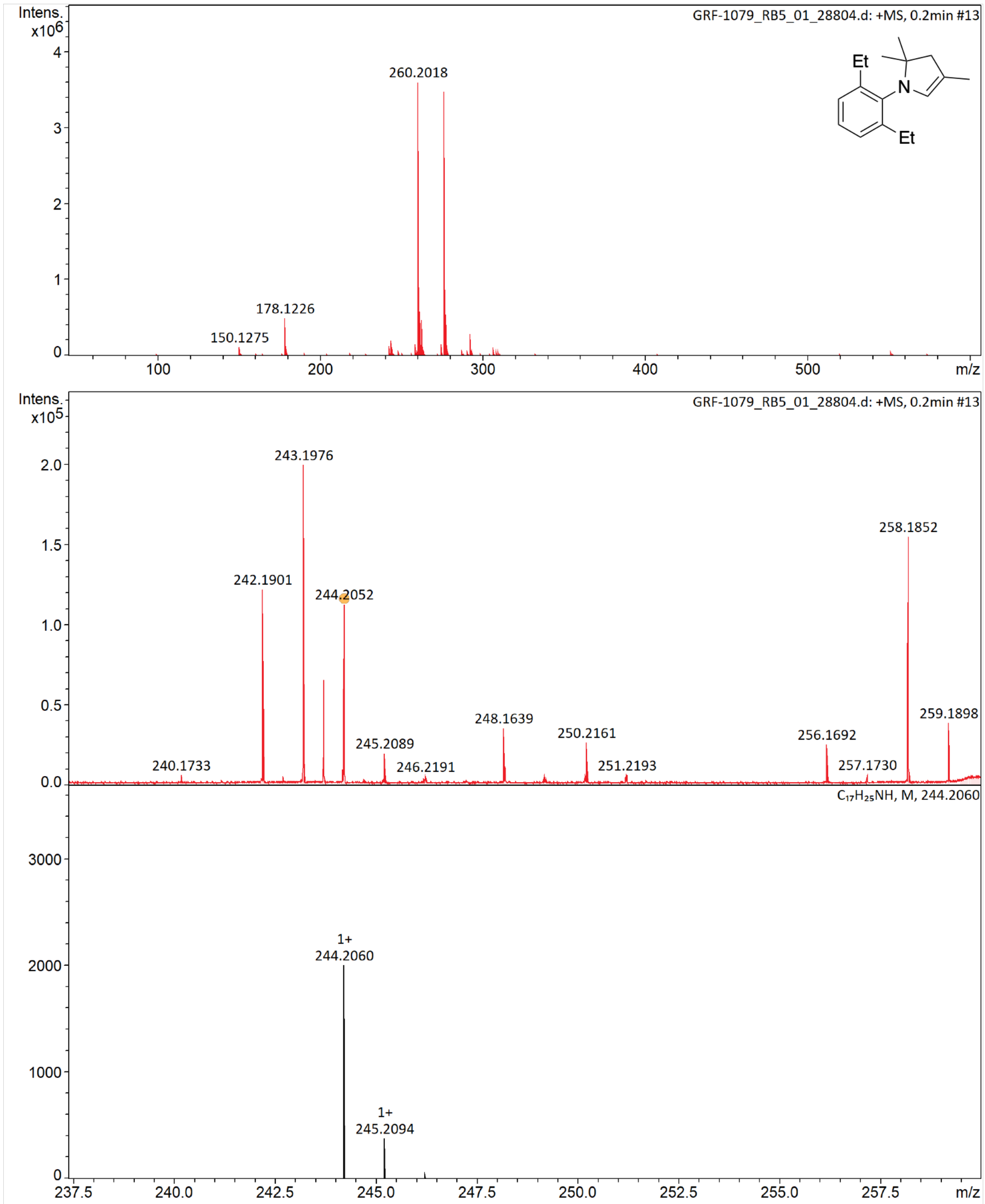

Figure S66. HRMS-ESI+ Spectrum of **Et2CAAC-5-H BF4 S9** in MeCN.

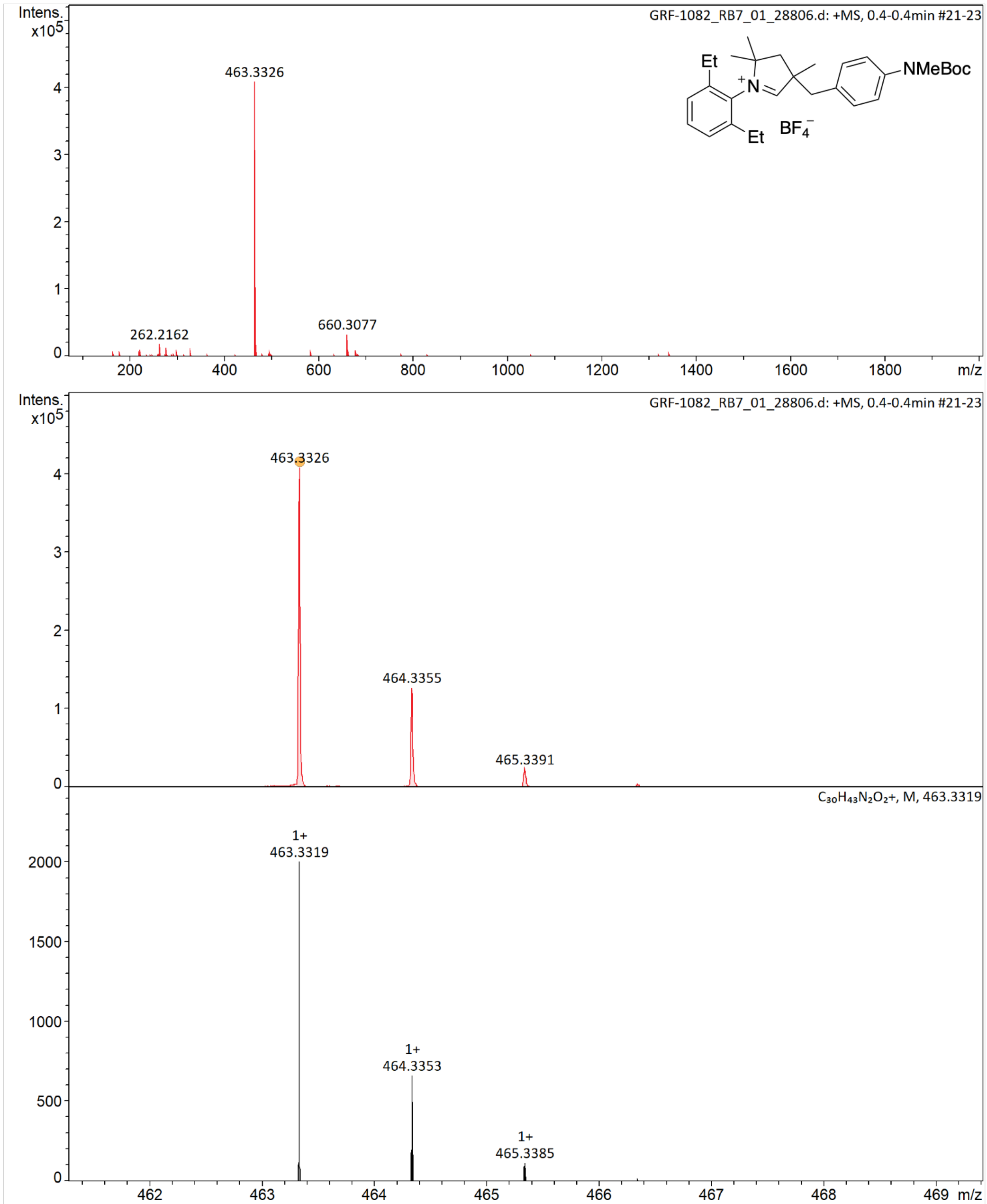

Figure S67. HRMS-ESI$^+$ Spectrum of **[Ru(Et$_2$CAAC-5-Boc)] S10** in MeCN.

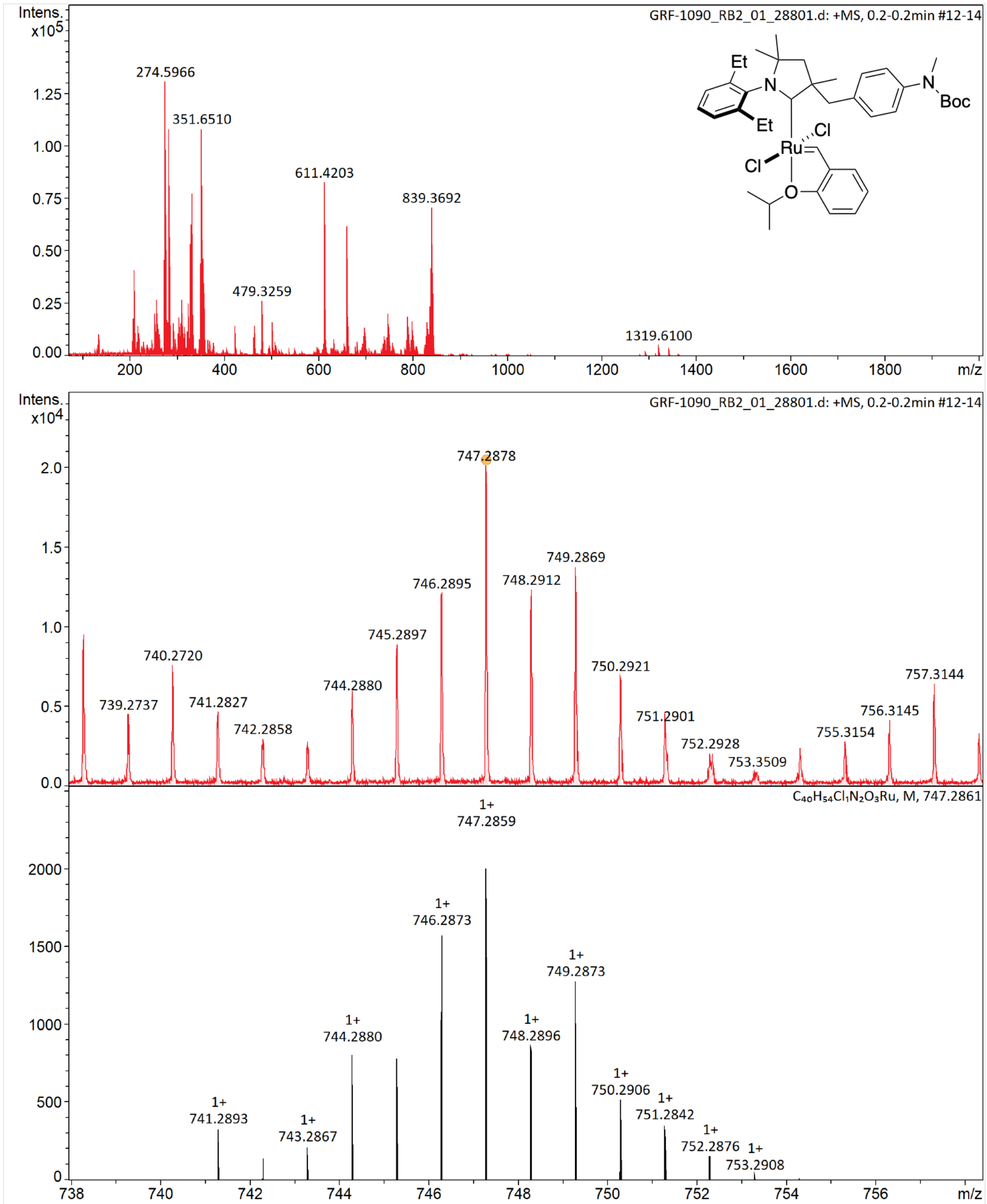

Figure S68. HRMS-ESI$^+$ Spectrum of ^{i}**Pr$_2$CAAC-5-H BF$_4$ S15** in MeCN.

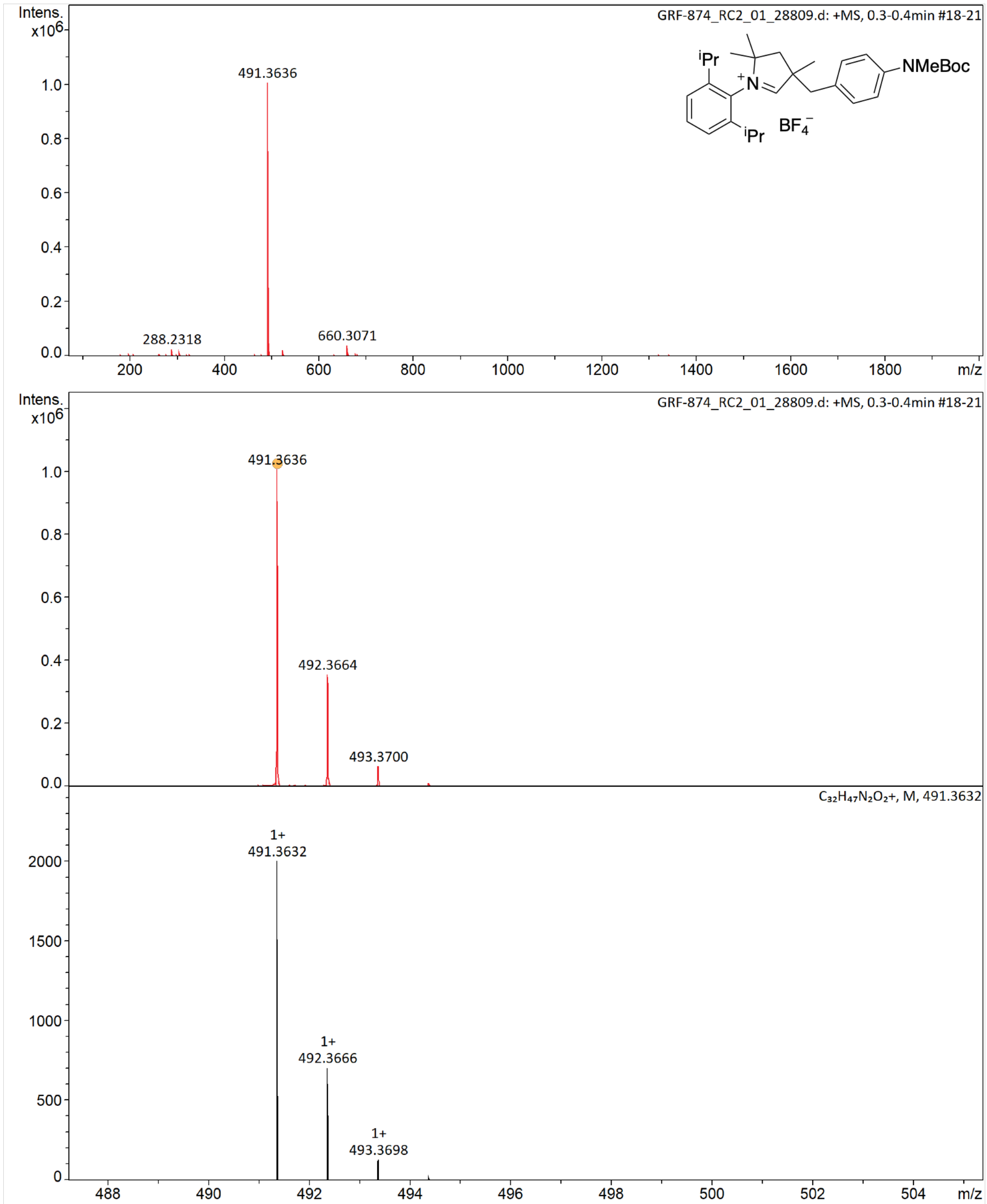

Figure S69. HRMS-ESI+ Spectrum of **[Ru(iPr2CAAC-5-Boc)] S16** in MeCN.

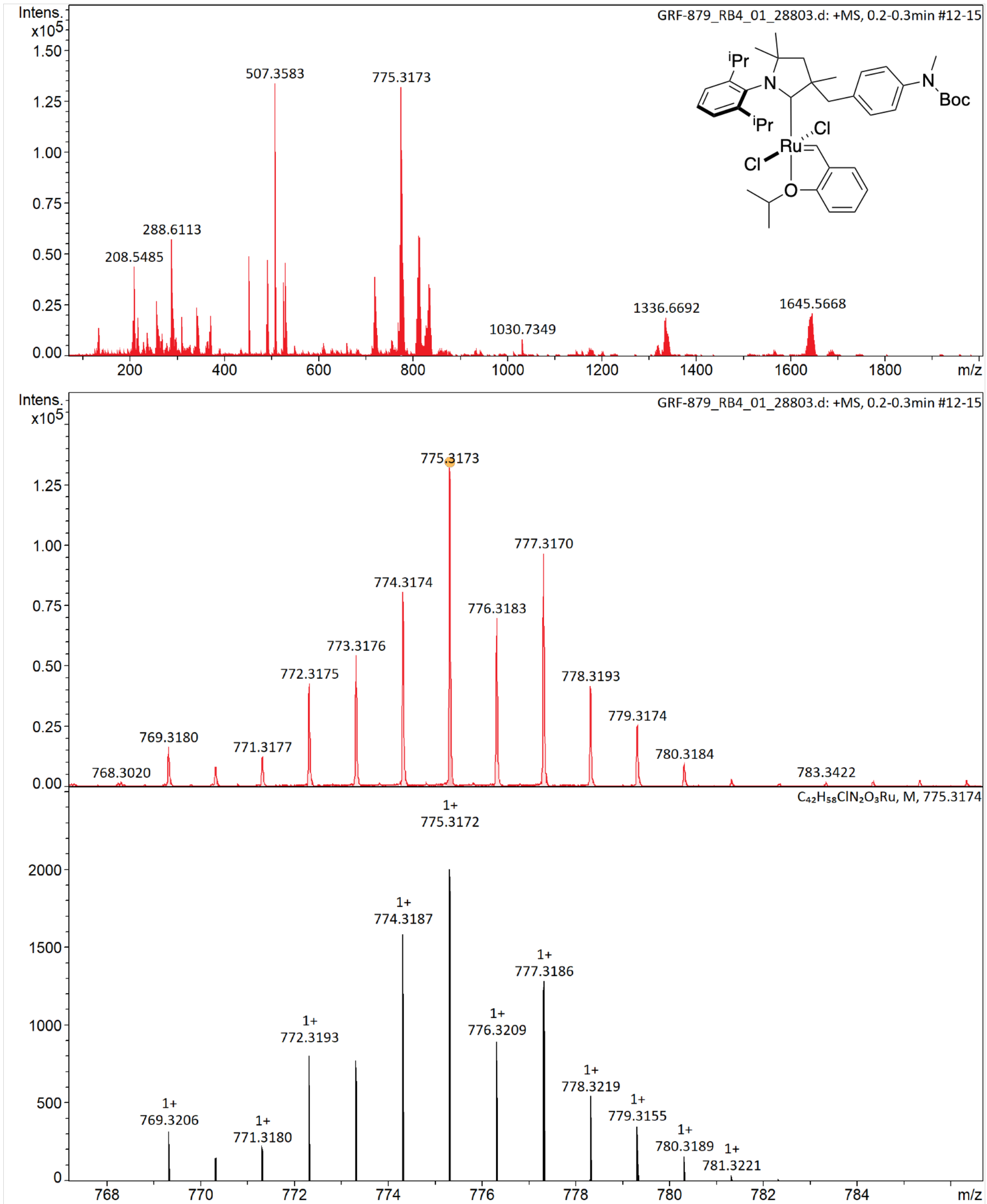

Figure S70. HRMS-ESI+ Spectrum of **[Ru(^{i}Pr$_2$CAAC-5)] S17** in MeCN.

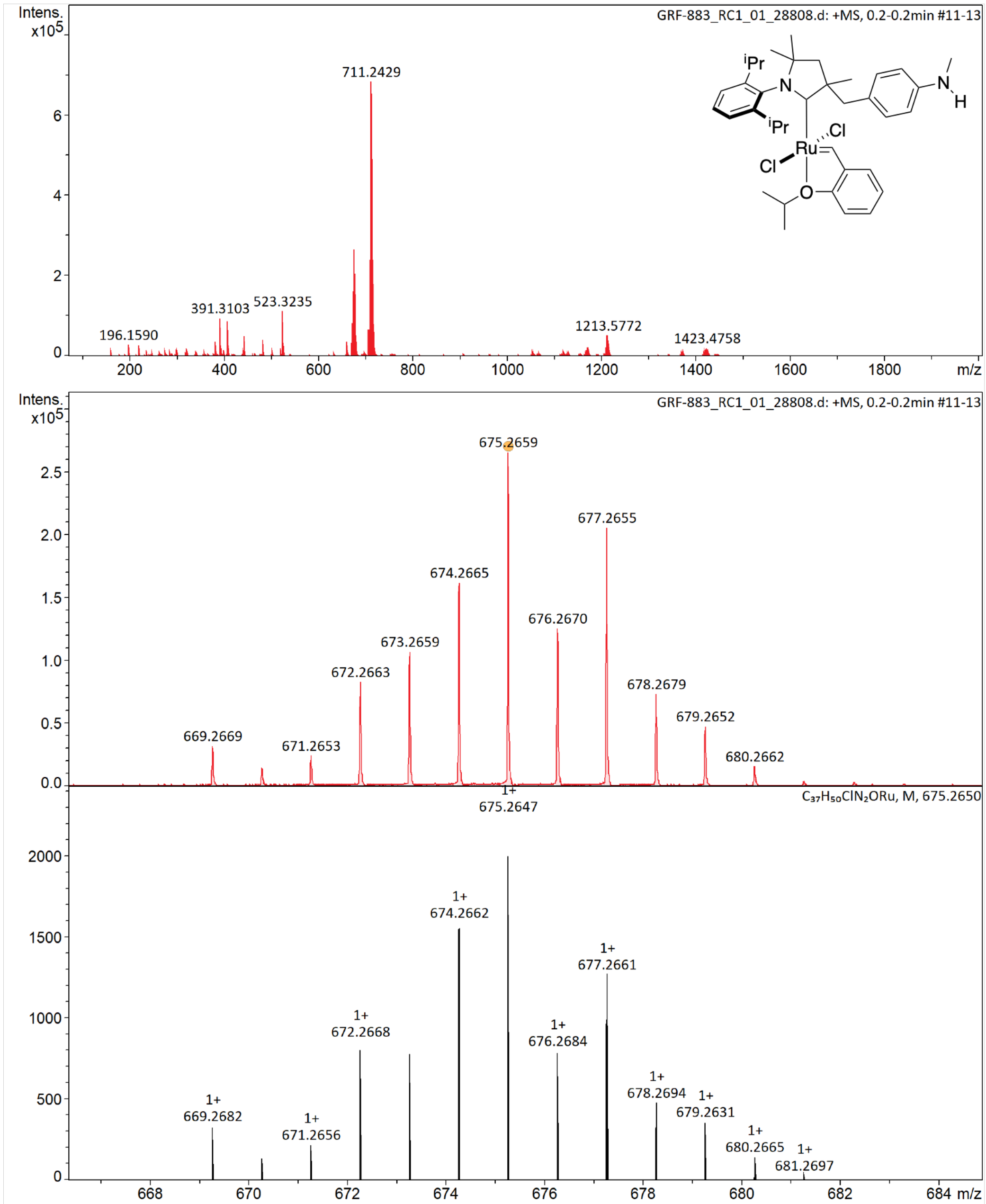

Figure S71. HRMS-ESI+ Spectrum of **[Ru(iPr2CAAC-5-SO2NH2)] S19** in MeCN.

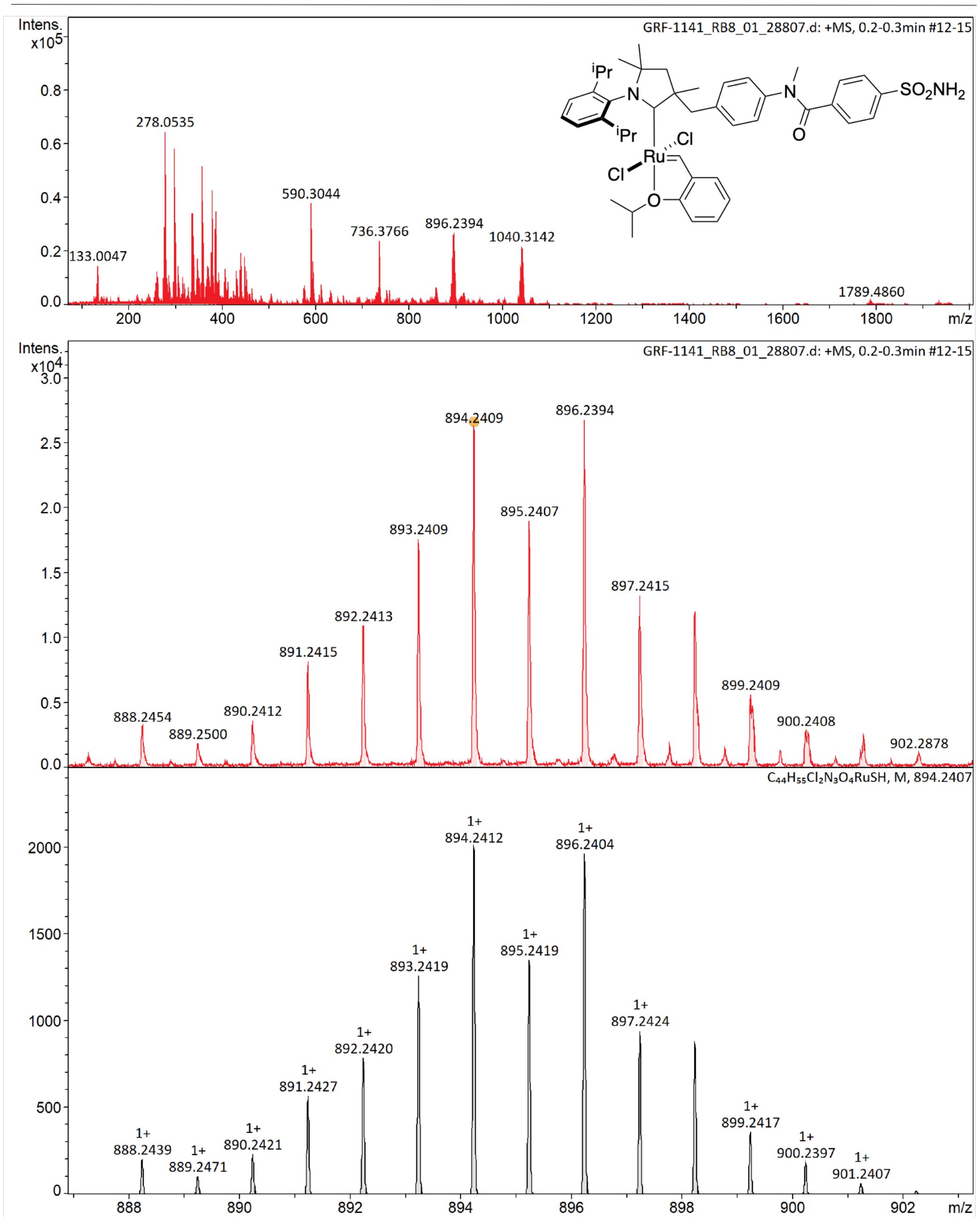

Figure S72. HRMS-ESI$^+$ Spectrum of **[Ru(^{i}Pr$_2$CAAC-5-N$_3$)] S21** in MeCN.

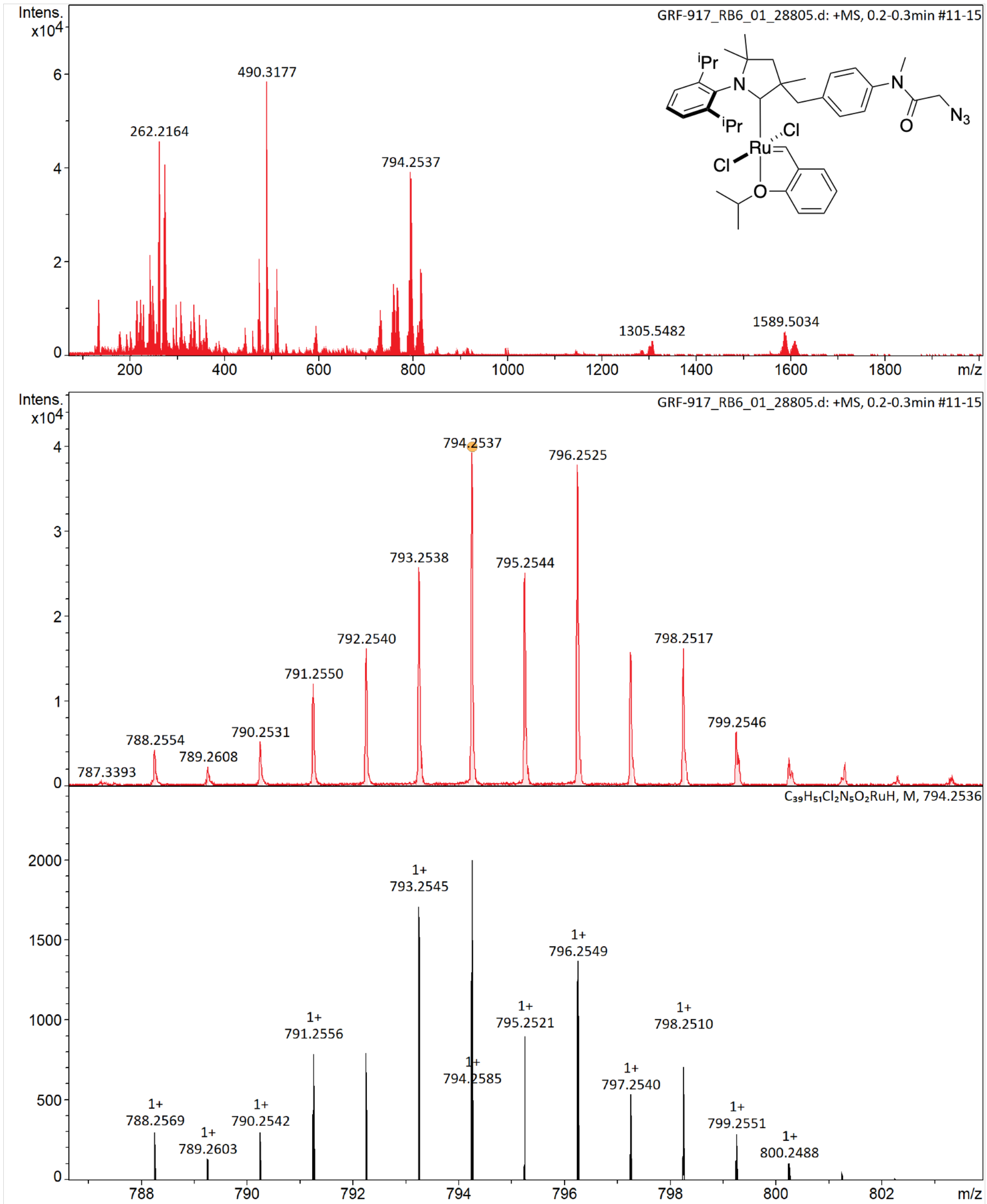

Figure S73. HRMS-ESI$^+$ Spectrum of **[Ru(^{i}Pr$_2$CAAC-5-Br)] S22** in MeCN.

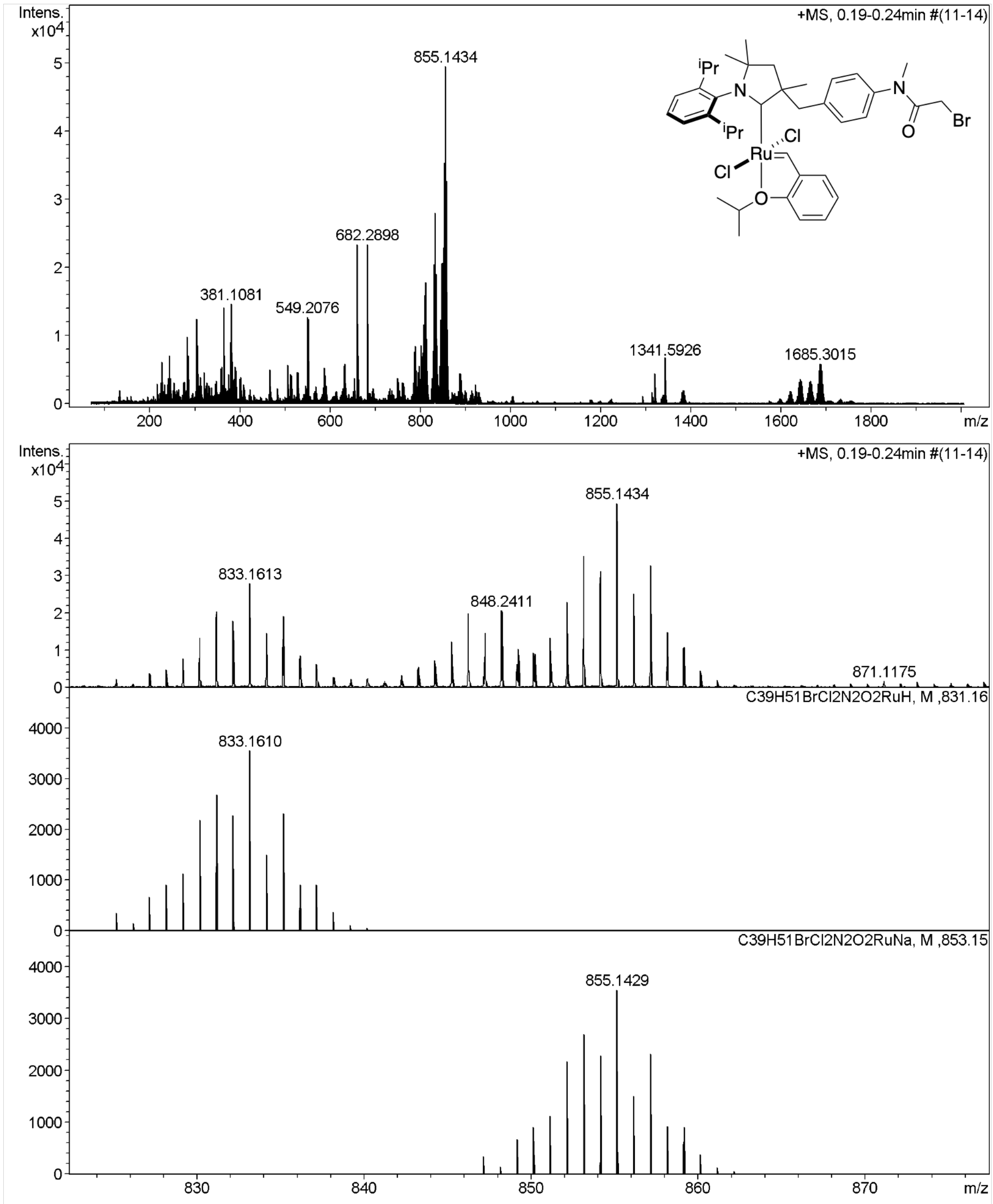

Figure S74. HRMS-ESI+ Spectrum of **SS01 alkyne S25** in MeCN.

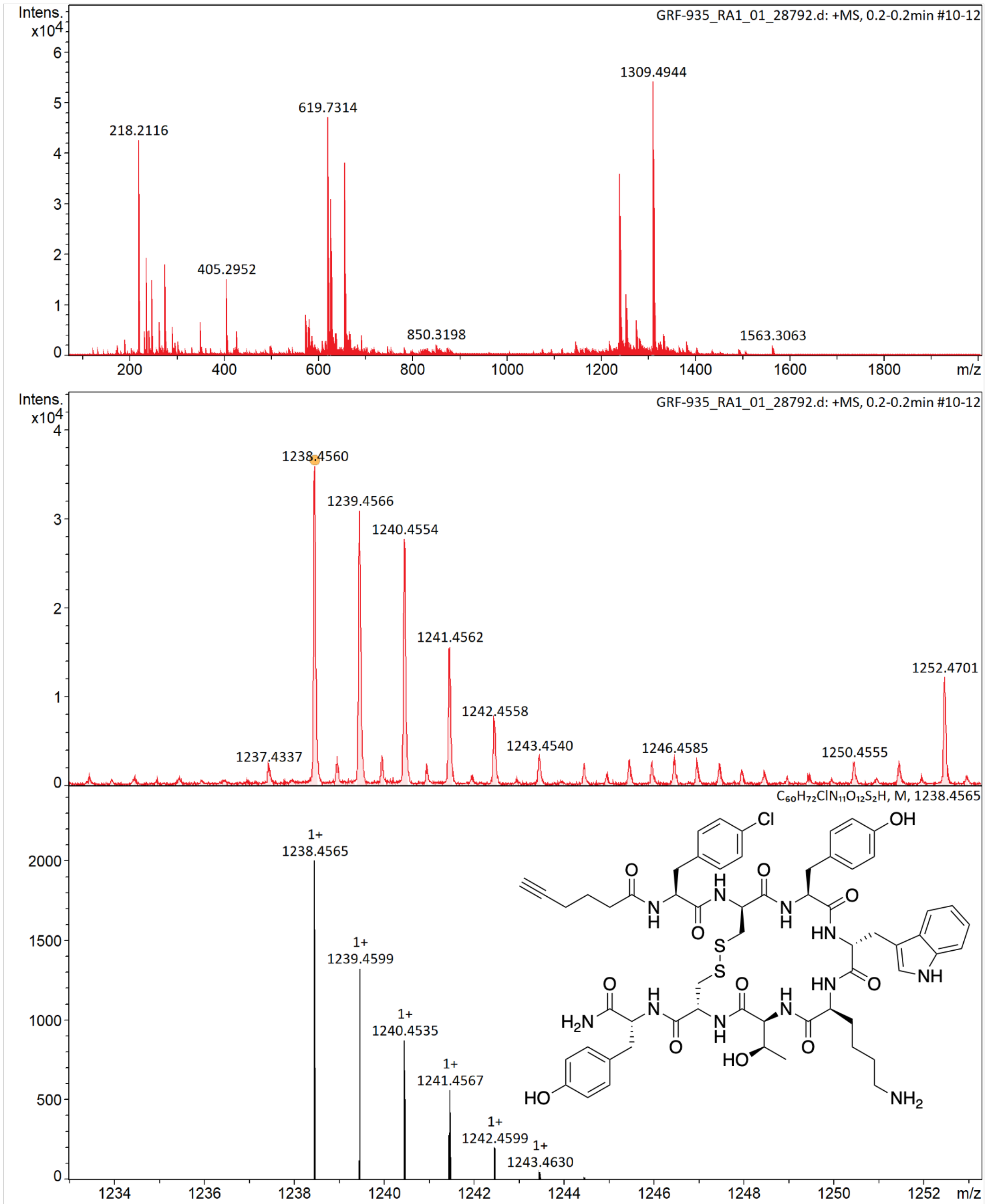

Figure S75. HRMS-ESI⁺ Spectrum of **[Ru(^{i}Pr$_2$CAAC-5)] · SS01** in MeOH.

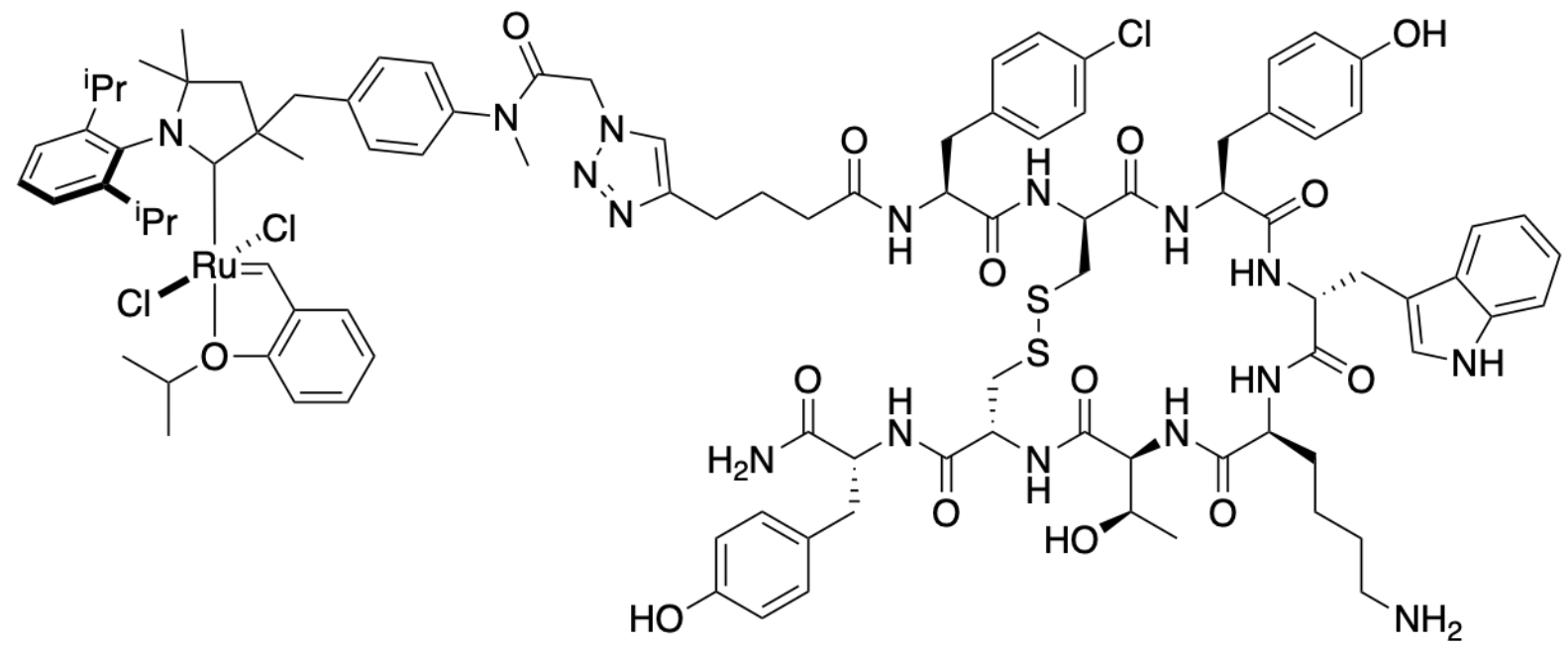

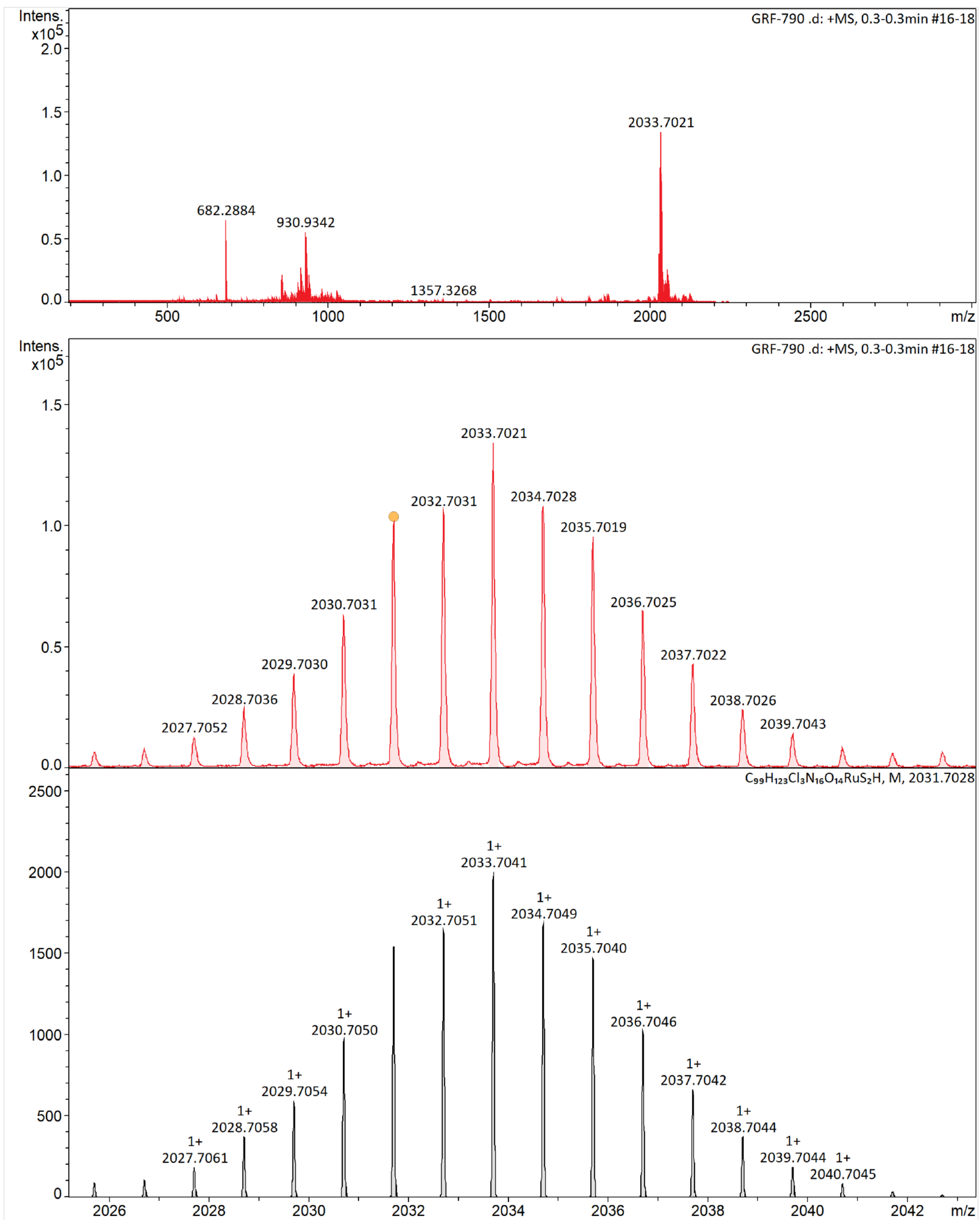

Figure S76. HRMS-ESI+ Spectrum of **MBP-Ubi K6C** in PBS buffer (50 mM, pH 7.2).

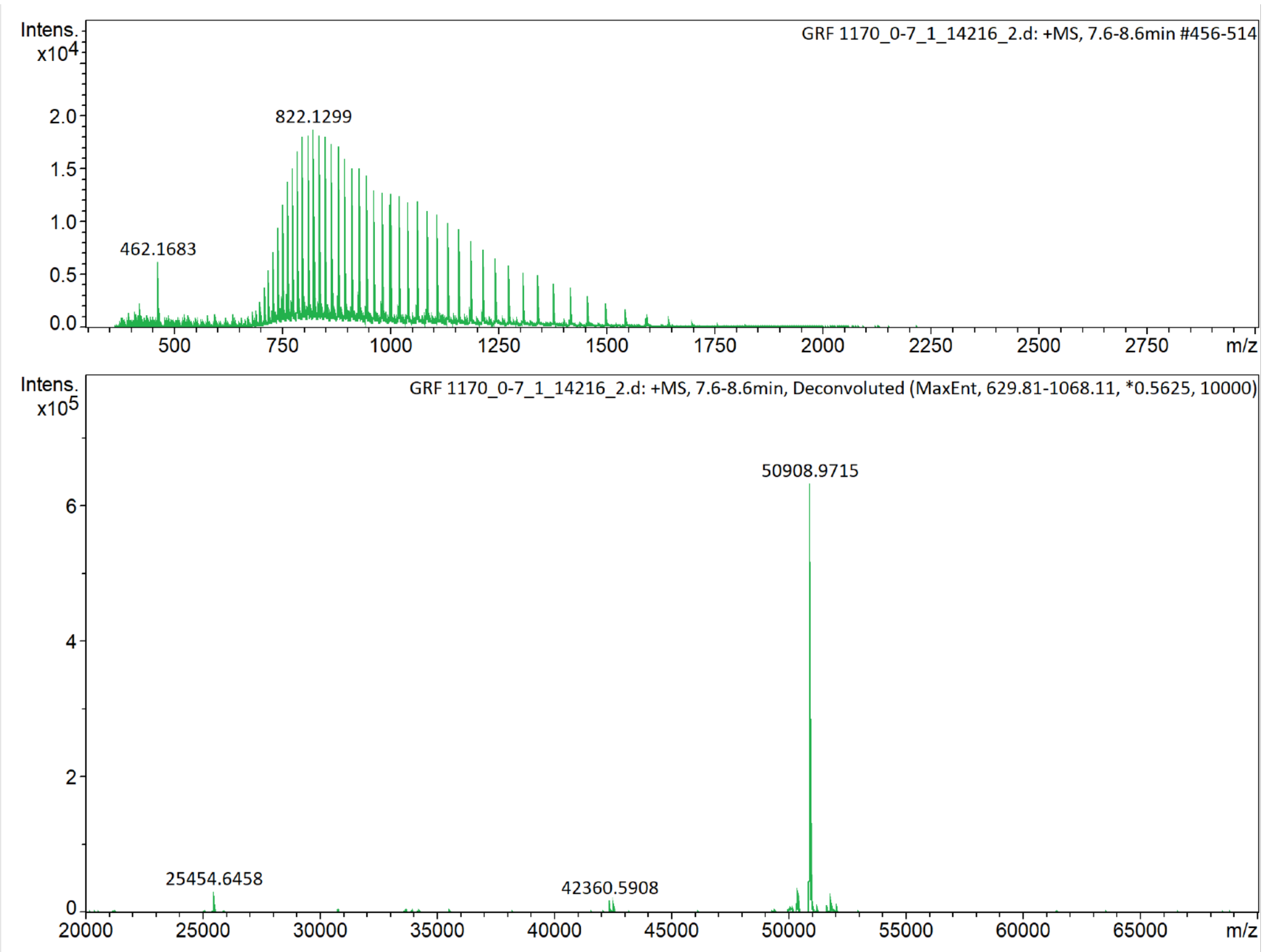

Figure S77. HRMS-ESI+ Spectrum of **Ubi K6C** in PBS buffer (50 mM, pH 7.2).

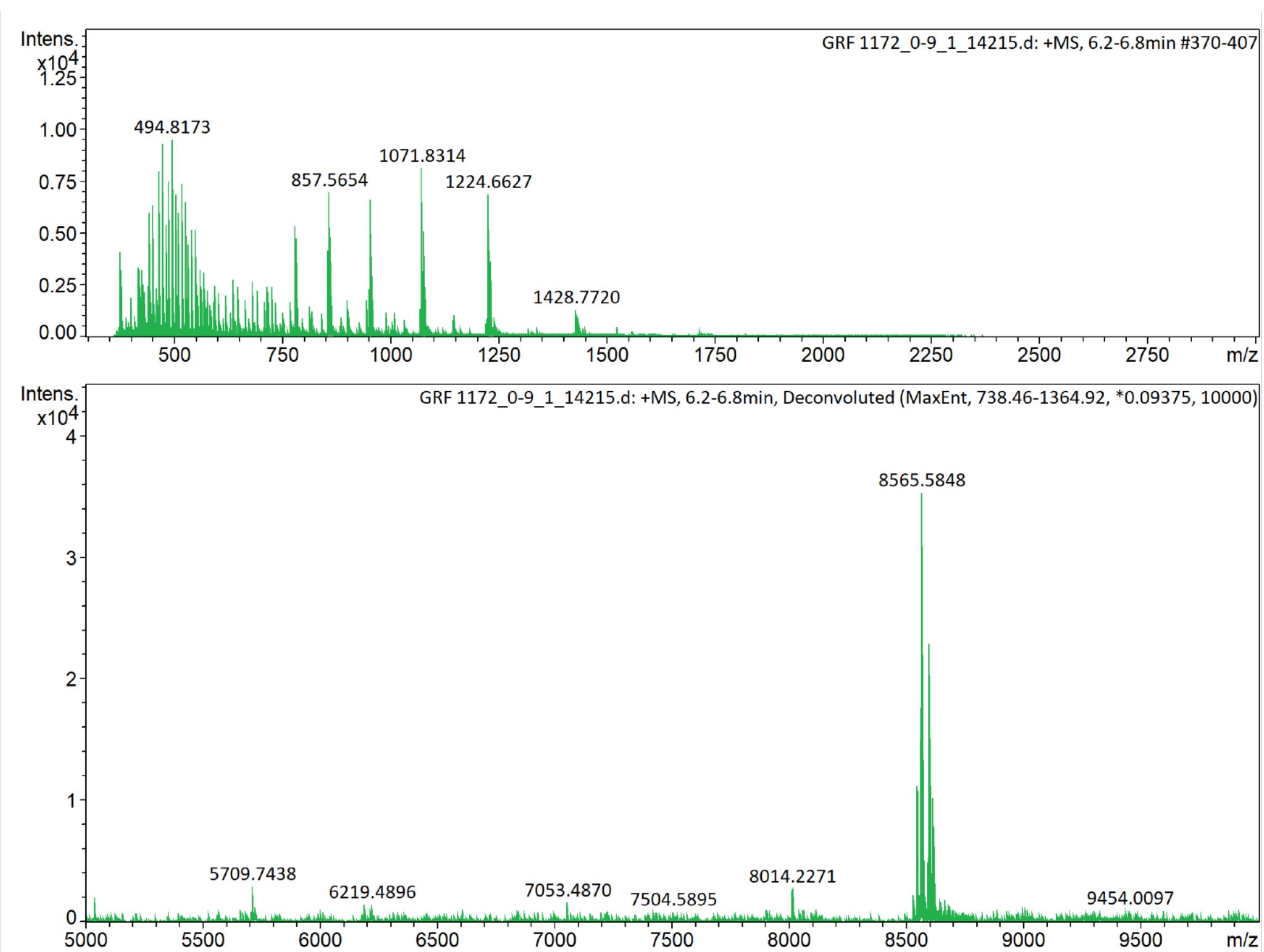

Figure S78. HRMS-ESI$^+$ Spectrum of **[Ru(^{i}Pr$_2$CAAC-5)] · MBP-Ubi K6C** in MES buffer (50 mM, pH 7.2).

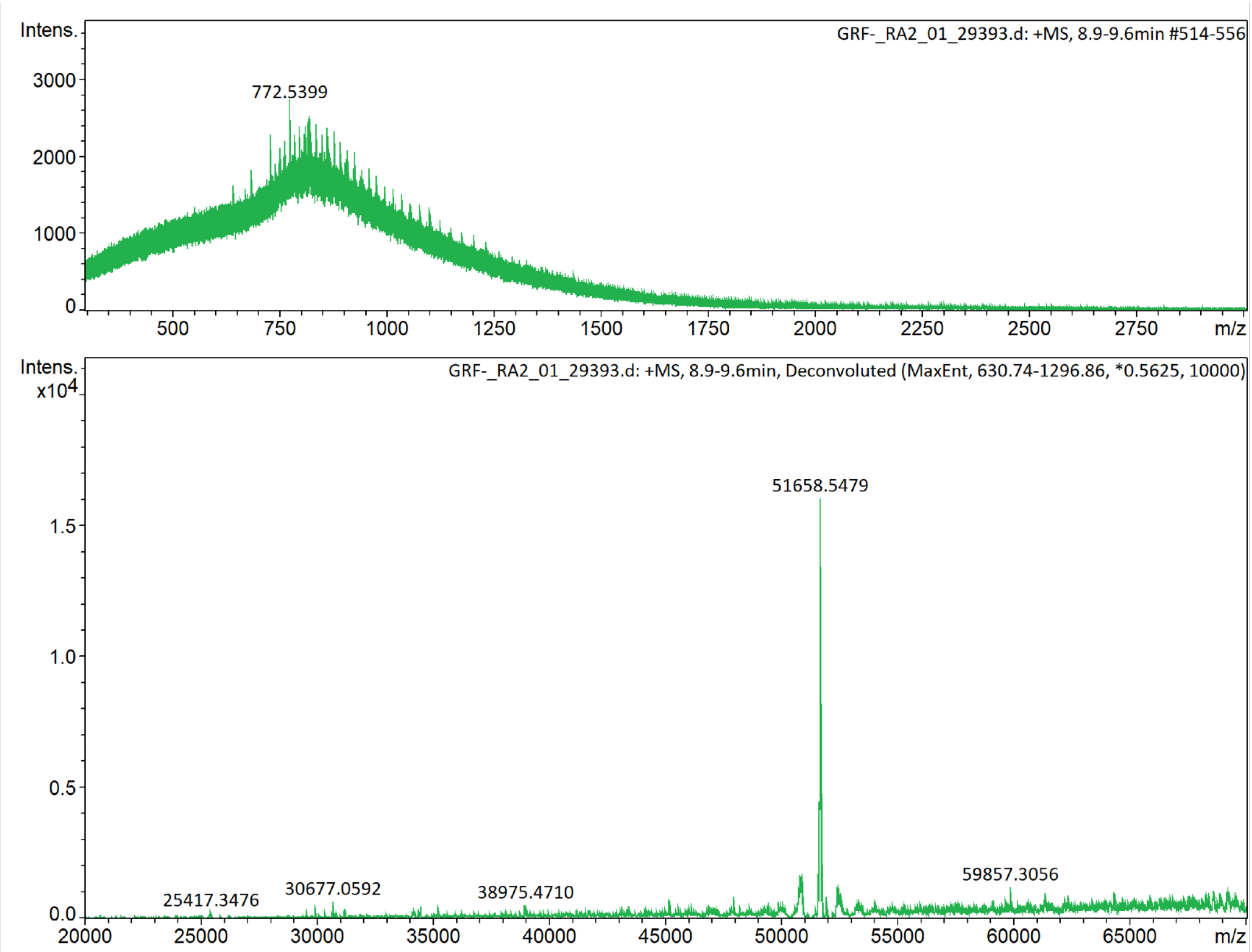

Figure S79. HRMS-ESI+ Total ion count (TIC) and extracted ion count (EIC) of **[Ru(Et₂CAAC-5-Boc)]**.

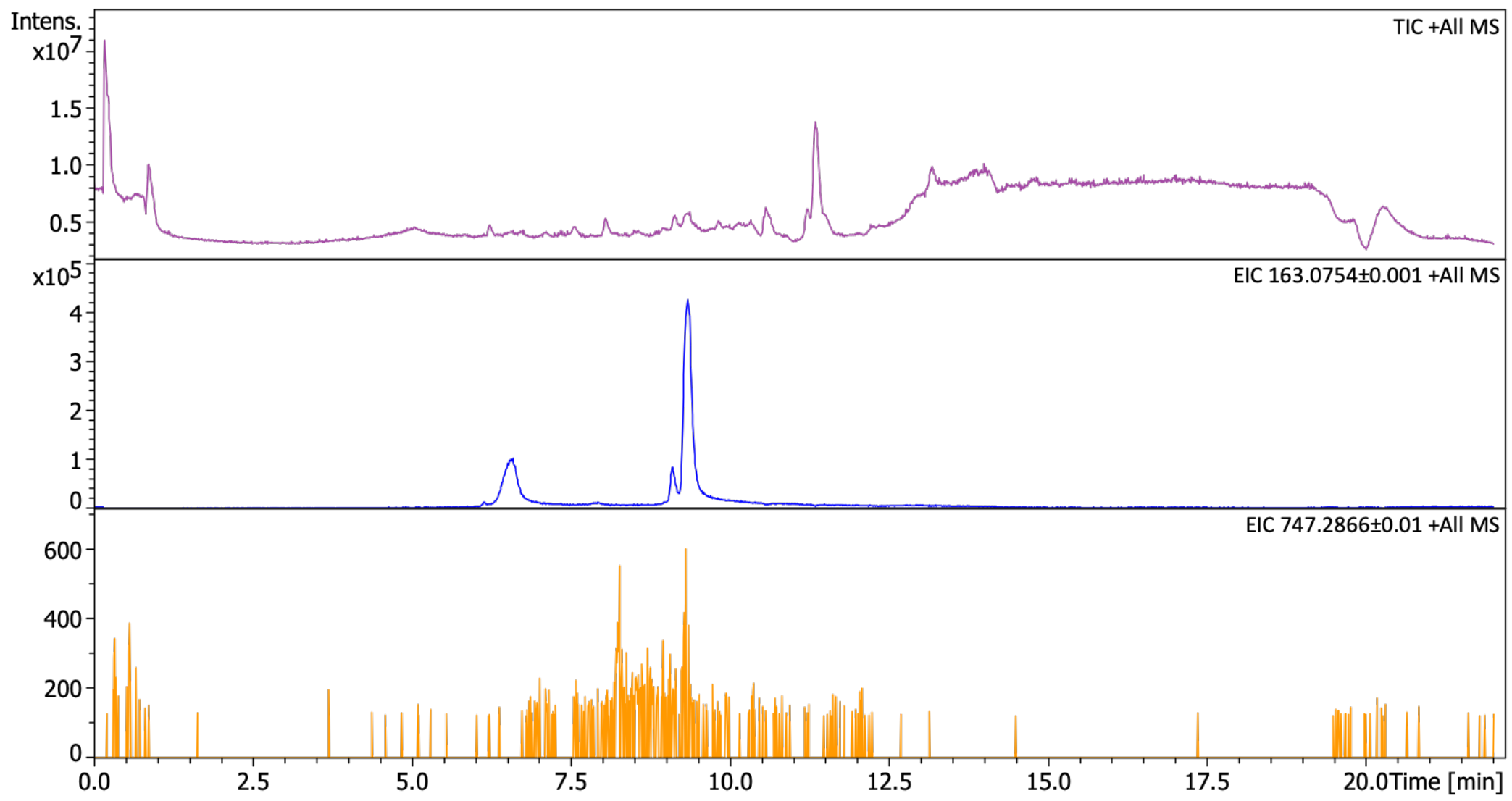

Figure S80. HRMS-ESI+ Total ion count (TIC) and extracted ion count (EIC) of **[Ru(Et₂CAAC-5-Boc)]** in the presence of tosyl diolefin **1** (10 eq.).

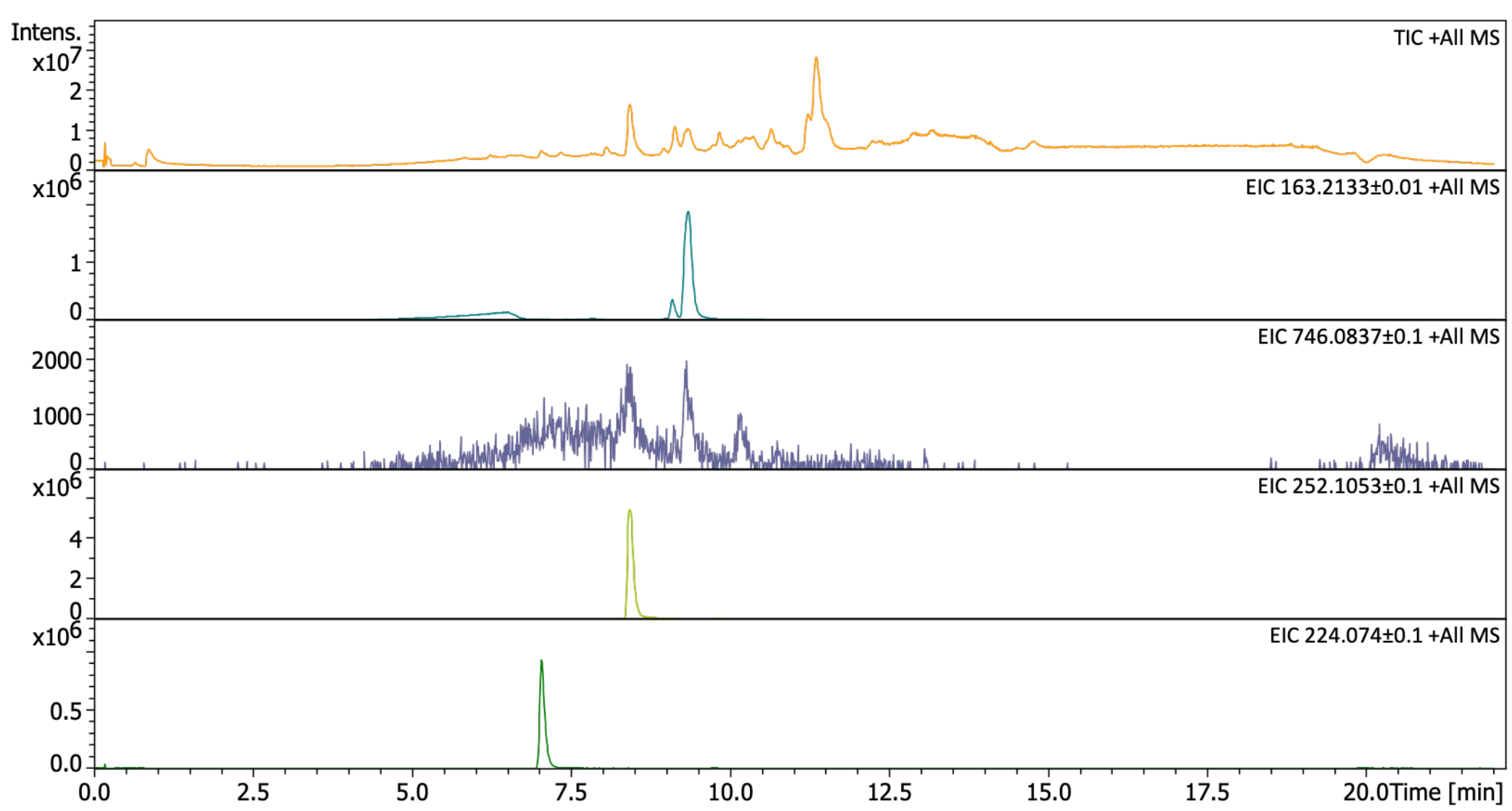

Figure S81. HRMS-ESI$^+$ Total ion count (TIC) and extracted ion count (EIC) of **[Ru(^{i}Pr$_2$CAAC-5-Boc)]**.

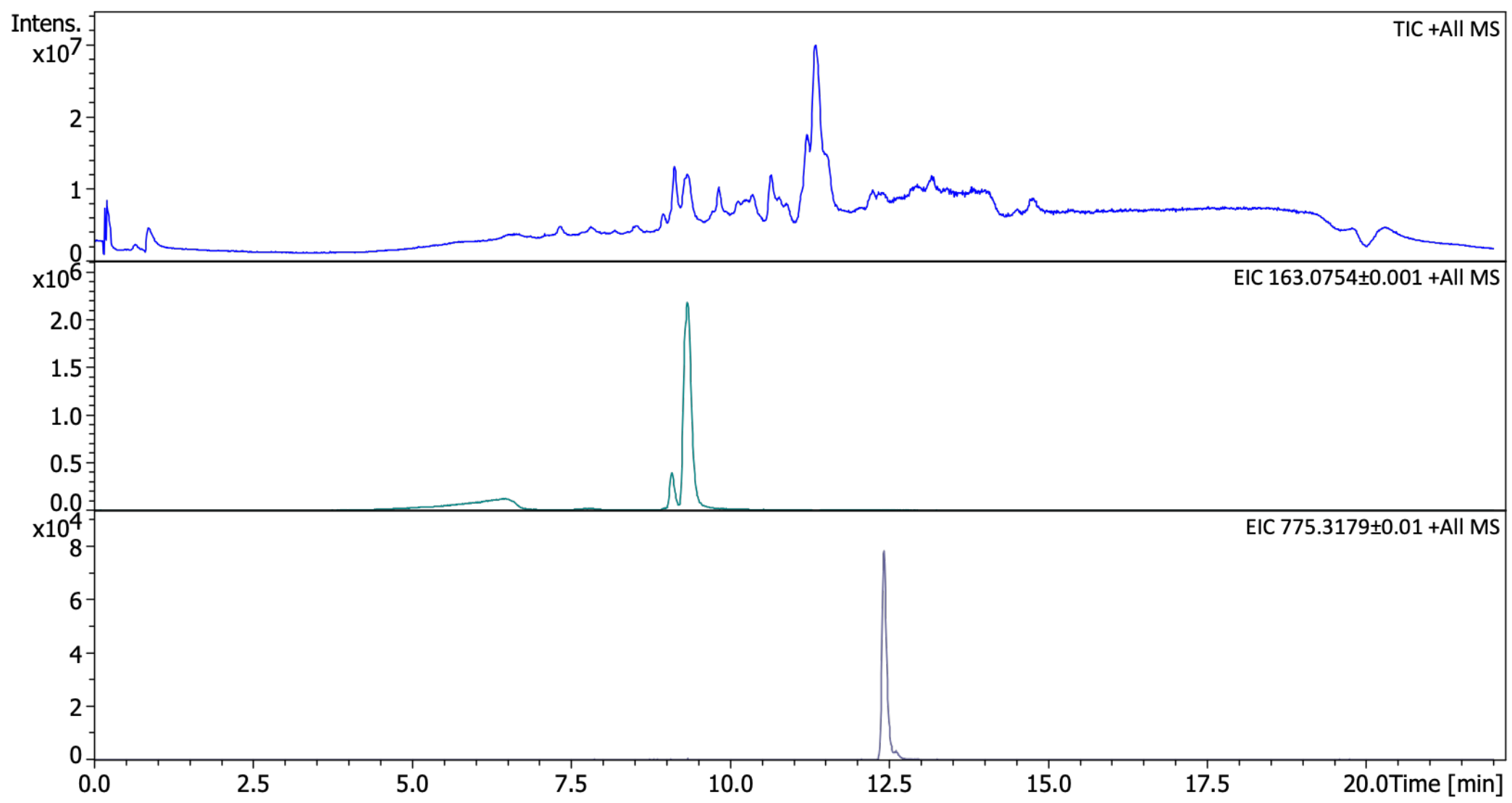

Figure S82. HRMS-ESI$^+$ Spectrum at 12.4 min of **[Ru(^{i}Pr$_2$CAAC-5-Boc)]**.

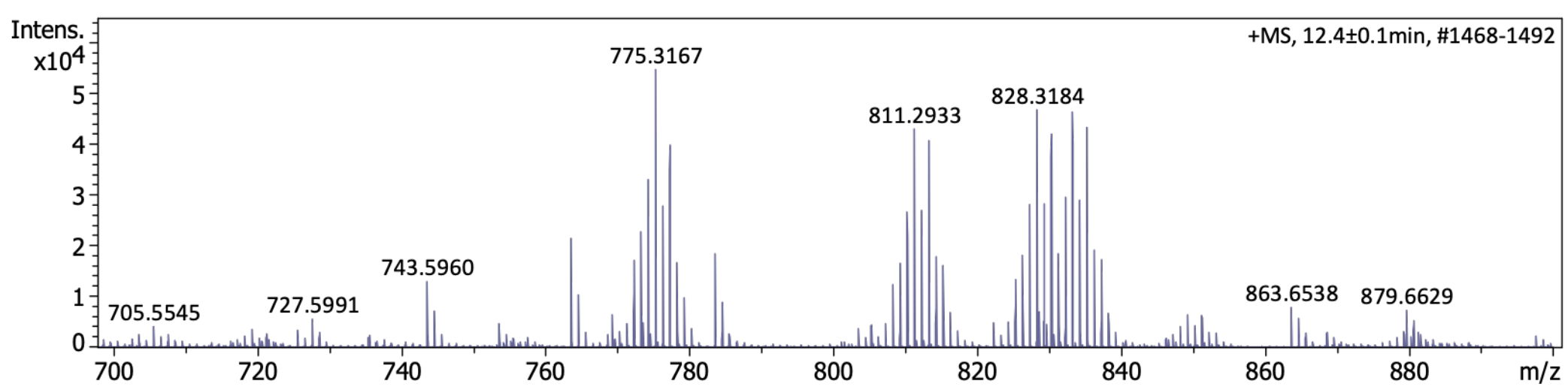

Figure S83. HRMS-ESI+ Total ion count (TIC) and extracted ion count (EIC) of **[Ru(^{i}Pr$_2$CAAC-5-Boc)]** in the presence of tosyl diolefin **1** (10 eq.).

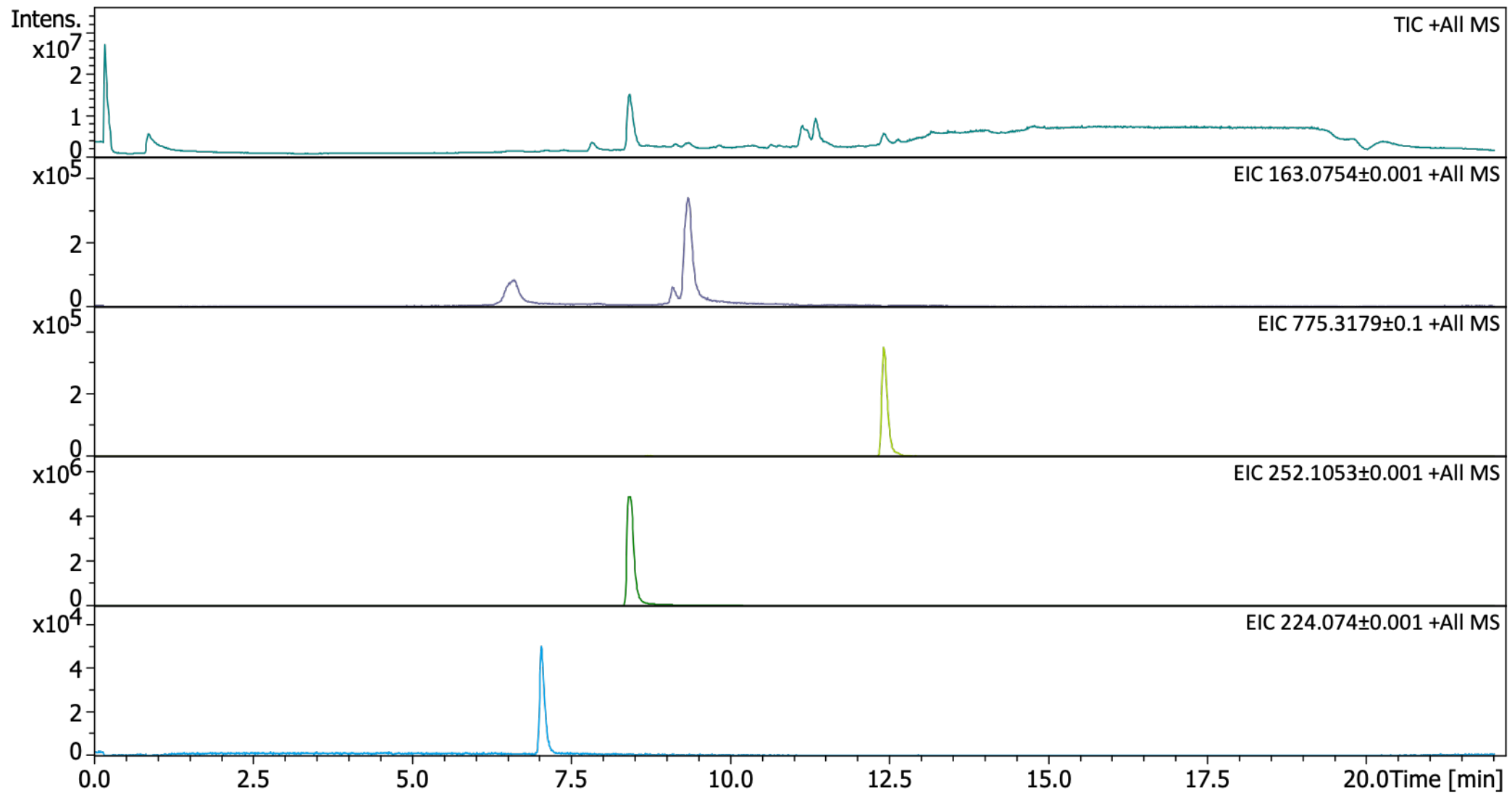

Figure S84. HRMS-ESI+ Spectrum at 12.4 min of **[Ru(^{i}Pr$_2$CAAC-5-Boc)]** in the presence of tosyl diolefin **1** (10 eq.).

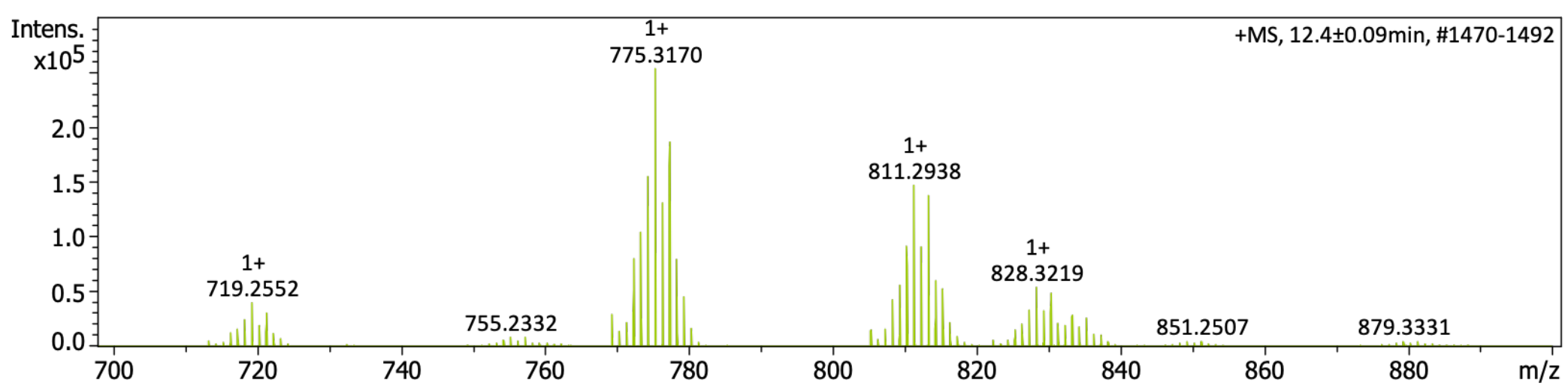

Supplement: Supplementary file 1 [file ANIE-65-e3685338-s001.pdf]
